# Comparative analysis of the *Trichoderma reesei* transcriptome during growth on the cellulase inducing substrates wheat straw and lactose

**DOI:** 10.1186/1754-6834-6-127

**Published:** 2013-09-09

**Authors:** Robert Bischof, Lukas Fourtis, Andreas Limbeck, Christian Gamauf, Bernhard Seiboth, Christian P Kubicek

**Affiliations:** 1 Austrian Centre of Industrial Biotechnology (ACIB) GmBH c/o Institute of Chemical Engineering, University of Technology of Vienna, Gumpendorferstraβe 1a, Vienna A-1060, Austria; 2 Institute of Chemical Engineering, University of Technology of Vienna, Gumpendorferstraβe 1a, Vienna A-1060, Austria; 3 Institute of Chemical Technologies and Analytics, University of Technology of Vienna, Getreidemarkt 9, Vienna A-1060, Austria; 4 Biotech & Renewables Center, Clariant GmbH, München 81477, Germany

## Abstract

**Background:**

Renewable lignocellulosic biomass is an advantageous resource for the production of second generation biofuels and other biorefinery products. In Middle Europe, wheat straw is one of the most abundant low-cost sources of lignocellulosic biomass. For its efficient use, an efficient mix of cellulases and hemicellulases is required. In this paper, we investigated how cellulase production by *T. reesei* on wheat straw compares to that on lactose, the only soluble and also cheap inducing carbon source for enzyme production.

**Results:**

We have examined and compared the transcriptome of *T. reesei* growing on wheat straw and lactose as carbon sources under otherwise similar conditions. Gene expression on wheat straw exceeded that on lactose, and 1619 genes were found to be only induced on wheat straw but not on lactose. They comprised 30% of the CAZome, but were also enriched in genes associated with phospholipid metabolism, DNA synthesis and repair, iron homeostatis and autophagy. Two thirds of the CAZome was expressed both on wheat straw as well as on lactose, but 60% of it at least >2-fold higher on the former. Major wheat straw specific genes comprised xylanases, chitinases and mannosidases. Interestingly, the latter two CAZyme families were significantly higher expressed in a strain in which *xyr1* encoding the major regulator of cellulase and hemicellulase biosynthesis is non-functional.

**Conclusions:**

Our data reveal several major differences in the transcriptome between wheat straw and lactose which may be related to the higher enzyme formation on the former and their further investigation could lead to the development of methods for increasing enzyme production on lactose.

## Background

The utilization of cellulosic and hemicellulosic polymers in plant biomass for the production of bioethanol or platform chemicals is considered as a possible strategy to reduce carbon dioxide emissions and bypass the current dependence on fossil resources. Renewable lignocellulosic biomass, besides being cheap and abundant, has also the advantage that it does not compete with food production. Currently favored raw materials for this purpose include –among others – wood residues, “energy crops” such as switch grass or *Miscanthus*, and agricultural byproducts such as wheat straw [1]. The latter is one of the most abundant low-cost sources of lignocellulosic biomass in middle European countries with an annual production of over 130 million tons [2].

For the biotechnological use of these materials, they first need to be pretreated and then hydrolyzed to their monomers. The fungus *Trichoderma reesei* is currently the major industrial producer of enzymes needed to degrade the above polymers to soluble monosaccharide [1,3]. Most of these enzymes are not formed during cultivation on monosaccharides such as glucose; the fungus must therefore be grown in the presence of an inducer which is mostly a cellulose and hemicellulose containing waste material [4]. Optimally, this would be the same material for which the produced enzymes are aimed to be applied, because this would ensure the induction of the whole spectrum of enzymes needed. However, this is often not possible because commercial producers prefer the manufacture of an enzyme preparation for a broad range of substrates.

One of the carbon sources that is used for the production of cellulases and hemicellulases by *T. reesei* is lactose, which is favoured when a soluble and cheap inducing carbon source is preferred, e.g. to facilitate and reduce costs for fermentation control and enzyme recovery [5]. However, cellulase production on lactose occurs at a slower rate and a lower final enzyme yield than on cellulosic materials, and it has also been reported to lead to an enzyme preparation with lower specific activities [6]. Yet an in depth comparison of the enzymes produced on cellulose and lactose has to our knowledge not been published so far.

Here we report a comparison of the transcriptome of *T. reesei* growing on lactose and cellulose (wheat straw), which not only demonstrates the differences in the enzymes produced but also in the molecular physiology of growth on these two carbon sources.

## Results

### Comparison of the wheat straw and lactose-regulated transcriptome of *T. reesei*

**Figure 1:**
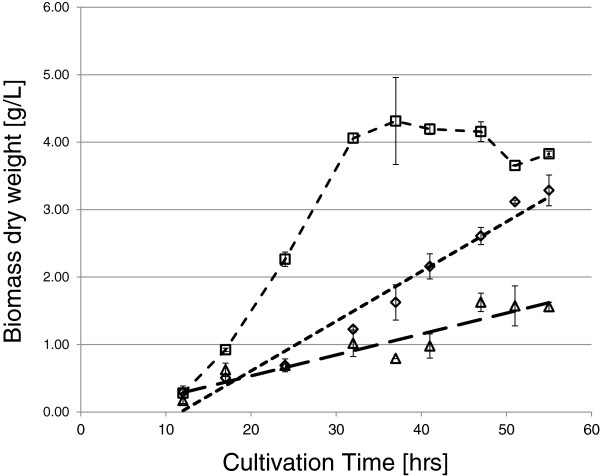
**Growth profiles of *T. reesei *QM 9414 on different substrates.** The biomass dry weight per liter was directly measured gravimetrically for glucose (squares) and lactose (diamonds) or calculated from the intracellular protein content for wheat straw (triangles) on the basis of 0.35 g intracellular protein per g dry biomass. Error bars show the respective standard deviation of three biological replicates.

As a prerequisite for this study, we examined the growth of *T. reesei* QM 9414 on wheat straw, lactose and glucose. As shown in Figure 1, growth on glucose occurred at a faster rate than on the other two carbon sources. Growth on lactose and on wheat straw occurred at slower rates, that on wheat straw being lowest. In order to compare the expression of genes that are induced in *T. reesei* on lactose and on wheat straw to that on glucose, we therefore determined the transcriptional profiles during the initial growth phase (i.e. when 25–30% of the carbon source have been consumed). We then searched for those transcripts that were >2-fold less abundant at a p <0.05 on glucose than either on lactose and wheat straw. This retrieved a total of 3120 genes, of which 2832 and 1501 were significantly upregulated on wheat straw and lactose, respectively. The significantly higher number on cellulose suggested to us that the slower growth and the complex nature of wheat straw may cause a generally more enhanced transcriptional activity. In order to test this, we examined those 1100 genes that were expressed on glucose, lactose and wheat straw at a comparable level (± 1.4-fold; Additional file 1: Table S1): they were highly enriched in the KOG groups J (Translation, ribosomal structure and biogenesis; 12.3% of all genes of this category) and K (transcription; 10.3%) (KOG numbers taken from http://genome.jgi-psf.org/cgi-bin/kogBrowser?db=Trire2). The 1100 genes also contained several housekeeping genes such as *tef1* encoding elongation factor 1-α and *act1*, encoding actin [7]. Interestingly, *sar1*, encoding a small protein involved in the secretory pathway, which was recommended as the most reliable housekeeping gene [7] exhibited > 2-fold upregulation on wheat straw, probably because of the general upregulation of secretory genes under these conditions.

Within the 3120 genes, five patterns (further called “transcript groups”) could be distinguished: 1213 genes were significantly upregulated both on wheat straw and on lactose (Table 1); 1619 genes were upregulated only on wheat straw (transcript group A), in contrast to only 288 genes that were upregulated on lactose (transcript group B) only. Among the 1213 genes, 344 were significantly stronger upregulated on wheat straw (transcript group C) and 65 on lactose (transcript group D; Table 1), whereas 804 were equally strong upregulated on wheat straw and lactose (transcript group E). 1288 and 238 of the 3120 genes encoded unknown or orphan proteins, respectively. For a complete list of these genes see Additional file 2: Table S2.

**Table 1:** **Overview of the *T. reesei *** transcriptome on wheat straw (W) and lactose (L)*

			**Wheat straw**	**Lactose**	**Wheat straw and lactose**		
					**W > L**	**L > W**	**W = L**
**Transcript category**			**A**	**B**	**C**	**D**	**E**
Gene category		FunCat					
All genes			1619	288	344	65	804
unknown genes			743	117	98	21	309
orphans			90	44	18	5	81
Metabolism		01					
	glycolysis	02.01	10		1		10
	PPP	02.07	1	2			1
	TCA cycle	02.10	4		2		5
	pentose catabolism	NN**	1	1	1		2
	gluconeogenesis	NN***			1	5	
	amino acids	01.01	17	8	6	3	15
	nucleotide	01.03	6		1	4	
	fatty acids	01.06.01	35	6	6	3	22
	phospholipids	01.06.03	15				6
	secondary metabolism	01.20	8	3			
Transport							
	MFS	20.01.03	28	7	28	3	46
	nitrogenous compounds	20.01.07, 20.01.09	4	5	2	3	8
	ions	20.01.01	6	1	1		5
	ABC transporters	20.03.25	4	2		1	4
	mitochondrial transport	20.09.04		1			1
	aquaporins	20.03.01			1		4
	iron transport and reduction	20.01.01.01	6		6		1
Transcription							
	transcription factors	11.02.03.04	98	16	18	3	57
	DNA	10.01	76		4		17
Cell cycle		10.03	13	2	2	1	9
Translation		12.04	7	1	1		1
Secretion		20.09.16	35	1	1		1
Signal transduction							
	G-protein signalling	30.01.05.05	6	1	4	3	9
	protein kinases/phosphatases	30.01.05	27	1	1		3
Extracellular products							
	SSCPs	NN****	39	6	12	1	18
	glycosyl transferases	14.07.02	5	1	2		1
Hydrolytic enzymes		01.25.01					
	CAZys		40	7	54	4	27
	proteases		26	6	8		8
	lipases		11	2	3	1	7
	nucleases		9		2	1	
	amidases and nitrilases		8	2	2		4
Oxidative enzymes		32.07					
	cytochrome P450 monooxygenases		11	1	5		12
	FAD-dependent monooxygenases		4		5		2
	dioxygenases		4	2	1		4
	multicopper oxidases		3		4		3
	peroxide/superoxide metabolism		7		1	1	4

*Only genes that are at least 2-fold upregulated with respect to glucose (p < 0.05) are considered.

**Not noted in FunCat; refers to enzymes involved in the fungal pentose catabolic pathway.

***FunCat combined gluconeogenesis and glycolysis; here we list genes specific only for gluconeogenesis (and which were consequently left out from glycolysis).

****Small secreted, cysteine-rich proteins; not listed in FunCat.

**Figure 2:**
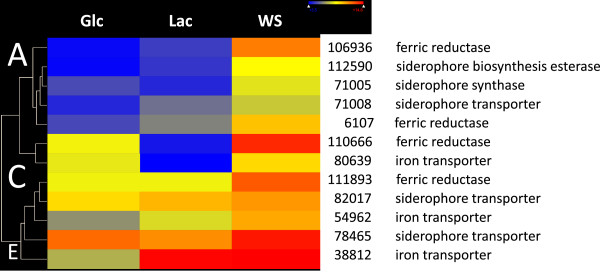
**Hierarchical cluster analysis of expression of genes related to iron homeostasis. Glc, glucose; Lac, lactose; WS, wheat straw.** Data are shown as a heat map, and the color code of respective expression values (dark blue: 0; dark red: 16; numbers indicate the log_2_ of the mean expression level, n = 2). Numbers and names indicate the respective Trire2: number and putative gene function. The letters at the major branches specify the transcript groups, as defined in the text.

When the percentage of individual gene groups (defined as FunCat categories; [8]) in the total number of genes in the individual group was compared, transcript group A displayed some unique features (Table 1): it was strongly enriched in Funcat categories for phospholipid metabolism, iron homeostasis, secretion, protein kinases/phosphatases and DNA repair. The genes related to iron homeostasis comprised four ferric reductases, three iron transporters, three siderophore transporters, and two enzymes involved in the biosynthesis of siderophores, including one of the two siderophore synthases (Trire2:71005; [9]; Figure 2). Genes for gluconeogenesis were significantly stronger expressed on lactose than on wheat straw. All other Funcat categories revealed no significant differences between the five transcript categories.

The significant differences in expression of genes related to iron homeostasis prompted us to check whether wheat straw would bind iron and thus decrease its bioavailability to *T. reesei*. As shown in Additional file 3: Table S3, this was found to be indeed the case: although the same concentration of FeSO_4_*7H_2_O had been added to both media (5 mg/L), the supernatant of the wheat straw medium contained only 16% of it prior to inoculation. Until the time of harvesting the mycelia for transcriptome analysis the cultures on wheat straw consumed 0.21 ppm of the available iron, whereas the lactose culture only consumed 0.06 ppm, which correlates well with the higher expression of the iron homeostasis genes on wheat straw.

### The *T. reesei* secretome on wheat straw and lactose

**Figure 3:**
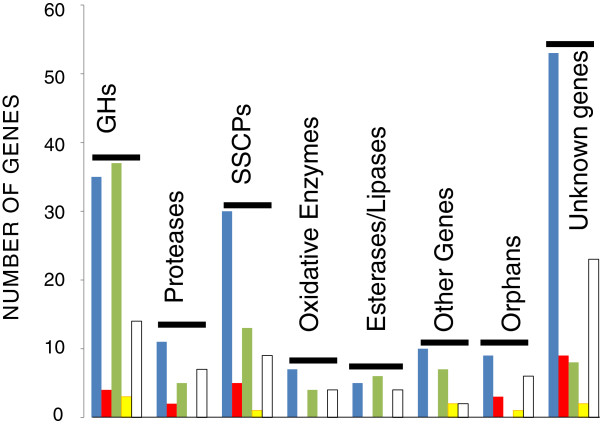
**At least >2-fold upregulated genes encoding putative secreted proteins during growth of *T. reesei *on wheat straw and lactose.** Different transcript groups (for explanation see text) are: transcript group A, blue; transcript group B, red; transcript group C, green; transcript group D, yellow; and transcript group E, white. Esterases and lipases include both carbohydrate esterases as well as lipases.

We also examined how many of the genes found in transcript groups A-E would encode secreted proteins. Druzhinina et al. [10] have recently *in silico* identified 747 genes for proteins that are secreted by *T. reesei* into the medium. 341 of these genes were indeed found to be significantly transcribed under at least one of the present conditions, of which 160 were only expressed on wheat straw. CAZys and unknown proteins comprised the major portion (93 and 95, respectively), followed by small, secreted cysteine rich proteins (58), and all three were most abundant in transcript group A (Figure 3). Proteases, lipases and oxidative enzymes were also detected but only in much smaller numbers (25, 15 and 15, respectively). It is also of interest that transcript groups B and D (i.e. genes expressed either only on lactose, or at high abundance on it) lacked such coding for oxidative enzymes and lipases (Figure 3).

### Common and unique features of the wheat-straw and lactose-induced CAZome

**Figure 4:**
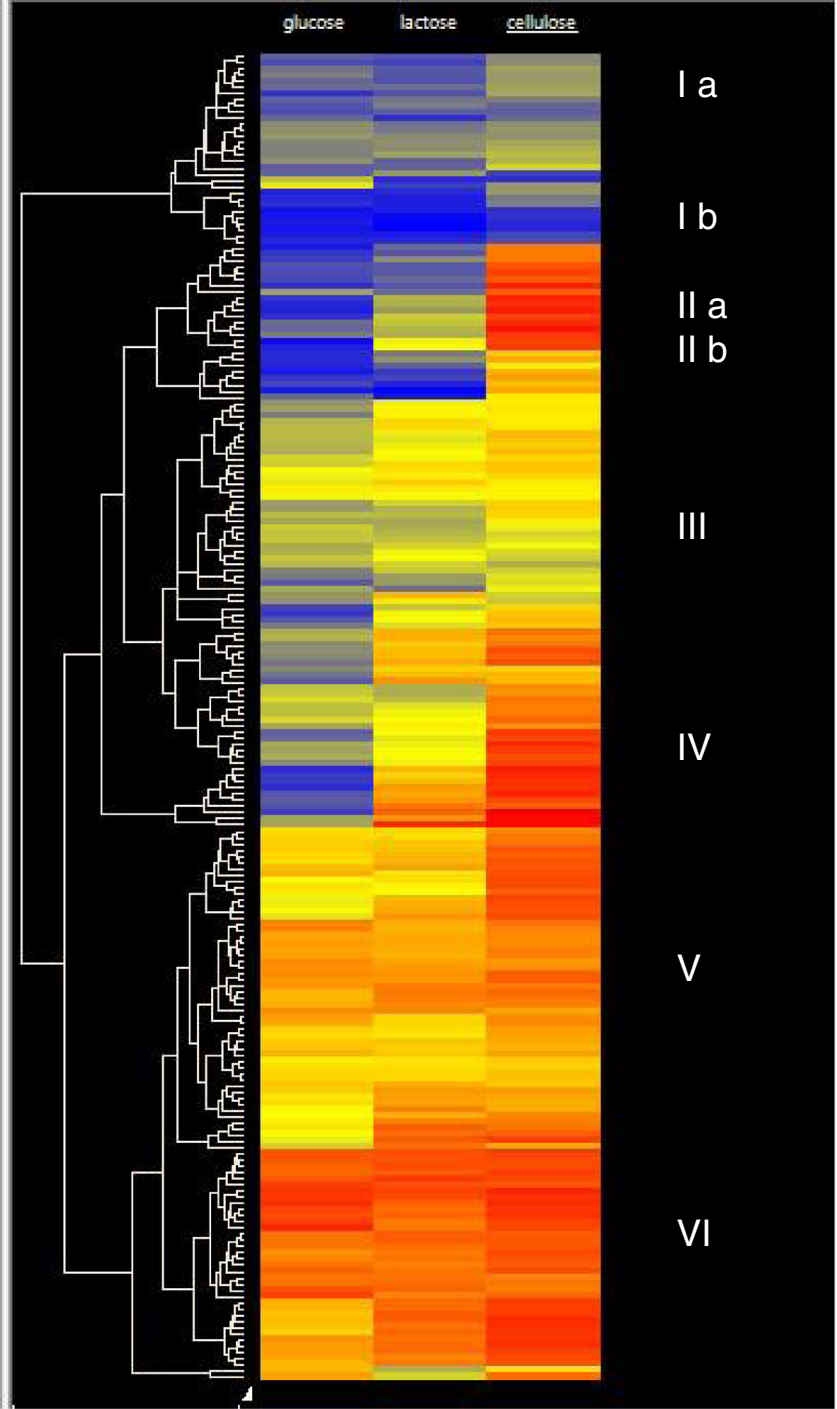
**Hierarchical cluster analysis of glycosyl hydrolase gene expression.** Data are shown as a heat map, and the color code of respective expression values (dark blue: 0; dark red: 16; numbers indicate the log_2_ of the mean expression level, n = 2). Roman numbers and (in certain cases) lower case letters specify clades characterized by consistent expression patterns.

Häkkinen et al. [11] have recently revised and expanded the repertoire of CAZys in *T. reesei*, which resulted in 210 genes encoding glycosyl hydrolases, carbohydrate esterases and carbohydrate binding proteins. A hierarchical cluster analysis with all of them illustrates that their expression on wheat straw, lactose and glucose falls into several categories (indicated by clusters; Figure 4 and Additional file 4: Table S4): they comprise genes not expressed at all or – if so only weakly on wheat straw (I a), genes much stronger expressed on wheat straw than on lactose (clusters II a and b), but also genes that are expressed on all carbon sources (cluster VI). As noted above, 132 of these genes fulfilled our criterion of at least 2-fold increased expression on wheat straw versus glucose and at least 2-fold on lactose versus glucose, respectively. This number is higher than that of secreted CAZymes, which is due to the fact that several of the upregulated α- and β-glycosidases lacked a signal peptide and apparently represent intracellular enzymes.

A detailed analysis showed that the majority of genes for cellulases, cellulose monooxygenases and cellulose binding proteins were > 2-fold stronger expressed on wheat straw than on lactose (Table 2). This included almost all of the cellulases with a CBM1 cellulose binding domain, and also the auxiliary proteins swollenin, CIP1 and one GH61 polysaccharide monooxygenase. Only the cellobiohydrolase 1-encoding gene *cel7a*, as well as two GH1 and one GH3 β-glucosidase genes (*cel1a, cel1b* and *bgl3j*) and two GH61 polysaccharide monooxygenases were equally expressed on wheat straw and lactose, and one other polysaccharide monooxygenase was only expressed on wheat straw. A contrasting picture was obtained for the GH10, GH11 and GH30 xylanases, half of which were only expressed on wheat straw. In contrast, most of the genes encoding enzymes that cleave hemicelluloses side chains (α-L-arabinosidases, α-(methyl)-D-glucuronidases, α-D-fucosidases and polysaccharide deacetylases) were equally well expressed on lactose and wheat straw (transcript group E). Finally, it was noted that the presence of wheat straw (transcript groups A and C) also specifically induced an array of GH18 chitinases –particularly such that also contain a cellulose binding domain (CHI18-14, CH18-16, and CHI18-17 [12]) GH2 and GH47 β-D-mannosidases and GH55 endo-β-1,3-glucanases.

**Table 2:** **Glycosyl hydrolases and auxiliary enzymes or proteins that are significantly expressed in *T. reesei *** **on wheat straw and/or lactose**

			**Wheat straw**	**Lactose**	**Wheat straw and lactose**		
					**W > L**	**L > W**	**W = L**
**Transcript category**			**A**	**B**	**C**	**D**	**E**
All CAZYs			40	7	54	4	27
cellulases	cellobiohydrolases	GH6			1		
		GH7					1
	endo-β-1,4-glucanases	GH5			1		
		GH7			1		
		GH12			1		
		GH45			1		
	β-glucosidases	GH1					
		GH3			7		2
	swollenin, CIP1				2		
	polysaccharide monooxygenase	GH61	1				2
hemicellulases	endo-β-1,4-xylanases	GH10	1		1		
		GH11	2		1		
	exo-β-1,4-xylanases	GH30	1		2		
	β-xylosidases	GH3	2		1		
	xyloglucanase	GH74			1		
	α-L-arabinofuranosidases	GH43	1		1		
		GH54			2		
		GH62			1		
	α-D-galactosidases	GH27	1		2		4
		GH36					1
	α-D-fucosidases	GH95			1		2
	β-D-mannanases	GH5	1				
	β-D-mannosidases	GH2	1		3		1
		GH38					1
		GH47	3				1
		GH76			2		1
		GH92			1		2
β-glucanases	endo-1,3/1,4-β-glucanase	GH16	2		1		1
	endo-1,3-β-glucanase	GH55	4		1		1
	endo-1,3-β-glucanase	GH64	1		1		
	β-1 3-glucanosyltransferase	GH72	1	1			
	endo-1,3-β-glucanase	GH81	1				
polygalacturonases	exo-polygalacturonase	GH28	1				
	endopolygalacturonase	GH28			1		
	exo-rhamnogalacturonase	GH28			1		
	α-L-rhamnosidase	GH78	1				
chitinases	exo-β-D-glucosaminidase GLS93	GH20			1		
	endochitinases	GH18	4		3		
	N-acetyl-β-glucosaminidases	GH20	1		1		
	chitosanases	GH75		1	1		
	N-acetyl-β-galactosaminidases	GH89	1				
carbohydrate binding proteins		CBM13				1	
		CBM18				1	
α-glucan hydrolases	α-D-1,4-glucosidases	GH13	1				1
	glucamylase	GH15	1				
	α-D-1,4-glucosidases	GH31			3		
	α,α'-trehalase	GH37				1	
	α,α'-trehalase	GH65					2
	α-D-1,3-glucosidases	GH71	2				1
carbohydrate esterases		CE1	2				
		CE4	1		1		
	acetyl xylan esterase	CE5			3		
	acetyl esterase	CE16					1
	chitin deacetylase		1				
α-glucuronidases		GH67			1		
		GH79					1
		GH105	1		1		
		GH115			1		1

### Chitinases, mannanases and galactosidases are negatively regulated in a strain in which the cellulase regulator XYR1 is nonfunctional

**Figure 5:**
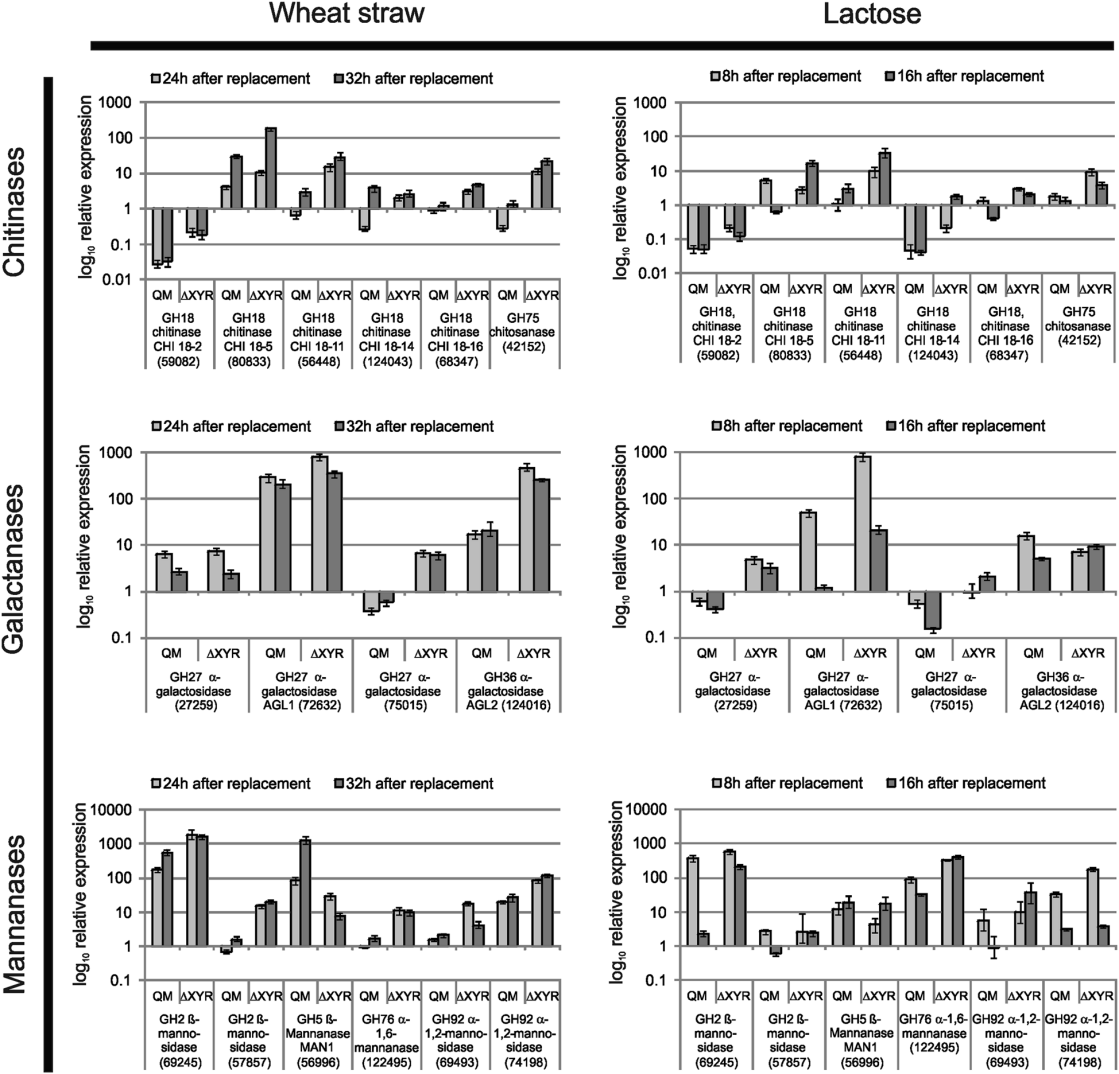
**Results of qPCR analyses of those chitinases, galactanases and mannanases that were upregulated on either lactose, wheat straw or both carbon sources.** The expression of the lactose samples and wheat straw samples, taken at the indicated time points (see legend) is related to that of an 8 h sample from a QM 9414 glucose culture and normalized on *tef1*. Error bars show the standard deviation of two independent experiments.

The polysaccharides present in pretreated wheat straw are mainly cellulose and (a small amount of) xylan [11]. Yet the above data (Table 2) have shown that several genes not associated with degradation of cellulose or xylan, such as chitinases, mannosidases or α-D-galactosidases, are specifically or higher induced by wheat straw. Since the genes encoding the cellulose and xylan depolymerising enzymes are regulated by the Zn2Cys6 transcription factor XYR1 [13], we also wanted to learn whether transcription of these other differentially expressed genes is also controlled by XYR1. To this end, we examined their expression on wheat straw both in *T. reesei* strain QM 9414 as well as in a *∆xyr1* knock-out strain. The results are shown in Figure 5: while qPCR confirmed the induced expression of these genes in QM 9414, we found that almost all of them – with the only clear exception of the β-mannanase encoding gene *man1* – were 2- to >100-fold overexpressed in the *∆xyr1* mutant. Most notably this was true for all the tested chitinases, while there were one and two ambiguous cases for the mannanases and galactosidases respectively. The significance of these results was also tested by an unequal variance *t*-test and the respective result are reported in Additional file 5: Table S5.

This finding prompted us to test whether the 5’-upstream nontranslated sequences of these chitinase, mannanase and galactosidase genes would bear consensus sites for binding of XYR1 (GGCW_4_). Furukawa et al. [14] assessed that the whole genome of *T. reesei* contains 20692 XYR1 consensus binding sites, which – in view of the estimated 33 Mbp of the *T. reesei* genome [15] – implies that on the average one binding site may occur about every 1500 bp. When this value is compared to the number of consensus sites in four major cellulase genes *cel7a, cel7b, cel6a* and *cel5a*, they all contain >10 consensus sites in the first 1500 5’ bp upstream of the ATG [14]. A similar analysis for the 16 chitinase, α-galactosidase and α-mannanase/mannosidase genes that were analyzed by qPCR (Figure 5) revealed a broad range of number of consensus sites within 1000 bp upstream of their start codon, ranging from 6 (MAN1) to 0 (for two chitinases and one mannosidase; Additional file 6: Table S6). The significance of the number of consensus sites versus the mean statistical occurrence (every 1500 bp) was rejected by the Anderson-Darling test [16].

### Wheat straw induces the expression of genes involved in autophagy

**Figure 6:**
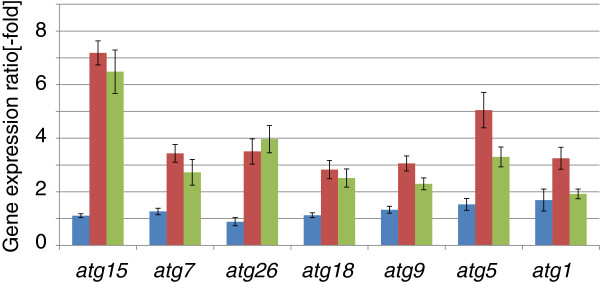
**Changes in gene expression of autophagy related *T. reesei *genes on glucose, lactose and wheat straw: blue bars show the expression of lactose (Lac) cultures relative to glucose (Glc) cultures, red bars show the expression of wheat straw (Wheat) cultures relative to glucose (Glc) cultures, and green bars show the expression of wheat straw (Wheat) cultures relative to lactose (Lac) cultures.** Values represent the mean of three biological replicates, error bars show the standard deviation.

The above described upregulation of chitinases could be a consequence of enhanced autophagy. This term specifies an intracellular degradation process functioning in the delivery of cytoplasmic proteins and organelles to vacuoles for macromolecule turnover and recycling [17,18]. To investigate this possibility, we screened for the potential of genes known to be involved in fungal autophagy in *T. reesei* growing on wheat straw and lactose. Indeed, as shown in Figure 6, we found 7 autophagy genes (*atg1, atg5, atg7, atg9, atg15, atg18* and *atg26*) to be significantly upregulated on wheat straw, but not on glucose or lactose.

## Discussion

We have previously described that lactose can induce an almost complete cellulase and hemicellulase enzyme system in *T. reesei*, and reasoned that this could be due to a preference of the fungus to initiate feeding on lignocellulose by hydrolysing the β-galactoside side chains in the xyloglucans, which are linked to cellulose in the primary cell wall of dicotyledons [19]. In order to test how this induction on lactose would compare to a complex lignocellulosic material – wheat straw, which not only contains cellulose but also xylans but with very little galactose side chains (< 0.1%; [11]) – we compared the transcriptome under both conditions. The results showed that 85 of the 132 genes of the CAZome were expressed both on lactose as well as on wheat straw. However, two thirds of them were significantly stronger expressed on wheat straw than on lactose. One of the major qualitative differences between wheat straw and lactose was a strict dependence of 4 xylanases and 2 β-xylosidases on wheat straw for expression, implying that xylanases are only poorly expressed on lactose. Only *xyn2* and *xyn3* were also expressed on the latter. These findings suggest that there is indeed no major difference between the induction of cellulolytic enzymes by lactose and cellulose, but that the observed differences are due to the content of xylan. Expression of x*yn2* has previously been demonstrated to be triggered by both sophorose (which is considered to be a “cellulose-specific” inducer) as well as xylobiose (considered to be “xylan-specific”) and by lactose [20]. However, *xyn1, xyn2*, *xyn4* and *xyn5* have recently also been shown to be differentially induced by D-xylose and L-arabinose [21]. Similarly, Akel et al. [22] have shown that the α-L-arabinofuranosidase genes require the presence of L-arabinose for full induction. We therefore interpret the significantly higher expression of most of the xylanases and hemicelluloses side chain hydrolases to be due to the presence of additional specific inducers for their genes.

These results raise an interesting question: it has been demonstrated that expression of the cellulase and hemicellulase genes in *T. reesei* is completely dependent on the function of the Zn2Cys6-transcription factor XYR1 [13,22], and *xyr1* itself is induced both on lactose [19], and cellulose [23]. So how could a single transcription factor respond to different inducers in quantitative different ways? The regulation of genes encoding xylanolytic enzymes of the model organism *Neurospora crassa* has been suggested to involve several regulatory groups: the xylanase regulator XLR-1 (the *N. crassa* orthologue of XYR1) was suggested to work alone or in combination with other unknown regulators and an XLR-1 independent group of genes was also suggested to exist [24]. Häkkinen et al. [11] have also hypothesized that several regulatory mechanisms, depending on the inducers present, may act on the CAZyme gene promoters simultaneously, and in some cases also in an additive manner. An example of such an additional regulator could be ACE2, which has been demonstrated to assist xylanase gene transcription by enhancing *xyr1* transcription and by forming a putative heterodimer with XYR1 [25], whereas it has only a small effect on induction of cellulase gene transcription by solka floc cellulose, and none at all when sophorose is used as an inducer [26]. Another candidate could be the orthologue (Trire2: 26163) of the recently described *N. crassa* cellulase regulator CLR-2 [27]. In this study, *clr2* was found to be induced during growth on lactose or wheat straw. A blastp search with the *T. reesei* CLR2 protein sequence against the NCBI database revealed that it is an orthologue of the *A. nidulans* mannanase regulator ManR, for which the DNA-binding motif has been determined as 5’-YAGAAT-3’ [28]. However, a search of the presence of this motif in 1 kb upstream of several CAZome genes that were found to be significantly regulated in this study revealed no consistent picture: 1–2 copies were present in some genes, but completely absent in the majority of them, including the major β-mannanase gene *man5* (unpublished data). Whether or not CLR2 or another transcription factor cooperate with XYR1 in the expression of some CAZome genes therefore remains to be determined. We should like to stress that both *xyr1* and *clr2* belonged to transcript group E, i.e. they were expressed to similar levels on lactose and wheat straw. The different level of expression of various genes on wheat straw and lactose can therefore not be simply the result of an enhanced expression of *xyr1*.

In this study, we also detected a significant upregulation of genes encoding chitinases, α-galactosidases and mannosidases*.* These genes were also recently observed to behave different from the major set of cellulase and hemicellulase genes by being moderately or even very low expressed during growth on birch xylan, steam exploded and enzymatically treated bagasse already at the early time points [11]. Interestingly, this upregulation was strongly enhanced in a strain in which the *xyr1* gene had been deleted, indicating that XYR1 is a repressor of these genes. While the XYR1 orthologues from *A. oryzae, N. crassa* and *Fusarium graminearum* all function in the regulation of xylanase gene expression, XYR1 regulates cellulase gene expression only in *T. reesei* and *A. oryzae.* In both *N. crassa* and *A. oryzae –* the two fungi in which the effect of *xyr1* manipulation has been studied on a genome-wide scale [24,29] - it has so far been shown only to activate gene expression. However, the variation of occurrence (and in 3 cases even absence) of XYR1 binding sites in the promoters of these genes makes an action of XYR1 as a repressor of these genes unlikely. We consider it rather possible that their strong upregulation in the ∆ *xyr1* mutant is due to its inability to grow on wheat straw and the reduced growth on lactose [30], i.e. autophagy. In fact, upregulation of some of the *T. reesei* chitinases by carbon starvation has been shown [12], but is so far not known for the α-mannanases/mannosidases or α-galactosidases. Since oligosaccharides with these monosaccharide and linkage types are not part of the cell wall polymers of the fungus [31], their induction under starvation requires further studies.

A comparison of the wheat straw and lactose transcriptome did not lead to the detection of major changes in metabolic pathways and the signaling to them with four exceptions: one was a high number of genes associated with phospholipid metabolism and protein secretion, with DNA replication and repair, and finally the massive upregulation of genes for iron homeostasis. As for the first, Schreiber et al. [32] showed that the addition of the phospolipid precursor cholin increased cellulase formation in *T. reesei*, and at the same time led to an increase in the hyphal content of endoplasmic reticulum. Also, Glenn et al. [33] showed that the hypercellulolytic mutant *T. reesei* RUT C30 exhibits a proliferated amount of endoplasmic reticulum. It is thus possible that the increased expression of genes associated with phospholipid synthesis is responsible for an enhanced synthesis of endomembrane components required for increased cellulase export from the hyphae.

Another group of upregulated genes was those related to autophagy. To date, more than 30 autophagy-related (ATG) genes have been identified for *Saccharomyces cerevisiae* and other fungi [34,35]. BLASTP search of the predicted *T. reesei* proteins (http://genome.jgi-psf.org/Trire2/Trire2.home.html) against the NCBI database (at a cutoff of E-value < −70 over at least 80% of the sequence length) detected 22 orthologues of ATG genes (see Additional file 7: Table S7) of which 7 were found to be strongly upregulated on wheat straw but not on lactose or glucose. Notably this group included the serine/threonine protein kinase Atg1 [17,18], which – together with Atg17, Atg29, and Atg31 – forms a protein complex that initiates the formation of autophagosomes [36,37]. Nitsch et al. [38] have recently shown that autophagy plays important roles in physiological adaptation in submerged cultures under conditions of carbon depletion by organelle turnover and protection against cell death. Our data suggest that the slow growth rate on wheat straw may lead to a carbon uptake rate slow enough to already signal starvation. However, autophagy has also been shown to be necessary for fungal morphogenesis, particularly when attacking other organisms [39,40], and we can therefore also not rule out that it is crucial for the growth of *T. reesei* on an insoluble substrate.

The upregulation of genes associated with DNA synthesis and repair appears to indicate the operation of mechanisms leading to damage of DNA during growth on cellulose. While the occurrence of such mechanisms has not been reported so far, we detected a wheat-straw-specific 2.7-fold upregulation of the *T. reesei* orthologue of the NADPH oxidase NoxA (NOX1; Trire2:79498; see Additional file 2: Table S2). This enzyme generates reactive oxygen species in a regulated manner and is involved in several aspects of fungal biology, including development and plant pathogenesis [41]. We consider it possible that the upregulation of enzymes for DNA repair is a response to the formation of reactive oxygen species by NOX1, although the reason for its upregulation is not clear. Brun et al. [42] reported that *Podospora anserina* hyphae form specialized structures for cellulose degradation and their formation is differentially regulated by NOX1 and NOX2. Interestingly, Montero-Barrientos et al. [43] reported that an overexpression of *nox1* in *T. harzianum* leads to the increased formation of protease, cellulase, and chitinase activities during mycoparasitic interaction with a fungal host. Nitsche at al. (2013) reported that in *A. niger* autophagy influences the sensitivity towards oxidative stress. We consider it likely that the detected enhanced autophagic activity on wheat straw (*vide supra*) also causes the increased expression of genes related to DNA damage repair and oxidative stress.

Finally, a very intriguing finding from this study was the massive upregulation of genes involved in iron homeostasis. This elevated expression of iron-uptake systems on wheat straw can be explained by a lower bioavailable concentration of iron during growth on wheat straw (as shown in this study), which is probably due to the ability of cellulose for sequestering Fe^3+^[44]. In nature, this shortage can be reinforced by the formation of Fe^3+^-oxalate chelates with the oxalic acid secreted by basidiomycetes [45]. It is also possible that this induction of iron assimilating enzymes has the additional benefit of triggering the synthesis of the large number of flavin containing oxidases and monooxygenases that are upregulated during growth on wheat straw and result in an increased demand for iron. As already hypothesized [19], this increased expression of oxidative enzymes could indicate the operation of Fenton chemistry during cellulose degradation by *T. reesei*. The increased expression of a glucose oxidase (Trire2:22915) and a gluconate kinase (Trire2:71072), as observed in this study, suggests partial degradation of the glucose from cellulose, which could be involved in the provision of hydrogen peroxide for this process.

## Conclusions

Our study shows that growth on wheat straw leads in part to an enhanced expression of cellulase and hemicellulase genes by *T. reesei*, but also to the selective induction of a set of enzymes, notable the majority of the xylanolytic enzymes. This implies that the expression of genes associated with lignocelluloses degradation by *T. reesei* is subject to as yet unknown regulator proteins which are supposed to cooperate with XYR1. A detailed analysis of transcriptomic changes of genes involved in cellular metabolism and its regulation further identified autophagy, phospholipid biosynthesis, iron homeostasis and DNA repair as processes related to degradation of wheat straw, whose roles warrant further investigations. In addition, manipulation of these genes may be a new tool for strain improvement in *T. reesei*.

## Materials and methods

### Strains and cultivations

*T. reesei* QM9414 (ATCC 26921), a moderately cellulase producing mutant, and a *∆xyr1* mutant prepared from it [13] was used throughout this work and kept on potato dextrose agar (Sigma, St. Louis, MO).

Cultures were grown in 250 ml of Mandels Andreotti (MA) medium (per liter: 1.4 g (NH_4_)_2_SO_4_, 2.0 g KH_2_PO_4_, 0.3 g MgSO_4_*7H_2_O, 0.3 g CaCl_2_*2H_2_O, 0.3 g urea, 1 g peptone (casein), 5 mg FeSO_4_*7H_2_O, 1.6 mg MnSO_4_*H_2_O, 1.4 mg ZnSO_4_*7H_2_O and 2 mg CoCl_2_*2H_2_O) with 10 g/l glucose monohydrate, lactose monohydrate or pretreated wheat straw (dry basis) as the sole carbon source and inoculated with 10^6^ ml^-1^ conidiospores. Pretreated wheat straw was kindly provided by Clariant Produkte Deutschland GmbH. In brief, the substrate was mechanically ground, and subjected to slightly acidic, thermochemical pretreatment.

Furthermore, 0.5 g l^-1^ of tween 80 were added in the case of lactose cultures and the pH of wheat straw media was adjusted to 4.8 with 1 M KOH. All cultivations were performed in a rotary shaker at 28°C and 250 rpm. Biomass samples for total RNA extraction or measurement of biomass were withdrawn at appropriate time points. Cultures for the qPCR analyses were pregrown for 24 h in glycerol containing (10 g l^-1^) MA medium and equal portions of the harvested and washed mycelium were aseptically replaced into MA medium, again containing either 10 g l^-1^ glucose monohydrate, lactose monohydrate or pretreated wheat straw (dry basis) as the sole carbon source, but this time devoid of urea and peptone.

### Transcriptome analysis

Mycelia were harvested from cultures growing on wheat straw, lactose, and glucose, for 50 (wheat straw) and 28 hrs (lactose and glucose), respectively. Total RNAs from glucose and lactose cultures were extracted using TRIzol® reagent (Invitrogen Life Technologies, Carlsbad, CA, USA), according to the manufacturer's instructions, and then purified using the RNeasy MinElute Kit (Qiagen, Hilden, Germany). For isolation and purification of total RNA from wheat straw cultures, the RNeasy Plant Mini Kit and the RNeasy MinElute Kit (both Qiagen, Hilden, Germany) respectively were used according to the manufacturer’s instructions. cDNA synthesis, labelling and hybridization was performed by Roche NimbleGen (Roche-NimbleGen, Inc., Madison, WI, USA) with a high density oligonucleotide microarray using 60-mer probes representing the 9.129 genes of *T. reesei*. Microarray scanning, data acquisition and identification of probe sets showing a significant difference (p = 0.05) in expression level between the different conditions were performed essentially as described previously [13,46]. Gene accession numbers were annotated according to version 2 of the *T. reesei* genome database (http://genome.jgi-psf.org/Trire2/Trire2.home.html), and ambiguous cases annotated manually. The Euclidean distance metric method, as implemented in DNASTAR v5.1.2. build 3 (DNAstar Inc., Madison, WI), was used for Hierarchical Clustering.

Genes were then classified according to their major annotation in the GO (Gene Ontology), KOG (EuKaryotic Orthologous Groups) classification available at the *T. reesei* genome database v2.0 (http://genome.jgipsf.org/Trire2/Trire2.home.html), and the MIPS Functional Catalogue (http://mips.helmholtz-muenchen.de/proj/funcatDB; [8]). To determine whether there were differences in the functional categories in each cluster, the distribution within each cluster was compared to the total distribution of all the annotated genes using independent chi-square tests.

The microarray data and the related protocols are available at the GEO web site (http://www.ncbi.nlm.nih.gov/geo/) under accession number GSE46155.

### Promoter sequence analysis

All analyses were performed with the RSAT software suite [47]. Promoter sequences from −1000 to −1 were obtained from the *T. reesei* genome database (http://genome.jgi-psf.org/Trire2/Trire2.home.html). Motifs were searched using the "DNA Pattern Matching" algorithm, with the "prevent overlapping matches" parameter checked. For a given set of genes (any cluster, or whole genome), the total number of motifs found was collected and an average number of sites per gene was calculated.

### qPCR

DNase treated (DNase I, RNase free; Fermentas) total RNA (5 μg) was reversely transcribed with the RevertAid™ First Strand cDNA Kit (Fermentas) according to the manufacturer’s protocol with a combination (1:1) of the provided oligo-dT and random hexamer primers. All assays were carried out in 96-well plates which were covered with optical tape, as described [13,46]. Primers, amplification efficiency and R-square values are given in Additional file 8: Table S8. Determination of the PCR efficiency was performed using triplicate reactions from a dilution series of cDNA, and the amplification efficiency then calculated from the given slopes in the realplex v2.2 software. Expression ratios were calculated using REST© Software [48]. All samples were analyzed in two independent experiments with three replicates in each run. The unequal varaiance *t-*test was performed as previously described [49].

### Measurement of biomass dry weight

Biomass formation of lactose and glucose cultures was determined gravimetrically, as previously reported [16]. Biomass concentrations of wheat straw cultures were indirectly measured by the amount of intracellular protein essentially as in [50]. In brief, a 1 ml sample of the culture broth was withdrawn and the solids collected by centrifugation. One mL 1 M NaOH was added and the mixture was incubated for two hours and frequently vortexed. This suspension was then clarified by centrifugation and the protein concentration of the supernatant was determined with the BioRad protein assay reagent (BioRad, Hercules, USA) against a BSA standard. The protein content was furthermore corrected by a set of substrate controls where no inoculum was added to the wheat straw medium. The biomass dry weight was then calculated assuming an average content of 0.35 g intracellular protein per g of dry cell mass. Three independent cultivations were performed for each carbon source and the mean of the three experiments is reported.

### Measurement of iron

The concentration of iron in the soluble supernatant of the cultures was measured by inductively coupled plasma mass spectrometry (ICP-MS). To overcome possible matrix effects during sample introduction and ICP-MS analysis, the samples were diluted 1:100 with HNO_3_ (1% v/v) prior to measurement. Indium (1.0 μg L^-1^) was been added as an internal standard to all samples. The measurements were performed using an iCAP Q ICP-MS System from Thermo Fisher Scientific (Bremen, Germany) equipped with a standard quartz tube torch and nickel sample and skimmer cones. For sample introduction a set consisting of a concentric nebulizer and a Peltier cooled cyclonic spray chamber has been used. Transportation of sample solutions was performed by the peristaltic pump of the iCAP Q coupled to an ESI SC2 DX auto sampler (ESI, USA). For separation of spectral interferences caused from polyatomic ions produced in the argon plasma by matrix constituents all ICP-MS measurements were performed in the collision mode using He with 7% H_2_ as collision gas at a flow rate of 5 mL min^-1^ and a KED value of 3 eV. Plasma power was maintained at 1550 W, cooling gas and auxiliary gas flow set at 14 L.min^-1^ and 0.8 L.min^-1^, respectively. Make up gas flow, nebulizer flow rate and sample uptake rate were adjusted at 0.7, 0.99 and 0.4 L.min^-1^, respectively. For analysis the elemental isotopes (*m*/*z* ratios) ^56^Fe, ^57^Fe, ^58^Ni, ^59^Co and ^58^Ni and ^115^In (as internal standard) were monitored. Quantification of derived signals was based on an external calibration function determined with aqueous standard solutions using Indium as internal standard. The ICP-MS operation and data acquisition (by peak hopping) was accomplished by using Qtegra software, using a dwell time of 10 ms and 20 sweeps per reading, and 4 replicates per sample.

## Abbreviations

CAZome: Carbohydrate active enzyme proteome; CAZys: Carbohydrate active enzymes; ESI: Electron spray ionization; GEO: Gene expression omnibus; GH: Glycosyl hydrolase; GO: Gene ontology; ICP-MS: Inductively coupled plasma mass spectrometry; KED: Kinetic energy discrimination; KOG: EuKaryotic Orthologous Groups; MIPS: MUNICH Information Center for Protein Sequences; qPCR: Quantitative polymerase chain reaction; REST: Relative expression software tool.

## Competing interests

The authors declare that they have no competing interests.

## Authors’ contributions

BS and CPK initiated, designed and coordinated the study and reviewed the manuscript. RB planned and carried out experiments and measurements and interpreted experimental data. LF carried out experiments and measurements and analysed experimental data. AL performed the iron analyses. CG analyzed and discussed data. CPK drafted the paper. All authors have read and approved the final manuscript.

## Supplementary Material

Additional file 1: Table S1 Genes constitutively expressed in *T. reesei* on glucose (Glc), lactose (Lac) and wheat straw (WS). Click here for file

Additional file 2: Table S2 All genes that are at least 2-fold differentially regulated on lactose or wheat straw vs glucose. Click here for file

Additional file 3: Table S3 Concentration of iron in the culture supernatant on lactose and wheat straw. Click here for file

Additional file 4: Table S4 Genes present in the clusters shown in Figure 4. Click here for file

Additional file 5: Table S5 Unequal variance *t*-test of qPCR results shown in Figure 5. Click here for file

Additional file 6: Table S6 Presence of the XYR1 binding consensus motif in the first 1000 bp upstream of the start codon in the genes upregulated in the *Δxyr1* mutant strain. Click here for file

Additional file 7: Table S7 Autophagy genes in *T. reesei.* Click here for file

Additional file 8: Table S8 Nucleotide sequences and Reaction efficiencies of RT-qPCR oligos used in this study. Click here for file
