# Supplementary material for: Comparative analysis of the Trichoderma reesei transcriptome during growth on the cellulase inducing substrates wheat straw and lactose
Source: Biotechnol Biofuels. 2013 Sep 9;6:127. doi: 10.1186/1754-6834-6-127 (PMC3847502; doi:10.1186/1754-6834-6-127)
Supplement: Additional file 1: Table S1 — Genes constitutively expressed in T. reesei on glucose (Glc), lactose (Lac) and wheat straw (WS). [file 1754-6834-6-127-S1.docx]

| **Supplementary Table S1:** Genes constitutively expressed in T. reesei on glucose (Glc), lactose (Lac) and wheat straw (WS) | | | | |
| --- | --- | --- | --- | --- |
|  |  |  |  |  |
| **SEQ_ID** | **P value** | **Glc vs Lac** | **Glc vs WS** |  |
| 49213 | 0.038 | 1.093 up | 1.236 up | 14-3-3 protein |
| 78925 | 0.00128 | 1.402 down | 1.346 down | 20S proteasome beta-type subunit Pre4 |
| 55589 | 0.012 | 1.053 down | 1.470 up | 3' exoribonuclease involved in RNA processing during translation. |
| 53350 | 0.0427 | 1.080 up | 1.157 down | 39S ribosomal protein L53/MRP-L53, putative |
| 74453 | 0.000211 | 1.052 up | 1.313 up | 3-hydroxyacyl-CoA dehydrogenase |
| 2912 | 0.000857 | 1.154 up | 1.312 down | 3-oxo-5-alpha-steroid 4-dehydrogenase |
| 79106 | 0.0339 | 1.289 down | 1.344 down | 40s ribosomal protein L44e by homology to the corresponding protein of several yeasts and fungi. Homologue of yeast RPL42a/b. |
| 119626 | 0.0485 | 1.085 down | 1.473 down | 40s ribosomal protein S22 (S8 family) by homology to the corresponding protein of N. crassa. Homologue of yeast RPS22a/b. |
| 68909 | 0.00906 | 1.277 down | 1.330 down | 40s ribosomal protein S26E (CRP5) (13.6 kDa ribosomal protein). |
| 75079 | 0.000232 | 1.431 down | 1.440 up | 5-phospho-ribosyl-1(alpha)-pyrophosphate synthetase |
| 123850 | 0.0262 | 1.472 down | 1.350 down | 60s acid ribosomal protein P1 based on homology to corresponding proteins in fungi and yeasts. |
| 104491 | 0.0205 | 1.282 down | 1.429 down | 60S ribosomal protein L35 (L29 family) by homology with the corresponding protein in other fungi. |
| 80515 | 0.00979 | 1.106 down | 1.425 up | 60S ribosome biogenesis protein Mak11, putative |
| 2091 | 0.0116 | 1.242 down | 1.051 up | 6-phosphofructokinase |
| 47175 | 0.0067 | 1.364 up | 1.163 up | Aa_transTransmembrane amino acid transporter protein |
| 110586 | 0.00364 | 1.291 up | 1.411 down | AAA ATPase |
| 44097 | 0.00329 | 1.496 down | 1.040 down | AAA family ATPase Pontin, putative |
| 50542 | 0.00648 | 1.118 up | 1.294 down | ABC transporter |
| 76324 | 0.000747 | 1.409 down | 1.420 up | acetoacetyl-CoA synthase |
| 74123 | 0.0278 | 1.283 down | 1.424 up | acetolactate synthase |
| 121191 | 0.00199 | 1.121 down | 1.430 up | Acetyltransf_Acetyltransferase (GNAT) family |
| 68973 | 0.00167 | 1.364 down | 1.339 up | acid phosphatase with metalloesterase domain |
| 22632 | 0.0379 | 1.270 down | 1.352 up | aconitate hydratase |
| 44504 | 0.023 | 1.083 down | 1.359 down | actin |
| 68926 | 0.0332 | 1.483 down | 1.204 up | actin polymerization protein Bzz1, putative |
| 57891 | 0.0434 | 1.162 down | 1.383 up | actinin actin binding and calcium-binding EF-hand domains |
| 111468 | 0.00761 | 1.398 down | 1.431 up | actin-like protein; high identity to predicted Fusarium graminearum FG04485.1 |
| 75175 | 0.00563 | 1.010 up | 1.332 up | acyl-CoA dehydrogenase |
| 64066 | 0.00151 | 1.301 up | 1.217 up | acyltransferase 3 |
| 120539 | 0.0281 | 1.236 down | 1.043 down | Adenylate kinase |
| 45604 | 0.00688 | 1.247 down | 1.139 up | ADP-ribosylation factor Arf1 |
| 23263 | 0.0293 | 1.436 up | 1.371 up | ADP-ribosylation factor Arf6 |
| 4537 | 0.0114 | 1.366 down | 1.102 down | alanyl-tRNA synthetase, class IIc. |
| 61910 | 0.0441 | 1.253 up | 1.012 down | Aldehyde dehydrogenase |
| 123795 | 0.033 | 1.407 down | 1.243 down | allantoinase |
| 119767 | 0.00285 | 1.277 down | 1.198 down | alpha-tubulin cofactor B. |
| 81125 | 0.000661 | 1.291 up | 1.056 up | Amino acid permease |
| 2790 | 0.000985 | 1.332 down | 1.226 up | aminoacyl-tRNA synthase. |
| 69816 | 0.00135 | 1.108 down | 1.265 up | aminoglycoside 3-N-acetyltransferase |
| 82452 | 0.00264 | 1.029 up | 1.332 up | Aminopeptidase P |
| 45503 | 0.0103 | 1.166 up | 1.067 up | ankyrin containing protein |
| 33723 | 0.0162 | 1.194 down | 1.176 down | ankyrin repeat-containing protein, putative |
| 58076 | 0.0132 | 1.115 down | 1.407 up | apurinic/apyrimidinic endonuclease-like protein |
| 43877 | 0.0153 | 1.183 down | 1.493 up | Arfs GEF Sec7 |
| 44419 | 0.00758 | 1.339 down | 1.083 down | ARG5,6 |
| 107383 | 0.0411 | 1.051 down | 1.180 down | arginine N-methyltransferase skb1 |
| 78552 | 0.00149 | 1.036 down | 1.238 up | Aromatic L-amino acid decarbounknown proteinlase |
| 105202 | 0.0463 | 1.181 down | 1.028 down | Arp2/3 complex, 34kDa subunit p34-Arc |
| 122505 | 0.0378 | 1.348 down | 1.476 down | aryl-alcohol dehydrogenase |
| 54694 | 0.00109 | 1.120 down | 1.104 down | aryl-alcohol dehydrogenases |
| 77579 | 0.00253 | 1.084 down | 1.385 up | aspartyl protease |
| 58669 | 0.00321 | 1.261 down | 1.474 up | aspartyl protease |
| 123801 | 0.0474 | 1.192 down | 1.175 down | aspartyl-tRNA synthetase, class IIb. |
| 62040 | 0.0051 | 1.196 down | 1.476 up | ATPase Sec18, required for ER to Golgi transport etc |
| 74818 | 0.0104 | 1.419 down | 1.285 down | ATP-dependent RNA helicase DBP10 |
| 51455 | 0.00882 | 1.286 down | 1.134 down | ATP-dependent RNA helicase DBP5 |
| 58627 | 0.0132 | 1.353 up | 1.192 up | ATP-dependent RNA helicase dbp9 |
| 46619 | 0.044 | 1.338 down | 1.082 down | ATP-dependent RNA helicase eIF4A |
| 75551 | 0.00302 | 1.180 down | 1.489 up | ATP-NAD kinase |
| 67420 | 0.0419 | 1.012 up | 1.359 up | Autophagic death protein Aut7/IDI-7 |
| 123820 | 0.00321 | 1.017 up | 1.379 up | BCAT_beta_family |
| 75294 | 0.0299 | 1.132 up | 1.385 down | BCAT_beta_family |
| 121588 | 0.000514 | 1.393 down | 1.315 up | beta-adaptin, apl2, large subunit of the adaptor protein (AP-1) complex of clathrin-coated vesicles |
| 22774 | 0.0087 | 1.180 up | 1.253 up | bHLH transcriptional regulator |
| 48707 | 0.00359 | 1.393 down | 1.115 up | Bifunctional trehalose-6-phosphate synthase/trehalose-6-phosphate phosphatase |
| 77714 | 0.000479 | 1.317 up | 1.345 down | biotin-protein ligase |
| 120897 | 0.00303 | 1.305 up | 1.479 up | BRCT domain protein Rad4/Cut5 of Schizosaccharomyces pombe |
| 57840 | 0.04 | 1.102 down | 1.109 up | bZIP transcription factor |
| 103372 | 0.00942 | 1.203 down | 1.276 up | BZIP transcriptional regulator |
| 22532 | 0.0111 | 1.300 up | 1.001 up | BZIP transcriptional regulator |
| 74576 | 0.0163 | 1.301 up | 1.163 down | BZIP transcriptional regulator |
| 112538 | 0.0072 | 1.191 down | 1.246 up | C2H2 transcription factor |
| 58628 | 0.0157 | 1.023 down | 1.450 up | C2H2 transcription factor (Con7) |
| 120363 | 0.000642 | 1.289 down | 1.179 down | C2H2 transcriptional regulator |
| 58897 | 0.00578 | 1.395 up | 1.113 up | C2H2 transcriptional regulator |
| 122541 | 0.00962 | 1.323 up | 1.317 up | C2H2 transcriptional regulator |
| 119826 | 0.015 | 1.296 up | 1.276 down | C2H2 transcriptional regulator |
| 5675 | 0.0228 | 1.194 down | 1.432 up | C2H2 transcriptional regulator |
| 110658 | 0.00306 | 1.104 down | 1.378 up | C2HC transcription factor |
| 52130 | 0.0157 | 1.410 down | 1.211 up | Calcineurin B subunit, Protein phosphatase 2B subunit |
| 59944 | 0.00913 | 1.217 down | 1.013 down | calcineurin catalytic subunit |
| 75347 | 0.0176 | 1.264 down | 1.417 up | Calcium ion pump, ATPase |
| 56744 | 0.00584 | 1.068 up | 1.047 down | Calcium transporter |
| 22381 | 0.000901 | 1.174 down | 1.336 up | calcium/calmodulin-dependent protein kinase |
| 73678 | 0.00629 | 1.197 down | 1.144 up | calnexin, high identity with A. niger clxA |
| 57399 | 0.00278 | 1.019 down | 1.401 up | cAMP dependent protein kinase, protein kinase A, catalytic subunit |
| 119614 | 0.00377 | 1.338 down | 1.244 up | CAMP dependent serine/threonine protein kinase related to S.Cerevisiae Sch9 |
| 122226 | 0.0483 | 1.088 down | 1.229 down | Carbamoyl-phosphate synthase, small chain |
| 123714 | 0.00129 | 1.012 up | 1.233 up | carbonic anhydrase |
| 4843 | 0.00244 | 1.012 down | 1.395 up | Carbon-nitrogen hydrolase |
| 21746 | 0.00583 | 1.286 up | 1.359 down | carboxymuconolactone decarboxylase, putative |
| 56318 | 0.00231 | 1.045 down | 1.142 up | Cargo transport protein Emp24 (p24 protein family) |
| 119578 | 0.0246 | 1.467 up | 1.156 up | Cargo transport protein Erv29 |
| 112190 | 0.0175 | 1.256 down | 1.467 up | CDC31, putative spindle pole body component |
| 70703 | 0.00137 | 1.322 down | 1.124 down | CDP-alcohol phosphatidyltransferase |
| 55627 | 0.00727 | 1.265 down | 1.106 up | CDP-alcohol phosphatidyltransferase |
| 44214 | 0.0225 | 1.448 down | 1.375 up | CE5 acetyl xylan esterase AXE2 |
| 105351 | 0.00272 | 1.460 down | 1.211 up | cell cycle control protein cwf8 |
| 22314 | 0.000291 | 1.134 up | 1.314 up | cell division control protein |
| 122199 | 0.0386 | 1.204 down | 1.361 up | cell division control protein 3 |
| 55912 | 0.0421 | 1.091 down | 1.311 up | Cell division/GTP binding protein |
| 77084 | 0.0395 | 1.080 down | 1.056 up | cell wall biogenesis protein phosphatase Ssd1, putative |
| 122047 | 0.00277 | 1.264 up | 1.265 up | cell wall protein. Ser/Thr-rich. |
| 58549 | 0.0108 | 1.390 down | 1.192 up | cellular morphogenesis regulator DopA, putative |
| 54366 | 0.00442 | 1.452 down | 1.301 down | ceramidase, nonlysosomal |
| 121061 | 0.0106 | 1.424 up | 1.013 up | checkpoint kinase 2-like protein |
| 58188 | 0.00913 | 1.040 down | 1.108 up | Chitin synthase |
| 51492 | 0.0402 | 1.373 up | 1.458 up | Chitin synthase |
| 120219 | 0.0167 | 1.089 down | 1.141 down | Chorismate_mutChorismate mutase aligned |
| 77969 | 0.000626 | 1.010 down | 1.376 up | chromate transporter |
| 21557 | 0.0134 | 1.292 up | 1.422 up | chromatin remodelling factors, contains a SWIRM domain and a DNA-binding Myb-domain |
| 5504 | 0.00435 | 1.033 up | 1.486 up | class I glutamine amidotransferase, putative |
| 123155 | 0.00501 | 1.218 down | 1.249 up | Clathrin lightChain,Clc1, vesicleCoat protein |
| 106150 | 0.0247 | 1.270 down | 1.372 down | Coenzyme Q (ubiquinone) biosynthesis protein Coq4, putative |
| 73967 | 0.0345 | 1.189 down | 1.175 up | cofilin |
| 74177 | 0.0127 | 1.401 down | 1.302 down | coiled-coil domain-containing protein, putative |
| 76930 | 0.0101 | 1.039 up | 1.078 up | complex I intermediate-associated protein 30 |
| 54042 | 0.00416 | 1.189 down | 1.379 up | Component of oligomeric golgi complex Cog4/Sec38 |
| 69068 | 0.0194 | 1.457 down | 1.042 up | contains: UBA/TS-N domain and DNA-binding domain |
| 67272 | 0.0092 | 1.211 up | 1.481 up | CRO1, required for syncytial to cellular transision, involved in sexual development |
| 77989 | 0.0059 | 1.258 up | 1.137 down | Csh7, an ER membrane chaperone protein |
| 123457 | 0.00676 | 1.318 down | 1.446 up | CTP synthase |
| 120067 | 0.00911 | 1.449 down | 1.345 up | Cue1 domain protein involved in ER-associated protein degradation. Distantly related to mammalian AMFR2 |
| 82651 | 0.0416 | 1.305 up | 1.451 up | Cullin |
| 54195 | 0.00286 | 1.161 down | 1.292 up | Cyclin-K , putative |
| 82547 | 0.0053 | 1.394 down | 1.087 up | cystathionine beta-lyase |
| 60676 | 0.000183 | 1.436 down | 1.235 up | cysteine protease, mammalian caspases |
| 4901 | 0.0102 | 1.121 up | 1.395 down | cytochrome c oxidase assembly protein COX16, putative |
| 121275 | 0.0214 | 1.331 down | 1.115 down | cytochrome c oxidase subunit VIa |
| 121977 | 0.0306 | 1.394 down | 1.093 down | CytoplasmicCyclophilin |
| 62198 | 0.00255 | 1.402 down | 1.464 down | Cytosine-purine permease |
| 82039 | 0.00211 | 1.295 up | 1.176 up | D-aminoacylase, putative |
| 61550 | 0.000712 | 1.095 up | 1.001 up | DEAD/DEAH box helicase |
| 36941 | 0.0482 | 1.040 up | 1.065 up | DEAD/DEAH box helicase |
| 74267 | 0.0244 | 1.273 down | 1.063 down | Delta subunit, ret2, of the coatomer complex (COPI) |
| 121733 | 0.00768 | 1.322 down | 1.405 up | delta(14)-sterol reductase |
| 123805 | 0.00447 | 1.330 down | 1.206 up | DHBP_synthase,-dihydroxy--butanone -phosphate synthase |
| 65882 | 0.00279 | 1.331 up | 1.159 down | dihydrodipicolinate synthase , putative |
| 79545 | 0.00216 | 1.495 down | 1.002 down | Dioxygenases related to -nitropropane dioxygenase |
| 107669 | 0.00297 | 1.123 up | 1.326 down | Dipeptidyl aminopeptidase |
| 70994 | 0.0059 | 1.121 up | 1.475 down | dipeptidyl peptidase 5 |
| 59723 | 0.0284 | 1.293 down | 1.366 up | dipeptidyl peptidase 5 |
| 60752 | 0.04 | 1.362 down | 1.208 up | dipeptidyl peptidase 5 |
| 81043 | 0.00162 | 1.401 up | 1.303 up | DNA directed RNA polymerase II 15 kDa subunit, putative |
| 58658 | 0.00215 | 1.482 up | 1.470 up | DNA helicase, putative |
| 2095 | 0.0262 | 1.063 up | 1.482 up | DNA polymerase delta, catalytic subunit |
| 1702 | 0.00783 | 1.469 down | 1.074 up | DNA polymerase V (phi) |
| 75069 | 0.000808 | 1.019 up | 1.466 up | DNA polymeraseDelta, subunit 3 |
| 61551 | 0.00364 | 1.111 down | 1.350 up | DNA polymeraseDelta, subunit 4 |
| 53721 | 0.00214 | 1.271 up | 1.262 down | DNA replication complex GINS protein SLD5, putative |
| 63454 | 0.035 | 1.119 down | 1.297 up | DNA replication licensing factor mcm2 |
| 3212 | 0.0186 | 1.226 down | 1.496 up | DNA replication licensing factor mcm7 |
| 79947 | 0.0188 | 1.208 down | 1.174 up | DNA-dependent RNA polymerase |
| 105191 | 0.00108 | 1.133 up | 1.349 down | DNA-directed RNA polymerase, 30-40 kDa subunit |
| 22064 | 0.00115 | 1.408 up | 1.086 down | DNAJ heat shock family protein |
| 74168 | 0.00166 | 1.157 down | 1.013 down | Dolichyl-phosphate mannosyltransferase |
| 22005 | 0.034 | 1.246 up | 1.422 up | Dolichyl-P-mannose:protein O-mannosyl transferase |
| 75421 | 0.0487 | 1.268 up | 1.321 up | Dolichyl-P-mannose:protein O-mannosyl transferase |
| 62769 | 0.00282 | 1.366 down | 1.267 down | DPH2, required for diphthamide synthesis. |
| 43599 | 0.00839 | 1.046 down | 1.246 down | Dynamin-like GTPase Vps1 |
| 57508 | 0.00873 | 1.341 down | 1.369 down | Dynamin-related GTPase |
| 4915 | 0.00216 | 1.031 up | 1.129 up | dynein light intermediate chain (DLIC). |
| 102579 | 0.00804 | 1.436 down | 1.387 up | E3 ubiquitin-protein ligase BRE1 (EC 6.3.2.-) [BRE1] |
| 4707 | 0.00418 | 1.468 down | 1.267 up | E3 ubiquitin-protein ligase, IQ and HECT domain |
| 120235 | 0.00799 | 1.373 down | 1.353 down | Elongation factor 2 |
| 55422 | 0.00393 | 1.369 down | 1.208 up | elongation factor 2 kinase, putative |
| 121292 | 0.0193 | 1.022 up | 1.221 up | Emp24/gp25L/p24 family protein Erp3 |
| 71399 | 0.0167 | 1.046 up | 1.091 down | endo-1,3-β-glucanase |
| 21588 | 0.0413 | 1.337 down | 1.005 down | endomembrane protein 70 |
| 123577 | 0.00483 | 1.150 up | 1.214 down | Endoplasmatic reticulum oxidising protein Ero1 |
| 120765 | 0.0119 | 1.288 up | 1.498 up | enoyl-CoA hydratase/isomerase |
| 21982 | 0.00299 | 1.265 down | 1.165 down | Epl1/Sm1 |
| 111449 | 0.00143 | 1.281 down | 1.359 up | Epl1-like protein |
| 79104 | 0.0144 | 1.301 down | 1.032 down | Epsilon subunit of the coatomer complex, sec28 |
| 52550 | 0.0137 | 1.404 down | 1.236 up | Epsin-like protein,Ent2, involved inEndocytosis and actin patch assembly |
| 81263 | 0.0113 | 1.164 up | 1.396 up | ER lumen protein retaining receptor Erd2 |
| 79295 | 0.000423 | 1.155 down | 1.375 up | ER membrane proteins involved in ER exit of secreted proteins. |
| 122071 | 0.0194 | 1.171 down | 1.142 down | ER-derived vesicles protein Erv14 |
| 23271 | 0.0247 | 1.217 down | 1.322 up | ER-derived vesicles protein Erv41 |
| 58689 | 0.0144 | 1.055 down | 1.245 up | ERG5 C-22 sterol desaturase, a cytochrome P450 enzyme |
| 68961 | 0.0142 | 1.107 down | 1.049 down | esterase |
| 71326 | 0.00858 | 1.423 down | 1.458 up | Esterase;too long in N-term; 214 was better |
| 66296 | 0.0224 | 1.126 down | 1.406 up | eukaryotic and archaeal DNA primase |
| 78046 | 0.0212 | 1.134 down | 1.469 up | eukaryotic translation initiation factor 3 |
| 121901 | 0.00176 | 1.312 down | 1.164 up | eukaryotic translation initiation factor 3 subunit 8, N-terminal. |
| 122841 | 0.00149 | 1.186 down | 1.223 up | Exocyst component Sec3 |
| 60769 | 0.0119 | 1.122 down | 1.390 up | Exonuclease |
| 112211 | 0.0143 | 1.139 up | 1.431 up | exonuclease, putative |
| 124317 | 0.0187 | 1.360 up | 1.435 up | exoribonuclease Dhp1 |
| 2087 | 0.00448 | 1.088 down | 1.230 down | exosome complex exonuclease RRP43 |
| 23115 | 0.0122 | 1.335 down | 1.229 up | expansin |
| 57600 | 0.0208 | 1.158 up | 1.292 up | FACT complex protein (Facilitates chromatin transcription complex subunit SPT16) |
| 110983 | 0.00116 | 1.117 down | 1.047 down | FAD dependent oxidoreductase |
| 76617 | 0.0177 | 1.287 down | 1.318 up | FAD-binding dehydrogenase |
| 65055 | 0.00241 | 1.160 up | 1.368 up | fatty acid desaturase |
| 119931 | 0.0044 | 1.079 down | 1.250 up | fatty acid elongase |
| 1925 | 0.00169 | 1.325 down | 1.180 down | fatty acid hydroxylase, Cytb5, SUR2-type hydroxylase/desaturase, catalytic region |
| 78641 | 0.0192 | 1.354 down | 1.299 up | F-box and WD domain-containing protein |
| 4592 | 0.000918 | 1.316 down | 1.348 up | F-box domain-containing protein |
| 61116 | 0.0127 | 1.480 up | 1.378 up | ferric reductase |
| 124195 | 0.000769 | 1.205 up | 1.081 up | FeS cluster assembly scaffold IscU |
| 78496 | 0.0146 | 1.101 down | 1.428 down | Flavin-containing monooxygenase Fmo1 like |
| 120172 | 0.0161 | 1.356 up | 1.352 up | flavodoxin and radical SAM domain protein |
| 56845 | 0.0362 | 1.439 up | 1.054 down | flavoprotein monooxygenase |
| 69210 | 0.00589 | 1.499 down | 1.246 up | FoNIIA |
| 23200 | 0.0135 | 1.159 up | 1.345 down | fructose-bisphosphate aldolase |
| 57421 | 0.00852 | 1.069 down | 1.421 up | G2/mitotic-specific cyclin-B [Neurospora crassa OR74A] |
| 123422 | 0.0196 | 1.073 up | 1.301 up | GAP Gyp1 |
| 3671 | 0.00117 | 1.129 up | 1.463 down | Gar1 protein with RNA-binding region |
| 4231 | 0.0401 | 1.413 down | 1.011 up | GATA type transcriptional regulator |
| 66433 | 0.00434 | 1.039 up | 1.007 down | GCN5 N-acetyltransferase |
| 4484 | 0.00109 | 1.321 up | 1.075 down | GCN5-N-acetyltransferase activity |
| 104222 | 0.0373 | 1.137 down | 1.095 down | GCN5-related acetyltransferase |
| 82246 | 0.00237 | 1.425 down | 1.390 up | GCPR, mPR-type |
| 56426 | 0.0164 | 1.304 down | 1.357 up | GCPR, mPR-type |
| 82145 | 0.000507 | 1.130 up | 1.224 up | GDP-fucose transporter (putative) |
| 77284 | 0.00422 | 1.273 down | 1.024 up | GH12 endo-ß-1,4-glucanase |
| 121355 | 0.0017 | 1.409 up | 1.470 up | GH18 chitinase CHI18-rel2 |
| 62704 | 0.0478 | 1.074 up | 1.271 up | GH18, chitinase CHI18-3 |
| 62645 | 0.00718 | 1.095 up | 1.440 up | GH18, chitinase CHI18-4 |
| 105246 | 0.00454 | 1.484 down | 1.215 up | GH23 exo-ß-1,3-glucanase, distantly related |
| 69736 | 0.0067 | 1.465 up | 1.238 down | GH30 glucan endo 1,6-β-glucanase |
| 111953 | 0.00567 | 1.025 up | 1.064 down | GH47 α-1,2-mannosidase |
| 64906 | 0.00304 | 1.265 down | 1.129 down | GH5 endo-β-1,6-glucanase |
| 22914 | 0.00317 | 1.327 down | 1.287 up | GH72 β-1 3-glucanosyltransferase |
| 67844 | 0.00218 | 1.448 down | 1.385 up | GH76 α-1,6-mannanase |
| 72568 | 0.023 | 1.474 down | 1.299 up | GH79 ß-glucuronidase |
| 73005 | 0.00203 | 1.431 down | 1.308 up | GH79 β-glucuronidase |
| 79602 | 0.0065 | 1.074 down | 1.326 up | GH81 endo-1,3-β-glucanase |
| 120952 | 0.00786 | 1.468 down | 1.168 up | Glutamine Phosphoribosylpyrophosphate amidotransferase PurF |
| 122811 | 0.00651 | 1.317 down | 1.033 up | glutamine synthetase |
| 60814 | 0.00127 | 1.332 down | 1.173 up | glutamyl-tRNA amidotransferase subunit B in other fungi. |
| 4373 | 0.00937 | 1.163 down | 1.384 down | glutaredoxin domain-containing protein |
| 5330 | 0.00129 | 1.296 down | 1.126 up | glutathione S-transferase, putative |
| 104972 | 0.00372 | 1.079 up | 1.371 down | glutathione synthase |
| 64704 | 0.0151 | 1.384 down | 1.491 up | glycerol:H+ symporter (Gup1), putative |
| 44529 | 0.0262 | 1.003 down | 1.361 up | glycogen synthase involved in carbohydrate transport and metabolism |
| 74400 | 0.0247 | 1.123 up | 1.184 up | glycogen synthase kinase 3 |
| 120424 | 0.0287 | 1.158 up | 1.142 down | Glyoxalase I |
| 59778 | 0.0015 | 1.061 up | 1.467 up | GPCR, related to A nidulans GprC |
| 66844 | 0.0452 | 1.492 down | 1.327 up | GPI ethanolamine phosphate transferase, putative |
| 70823 | 0.0384 | 1.133 down | 1.199 up | GPI transamidase component GPI16 |
| 63946 | 0.0151 | 1.315 down | 1.408 up | GPI transamidase subunit PIG-U |
| 123302 | 0.00795 | 1.368 down | 1.435 up | G-protein alpha subunit 1 GNA1 |
| 46469 | 0.0205 | 1.050 down | 1.031 up | G-protein beta subunit |
| 54633 | 0.000901 | 1.093 up | 1.145 up | GT ß-glycosyltransferases |
| 75336 | 0.00999 | 1.219 up | 1.335 up | GT alpha 1,2 mannosyltransferase |
| 69211 | 0.00852 | 1.114 up | 1.033 down | GT α-1,2-mannosyltransferase |
| 46421 | 0.00853 | 1.214 down | 1.361 down | GT α-1,2-mannosyltransferase |
| 66888 | 0.00153 | 1.326 up | 1.415 down | GT α-1,3-mannosyltransferase |
| 81211 | 0.000616 | 1.019 up | 1.351 up | GT α-1,6-mannosyltransferase |
| 58609 | 0.00933 | 1.253 down | 1.308 up | GT α-1,6-mannosyltransferase |
| 65646 | 0.0114 | 1.331 down | 1.107 down | GT α-1,6-mannosyltransferase |
| 77283 | 0.00231 | 1.221 down | 1.140 up | GT2 glycosyltransferase |
| 79396 | 0.00484 | 1.321 up | 1.056 down | GT2 ß-glycosyltransferase, related to hyaluronan synthases |
| 55005 | 0.0175 | 1.035 up | 1.303 up | GT22 ALG9 mannosyltransferase |
| 120923 | 0.000111 | 1.367 down | 1.093 down | GT32 α-glycosyltransferase |
| 75547 | 0.00881 | 1.492 down | 1.068 up | GTP binding nuclear protein Ran, member of the superfamily of RAS small GTPases, related to N. crassa GTP-binding nuclear protein SPI1 |
| 80713 | 0.00957 | 1.325 up | 1.285 up | GTPase acitvating protein for Rho subfamily of RAS smallGTPases, related to S. cerevisiae SAC7p |
| 74701 | 0.00628 | 1.168 down | 1.449 up | GTP-binding protein involved in protein synthesis |
| 81447 | 0.00897 | 1.134 down | 1.430 up | GTP-binding protein YchF |
| 121774 | 0.0235 | 1.267 up | 1.028 up | Guanine nucleotide exchange factor Sec12 |
| 107402 | 0.0115 | 1.396 down | 1.033 up | guanylate kinase |
| 73975 | 0.00851 | 1.056 up | 1.420 up | GYF domain-containing protein |
| 78373 | 0.013 | 1.312 up | 1.446 up | half-sized ABC transporter |
| 51483 | 0.00312 | 1.327 down | 1.410 up | Haloacid dehalogenase-like hydrolase |
| 81930 | 0.0272 | 1.240 up | 1.177 up | HAM1-like protein, probably related to DNA-repair |
| 119593 | 0.0059 | 1.166 down | 1.329 down | Has homology to tubuling binding cofactor A. |
| 21937 | 0.0313 | 1.218 down | 1.086 down | Heat shock protein DnaJ |
| 54129 | 0.0105 | 1.129 down | 1.107 down | Heme peroxidase, unknown in Sordariomycetes |
| 44117 | 0.00471 | 1.141 up | 1.027 up | Heterokaryon incompatibility protein HEC-C |
| 123056 | 0.0183 | 1.295 down | 1.138 down | hex1, encodes the major protein of the Woronin body forming a plug between fungal compartments. |
| 73665 | 0.0286 | 1.387 down | 1.390 down | hexokinase |
| 73173 | 0.00581 | 1.103 up | 1.452 up | HFB1 |
| 53947 | 0.014 | 1.440 up | 1.298 up | HFBs |
| 21468 | 0.0495 | 1.035 down | 1.112 down | HFBs |
| 124052 | 0.0159 | 1.029 down | 1.090 up | histone 2A |
| 4989 | 0.0321 | 1.027 up | 1.411 up | histone acetyltransferase |
| 21542 | 0.00264 | 1.141 down | 1.427 up | histone acetyltransferase ESA1 |
| 79441 | 0.021 | 1.143 down | 1.299 up | histone deacetylase complex subunit |
| 121522 | 0.0306 | 1.136 down | 1.146 down | histone H2A |
| 121516 | 0.0246 | 1.354 down | 1.118 down | histone H2B |
| 53628 | 0.00683 | 1.249 down | 1.443 up | histone H3 methyltransferase complex and RNA cleavage factor II complex, subunit SWD2 |
| 71380 | 0.0137 | 1.350 up | 1.107 up | HMG-CoA reductase. |
| 75646 | 0.00683 | 1.164 up | 1.027 up | HNRNP arginine N-methyltransferase |
| 77154 | 0.0392 | 1.056 up | 1.108 up | homeobox transcriptional regulator |
| 64469 | 0.0425 | 1.313 up | 1.289 down | Hydrolases of alpha/beta hydrolase superfamily |
| 102382 | 0.000875 | 1.010 up | 1.215 down | hydroxyacylglutathione hydrolase |
| 75992 | 0.0312 | 1.239 down | 1.002 down | hydroxyethylthiazole kinase |
| 75589 | 0.00876 | 1.445 down | 1.441 up | hydroxymethylglutaryl CoA synthase |
| 54128 | 0.00196 | 1.391 up | 1.041 down | IlvA Threonine dehydratase |
| 73525 | 0.00245 | 1.028 up | 1.466 up | importin ß KapE |
| 62120 | 0.0268 | 1.068 down | 1.391 up | importin ß KapK |
| 82037 | 0.0491 | 1.247 down | 1.312 up | Inorganic phosphate transporter |
| 53728 | 0.0188 | 1.151 down | 1.301 down | inorganic pyrophosphatase |
| 61382 | 0.00837 | 1.475 down | 1.084 up | Integral membrane protein Sys1 |
| 41761 | 0.0011 | 1.274 up | 1.284 down | iron transporter |
| 53815 | 0.00241 | 1.397 up | 1.462 down | isochorismatase family hydrolase, putative |
| 74983 | 0.034 | 1.214 down | 1.158 down | Isocitrate dehydrogenase, subunit 2, NAD-dependent, mitochondrial |
| 56370 | 0.00863 | 1.137 up | 1.160 up | Isocitrate/isopropylmalate dehydrogenase |
| 107896 | 0.0454 | 1.025 up | 1.469 up | Isoflavone reductase |
| 111832 | 0.00189 | 1.208 down | 1.020 up | Isoflavone reductase superfamily protein |
| 75304 | 0.00398 | 1.493 down | 1.390 up | Isopentenyl-diphosphate delta isomerase 2 |
| 120752 | 0.00203 | 1.069 down | 1.048 up | K+ transporter Trk1 |
| 66486 | 0.000452 | 1.444 down | 1.066 up | kinesin-like motor protein Kar3p required for karyogamy |
| 78783 | 0.00161 | 1.247 up | 1.427 up | kinetochore protein SPC25 |
| 120058 | 0.00262 | 1.413 down | 1.309 up | L-2-hydrounknown proteinglutarate dehydrogenase |
| 4009 | 0.0249 | 1.203 down | 1.124 down | lactate/malate dehydrogenase |
| 59846 | 0.0172 | 1.032 down | 1.099 up | lanosterol synthase |
| 120978 | 0.0216 | 1.380 down | 1.390 down | large ribosomal subunit (protein L34e). |
| 78773 | 0.0162 | 1.231 up | 1.467 up | long-chain-fatty-acid--CoA ligase 2 |
| 53529 | 0.00278 | 1.037 up | 1.065 up | low temperature viability protein |
| 3716 | 0.0155 | 1.206 down | 1.032 down | L-PSP endoribonuclease family protein Brt1 |
| 22076 | 0.00974 | 1.490 down | 1.059 up | lysine decarboxylase |
| 81700 | 0.00453 | 1.076 up | 1.420 up | mannose-6-phosphate isomerase |
| 121539 | 0.0132 | 1.265 down | 1.356 down | MAPK1, mitogen activated protein kinase 1 involved in biocontrol activity in H. virens |
| 21168 | 0.00259 | 1.153 down | 1.311 up | MAPKK, MAP kinase kinase Mkk1 |
| 75872 | 0.00671 | 1.228 up | 1.368 up | MAPKK, MAP kinase kinase, involved in induction of cytokinesis and appressorium formation by host signals |
| 120618 | 0.0263 | 1.054 down | 1.161 up | mediator of RNA polymerase II transcription subunit 14, putative |
| 60243 | 0.0432 | 1.014 up | 1.437 up | medium subunit, apm1, of the adaptor protein complex AP-1 of clathrin-coated vesicles; involved in vesicle-mediated transport |
| 22531 | 0.00761 | 1.238 down | 1.417 up | Membrane coat complex Retromer, subunit Vps29 |
| 110866 | 0.00488 | 1.456 up | 1.253 down | metal-dependent amidase/aminoacylase/carbounknown proteinpeptidase |
| 60671 | 0.00228 | 1.348 down | 1.395 up | metallo-beta-lactamase domain protein, putative |
| 73235 | 0.000794 | 1.101 up | 1.340 up | Metallopeptidase M48, Ste24p, involved in a-factor peptide pheromone processing |
| 45727 | 0.00463 | 1.240 down | 1.306 up | Metallophosphoesterase |
| 120140 | 0.00522 | 1.424 down | 1.128 up | metallophosphoesterase domain-containing protein |
| 81804 | 0.00591 | 1.309 down | 1.115 up | methionine aminopeptidase |
| 62335 | 0.0419 | 1.246 down | 1.230 down | methionine aminopeptidase |
| 82385 | 0.00513 | 1.441 down | 1.053 down | methionyl-tRNA synthetase. |
| 54999 | 0.000286 | 1.380 down | 1.235 down | MFS permease |
| 60945 | 0.000503 | 1.335 down | 1.231 down | MFS permease |
| 47710 | 0.00175 | 1.139 up | 1.168 down | MFS permease |
| 57749 | 0.00395 | 1.165 down | 1.464 down | MFS permease |
| 59272 | 0.00406 | 1.377 down | 1.017 up | MFS permease |
| 44956 | 0.00849 | 1.322 up | 1.062 up | MFS permease |
| 76235 | 0.0266 | 1.008 down | 1.123 down | MFS permease |
| 76992 | 0.023 | 1.225 down | 1.229 up | MFS permease |
| 82296 | 0.0263 | 1.210 down | 1.188 up | MFS permease |
| 111787 | 0.0266 | 1.238 down | 1.153 down | mitochondrial ATP-dependent RNA-helicase Suv3 |
| 56421 | 0.0114 | 1.374 down | 1.278 up | mitochondrial carrier protein |
| 52670 | 0.0229 | 1.285 down | 1.276 up | Mitochondrial carrier protein |
| 75686 | 0.0194 | 1.076 down | 1.145 up | mitochondrial distribution and morphology protein |
| 78765 | 0.0354 | 1.257 up | 1.259 up | mitochondrial DNA replication protein (Yhm2), putative |
| 108082 | 0.000327 | 1.156 down | 1.396 up | mitochondrial membrane fission protein (Fis1), putative |
| 58201 | 0.0305 | 1.083 down | 1.326 down | mitochondrial NADH-ubiquinone oxidoreductase 20 kD subunit |
| 119845 | 0.0451 | 1.419 down | 1.161 up | mitochondrial oxaloacetate/sulfate/thiosulfate transporter-like protein |
| 63441 | 0.00224 | 1.375 down | 1.051 down | mitochondrial precursor proteins import receptor |
| 121890 | 0.017 | 1.424 down | 1.085 up | mitochondrial processing peptidase, α-subunit |
| 4721 | 0.0191 | 1.315 down | 1.283 up | mitochondrial Rho GTPase1 |
| 21749 | 0.0141 | 1.176 up | 1.476 up | mitogen-activated protein kinase MAF1 , putative |
| 23446 | 0.00987 | 1.168 up | 1.131 up | mitotic spindle checkpoint component mad2 |
| 21460 | 0.0321 | 1.457 up | 1.417 up | MMR; HR regulation |
| 35465 | 0.000249 | 1.286 down | 1.257 up | Molecular chaperone Hsp70 family Lhs1 |
| 122551 | 0.000817 | 1.388 down | 1.163 up | mpg1 encoding mannose-1-phosphate guanyltransferase involved in the O-glycosylation pathway. (PMID: 9644208) |
| 3803 | 0.0111 | 1.312 down | 1.237 up | mRNA 3'-end-processing protein RNA14, putative |
| 54450 | 0.00532 | 1.018 up | 1.404 up | mRNA capping enzyme |
| 66111 | 0.000111 | 1.491 down | 1.490 up | MRP-type ABC transporter |
| 22104 | 0.00341 | 1.283 down | 1.297 up | MRP-type ABC transporter |
| 60987 | 0.006 | 1.068 up | 1.036 up | MRP-type ABC transporter |
| 122988 | 0.0193 | 1.256 down | 1.228 up | MRP-type ABC transporter |
| 79510 | 0.0471 | 1.388 down | 1.064 down | MRP-type ABC transporter |
| 104373 | 0.0121 | 1.238 down | 1.378 up | MRSP1/expansin-like |
| 37933 | 0.00305 | 1.111 up | 1.457 up | myb transcriptional regulator |
| 120908 | 0.0195 | 1.440 down | 1.108 up | myb transcriptional regulator |
| 122064 | 0.00101 | 1.452 down | 1.037 up | myosin heavy chain |
| 55790 | 0.0398 | 1.035 down | 1.492 up | N2,N2-dimethylguanosine tRNA methyltransferase. |
| 3412 | 0.0147 | 1.329 down | 1.257 down | Na+/proline symporter PutP |
| 42449 | 0.0131 | 1.315 up | 1.110 up | N-acetyltransferase activity |
| 73511 | 0.00804 | 1.355 up | 1.488 up | NAD kinase |
| 54071 | 0.0392 | 1.019 up | 1.299 up | NAD+ synthetases/Nitrilase/cyanide hydratase/apolipoprotein N-acyltransferase |
| 106617 | 0.029 | 1.308 down | 1.467 down | NADH:ubiquinone oxidoreductase 17.2 kD subunit |
| 62219 | 0.0262 | 1.123 down | 1.409 down | NADH-quinone oxidoreductase |
| 52847 | 0.0387 | 1.134 up | 1.134 down | NADH-ubiquinone oxidoreductase 299 kDa subunit, putative |
| 120154 | 0.0302 | 1.137 up | 1.455 up | NADPH cytochrome P450 oxidoreductase |
| 81646 | 0.00837 | 1.361 up | 1.276 up | NADPH oxidase |
| 103960 | 0.00295 | 1.215 down | 1.316 down | nitrilase |
| 5091 | 0.0286 | 1.424 up | 1.219 up | Nitrilase/cyanide hydratase and apolipoprotein N-acyltransferase |
| 121801 | 0.0212 | 1.144 down | 1.041 up | NOP58; component of the SSU processome. |
| 66012 | 0.00813 | 1.483 down | 1.055 up | nuclear cap-binding protein complex, small subunit |
| 49059 | 0.028 | 1.141 down | 1.156 up | nuclear exosomal RNA helicase, related to N. crassa FRH |
| 122396 | 0.00406 | 1.481 down | 1.094 up | Nuclear transport regulator Npl4 |
| 55766 | 0.00387 | 1.132 up | 1.425 up | nucleolar GTP-binding protein 1 |
| 3001 | 0.0268 | 1.272 down | 1.049 up | nucleolar protein 12 |
| 56384 | 0.00357 | 1.199 down | 1.141 up | Nucleolar protein that binds nuclear localization sequences |
| 47221 | 0.0211 | 1.304 down | 1.486 down | Nucleoside diphosphate kinase |
| 79324 | 0.00654 | 1.289 down | 1.390 down | nucleoside-diphosphate-sugar epimerase, putative (check!) |
| 55335 | 0.0424 | 1.127 down | 1.257 up | nucleotide binding protein Nbp35, putative |
| 67698 | 0.00281 | 1.416 down | 1.120 down | nucleotide sugar dehydrogenase (UDP-Glc?) |
| 119600 | 0.0283 | 1.280 down | 1.325 up | Oligosaccharyltransferase, alpha subunit |
| 74601 | 0.0442 | 1.183 up | 1.323 up | OPT family small oligopeptide transporter |
| 49979 | 0.00189 | 1.440 down | 1.473 up | OPT oligopeptide transporter, putative |
| 77661 | 0.0223 | 1.332 up | 1.382 up | ounknown proteinsterol binding protein |
| 77218 | 0.0254 | 1.346 down | 1.397 up | outer membrane protein TOM13 |
| 58563 | 0.0319 | 1.355 down | 1.138 up | oxalate decarboxylase |
| 55353 | 0.0168 | 1.368 up | 1.437 up | pathotenate kinase |
| 58150 | 0.00269 | 1.200 down | 1.159 up | Peptidase C19, ubiquitin carboxyl-terminal hydrolase 2 |
| 75258 | 0.0111 | 1.154 down | 1.333 up | Peptidase C48, SUMO/Sentrin/Ubl1 |
| 23475 | 0.00156 | 1.018 up | 1.021 down | Peptidase family M28 protein |
| 122431 | 0.0158 | 1.023 down | 1.277 up | Pex19 (peroxin), a 40 kDa farnesylated protein associated with peroxisomes. |
| 80151 | 0.0116 | 1.392 down | 1.041 up | PheA Prephenate dehydratase |
| 58856 | 0.0109 | 1.319 down | 1.046 up | phosducin like protein, class I |
| 82150 | 0.00702 | 1.085 up | 1.351 up | phosphate transporter (Pho88), putative |
| 81389 | 0.0108 | 1.046 up | 1.065 down | phosphate/H+ symporter |
| 51806 | 0.0106 | 1.327 up | 1.438 up | Phosphatidate cytidylyltransferase |
| 29346 | 0.000323 | 1.070 up | 1.275 up | phosphatidate-phosphatase |
| 66913 | 0.0228 | 1.391 up | 1.388 up | Phosphatidylinositol 3-kinase Vps34 |
| 81972 | 0.0253 | 1.366 down | 1.098 down | phosphatidylinositol transporter, putative |
| 60194 | 0.0204 | 1.376 down | 1.388 up | phosphatidylinositol-specific phospholipase C (MPLC1) |
| 121498 | 0.00866 | 1.421 up | 1.410 down | Phosphatidylserine decarboxylase |
| 82326 | 0.0252 | 1.137 up | 1.423 up | phosphoglycerate mutase family protein |
| 122592 | 0.00438 | 1.489 down | 1.382 up | PhosphoinositidePhosphatase, sac1, involved inProtein trafficking and secretion |
| 55631 | 0.00255 | 1.219 down | 1.319 down | phospholipase |
| 80872 | 0.00961 | 1.197 down | 1.208 down | phosphoribosylamine-glycine ligase |
| 120498 | 0.0104 | 1.176 up | 1.436 up | Phosphotyrosyl phosphatase activator, PTPA |
| 81964 | 0.0319 | 1.444 down | 1.333 up | PKS |
| 78757 | 0.0375 | 1.413 down | 1.208 down | plasma membrane H+ ATPase |
| 57252 | 0.00789 | 1.491 down | 1.118 up | polyadenylation factor subunit CstF64 |
| 55099 | 0.0411 | 1.096 up | 1.228 up | polynucleotide adenylyltransferase. |
| 60771 | 0.0372 | 1.418 down | 1.006 up | prefoldin chaperone |
| 58036 | 0.0196 | 1.092 up | 1.462 up | prefoldin subunit 1, putative |
| 58734 | 0.0291 | 1.437 down | 1.167 up | prefoldin subunit 2 |
| 78109 | 0.0308 | 1.432 down | 1.209 up | pre-mRNA processing splicing factor 8 |
| 122864 | 0.00249 | 1.443 down | 1.223 up | pre-mRNA splicing factor |
| 78939 | 0.00616 | 1.478 down | 1.062 up | pre-mRNA splicing helicase |
| 22598 | 0.0053 | 1.027 up | 1.359 up | pre-mRNA-processing factor 39 |
| 75739 | 0.0139 | 1.469 down | 1.323 down | pre-mRNA-splicing factor clf1 |
| 50882 | 0.00804 | 1.217 down | 1.414 up | pre-mRNA-splicing factor CWC25, putative |
| 102603 | 0.0381 | 1.197 down | 1.430 up | Pre-mRNA-splicing factor ini1 |
| 5095 | 0.0166 | 1.178 up | 1.284 up | Pre-mRNA-splicing factor ISY1 |
| 75965 | 0.029 | 1.124 down | 1.354 up | Pre-mRNA-splicing factor RSE1 |
| 81599 | 0.00199 | 1.386 down | 1.466 down | pre-rRNA processing nucleolar protein Sik1, putative |
| 4294 | 0.0462 | 1.089 down | 1.023 down | pre-rRNA processing protein Rrp12 |
| 73873 | 0.00452 | 1.040 up | 1.259 down | ProB Glutamate -kinase [Amino acid transport and metabolism] |
| 123364 | 0.0169 | 1.308 down | 1.352 up | Protein involved in ER to Golgi transport Sly41 |
| 45456 | 0.00739 | 1.254 down | 1.022 down | Protein kinase |
| 2526 | 0.00298 | 1.302 up | 1.380 up | Protein kinase C |
| 120545 | 0.0165 | 1.236 down | 1.173 up | protein phosphatase 2A regulatory B subunit |
| 77135 | 0.0469 | 1.144 up | 1.460 up | Protein phosphatase 2A, regulatory B subunit, B56 |
| 124001 | 0.0144 | 1.339 down | 1.098 down | Protein phosphatase 2C/pyruvate dehydrogenase (lipoamide) phosphatase |
| 81164 | 0.0199 | 1.235 down | 1.031 down | Protein phosphatase type 2C Ptc2 |
| 2561 | 0.00769 | 1.107 down | 1.432 up | Protein transport protein Sec1 |
| 53004 | 0.025 | 1.302 down | 1.098 up | protein tyrosine phosphatase activity, M phase of mitotic cell cycle |
| 22826 | 0.000682 | 1.365 down | 1.264 up | PrsA Phosphoribosylpyrophosphate synthetase |
| 121990 | 0.0263 | 1.290 down | 1.117 up | PTH11 GPCR |
| 25159 | 0.00243 | 1.399 up | 1.308 up | PTPc, Protein tyrosine phosphatases (PTP) |
| 56267 | 0.0148 | 1.106 up | 1.252 down | pyridoxal kinase |
| 63001 | 0.00263 | 1.367 down | 1.431 up | pyridoxamine phosphate oxidase family protein |
| 21957 | 0.0214 | 1.246 down | 1.351 up | pyruvate carboxylase (cytosolic) |
| 81855 | 0.00182 | 1.359 down | 1.392 up | pyruvate dehydrogenase kinase, putative |
| 120150 | 0.0105 | 1.106 down | 1.062 up | RAS like small GTPase, Ras subfamily |
| 78314 | 0.0171 | 1.194 up | 1.129 up | RgsB, regulator of G-protein signaling |
| 21294 | 0.00465 | 1.325 down | 1.463 up | rho2 |
| 110678 | 0.0086 | 1.250 down | 1.279 up | riboflavin synthase |
| 110423 | 0.0176 | 1.113 up | 1.457 up | Ribonuclease CAF1 |
| 59751 | 0.0201 | 1.133 up | 1.361 up | Ribonucleases P/MRP protein subunit POP1 containing protein |
| 75564 | 0.0268 | 1.031 up | 1.396 down | Ribosomal protein (60S) L11 |
| 51430 | 0.0427 | 1.225 down | 1.236 down | ribosomal protein L10e. |
| 122001 | 0.0145 | 1.034 down | 1.396 down | ribosomal protein L14b/L23e. |
| 72918 | 0.0325 | 1.318 down | 1.227 up | ribosomal protein L19 |
| 34112 | 0.0078 | 1.308 down | 1.438 down | ribosomal protein L22. |
| 48170 | 0.0052 | 1.250 down | 1.307 down | ribosomal protein L27e. Homologue of yeast RPL27a/b. |
| 122141 | 0.0043 | 1.084 down | 1.361 down | Ribosomal protein L28 (L29/L15, rp44, YL24 family). Homologue of S. cerevisiae RPL28. |
| 55752 | 0.00422 | 1.218 up | 1.424 down | ribosomal protein L29. Shows amino acid sequence similarity to S. cerevisiae mitochondrial ribosomal protein MRPL4. |
| 21890 | 0.00172 | 1.167 up | 1.329 down | Ribosomal protein L32 based on homology to the corresponding protein in N. crassa. |
| 73231 | 0.00938 | 1.103 down | 1.355 down | ribosomal protein L34. |
| 120621 | 0.037 | 1.189 down | 1.420 down | ribosomal protein L37 of the large (60S) ribosomal subunit. Belongs to the same family as yeast Rpl33Ap and has similarity to rat L35a. |
| 120020 | 0.0228 | 1.022 down | 1.463 down | ribosomal protein L37e from several filamentous fungi and yeasts. |
| 74409 | 0.00482 | 1.372 down | 1.454 down | Ribosomal protein L5 domain. |
| 124110 | 0.0497 | 1.184 up | 1.337 down | Ribosomal protein L8 |
| 122900 | 0.00579 | 1.369 down | 1.047 up | ribosomal protein RSM22. |
| 120868 | 0.0406 | 1.317 down | 1.226 down | Ribosomal protein S13 (S15 family) based on homology to the corresponding protein in Gibberella zeae. |
| 81713 | 0.0236 | 1.333 down | 1.339 down | ribosomal protein S24e. Homologue of yeast RPS24a/b. |
| 122956 | 0.0388 | 1.172 down | 1.172 down | Ribosomal protein S27 based on homology to the corresponding protein of N. crassa. |
| 121837 | 0.0144 | 1.229 down | 1.256 down | Ribosomal protein S5, S7 family |
| 61804 | 0.00223 | 1.296 down | 1.408 down | ribosomal protein YmL32 precursor. Contains possible InterPro domain for ribosomal L32p proteins. |
| 56631 | 0.00374 | 1.455 down | 1.160 down | ribulose-phosphate 3-epimerase |
| 77667 | 0.00961 | 1.496 down | 1.275 up | RING-5 |
| 2716 | 0.00584 | 1.486 down | 1.271 down | RNA 3'-terminal phosphate cyclase |
| 50712 | 0.00697 | 1.403 down | 1.381 up | RNA binding protein |
| 103993 | 0.00712 | 1.028 up | 1.003 up | RNA exonuclease 4 |
| 80748 | 0.0181 | 1.213 down | 1.418 down | RNA polymerase I specific transcription stimulatory factor composed of Uaf30p |
| 81742 | 0.00159 | 1.104 down | 1.489 down | RNA polymerase I subunit Rpa43, putative |
| 1847 | 0.00148 | 1.050 down | 1.485 up | RNA polymerase II transcription elongation factor (Ctr9) |
| 5064 | 0.0212 | 1.107 up | 1.442 up | RNA polymerase III subunitRpc25 |
| 79315 | 0.0128 | 1.181 down | 1.374 up | RNA polymerase Rpb1 |
| 55185 | 0.0182 | 1.284 down | 1.025 up | RNA polymerase Rpb5 |
| 104015 | 0.0172 | 1.103 up | 1.153 up | RNA polymerase Rpb8 |
| 4040 | 0.00178 | 1.105 up | 1.240 up | RNA polymerase Rpc34 subunit |
| 78543 | 0.0249 | 1.153 up | 1.403 up | RNA-binding La domain protein |
| 77747 | 0.0112 | 1.153 up | 1.109 up | RNA-binding protein Vip1 |
| 73895 | 0.0118 | 1.149 up | 1.411 up | RNA-binding protein with KH domain |
| 75534 | 0.0264 | 1.364 down | 1.038 down | RNA-binding protein with KH domain |
| 65750 | 0.0171 | 1.444 down | 1.458 up | RNA-binding, negative regulator of differentiation 1 |
| 75609 | 0.000776 | 1.311 down | 1.107 down | Rpl18 gene encoding 60sRibosomal protein L18. |
| 46490 | 0.0114 | 1.196 down | 1.429 down | Rps16 gene encoding protein component of the small (40S)Ribosomal subunit (S9 family). |
| 58299 | 0.0328 | 1.371 down | 1.223 up | RRB1 gene encoding an essential nuclear protein involved in early steps ofRibosome biogenesis. |
| 78062 | 0.0133 | 1.335 down | 1.082 up | rRNA biogenesis protein RRP5 |
| 119554 | 0.00148 | 1.005 up | 1.382 up | rRNA processing protein Bystin |
| 55029 | 0.00325 | 1.424 down | 1.082 up | rRNA processing protein Rrp8 |
| 56291 | 0.000657 | 1.383 up | 1.323 down | SAM dependent methyltransferase |
| 110877 | 0.00128 | 1.492 up | 1.146 up | SAM dependent methyltransferase, putative |
| 60338 | 0.0154 | 1.340 down | 1.136 up | SAM methyltransferase, tRNA-methylztransferase subunit GCD14 (yeast). |
| 62643 | 0.00236 | 1.071 up | 1.465 up | SAM-dependent methyltransferases |
| 110409 | 0.0391 | 1.339 down | 1.479 down | SAS10, encoding a component of theSSU processosome required for pre-18S rRNa processing. |
| 55706 | 0.007 | 1.010 down | 1.472 up | SCF ubiquitin ligase complex subunit, cullin |
| 79166 | 0.00507 | 1.226 up | 1.017 up | SCP-2 sterol transfer family protein |
| 65623 | 0.0043 | 1.483 up | 1.025 down | Sec61 gamma subunit |
| 5209 | 0.0105 | 1.165 up | 1.189 up | Secretion related small GTPase Rab11/Ypt3 |
| 121223 | 0.0139 | 1.261 up | 1.426 up | Secretion related small GTPase Sec4 |
| 59544 | 0.0212 | 1.495 down | 1.466 up | SEN1 (tRNA splicing complex component). |
| 22874 | 0.0141 | 1.299 down | 1.371 up | septin-like protein |
| 64249 | 0.0364 | 1.091 up | 1.030 up | septum formation protein Maf |
| 81136 | 0.00212 | 1.378 down | 1.377 down | serine/threonine phosphatase 2 C, PTC2 |
| 53402 | 0.0191 | 1.029 down | 1.300 up | Serine/threonine protein kinase |
| 82175 | 0.0309 | 1.204 up | 1.100 up | serine/threonine protein kinase |
| 78909 | 0.0143 | 1.341 down | 1.043 up | serine/threonine protein kinase cot1 |
| 79535 | 0.0115 | 1.085 down | 1.069 up | serine/threonine protein phosphatase |
| 120722 | 0.0282 | 1.174 up | 1.418 up | Serine/threonine protein phosphatases |
| 69496 | 0.0182 | 1.007 down | 1.433 up | serine/threonine-protein kinase csk1 |
| 53647 | 0.0415 | 1.101 up | 1.339 up | serine/threonine-protein kinase hal4 |
| 81007 | 0.00118 | 1.421 down | 1.327 up | serine/threonine-protein kinase RIO1 |
| 82442 | 0.0195 | 1.029 up | 1.347 up | Several tryptophanyl-tRNASynthetases from other fungi. |
| 120288 | 0.00252 | 1.022 up | 1.051 up | short chain dehydrogenase/reductase |
| 76288 | 0.00508 | 1.498 down | 1.102 up | short chain dehydrogenase/reductase |
| 124136 | 0.0173 | 1.049 up | 1.215 down | short chain dehydrogenase/reductase |
| 104103 | 0.0215 | 1.290 up | 1.271 down | short chain dehydrogenase/reductase |
| 105884 | 0.0311 | 1.035 down | 1.388 up | short chain dehydrogenase/reductase |
| 70912 | 0.00913 | 1.076 up | 1.255 down | short chain dehydrogenase/reductase |
| 107526 | 0.00203 | 1.421 down | 1.358 up | short chain dehydrognease/reductase |
| 59700 | 0.0139 | 1.008 up | 1.099 up | short chain dehydrognease/reductase |
| 62003 | 0.0458 | 1.056 down | 1.002 up | signalosome subunit 5 (CsnE) putatively involved in regulation of sexual development |
| 67057 | 0.000753 | 1.444 down | 1.210 up | silencing information regulator, Sir2 family |
| 57935 | 0.0336 | 1.033 up | 1.370 up | snf2 chromatin remodeling protein |
| 75072 | 0.0394 | 1.130 down | 1.459 up | Snf7 family protein |
| 123111 | 0.00853 | 1.191 down | 1.303 up | Sorting nexin-1-like protein Vps5 |
| 21214 | 0.00521 | 1.073 down | 1.273 down | Sorting nexin-41 |
| 65490 | 0.0284 | 1.313 up | 1.248 up | SpermidineSynthase |
| 68412 | 0.00071 | 1.256 down | 1.420 up | Sphingoid long-chain base kinase |
| 75535 | 0.0202 | 1.211 up | 1.266 up | sphingomyelinase family protein, putative |
| 109652 | 0.000131 | 1.282 up | 1.373 up | sphingosine N-acyltransferase lac1 |
| 76191 | 0.000653 | 1.240 down | 1.359 up | splicing factor 3B subunit 1, putative |
| 122653 | 0.0323 | 1.367 down | 1.107 down | squalene synthase |
| 56605 | 0.00347 | 1.042 up | 1.493 up | ß-arrestin, probable adaptor and transducer involved in signalling, related to the E. nidulans pH-response regulator protein palF |
| 3043 | 0.00202 | 1.252 down | 1.108 up | SSCRP |
| 121312 | 0.00548 | 1.407 down | 1.163 down | SSCRP |
| 104206 | 0.0126 | 1.144 up | 1.403 up | SSCRP |
| 70840 | 0.0154 | 1.247 up | 1.036 down | SSCRP |
| 104354 | 0.0215 | 1.123 down | 1.489 up | SSCRP |
| 54723 | 0.0217 | 1.377 down | 1.405 up | SSCRP |
| 39156 | 0.024 | 1.282 down | 1.014 up | SSCRP |
| 124173 | 0.0342 | 1.091 down | 1.175 up | SSCRP |
| 81331 | 0.0397 | 1.253 up | 1.253 up | SSCRP |
| 108412 | 0.0403 | 1.419 down | 1.102 up | SSCRP |
| 121177 | 0.0406 | 1.241 down | 1.333 down | SSCRP |
| 50607 | 0.017 | 1.278 down | 1.094 up | Sterol O-acyltransferase/Diacylglycerol O-acyltransferase |
| 4240 | 0.000995 | 1.027 down | 1.275 down | stress response protein Rds1; secreted |
| 61750 | 0.00367 | 1.301 down | 1.232 down | Sur2 sphinganine C4-hydroxylase of Saccharomyces cerevisiae |
| 74782 | 0.00547 | 1.437 down | 1.025 up | survival factor 1 |
| 80835 | 0.0229 | 1.407 down | 1.127 up | SWI/SNF complex protein |
| 103804 | 0.00475 | 1.354 down | 1.247 down | SWI-SNF chromatin-remodeling complex protein |
| 54670 | 0.00261 | 1.453 down | 1.373 up | SWI-SNF complex subunit (Snf5), putative |
| 57720 | 0.00247 | 1.495 down | 1.282 up | Swr1p complex component (Swc5), putative |
| 54852 | 0.0419 | 1.150 up | 1.453 up | SYF2 splicing factor |
| 111788 | 0.00338 | 1.313 down | 1.432 up | Synaptobrevin/VAMP-like protein (VAMP71 family) |
| 77485 | 0.00557 | 1.176 down | 1.422 up | T-complex protein 1 |
| 120994 | 0.0289 | 1.430 up | 1.053 up | T-complex protein 1 |
| 46446 | 0.00462 | 1.184 down | 1.395 up | Thioredoxin binding protein TBP-2 |
| 75568 | 0.00504 | 1.458 down | 1.017 up | thioredoxin, putative |
| 78409 | 0.0437 | 1.465 down | 1.013 down | ThrC Threonine synthase |
| 123223 | 0.00207 | 1.418 up | 1.091 down | threonine aldolase GLY1 |
| 77266 | 0.0036 | 1.488 up | 1.189 up | Thymidylate kinase |
| 55660 | 0.00181 | 1.342 up | 1.056 up | Transcription factor TFIIS |
| 46902 | 0.00783 | 1.254 down | 1.094 up | Transcriptional activator HAC1, UPR regulator |
| 122387 | 0.0045 | 1.212 down | 1.222 up | transcriptional regulator HMG type |
| 3007 | 0.0263 | 1.411 down | 1.306 down | transcriptional regulator HMG type |
| 104004 | 0.00224 | 1.206 up | 1.403 up | transcriptional regulator, unknown |
| 112554 | 0.00622 | 1.019 down | 1.244 up | transcriptional regulator, unknown |
| 23154 | 0.0139 | 1.010 up | 1.390 up | transcriptional regulator, unknown |
| 120635 | 0.0352 | 1.085 down | 1.116 up | transketolase-like protein |
| 82613 | 0.0113 | 1.296 down | 1.035 down | translation elongation factor 1 beta by homology to the corresponding gene in other fungi. |
| 46958 | 0.00565 | 1.473 down | 1.454 down | Translation elongation factor 1a |
| 62345 | 0.00434 | 1.220 up | 1.202 up | translation initiation factor ? |
| 74498 | 0.0304 | 1.039 down | 1.245 up | translation initiation factor 2 beta subunit (Interpro motif IF5). |
| 74774 | 0.00629 | 1.440 down | 1.072 down | Translation initiation factor 3, subunit g (eIF-3g); homologue of yeastTIF35. |
| 57676 | 0.0286 | 1.444 down | 1.092 down | Translation initiation factor 3, subunit i (elf-3i) |
| 103322 | 0.0305 | 1.211 up | 1.079 down | translation initiation factor 4e [Neurospora crassa]. |
| 74252 | 0.0287 | 1.392 down | 1.063 down | Translation initiation factor 6 (eIF6) by homologyToThe corresponding protein in other eukaryotes. |
| 75954 | 0.00488 | 1.292 down | 1.161 up | translation initiation factor eIF3 subunit. |
| 71410 | 0.0282 | 1.070 down | 1.315 up | translation initiation factor eIF4-gamma/eIF5/eIF2-epsilon. |
| 76927 | 0.00435 | 1.022 down | 1.105 up | translation initiation factor SUI1. |
| 55969 | 0.00988 | 1.390 down | 1.079 down | Translation initiation factor, eIF1A. |
| 70305 | 0.0248 | 1.295 down | 1.113 up | Translation release factor eRF3 in other fungi. |
| 104639 | 0.00895 | 1.279 down | 1.489 up | Transport protein Uso1 |
| 61971 | 0.0131 | 1.377 down | 1.102 down | TRAPP complex component Trs33 |
| 67507 | 0.021 | 1.482 down | 1.453 down | Triacylglycerol lipase |
| 78004 | 0.00425 | 1.487 down | 1.137 up | triose phosphate/3-phosphoglycerate/phosphate translocator |
| 74356 | 0.0338 | 1.144 down | 1.374 down | tRNA (cytosine-5-)-methyltransferase NCL1 |
| 78693 | 0.0129 | 1.272 up | 1.179 up | tRNA intron endonuclease, N-terminal. |
| 68582 | 0.0126 | 1.346 down | 1.236 down | tRNA ligase TRL1 |
| 60402 | 0.006 | 1.293 down | 1.029 up | tRNA modification GTPase TrmE, putative |
| 21549 | 0.00444 | 1.364 down | 1.048 up | tRNA-splicing endonuclease subunit (tRNA-intron endonuclease). |
| 3340 | 0.00458 | 1.337 down | 1.184 up | t-SNARE syntaxin,cis-Golgi |
| 120402 | 0.0106 | 1.495 up | 1.141 down | tubulin-tyrosine ligase |
| 27406 | 0.00632 | 1.424 down | 1.498 up | tyrosine-protein phosphatase CDC14 |
| 58055 | 0.0367 | 1.114 down | 1.075 up | UbiA prenyltransferase containing 9 transmembrane domains |
| 121359 | 0.049 | 1.063 up | 1.362 up | Ubiquitin associated protein probably involved in cell signalling via protein kinases and theUbiquitin/proteasome pathway |
| 109797 | 0.00228 | 1.352 down | 1.283 down | ubiquitin carboxyl-terminal hydrolase |
| 106205 | 0.0119 | 1.450 down | 1.122 up | ubiquitin carboxyl-terminal hydrolase 2, putative |
| 77732 | 0.00372 | 1.226 down | 1.221 up | Ubiquitin conjugating enzyme Ubc6 |
| 53498 | 0.00394 | 1.157 down | 1.254 up | ubiquitin related modifier 1 |
| 123559 | 0.012 | 1.144 down | 1.230 down | ubiquitin-conjugating enzyme |
| 22891 | 0.0364 | 1.448 down | 1.034 down | ubiquitin-conjugating enzyme |
| 123773 | 0.0434 | 1.329 down | 1.075 down | Ubiquitin-conjugating enzyme |
| 120075 | 0.00499 | 1.256 up | 1.409 up | Ubiquitin-conjugating enzyme (E2) |
| 57208 | 0.00388 | 1.465 up | 1.146 up | ubiquitin-like modifier SUMO, putative |
| 65672 | 0.0187 | 1.498 down | 1.431 down | ubiquitin-protein ligase molybdopterin-converting factor |
| 48178 | 0.00183 | 1.331 down | 1.158 up | UDP-galactopyranose mutase |
| 79568 | 0.0021 | 1.445 down | 1.206 up | UDP-N-acetylglucosamine pyrophosphorylase |
| 107524 | 0.000188 | 1.278 down | 1.356 up | unique protein |
| 105150 | 0.00055 | 1.012 down | 1.029 down | unique protein |
| 106223 | 0.000722 | 1.069 down | 1.296 down | unique protein |
| 123649 | 0.00166 | 1.065 up | 1.389 up | unique protein |
| 109476 | 0.00218 | 1.210 down | 1.490 down | unique protein |
| 106647 | 0.00255 | 1.299 up | 1.192 down | unique protein |
| 107112 | 0.00398 | 1.025 down | 1.494 up | unique protein |
| 102680 | 0.0069 | 1.113 down | 1.430 up | unique protein |
| 109044 | 0.00696 | 1.005 up | 1.488 up | unique protein |
| 104202 | 0.00795 | 1.250 up | 1.436 up | unique protein |
| 110825 | 0.00872 | 1.082 down | 1.227 down | unique protein |
| 112094 | 0.00889 | 1.002 up | 1.029 down | unique protein |
| 106470 | 0.00938 | 1.331 down | 1.110 up | unique protein |
| 104289 | 0.0104 | 1.412 down | 1.477 up | unique protein |
| 104533 | 0.0104 | 1.168 down | 1.472 up | unique protein |
| 120030 | 0.0123 | 1.253 up | 1.015 up | unique protein |
| 103016 | 0.0127 | 1.190 down | 1.456 up | unique protein |
| 107339 | 0.0142 | 1.132 up | 1.394 down | unique protein |
| 106482 | 0.0169 | 1.053 up | 1.171 down | unique protein |
| 104399 | 0.0173 | 1.490 down | 1.487 up | unique protein |
| 110752 | 0.0177 | 1.140 down | 1.220 up | unique protein |
| 105866 | 0.0184 | 1.376 up | 1.071 down | unique protein |
| 103653 | 0.0202 | 1.302 down | 1.372 up | unique protein |
| 107513 | 0.021 | 1.122 down | 1.432 up | unique protein |
| 109648 | 0.0215 | 1.133 up | 1.389 up | unique protein |
| 104157 | 0.0241 | 1.205 down | 1.206 up | unique protein |
| 109608 | 0.026 | 1.210 down | 1.315 up | unique protein |
| 110153 | 0.0272 | 1.102 down | 1.198 up | unique protein |
| 104695 | 0.0278 | 1.032 up | 1.170 up | unique protein |
| 104715 | 0.028 | 1.465 down | 1.103 up | unique protein |
| 112604 | 0.0289 | 1.349 down | 1.376 up | unique protein |
| 104220 | 0.0299 | 1.255 down | 1.221 down | unique protein |
| 107595 | 0.037 | 1.063 down | 1.374 up | unique protein |
| 109320 | 0.0373 | 1.285 down | 1.211 up | unique protein |
| 107298 | 0.0382 | 1.053 up | 1.109 up | unique protein |
| 106236 | 0.0395 | 1.221 up | 1.016 down | unique protein |
| 112605 | 0.0398 | 1.321 down | 1.135 up | unique protein |
| 107867 | 0.04 | 1.108 down | 1.466 up | unique protein |
| 104228 | 0.0414 | 1.088 down | 1.358 up | unique protein |
| 112688 | 0.0446 | 1.110 down | 1.226 up | unique protein |
| 109726 | 0.0455 | 1.225 down | 1.134 up | unique protein |
| 112603 | 0.0469 | 1.394 down | 1.213 up | unique protein |
| 123951 | 0.0176 | 1.481 down | 1.113 up | unknown ER membrane protein |
| 123047 | 0.0000713 | 1.277 down | 1.476 up | unknown protein |
| 77699 | 0.000189 | 1.278 down | 1.248 down | unknown protein |
| 78117 | 0.000232 | 1.384 down | 1.128 up | unknown protein |
| 59100 | 0.000308 | 1.099 up | 1.035 up | unknown protein |
| 21442 | 0.000323 | 1.158 down | 1.306 up | unknown protein |
| 56396 | 0.000361 | 1.191 down | 1.252 up | unknown protein |
| 107871 | 0.000442 | 1.140 down | 1.243 down | unknown protein |
| 77099 | 0.000444 | 1.465 down | 1.464 up | unknown protein |
| 21924 | 0.000514 | 1.241 down | 1.487 up | unknown protein |
| 74997 | 0.000544 | 1.100 down | 1.209 up | unknown protein |
| 2231 | 0.000628 | 1.348 up | 1.208 down | unknown protein |
| 21345 | 0.000638 | 1.209 down | 1.473 up | unknown protein |
| 77577 | 0.00066 | 1.165 down | 1.481 up | unknown protein |
| 23268 | 0.000663 | 1.126 up | 1.057 down | unknown protein |
| 58837 | 0.000692 | 1.162 up | 1.114 up | unknown protein |
| 103844 | 0.000712 | 1.425 up | 1.452 up | unknown protein |
| 80904 | 0.000795 | 1.226 down | 1.319 up | unknown protein |
| 33763 | 0.000812 | 1.147 down | 1.357 up | unknown protein |
| 52553 | 0.000823 | 1.293 down | 1.233 up | unknown protein |
| 58114 | 0.000871 | 1.415 down | 1.184 down | unknown protein |
| 82001 | 0.000889 | 1.403 down | 1.350 up | unknown protein |
| 121793 | 0.000946 | 1.177 down | 1.240 up | unknown protein |
| 121304 | 0.000957 | 1.144 down | 1.448 up | unknown protein |
| 120915 | 0.000996 | 1.207 up | 1.020 up | unknown protein |
| 21752 | 0.00101 | 1.487 down | 1.036 up | unknown protein |
| 102901 | 0.00105 | 1.205 down | 1.453 up | unknown protein |
| 21919 | 0.00107 | 1.305 down | 1.210 up | unknown protein |
| 122499 | 0.0011 | 1.331 down | 1.270 up | unknown protein |
| 123321 | 0.00111 | 1.218 up | 1.066 up | unknown protein |
| 64271 | 0.00112 | 1.369 down | 1.130 up | unknown protein |
| 79129 | 0.00117 | 1.481 down | 1.083 up | unknown protein |
| 122023 | 0.00127 | 1.110 up | 1.454 up | unknown protein |
| 1777 | 0.00129 | 1.355 down | 1.374 up | unknown protein |
| 76539 | 0.00134 | 1.018 down | 1.386 up | unknown protein |
| 69425 | 0.00135 | 1.312 down | 1.454 up | unknown protein |
| 53796 | 0.00138 | 1.487 down | 1.426 up | unknown protein |
| 78322 | 0.00141 | 1.193 down | 1.202 up | unknown protein |
| 111569 | 0.00153 | 1.366 up | 1.311 up | unknown protein |
| 55052 | 0.00156 | 1.185 up | 1.272 up | unknown protein |
| 103720 | 0.00159 | 1.265 down | 1.484 down | unknown protein |
| 106287 | 0.00159 | 1.101 up | 1.458 up | unknown protein |
| 64644 | 0.00161 | 1.368 down | 1.377 up | unknown protein |
| 122230 | 0.00165 | 1.227 up | 1.193 down | unknown protein |
| 4097 | 0.00171 | 1.075 up | 1.478 up | unknown protein |
| 107389 | 0.00173 | 1.241 down | 1.391 up | unknown protein |
| 65743 | 0.00174 | 1.250 up | 1.097 down | unknown protein |
| 109045 | 0.00175 | 1.011 down | 1.447 up | unknown protein |
| 102763 | 0.00183 | 1.273 up | 1.431 up | unknown protein |
| 60508 | 0.00187 | 1.305 down | 1.076 up | unknown protein |
| 82356 | 0.00194 | 1.485 up | 1.322 down | unknown protein |
| 120583 | 0.00204 | 1.270 down | 1.120 up | unknown protein |
| 124104 | 0.00207 | 1.269 up | 1.015 down | unknown protein |
| 108458 | 0.00213 | 1.181 down | 1.133 down | unknown protein |
| 44178 | 0.00217 | 1.128 up | 1.057 up | unknown protein |
| 121777 | 0.00218 | 1.443 down | 1.123 up | unknown protein |
| 47621 | 0.00222 | 1.368 up | 1.427 up | unknown protein |
| 64112 | 0.00228 | 1.422 down | 1.172 up | unknown protein |
| 56399 | 0.00231 | 1.225 up | 1.114 down | unknown protein |
| 75385 | 0.00233 | 1.369 down | 1.020 up | unknown protein |
| 21658 | 0.00239 | 1.374 down | 1.413 down | Unknown protein |
| 79300 | 0.00252 | 1.343 down | 1.284 up | unknown protein |
| 120485 | 0.00254 | 1.159 down | 1.332 up | unknown protein |
| 109353 | 0.00265 | 1.358 down | 1.148 up | unknown protein |
| 122050 | 0.00269 | 1.195 down | 1.142 up | unknown protein |
| 57666 | 0.0027 | 1.378 down | 1.124 up | unknown protein |
| 61043 | 0.00274 | 1.405 up | 1.102 up | unknown protein |
| 30018 | 0.00276 | 1.272 down | 1.307 up | unknown protein |
| 103114 | 0.00281 | 1.171 down | 1.390 up | unknown protein |
| 123508 | 0.00284 | 1.248 up | 1.389 up | unknown protein |
| 43191 | 0.00289 | 1.376 down | 1.476 up | unknown protein |
| 76083 | 0.00289 | 1.023 down | 1.461 up | unknown protein |
| 103127 | 0.00292 | 1.293 down | 1.124 down | unknown protein |
| 77850 | 0.00292 | 1.134 down | 1.463 up | unknown protein |
| 79287 | 0.00303 | 1.458 down | 1.262 up | unknown protein |
| 75334 | 0.0031 | 1.124 down | 1.484 up | unknown protein |
| 22559 | 0.00312 | 1.453 down | 1.003 up | unknown protein |
| 112536 | 0.00314 | 1.092 up | 1.376 down | unknown protein |
| 110791 | 0.00316 | 1.415 down | 1.307 up | unknown protein |
| 52960 | 0.0032 | 1.439 down | 1.383 up | unknown protein |
| 78966 | 0.00329 | 1.351 down | 1.181 up | unknown protein |
| 63382 | 0.00341 | 1.339 down | 1.366 up | unknown protein |
| 3783 | 0.00342 | 1.336 down | 1.394 up | unknown protein |
| 53027 | 0.00342 | 1.145 down | 1.438 up | unknown protein |
| 70377 | 0.00346 | 1.228 down | 1.056 down | unknown protein |
| 63542 | 0.00352 | 1.248 down | 1.154 up | unknown protein |
| 70927 | 0.00352 | 1.219 down | 1.301 up | unknown protein |
| 78415 | 0.00353 | 1.278 down | 1.318 up | unknown protein |
| 120524 | 0.00353 | 1.195 down | 1.455 up | unknown protein |
| 107058 | 0.00362 | 1.189 up | 1.061 down | unknown protein |
| 63632 | 0.00364 | 1.389 down | 1.005 down | unknown protein |
| 21426 | 0.00364 | 1.265 down | 1.371 up | unknown protein |
| 112491 | 0.00371 | 1.395 down | 1.099 down | unknown protein |
| 111239 | 0.00373 | 1.301 up | 1.399 up | unknown protein |
| 112001 | 0.00377 | 1.025 down | 1.337 up | unknown protein |
| 76123 | 0.00384 | 1.287 up | 1.295 up | unknown protein |
| 102935 | 0.00396 | 1.384 down | 1.287 up | unknown protein |
| 65927 | 0.00404 | 1.014 down | 1.486 down | unknown protein |
| 5871 | 0.00406 | 1.306 up | 1.396 up | unknown protein |
| 122914 | 0.00407 | 1.486 down | 1.219 up | unknown protein |
| 109958 | 0.00413 | 1.460 up | 1.005 up | unknown protein |
| 66474 | 0.00413 | 1.289 up | 1.408 up | unknown protein |
| 110452 | 0.00414 | 1.346 down | 1.437 up | unknown protein |
| 79919 | 0.00418 | 1.371 down | 1.154 up | unknown protein |
| 68479 | 0.00421 | 1.457 down | 1.350 down | unknown protein |
| 66210 | 0.00436 | 1.410 down | 1.176 down | unknown protein |
| 102985 | 0.00436 | 1.077 up | 1.241 up | unknown protein |
| 56720 | 0.0045 | 1.303 down | 1.356 up | unknown protein |
| 45980 | 0.00456 | 1.177 down | 1.239 up | unknown protein |
| 75342 | 0.00457 | 1.139 down | 1.415 up | unknown protein |
| 104942 | 0.00487 | 1.272 down | 1.332 up | unknown protein |
| 45369 | 0.00487 | 1.174 up | 1.084 up | unknown protein |
| 58765 | 0.00498 | 1.218 down | 1.462 up | unknown protein |
| 5164 | 0.005 | 1.213 down | 1.107 up | unknown protein |
| 80636 | 0.00507 | 1.326 up | 1.106 up | unknown protein |
| 56141 | 0.00513 | 1.153 up | 1.192 down | unknown protein |
| 68574 | 0.00514 | 1.094 down | 1.286 down | unknown protein |
| 105190 | 0.00518 | 1.228 down | 1.449 up | unknown protein |
| 106155 | 0.0052 | 1.413 up | 1.311 down | unknown protein |
| 69841 | 0.0052 | 1.400 up | 1.107 up | unknown protein |
| 63104 | 0.00522 | 1.413 down | 1.225 up | unknown protein |
| 62702 | 0.00523 | 1.263 up | 1.333 up | unknown protein |
| 81102 | 0.00524 | 1.351 up | 1.052 up | unknown protein |
| 122299 | 0.00531 | 1.325 up | 1.033 down | unknown protein |
| 102677 | 0.00534 | 1.383 up | 1.455 up | unknown protein |
| 102952 | 0.00551 | 1.072 down | 1.470 up | unknown protein |
| 70001 | 0.0056 | 1.117 up | 1.462 down | unknown protein |
| 22365 | 0.00563 | 1.346 down | 1.361 up | unknown protein |
| 58063 | 0.00564 | 1.007 down | 1.266 up | unknown protein |
| 102648 | 0.00567 | 1.421 down | 1.187 up | unknown protein |
| 54157 | 0.00567 | 1.179 up | 1.445 up | unknown protein |
| 2186 | 0.00573 | 1.200 down | 1.298 up | unknown protein |
| 78162 | 0.00593 | 1.232 up | 1.342 up | unknown protein |
| 109007 | 0.00606 | 1.344 up | 1.442 up | unknown protein |
| 80484 | 0.00611 | 1.294 down | 1.232 down | unknown protein |
| 23048 | 0.00616 | 1.431 down | 1.412 up | unknown protein |
| 66222 | 0.00635 | 1.320 up | 1.344 up | unknown protein |
| 123884 | 0.00635 | 1.140 up | 1.206 up | unknown protein |
| 68930 | 0.00638 | 1.406 up | 1.455 up | unknown protein |
| 60887 | 0.00642 | 1.188 down | 1.243 up | unknown protein |
| 2721 | 0.00681 | 1.138 down | 1.061 down | unknown protein |
| 51443 | 0.00685 | 1.096 down | 1.463 up | unknown protein |
| 120059 | 0.00686 | 1.207 up | 1.434 up | unknown protein |
| 59137 | 0.00687 | 1.123 up | 1.070 up | unknown protein |
| 76330 | 0.00693 | 1.257 up | 1.189 up | unknown protein |
| 120384 | 0.00714 | 1.146 up | 1.313 up | unknown protein |
| 82523 | 0.00725 | 1.215 up | 1.424 down | unknown protein |
| 109123 | 0.00726 | 1.010 up | 1.497 up | unknown protein |
| 49795 | 0.00732 | 1.051 up | 1.349 up | unknown protein |
| 81925 | 0.00734 | 1.081 down | 1.495 up | unknown protein |
| 64372 | 0.00737 | 1.369 down | 1.261 up | unknown protein |
| 60737 | 0.00739 | 1.459 down | 1.473 up | unknown protein |
| 53462 | 0.00758 | 1.433 up | 1.162 up | unknown protein |
| 121338 | 0.00759 | 1.327 up | 1.369 up | unknown protein |
| 105381 | 0.00763 | 1.131 down | 1.193 up | unknown protein |
| 108387 | 0.00766 | 1.116 down | 1.489 up | unknown protein |
| 65480 | 0.00767 | 1.363 up | 1.465 up | unknown protein |
| 73912 | 0.00785 | 1.414 down | 1.370 up | unknown protein |
| 110949 | 0.00803 | 1.482 down | 1.481 up | unknown protein |
| 43427 | 0.00809 | 1.297 down | 1.357 up | unknown protein |
| 66218 | 0.00814 | 1.127 down | 1.462 up | unknown protein |
| 121022 | 0.00821 | 1.075 up | 1.035 down | unknown protein |
| 54813 | 0.00835 | 1.498 down | 1.222 up | unknown protein |
| 111644 | 0.0084 | 1.277 up | 1.013 down | unknown protein |
| 109039 | 0.00841 | 1.216 down | 1.207 up | unknown protein |
| 104878 | 0.00846 | 1.082 down | 1.475 down | unknown protein |
| 57855 | 0.00847 | 1.051 up | 1.261 up | unknown protein |
| 77713 | 0.00859 | 1.265 down | 1.260 up | unknown protein |
| 5431 | 0.00861 | 1.465 down | 1.225 up | unknown protein |
| 64963 | 0.00862 | 1.431 down | 1.211 up | unknown protein |
| 49295 | 0.00874 | 1.135 down | 1.449 up | unknown protein |
| 81150 | 0.00879 | 1.086 up | 1.014 up | unknown protein |
| 110524 | 0.0088 | 1.165 down | 1.298 up | unknown protein |
| 76752 | 0.00894 | 1.047 down | 1.156 up | unknown protein |
| 65004 | 0.00916 | 1.102 up | 1.388 up | unknown protein |
| 57474 | 0.00924 | 1.075 down | 1.253 up | unknown protein |
| 120414 | 0.00933 | 1.426 down | 1.120 down | unknown protein |
| 3351 | 0.00957 | 1.458 up | 1.276 up | unknown protein |
| 123987 | 0.00958 | 1.169 up | 1.291 down | unknown protein |
| 75648 | 0.00974 | 1.035 down | 1.492 up | unknown protein |
| 105328 | 0.00984 | 1.128 up | 1.039 down | unknown protein |
| 106646 | 0.0101 | 1.386 down | 1.366 up | unknown protein |
| 104659 | 0.0101 | 1.146 up | 1.435 up | unknown protein |
| 105647 | 0.0101 | 1.116 up | 1.009 down | unknown protein |
| 80073 | 0.0102 | 1.362 up | 1.214 up | unknown protein |
| 107567 | 0.0103 | 1.098 up | 1.415 up | unknown protein |
| 53452 | 0.0104 | 1.400 down | 1.459 up | unknown protein |
| 47766 | 0.0104 | 1.159 down | 1.248 up | unknown protein |
| 110358 | 0.0105 | 1.418 down | 1.325 up | unknown protein |
| 107857 | 0.0105 | 1.203 down | 1.099 up | unknown protein |
| 30275 | 0.0105 | 1.102 up | 1.296 up | unknown protein |
| 80425 | 0.0106 | 1.225 up | 1.443 up | unknown protein |
| 75758 | 0.0107 | 1.183 up | 1.341 up | unknown protein |
| 57581 | 0.0107 | 1.134 down | 1.030 up | unknown protein |
| 61032 | 0.0109 | 1.497 down | 1.109 up | unknown protein |
| 4421 | 0.011 | 1.233 up | 1.494 down | unknown protein |
| 77476 | 0.011 | 1.190 down | 1.241 down | unknown protein |
| 106781 | 0.0112 | 1.225 up | 1.243 up | unknown protein |
| 71077 | 0.0115 | 1.362 up | 1.369 up | unknown protein |
| 110826 | 0.0115 | 1.315 up | 1.483 up | unknown protein |
| 105220 | 0.0115 | 1.037 up | 1.171 up | unknown protein |
| 103498 | 0.0116 | 1.062 down | 1.031 down | unknown protein |
| 23050 | 0.0117 | 1.398 up | 1.480 down | unknown protein |
| 112180 | 0.0117 | 1.033 down | 1.282 down | unknown protein |
| 119887 | 0.0118 | 1.148 down | 1.002 up | unknown protein |
| 109178 | 0.012 | 1.472 down | 1.239 up | unknown protein |
| 121950 | 0.0121 | 1.382 down | 1.486 up | unknown protein |
| 102863 | 0.0122 | 1.493 down | 1.409 down | unknown protein |
| 104807 | 0.0122 | 1.366 down | 1.341 up | unknown protein |
| 54285 | 0.0122 | 1.346 up | 1.061 down | unknown protein |
| 52349 | 0.0123 | 1.352 up | 1.233 up | unknown protein |
| 112602 | 0.0125 | 1.455 down | 1.067 up | unknown protein |
| 81774 | 0.0126 | 1.060 down | 1.308 up | unknown protein |
| 69805 | 0.0127 | 1.306 down | 1.380 up | unknown protein |
| 58848 | 0.0129 | 1.366 down | 1.164 up | unknown protein |
| 61967 | 0.013 | 1.133 up | 1.371 up | unknown protein |
| 124116 | 0.0135 | 1.463 up | 1.175 down | unknown protein |
| 60638 | 0.0135 | 1.426 down | 1.076 up | unknown protein |
| 112651 | 0.0137 | 1.152 down | 1.210 up | unknown protein |
| 75146 | 0.0138 | 1.217 up | 1.291 up | unknown protein |
| 104395 | 0.0139 | 1.248 up | 1.044 down | unknown protein |
| 109359 | 0.0139 | 1.205 up | 1.394 up | unknown protein |
| 62004 | 0.0139 | 1.136 down | 1.326 up | unknown protein |
| 108208 | 0.014 | 1.304 up | 1.235 down | unknown protein |
| 41800 | 0.0141 | 1.311 up | 1.149 down | unknown protein |
| 103937 | 0.0142 | 1.226 down | 1.408 up | unknown protein |
| 63397 | 0.0142 | 1.175 up | 1.408 up | unknown protein |
| 71180 | 0.0142 | 1.145 down | 1.329 up | unknown protein |
| 56896 | 0.0144 | 1.472 up | 1.135 up | unknown protein |
| 80743 | 0.0149 | 1.258 down | 1.459 up | unknown protein |
| 54384 | 0.0151 | 1.306 up | 1.194 up | unknown protein |
| 53785 | 0.0151 | 1.107 down | 1.200 up | unknown protein |
| 108011 | 0.0152 | 1.319 down | 1.477 up | unknown protein |
| 112675 | 0.0152 | 1.221 down | 1.404 up | unknown protein |
| 107260 | 0.0152 | 1.083 down | 1.463 up | unknown protein |
| 124094 | 0.0152 | 1.028 up | 1.219 up | unknown protein |
| 23462 | 0.0154 | 1.228 down | 1.158 up | unknown protein |
| 102641 | 0.0158 | 1.053 down | 1.442 up | unknown protein |
| 66679 | 0.016 | 1.474 up | 1.047 up | unknown protein |
| 59248 | 0.0161 | 1.383 down | 1.078 down | unknown protein |
| 75470 | 0.0161 | 1.005 up | 1.372 up | unknown protein |
| 5894 | 0.0162 | 1.032 down | 1.118 down | unknown protein |
| 33650 | 0.0163 | 1.214 down | 1.492 up | unknown protein |
| 111663 | 0.0167 | 1.004 down | 1.309 up | unknown protein |
| 119739 | 0.0168 | 1.355 up | 1.285 up | unknown protein |
| 57217 | 0.017 | 1.044 down | 1.307 up | unknown protein |
| 111272 | 0.0171 | 1.439 down | 1.168 up | unknown protein |
| 55401 | 0.0171 | 1.050 down | 1.107 up | unknown protein |
| 62975 | 0.0173 | 1.372 down | 1.371 down | unknown protein |
| 79461 | 0.0173 | 1.186 up | 1.338 up | unknown protein |
| 120391 | 0.0174 | 1.382 down | 1.053 up | unknown protein |
| 122689 | 0.0175 | 1.134 down | 1.193 up | unknown protein |
| 110660 | 0.0176 | 1.373 down | 1.031 down | unknown protein |
| 66437 | 0.0176 | 1.268 down | 1.346 down | unknown protein |
| 108899 | 0.0181 | 1.295 up | 1.280 up | unknown protein |
| 57869 | 0.0184 | 1.372 down | 1.344 up | unknown protein |
| 61965 | 0.0185 | 1.050 down | 1.118 up | unknown protein |
| 74491 | 0.0187 | 1.397 up | 1.046 up | unknown protein |
| 4847 | 0.0187 | 1.285 up | 1.347 up | unknown protein |
| 57923 | 0.0188 | 1.249 up | 1.229 up | unknown protein |
| 57277 | 0.0189 | 1.178 up | 1.003 down | unknown protein |
| 55183 | 0.0191 | 1.104 up | 1.292 up | unknown protein |
| 66165 | 0.0191 | 1.062 up | 1.444 up | unknown protein |
| 106353 | 0.0191 | 1.005 down | 1.467 up | unknown protein |
| 21564 | 0.0193 | 1.303 down | 1.120 down | unknown protein |
| 103031 | 0.0195 | 1.495 down | 1.394 down | unknown protein |
| 22143 | 0.0195 | 1.186 down | 1.120 up | unknown protein |
| 78811 | 0.0196 | 1.046 down | 1.015 up | unknown protein |
| 110593 | 0.0199 | 1.388 down | 1.304 down | unknown protein |
| 122113 | 0.02 | 1.141 down | 1.355 up | unknown protein |
| 54722 | 0.0202 | 1.097 down | 1.112 up | unknown protein |
| 106178 | 0.0203 | 1.398 up | 1.195 down | unknown protein |
| 63156 | 0.0203 | 1.159 down | 1.177 up | unknown protein |
| 44088 | 0.0204 | 1.218 down | 1.288 up | unknown protein |
| 48058 | 0.0204 | 1.165 up | 1.164 up | unknown protein |
| 29713 | 0.0208 | 1.043 up | 1.305 up | unknown protein |
| 53812 | 0.0209 | 1.321 down | 1.285 up | unknown protein |
| 43884 | 0.021 | 1.182 down | 1.191 up | unknown protein |
| 108287 | 0.021 | 1.050 down | 1.339 up | unknown protein |
| 81616 | 0.0211 | 1.188 up | 1.493 up | unknown protein |
| 54244 | 0.0221 | 1.323 down | 1.497 up | unknown protein |
| 22496 | 0.0222 | 1.217 down | 1.185 up | unknown protein |
| 36576 | 0.0222 | 1.208 down | 1.036 down | unknown protein |
| 68274 | 0.0222 | 1.202 down | 1.252 up | unknown protein |
| 3891 | 0.0223 | 1.252 up | 1.430 up | unknown protein |
| 64937 | 0.0224 | 1.083 up | 1.445 up | unknown protein |
| 121126 | 0.0228 | 1.077 down | 1.189 up | unknown protein |
| 76340 | 0.0231 | 1.224 down | 1.464 down | unknown protein |
| 112131 | 0.0235 | 1.231 up | 1.475 up | unknown protein |
| 42915 | 0.0237 | 1.152 down | 1.095 up | unknown protein |
| 64347 | 0.0238 | 1.107 down | 1.188 up | unknown protein |
| 54611 | 0.024 | 1.360 down | 1.430 up | unknown protein |
| 62017 | 0.0241 | 1.302 down | 1.423 up | unknown protein |
| 2348 | 0.0241 | 1.274 down | 1.075 down | unknown protein |
| 45748 | 0.0255 | 1.280 down | 1.216 up | unknown protein |
| 21278 | 0.0257 | 1.364 up | 1.050 up | unknown protein |
| 59598 | 0.0258 | 1.003 down | 1.113 up | unknown protein |
| 108421 | 0.0265 | 1.429 up | 1.391 up | unknown protein |
| 102377 | 0.0266 | 1.309 up | 1.189 up | unknown protein |
| 74163 | 0.0268 | 1.131 down | 1.272 up | unknown protein |
| 61858 | 0.0273 | 1.038 up | 1.449 up | unknown protein |
| 53443 | 0.0276 | 1.160 up | 1.278 up | unknown protein |
| 109590 | 0.0277 | 1.214 down | 1.342 up | unknown protein |
| 63744 | 0.028 | 1.204 down | 1.046 up | unknown protein |
| 23111 | 0.028 | 1.129 down | 1.044 down | unknown protein |
| 2599 | 0.028 | 1.051 up | 1.274 up | unknown protein |
| 120418 | 0.0282 | 1.038 up | 1.218 down | unknown protein |
| 77736 | 0.0283 | 1.352 up | 1.326 up | unknown protein |
| 104239 | 0.0283 | 1.204 up | 1.179 down | unknown protein |
| 42942 | 0.0287 | 1.275 down | 1.498 up | unknown protein |
| 123663 | 0.0287 | 1.034 up | 1.246 up | unknown protein |
| 55252 | 0.0289 | 1.267 up | 1.048 down | unknown protein |
| 122384 | 0.0293 | 1.372 down | 1.208 up | unknown protein |
| 53395 | 0.0296 | 1.087 down | 1.350 down | unknown protein |
| 32747 | 0.0297 | 1.020 down | 1.055 up | unknown protein |
| 54961 | 0.0299 | 1.188 down | 1.046 up | unknown protein |
| 111035 | 0.03 | 1.410 down | 1.057 up | unknown protein |
| 46242 | 0.03 | 1.377 up | 1.118 down | unknown protein |
| 78421 | 0.03 | 1.128 down | 1.269 up | unknown protein |
| 21412 | 0.0301 | 1.446 down | 1.490 down | unknown protein |
| 75012 | 0.0301 | 1.059 down | 1.395 up | unknown protein |
| 50536 | 0.0305 | 1.453 down | 1.016 down | unknown protein |
| 64989 | 0.0305 | 1.059 down | 1.385 up | unknown protein |
| 74624 | 0.0308 | 1.172 down | 1.219 up | unknown protein |
| 62323 | 0.0308 | 1.023 up | 1.026 up | unknown protein |
| 108235 | 0.0311 | 1.306 up | 1.026 up | unknown protein |
| 64308 | 0.0314 | 1.086 up | 1.353 up | unknown protein |
| 111250 | 0.0315 | 1.470 up | 1.010 up | unknown protein |
| 65603 | 0.0318 | 1.216 down | 1.107 up | unknown protein |
| 76250 | 0.032 | 1.321 down | 1.271 up | unknown protein |
| 119694 | 0.0326 | 1.323 up | 1.316 up | unknown protein |
| 60426 | 0.0327 | 1.065 down | 1.405 up | unknown protein |
| 73815 | 0.0328 | 1.055 up | 1.266 up | unknown protein |
| 53495 | 0.0331 | 1.123 down | 1.083 up | unknown protein |
| 108211 | 0.0332 | 1.247 down | 1.172 down | unknown protein |
| 4154 | 0.0333 | 1.337 down | 1.426 up | unknown protein |
| 49304 | 0.0337 | 1.224 down | 1.249 up | unknown protein |
| 33811 | 0.034 | 1.454 down | 1.121 up | unknown protein |
| 107072 | 0.034 | 1.072 down | 1.170 down | unknown protein |
| 112685 | 0.0347 | 1.341 down | 1.439 up | unknown protein |
| 121004 | 0.0348 | 1.247 down | 1.372 up | unknown protein |
| 104016 | 0.0355 | 1.369 up | 1.082 up | unknown protein |
| 123740 | 0.0357 | 1.278 up | 1.062 down | unknown protein |
| 22221 | 0.0364 | 1.081 down | 1.256 up | unknown protein |
| 106755 | 0.037 | 1.439 up | 1.023 up | unknown protein |
| 120160 | 0.0381 | 1.399 down | 1.008 down | unknown protein |
| 104370 | 0.0381 | 1.212 up | 1.182 up | unknown protein |
| 122381 | 0.0389 | 1.472 down | 1.494 up | unknown protein |
| 107132 | 0.039 | 1.327 down | 1.072 up | unknown protein |
| 49517 | 0.0393 | 1.274 up | 1.334 up | unknown protein |
| 119638 | 0.0394 | 1.048 down | 1.041 down | unknown protein |
| 74476 | 0.0394 | 1.001 up | 1.472 down | unknown protein |
| 54393 | 0.0396 | 1.304 up | 1.112 up | unknown protein |
| 80990 | 0.0397 | 1.300 up | 1.204 down | unknown protein |
| 21135 | 0.0398 | 1.287 up | 1.353 up | unknown protein |
| 109242 | 0.0401 | 1.082 up | 1.158 down | unknown protein |
| 108333 | 0.0409 | 1.003 up | 1.039 up | unknown protein |
| 120294 | 0.041 | 1.020 down | 1.160 up | unknown protein |
| 32204 | 0.0416 | 1.027 up | 1.071 down | unknown protein |
| 68154 | 0.0417 | 1.348 down | 1.025 down | unknown protein |
| 56864 | 0.0421 | 1.179 up | 1.358 up | unknown protein |
| 111306 | 0.0422 | 1.273 up | 1.389 down | unknown protein |
| 63328 | 0.0424 | 1.271 up | 1.297 up | unknown protein |
| 120892 | 0.0428 | 1.458 down | 1.118 down | unknown protein |
| 68647 | 0.0428 | 1.205 down | 1.215 up | unknown protein |
| 81405 | 0.0434 | 1.339 up | 1.241 up | unknown protein |
| 60729 | 0.0443 | 1.423 down | 1.054 down | unknown protein |
| 66163 | 0.0455 | 1.290 down | 1.197 up | unknown protein |
| 79464 | 0.0457 | 1.195 down | 1.008 up | unknown protein |
| 102890 | 0.0459 | 1.111 down | 1.192 down | unknown protein |
| 27020 | 0.0469 | 1.206 up | 1.018 down | unknown protein |
| 80365 | 0.0469 | 1.198 up | 1.261 up | unknown protein |
| 79817 | 0.047 | 1.298 down | 1.183 up | unknown protein |
| 4936 | 0.0476 | 1.273 up | 1.430 up | unknown protein |
| 5013 | 0.0479 | 1.241 down | 1.316 down | unknown protein |
| 66280 | 0.0483 | 1.060 up | 1.283 up | unknown protein |
| 54535 | 0.0483 | 1.034 up | 1.435 down | unknown protein |
| 30274 | 0.0484 | 1.415 down | 1.373 up | unknown protein |
| 54198 | 0.0495 | 1.133 up | 1.017 up | unknown protein |
| 110281 | 0.0495 | 1.061 down | 1.068 up | unknown protein |
| 105537 | 0.0498 | 1.409 up | 1.290 up | unknown protein |
| 108950 | 0.0498 | 1.294 down | 1.283 up | unknown protein |
| 60879 | 0.00539 | 1.158 up | 1.214 down | unknown protein |
| 120610 | 0.00967 | 1.181 down | 1.209 down | Unknown protein |
| 60149 | 0.0088 | 1.015 up | 1.270 up | unknown protein DUF227 |
| 79075 | 0.0464 | 1.169 up | 1.055 down | unknown protein of ORM1 family |
| 4244 | 0.0346 | 1.050 up | 1.098 down | unknown protein with DEAD/DEAH box helicase domain |
| 2529 | 0.00258 | 1.405 down | 1.061 up | unknown protein |
| 51197 | 0.0446 | 1.120 up | 1.178 up | unknown protein with CCHC finger |
| 109088 | 0.00346 | 1.168 up | 1.386 up | unknown protein with chromo domain |
| 78953 | 0.00235 | 1.267 up | 1.462 down | unknown protein with CORD and CS domain |
| 62213 | 0.00362 | 1.240 up | 1.058 up | unknown protein with fasciclin domain |
| 58541 | 0.00257 | 1.030 down | 1.243 up | unknown protein with Kelch repeats |
| 110293 | 0.0058 | 1.381 down | 1.176 up | unknown protein with NUDIX domain (hydrolase) |
| 67882 | 0.0118 | 1.385 down | 1.129 up | unknown protein with NUDIX domain (hydrolase) |
| 3422 | 0.00762 | 1.428 down | 1.492 up | unknown protein with oxidoreductase domain |
| 3434 | 0.000299 | 1.440 down | 1.011 down | unknown protein with patatin domain |
| 79686 | 0.0224 | 1.235 up | 1.406 up | Unknown protein with RNA binding domains |
| 104785 | 0.00344 | 1.071 down | 1.445 up | unknown protein with SacI domain |
| 108332 | 0.0119 | 1.321 up | 1.417 down | unknown protein with SDA1 domain |
| 124030 | 0.0128 | 1.063 up | 1.138 up | unknown protein with TIM barrel |
| 110396 | 0.0125 | 1.323 down | 1.389 up | unknown protein with Tim10/DDP family zinc finger |
| 63173 | 0.00142 | 1.207 down | 1.475 up | unknown protein with YIP1 domain |
| 111022 | 0.000683 | 1.449 down | 1.438 up | Unknown protein with zinc finger (PHD) domain. |
| 67958 | 0.0202 | 1.289 up | 1.442 up | unknown protein, only present in Magnaporthe, Chaetomium and Gibberella |
| 110150 | 0.0209 | 1.494 down | 1.264 down | unknown protein, 1 TM |
| 106576 | 0.00901 | 1.035 up | 1.086 up | unknown protein, 2 TM |
| 121172 | 0.00362 | 1.286 down | 1.360 up | unknown protein, 2 TM, related to N. crassa NADH-ubiquinone oxidoreductase |
| 73519 | 0.000395 | 1.061 down | 1.486 up | unknown protein, 4 TM |
| 80187 | 0.00593 | 1.423 down | 1.325 up | unknown protein, 8TM (auxin efflux carrier) |
| 50996 | 0.00047 | 1.219 up | 1.204 down | unknown protein, C2 domain |
| 65033 | 0.0127 | 1.370 up | 1.102 down | unknown protein, secreted |
| 124282 | 0.00116 | 1.319 down | 1.251 up | unknown protein, SET and MYND domain |
| 109835 | 0.0187 | 1.133 down | 1.440 down | unknown protein, thaumatin family |
| 57632 | 0.00296 | 1.020 up | 1.380 down | unknown protein, WD repeats |
| 79816 | 0.0397 | 1.326 up | 1.144 down | unknown protein; secreted |
| 4170 | 0.00235 | 1.048 down | 1.196 up | unknown proteiun with WD repeats |
| 53187 | 0.00983 | 1.034 up | 1.070 up | UreD urease accessory protein |
| 67840 | 0.00596 | 1.082 down | 1.296 down | UTP11, encoding a component of the SSU processome |
| 49373 | 0.00147 | 1.498 down | 1.239 up | UTP15, encoding a component of the SSU processome |
| 112556 | 0.00174 | 1.023 down | 1.375 down | UTP5, encoding a component of the SSU processome |
| 75024 | 0.000955 | 1.237 up | 1.478 up | vacuolar ATP synthase 98 kDa subunit |
| 76057 | 0.00695 | 1.249 down | 1.379 up | Vacuolar ATP synthase subunit B |
| 78054 | 0.0395 | 1.018 down | 1.208 up | Vacuolar protein sorting-associated protein Vps16 |
| 76515 | 0.0368 | 1.177 down | 1.338 up | Vacuolar protein sorting-associated protein Vps33 |
| 121169 | 0.0172 | 1.077 down | 1.181 up | Vacuolar protein sorting-associated protein Vps46 |
| 70251 | 0.017 | 1.113 up | 1.076 down | vacuolar protein-sorting machinery class E protein HSE1 |
| 112516 | 0.000762 | 1.169 up | 1.139 up | Vacuolar sorting protein VPS1, dynamin |
| 51734 | 0.0366 | 1.313 up | 1.351 up | Vesicle coat complex COPI, beta subunit |
| 52208 | 0.0246 | 1.120 down | 1.180 up | Vesicle coat complex COPII, Sec23 |
| 60626 | 0.00745 | 1.402 down | 1.112 up | Vesicle coat complex COPII, Sec24 |
| 67473 | 0.00957 | 1.357 down | 1.386 up | v-SNARE Vti1; cis-Golgi membrane traffic; vacuolar sorting pathways |
| 57465 | 0.0306 | 1.187 down | 1.188 down | Vsp9 domain protein |
| 78688 | 0.00913 | 1.462 up | 1.052 up | Winged helix repressor DNA-binding |
| 23316 | 0.0288 | 1.064 down | 1.000 down | Yip1 interacting protein Yop1 |
| 121602 | 0.000938 | 1.229 up | 1.341 down | Zn2Cys6 transcriptional regulator |
| 68254 | 0.00159 | 1.075 down | 1.423 down | Zn2Cys6 transcriptional regulator |
| 48281 | 0.00353 | 1.076 up | 1.399 up | Zn2Cys6 transcriptional regulator |
| 66828 | 0.00547 | 1.220 up | 1.274 up | Zn2Cys6 transcriptional regulator |
| 54567 | 0.00618 | 1.219 down | 1.476 up | Zn2Cys6 transcriptional regulator |
| 73559 | 0.00916 | 1.366 down | 1.231 up | Zn2Cys6 transcriptional regulator |
| 110689 | 0.0104 | 1.207 up | 1.255 down | Zn2Cys6 transcriptional regulator |
| 121130 | 0.0108 | 1.292 down | 1.045 up | Zn2Cys6 transcriptional regulator |
| 57534 | 0.0145 | 1.473 down | 1.067 down | Zn2Cys6 transcriptional regulator |
| 59546 | 0.0165 | 1.172 up | 1.210 up | Zn2Cys6 transcriptional regulator |
| 48438 | 0.019 | 1.436 down | 1.405 down | Zn2Cys6 transcriptional regulator |
| 59760 | 0.0202 | 1.068 up | 1.444 up | Zn2Cys6 transcriptional regulator |
| 104182 | 0.0243 | 1.137 up | 1.278 down | Zn2Cys6 transcriptional regulator |
| 123881 | 0.0381 | 1.486 down | 1.232 up | Zn2Cys6 transcriptional regulator |
| 120877 | 0.001 | 1.372 down | 1.237 up | Zn-dependent ß-lactamase |
