# Supplementary material for: Comparative analysis of the Trichoderma reesei transcriptome during growth on the cellulase inducing substrates wheat straw and lactose
Source: Biotechnol Biofuels. 2013 Sep 9;6:127. doi: 10.1186/1754-6834-6-127 (PMC3847502; doi:10.1186/1754-6834-6-127)
Supplement: Additional file 2: Table S2 — All genes that are at least 2-fold differentially regulated on lactose or wheat straw vs glucose. [file 1754-6834-6-127-S2.docx]

| **Supplementary Table S2**. All genes that are at least 2-fold differentially regulated on lactose or wheat straw vs glucose | | | | | | | | | | | | |
| --- | --- | --- | --- | --- | --- | --- | --- | --- | --- | --- | --- | --- |
|  |  |  |  | |  | |  |  | |  | |  |
|  |  | *log2 expression* | | | | | *ratio* | | | | |  |
| **SEQ_ID** | **P value** | **lactose** | | **cellulose** | | **glucose** | **Glc_Lac** | | **Glc_Cell** | | **Lac_Cell** |  |
| 107172 | 0.000224 | 6.89039 | | 8.38027 | | 6.10936 | 1.718 up | | 4.826 up | | 2.808 up | 2,2-dialkylglycine decarboxylase, AIB forming ? |
| 60490 | 0.000442 | 10.90491 | | 12.21485 | | 9.07817 | 3.547 up | | 8.795 up | | 2.479 up | 2-nitropropane dioxygenase |
| 3506 | 0.000352 | 10.95287 | | 10.64448 | | 9.19131 | 3.390 up | | 2.738 up | | 1.238 down | 2OG-Fe(II) oxygenase superfamily protein |
| 65029 | 0.00114 | 12.93892 | | 11.68027 | | 11.12432 | 3.517 up | | 1.470 up | | 2.392 down | 2OG-Fe(II) oxygenase superfamily protein |
| 102957 | 0.000322 | 8.15788 | | 9.15016 | | 6.29877 | 3.627 up | | 7.216 up | | 1.989 up | 2-oxoglutarate-dependent ethylene/succinate-forming enzyme, putative |
| 122745 | 0.000112 | 8.88514 | | 11.92295 | | 10.59247 | 3.265 down | | 2.514 up | | 8.212 up | 2-oxoisovalerate dehydrogenase subunit beta, putative |
| 52956 | 0.00335 | 11.42128 | | 12.67263 | | 10.98149 | 1.356 up | | 3.229 up | | 2.380 up | 3'-5' exonuclease |
| 57647 | 0.000289 | 9.87714 | | 10.74827 | | 7.76137 | 4.334 up | | 7.927 up | | 1.829 up | 3-beta hydroxysteroid dehydrogenase/isomerase, putative |
| 104140 | 0.000436 | 9.55121 | | 11.30706 | | 8.06462 | 2.802 up | | 9.463 up | | 3.377 up | 3-hydroxi-isobutyrate dehydrogenase |
| 106649 | 0.00797 | 5.62902 | | 5.20835 | | 4.27013 | 2.564 up | | 1.916 up | | 1.338 down | 3-hydroxyacyl-CoA dehydrogenase, NAD binding domain, putative |
| 112247 | 0.00021 | 11.69401 | | 9.81247 | | 6.38284 | 39.702 up | | 10.775 up | | 3.684 down | 3-isopropylmalate dehydrogenase |
| 3835 | 0.0157 | 10.40075 | | 11.03511 | | 9.31225 | 2.126 up | | 3.300 up | | 1.552 up | 3-ketoacyl-CoA thiolase |
| 123720 | 0.00243 | 12.56587 | | 12.81805 | | 11.20176 | 2.574 up | | 3.065 up | | 1.191 up | 3-ketoacyl-CoA thiolase-like protein |
| 50159 | 0.000424 | 8.77749 | | 10.96681 | | 9.62526 | 1.799 down | | 2.534 up | | 4.560 up | 3-methyl-2-oxobutanoate hydroxymethyltransferase |
| 64345 | 0.00108 | 7.01399 | | 10.11328 | | 8.11033 | 2.138 down | | 4.008 up | | 8.569 up | 3-Methylcrotonyl-CoA carboxylase, non-biotin containing |
| 82177 | 0.00436 | 9.15392 | | 8.95173 | | 7.76032 | 2.627 up | | 2.283 up | | 1.150 down | 3-oxo-5-alpha-steroid 4-dehydrogenase |
| 112521 | 0.000532 | 8.07809 | | 9.07 | | 5.75927 | 4.989 up | | 9.922 up | | 1.988 up | 4,5-dihydroxyphthalate decarboxylase |
| 121405 | 0.000145 | 12.11009 | | 9.85313 | | 7.67067 | 21.697 up | | 4.539 up | | 4.779 down | 4-aminobutyrate aminotransferase |
| 122110 | 0.00115 | 8.28182 | | 9.34831 | | 7.25743 | 2.034 up | | 4.260 up | | 2.094 up | 4-coumarate:coenzyme A ligase |
| 121856 | 0.000143 | 12.17727 | | 13.49089 | | 8.99335 | 9.087 up | | 22.588 up | | 2.485 up | 4-hydroxyphenylpyruvate dioxygenase |
| 50593 | 0.000228 | 10.16453 | | 13.57526 | | 8.59887 | 2.960 up | | 31.480 up | | 10.634 up | 5' nucleotidase |
| 50390 | 0.000905 | 9.74941 | | 12.44735 | | 11.06216 | 2.484 down | | 2.612 up | | 6.488 up | 5' nucleotidase |
| 39996 | 0.000545 | 6.25807 | | 8.61868 | | 6.50404 | 1.185 down | | 4.330 up | | 5.135 up | 6-phosphogluconolactonase, putative (check!) |
| 121942 | 0.00131 | 12.16451 | | 12.90585 | | 11.4876 | 1.598 up | | 2.672 up | | 1.671 up | Aa_transTransmembrane amino acid transporter protein |
| 49888 | 0.00712 | 12.09797 | | 11.51927 | | 10.47889 | 3.071 up | | 2.056 up | | 1.493 down | AAA ATPase |
| 104683 | 0.00111 | 9.70455 | | 11.853 | | 10.28561 | 1.495 down | | 2.963 up | | 4.433 up | AAA ATPase |
| 35556 | 0.000808 | 9.08704 | | 11.06962 | | 8.97614 | 1.079 up | | 4.267 up | | 3.951 up | AAA ATPase |
| 66132 | 0.0024 | 10.02369 | | 11.90375 | | 9.94274 | 1.057 up | | 3.893 up | | 3.680 up | AAA ATPase |
| 73099 | 0.000811 | 11.43642 | | 11.96746 | | 10.44076 | 1.993 up | | 2.881 up | | 1.444 up | AAA ATPase, DNA repair and recombination protein PIF1 (ATP dependent helicase) |
| 60018 | 0.000338 | 7.12473 | | 7.66798 | | 5.26045 | 3.640 up | | 5.305 up | | 1.457 up | AAA+ ATPase |
| 64710 | 0.000598 | 10.97706 | | 11.81517 | | 10.40457 | 1.487 up | | 2.658 up | | 1.787 up | AAA+-type ATPase |
| 74187 | 0.000336 | 6.94308 | | 9.57358 | | 7.39025 | 1.363 down | | 4.541 up | | 6.192 up | AAA+-type ATPase |
| 75247 | 0.00461 | 4.67208 | | 6.40489 | | 4.89681 | 1.168 down | | 2.844 up | | 3.323 up | AAA+-type ATPase |
| 55747 | 0.000476 | 10.57154 | | 12.62743 | | 11.29691 | 1.653 down | | 2.514 up | | 4.158 up | ABC transporter |
| 62693 | 0.000132 | 12.91074 | | 10.92549 | | 9.44323 | 11.061 up | | 2.793 up | | 3.959 down | ABC-transporter Ste6p |
| 5107 | 0.000306 | 12.81364 | | 9.39391 | | 8.60798 | 18.451 up | | 1.724 up | | 10.701 down | ACC deaminase [Trichoderma asperellum] |
| 31658 | 0.000133 | 9.00337 | | 11.86938 | | 6.93337 | 4.198 up | | 30.611 up | | 7.290 up | acetamidase |
| 103041 | 0.000617 | 5.09754 | | 10.6024 | | 4.67387 | 1.341 up | | 60.906 up | | 45.407 up | acetamidase |
| 54870 | 0.00053 | 12.56778 | | 12.72659 | | 10.36909 | 4.590 up | | 5.124 up | | 1.116 up | acetate—CoA ligase |
| 65921 | 0.00125 | 7.81316 | | 11.41451 | | 9.4516 | 3.113 down | | 3.898 up | | 12.137 up | Acetyl/propionyl-CoA carboxylase alpha subunit |
| 77093 | 0.000131 | 13.02806 | | 13.33856 | | 8.54347 | 22.386 up | | 27.762 up | | 1.240 up | acid sphingomyelin phosphodiesterase (probably vacuolar) |
| 77541 | 0.000741 | 8.86404 | | 10.83335 | | 9.3471 | 1.397 down | | 2.801 up | | 3.915 up | actin-like protein |
| 82403 | 0.00107 | 13.54541 | | 13.25194 | | 12.04574 | 2.827 up | | 2.307 up | | 1.225 down | Acyl-CoA dehydrogenase |
| 121629 | 0.0104 | 13.61265 | | 13.33914 | | 11.66597 | 3.854 up | | 3.189 up | | 1.208 down | acyl-CoA dehydrogenase |
| 106885 | 0.00132 | 7.71057 | | 9.35856 | | 7.42617 | 1.217 up | | 3.816 up | | 3.133 up | acyl-CoA dehydrogenase |
| 3464 | 0.000194 | 10.04257 | | 10.76833 | | 7.9887 | 4.152 up | | 6.866 up | | 1.653 up | acyl-CoA dehydrogenase, putative |
| 107947 | 0.000821 | 12.36114 | | 10.67715 | | 8.57407 | 13.804 up | | 4.296 up | | 3.213 down | Acyl-CoA synthetase |
| 54667 | 0.000101 | 9.6744 | | 13.55914 | | 3.85117 | 56.619 up | | 836.350 up | | 14.771 up | acyl-CoA synthetase |
| 55306 | 0.000426 | 6.72549 | | 8.44682 | | 6.30369 | 1.339 up | | 4.417 up | | 3.297 up | Acyl-CoA synthetase |
| 119875 | 0.00251 | 8.12619 | | 9.91422 | | 8.19387 | 1.048 down | | 3.295 up | | 3.453 up | acyl-CoA thioesterase |
| 53372 | 0.00242 | 11.2606 | | 11.35608 | | 9.6452 | 3.063 up | | 3.273 up | | 1.068 up | acyltransferase 3 |
| 69753 | 0.000145 | 11.45091 | | 12.78166 | | 8.40011 | 8.286 up | | 20.843 up | | 2.515 up | ADA, Adenosine deaminase |
| 66689 | 0.0036 | 5.6146 | | 7.41222 | | 4.70195 | 1.882 up | | 6.544 up | | 3.476 up | Adenine deaminase/adenosine deaminase |
| 68522 | 0.00687 | 11.76506 | | 12.23508 | | 10.9003 | 1.821 up | | 2.522 up | | 1.385 up | Adenosine deaminase-related growth factors |
| 59053 | 0.000662 | 12.54008 | | 10.67864 | | 10.76989 | 3.411 up | | 1.065 down | | 3.633 down | Adenosine/AMP deaminase |
| 81260 | 0.00144 | 11.9722 | | 12.39046 | | 10.52878 | 2.719 up | | 3.634 up | | 1.336 up | adenylate kinase |
| 58412 | 0.00726 | 7.15009 | | 7.57397 | | 6.14215 | 2.011 up | | 2.697 up | | 1.341 up | AdhP Zn-dependent alcohol dehydrogenases |
| 104106 | 0.00363 | 4.15554 | | 5.77393 | | 4.22765 | 1.051 down | | 2.920 up | | 3.070 up | ADP/ATP carrier protein |
| 107255 | 0.00456 | 10.56097 | | 11.77275 | | 10.01948 | 1.455 up | | 3.371 up | | 2.316 up | ADP-ribosylation factor-like protein 2, Ras small GTPase |
| 110709 | 0.000247 | 6.75409 | | 9.86228 | | 6.50745 | 1.186 up | | 10.230 up | | 8.623 up | ADP-ribosylglycohydrolase |
| 102998 | 0.000826 | 9.73845 | | 11.21774 | | 9.63309 | 1.075 up | | 2.999 up | | 2.788 up | ADP-ribosylglycohydrolase-like protein |
| 120696 | 0.00197 | 9.50121 | | 9.91837 | | 7.74972 | 3.367 up | | 4.496 up | | 1.335 up | alcohol dehydrogenase |
| 3333 | 0.000276 | 8.18152 | | 10.85415 | | 8.70403 | 1.436 down | | 4.438 up | | 6.375 up | Alcohol dehydrogenase zinc-binding domain protein |
| 2038 | 0.000742 | 7.26049 | | 7.99455 | | 4.79456 | 5.524 up | | 9.189 up | | 1.663 up | Alcohol dehydrogenase, class IV |
| 68590 | 0.0000999 | 10.65271 | | 11.25748 | | 6.78088 | 14.639 up | | 22.263 up | | 1.520 up | Alcohol dehydrogenase, class V |
| 80659 | 0.0000994 | 11.37824 | | 14.21387 | | 5.21616 | 71.609 up | | 511.189 up | | 7.138 up | alcohol oxidase AOX1 |
| 54525 | 0.00304 | 9.73252 | | 11.78883 | | 10.32708 | 1.510 down | | 2.754 up | | 4.159 up | AldedhAldehyde dehydrogenase family |
| 123274 | 0.00025 | 12.84837 | | 13.06651 | | 10.21094 | 6.222 up | | 7.237 up | | 1.163 up | Aldehyde dehydrogenase |
| 60418 | 0.00265 | 4.80541 | | 5.34572 | | 3.644 | 2.236 up | | 3.252 up | | 1.454 up | Aldehyde dehydrogenase |
| 105363 | 0.000263 | 11.53469 | | 9.152 | | 8.88284 | 6.284 up | | 1.205 up | | 5.215 down | aldolase/citrate lyase family protein |
| 121661 | 6.60E-06 | 13.51561 | | 13.98734 | | 12.31113 | 2.304 up | | 3.195 up | | 1.386 up | aldose-1-epimerase |
| 81843 | 0.000341 | 10.79803 | | 9.15848 | | 7.61646 | 9.072 up | | 2.912 up | | 3.115 down | Alkaline phosphatase |
| 105504 | 0.000464 | 10.23743 | | 12.89292 | | 10.28866 | 1.036 down | | 6.080 up | | 6.300 up | Alkaline phosphatase, putative |
| 81022 | 0.0032 | 13.0084 | | 12.46209 | | 10.80402 | 4.608 up | | 3.155 up | | 1.460 down | allantoate permease, 10 TM domains |
| 70860 | 0.00013 | 12.18185 | | 13.21959 | | 10.03226 | 4.437 up | | 9.109 up | | 2.053 up | allantoate permease, 10 TM domains |
| 32243 | 0.00016 | 11.55467 | | 12.33319 | | 8.78784 | 6.806 up | | 11.675 up | | 1.715 up | Alpha/beta hydrolase |
| 62576 | 0.000211 | 11.73232 | | 13.78984 | | 7.33319 | 21.099 up | | 87.830 up | | 4.162 up | alpha/beta hydrolase |
| 58783 | 0.00252 | 12.20578 | | 12.95982 | | 11.50781 | 1.622 up | | 2.735 up | | 1.686 up | alpha/beta hydrolase |
| 80778 | 0.000232 | 8.04195 | | 10.48458 | | 7.57261 | 1.384 up | | 7.526 up | | 5.436 up | alpha/beta hydrolase |
| 66324 | 0.00066 | 10.90564 | | 12.42528 | | 10.84067 | 1.046 up | | 2.999 up | | 2.867 up | alpha/beta hydrolase |
| 63202 | 0.00191 | 11.98786 | | 11.31494 | | 10.38203 | 3.043 up | | 1.909 up | | 1.594 down | alpha/beta hydrolase, |
| 67408 | 0.000146 | 11.26639 | | 12.81807 | | 9.40289 | 3.638 up | | 10.667 up | | 2.931 up | AMA1 which is an activator of meiotic anaphase promoting complex |
| 5889 | 0.0124 | 10.36886 | | 10.22993 | | 8.80831 | 2.949 up | | 2.678 up | | 1.101 down | amidase |
| 68615 | 0.000641 | 10.52447 | | 10.79262 | | 7.18978 | 10.088 up | | 12.149 up | | 1.204 up | Amidase |
| 70375 | 0.000626 | 11.87105 | | 11.13927 | | 8.70764 | 8.959 up | | 5.395 up | | 1.660 down | amidase |
| 80645 | 0.0000723 | 12.85648 | | 13.67648 | | 10.79185 | 4.183 up | | 7.385 up | | 1.765 up | Amidase |
| 108885 | 0.00254 | 12.48388 | | 11.94893 | | 10.83123 | 3.144 up | | 2.170 up | | 1.448 down | amidase |
| 27697 | 0.000537 | 8.96064 | | 8.1282 | | 7.48755 | 2.776 up | | 1.559 up | | 1.780 down | Amidase |
| 48792 | 0.00128 | 11.0177 | | 12.5422 | | 10.87138 | 1.106 up | | 3.183 up | | 2.876 up | amidase |
| 81070 | 0.00229 | 12.28767 | | 13.80445 | | 12.31514 | 1.019 down | | 2.807 up | | 2.861 up | amidase |
| 58885 | 0.000208 | 9.61867 | | 11.24001 | | 9.81542 | 1.146 down | | 2.684 up | | 3.076 up | Amidases |
| 59119 | 0.000511 | 5.70118 | | 7.51816 | | 4.49951 | 2.300 up | | 8.104 up | | 3.523 up | amidohydrolase family protein |
| 75430 | 0.00111 | 10.13119 | | 11.19391 | | 9.52424 | 1.523 up | | 3.181 up | | 2.088 up | Amino acid permease |
| 70098 | 0.00027 | 11.16963 | | 9.12455 | | 4.54131 | 98.929 up | | 23.971 up | | 4.126 down | amino acid permease (GABA) |
| 58511 | 0.000456 | 12.33868 | | 11.07641 | | 10.35186 | 3.963 up | | 1.652 up | | 2.398 down | amino acid permease (GABA) |
| 62172 | 0.00101 | 9.22182 | | 10.39915 | | 7.22282 | 3.997 up | | 9.040 up | | 2.261 up | amino acid permease (PotE?) |
| 23415 | 0.00111 | 12.68922 | | 13.13399 | | 10.89684 | 3.463 up | | 4.714 up | | 1.361 up | amino acid permease Dip5 |
| 61114 | 0.00112 | 9.98447 | | 10.0411 | | 7.62297 | 5.139 up | | 5.344 up | | 1.040 up | Amino acid transporter LysP |
| 66819 | 0.000133 | 13.16391 | | 12.62581 | | 4.37207 | 443.207 up | | 305.227 up | | 1.452 down | Amino acid transporter LysP |
| 110813 | 0.000287 | 11.60715 | | 12.88163 | | 10.358 | 2.377 up | | 5.750 up | | 2.419 up | Amino acid transporter LysP |
| 59952 | 0.000165 | 8.95689 | | 7.54908 | | 4.61821 | 20.233 up | | 7.625 up | | 2.653 down | Amino acid transporter PotE |
| 104077 | 0.000153 | 10.77877 | | 8.4864 | | 4.20955 | 94.957 up | | 19.384 up | | 4.898 down | Amino acid transporter PotE |
| 56314 | 0.000159 | 8.91196 | | 10.93308 | | 4.72938 | 18.158 up | | 73.705 up | | 4.058 up | Amino acid transporter PotE |
| 109122 | 0.000391 | 10.95833 | | 8.37457 | | 9.25876 | 3.248 up | | 1.845 down | | 5.995 down | Amino acid transporters |
| 5787 | 0.00226 | 9.89236 | | 7.51602 | | 7.99441 | 3.726 up | | 1.393 down | | 5.192 down | Amino acid transporters |
| 106297 | 0.00329 | 10.91684 | | 12.73489 | | 11.08302 | 1.122 down | | 3.142 up | | 3.526 up | Amino acid transporters |
| 57185 | 0.000373 | 7.09046 | | 9.2072 | | 7.06741 | 1.016 up | | 4.406 up | | 4.337 up | Amino acid transporters |
| 77616 | 0.000324 | 12.01655 | | 12.91545 | | 11.27989 | 1.666 up | | 3.107 up | | 1.864 up | aminoacyl-tRNA synthetase and proteasome regulatory subunit. |
| 81087 | 0.000119 | 10.24147 | | 11.98913 | | 4.23198 | 64.422 up | | 216.338 up | | 3.358 up | aminopeptidase Y |
| 55190 | 0.00229 | 11.77548 | | 11.15584 | | 10.22859 | 2.921 up | | 1.901 up | | 1.536 down | aminotransferase |
| 67484 | 0.00181 | 9.9133 | | 11.60366 | | 9.97307 | 1.042 down | | 3.096 up | | 3.227 up | AMP deaminase |
| 57370 | 0.000145 | 12.11442 | | 13.04349 | | 8.47572 | 12.455 up | | 23.715 up | | 1.904 up | AMP-dependent synthetase and ligase |
| 65292 | 0.000553 | 8.71056 | | 11.66333 | | 6.91132 | 3.480 up | | 26.946 up | | 7.742 up | AMP-dependent synthetase and ligase |
| 59843 | 0.000516 | 9.38252 | | 7.99069 | | 7.30545 | 4.219 up | | 1.607 up | | 2.624 down | AMP-dependent synthetase and ligase, acetoacetyl-CoA synthase-like |
| 66999 | 0.000168 | 5.65146 | | 10.27701 | | 4.66862 | 1.976 up | | 48.785 up | | 24.684 up | AMP-dependent synthetase and ligase, putative |
| 110663 | 0.00169 | 4.79614 | | 6.65823 | | 5.20215 | 1.325 down | | 2.743 up | | 3.635 up | AMP-dependent synthetase and ligase, putative |
| 62634 | 0.00133 | 9.93107 | | 11.24199 | | 9.63682 | 1.226 up | | 3.042 up | | 2.481 up | anaphase promoting complex subunit APC11 |
| 63653 | 0.0000866 | 8.3556 | | 12.48441 | | 6.75343 | 3.036 up | | 53.112 up | | 17.494 up | ankyrin |
| 70907 | 0.000278 | 10.29101 | | 13.49844 | | 5.8199 | 22.178 up | | 204.865 up | | 9.237 up | Ankyrin |
| 103193 | 0.00441 | 8.14466 | | 5.70791 | | 5.53732 | 6.093 up | | 1.125 up | | 5.414 down | ankyrin |
| 59050 | 0.00396 | 3.12515 | | 6.77112 | | 4.95882 | 3.564 down | | 3.512 up | | 12.518 up | Ankyrin |
| 123673 | 0.000755 | 8.25548 | | 11.03917 | | 7.95469 | 1.231 up | | 8.482 up | | 6.886 up | Ankyrin |
| 78010 | 0.00502 | 12.14165 | | 12.89092 | | 11.25725 | 1.845 up | | 3.103 up | | 1.680 up | ankyrin containing protein |
| 42267 | 0.000704 | 7.36005 | | 8.89232 | | 6.64542 | 1.641 up | | 4.746 up | | 2.892 up | ankyrin repeat protein |
| 73039 | 0.00266 | 7.30365 | | 6.1139 | | 5.57835 | 3.306 up | | 1.449 up | | 2.281 down | APHPhosphotransferase enzyme family aligned |
| 58584 | 0.000179 | 12.78109 | | 13.43582 | | 9.36755 | 10.655 up | | 16.775 up | | 1.574 up | aquaglyceroporin |
| 81149 | 0.000442 | 12.54819 | | 13.34208 | | 9.91741 | 6.193 up | | 10.738 up | | 1.733 up | aquaglyceroporin |
| 81082 | 0.000183 | 7.85623 | | 10.45903 | | 3.5687 | 19.528 up | | 118.630 up | | 6.074 up | aquaglyceroporin |
| 82321 | 0.000291 | 11.03641 | | 10.41882 | | 9.36522 | 3.184 up | | 2.075 up | | 1.534 down | Aquaporin |
| 105870 | 0.00069 | 11.45824 | | 10.55735 | | 9.11377 | 5.078 up | | 2.719 up | | 1.867 down | Aquaporin (major intrinsic protein family) |
| 41178 | 0.00112 | 8.25696 | | 8.86936 | | 6.71188 | 2.918 up | | 4.461 up | | 1.528 up | aquaporin-2 (major intrinsic protein) |
| 48835 | 0.000295 | 9.4441 | | 12.07807 | | 9.63493 | 1.141 down | | 5.438 up | | 6.207 up | ARF GAP zinc finger protein Gcs1 |
| 120412 | 0.0175 | 10.62699 | | 12.00139 | | 10.59941 | 1.019 up | | 2.642 up | | 2.592 up | ARF-GAP effector |
| 123738 | 0.000968 | 11.06438 | | 13.12354 | | 9.93958 | 2.180 up | | 9.088 up | | 4.167 up | Arginase |
| 55179 | 0.0000997 | 10.71292 | | 11.23704 | | 8.63341 | 4.226 up | | 6.078 up | | 1.438 up | Arginase family protein |
| 82619 | 0.000493 | 12.74354 | | 11.04893 | | 11.21311 | 2.888 up | | 1.120 down | | 3.236 down | arginosuccinate synthetase |
| 57322 | 0.000203 | 11.87523 | | 12.61024 | | 9.88894 | 3.962 up | | 6.594 up | | 1.664 up | ARO Transcriptional regulators containing a DNA-binding HTH domain |
| 105978 | 0.000819 | 9.13773 | | 9.50045 | | 7.46077 | 3.197 up | | 4.111 up | | 1.285 up | ARO8, Transcriptional regulators containing a DNA-binding HTH domain |
| 76421 | 0.000164 | 10.99707 | | 11.88751 | | 10.54775 | 1.365 up | | 2.531 up | | 1.853 up | aromatic ring-opening dioxygenase LigB subunit, putative |
| 59402 | 0.000739 | 9.98372 | | 9.47768 | | 6.49673 | 11.212 up | | 7.895 up | | 1.420 down | arsenate reductase Arc2 |
| 124170 | 0.000133 | 10.0226 | | 9.43762 | | 8.07245 | 3.864 up | | 2.576 up | | 1.500 down | arsenite methyltransferase |
| 70736 | 0.00513 | 11.03068 | | 11.94501 | | 10.36292 | 1.588 up | | 2.994 up | | 1.884 up | Arv1-like family protein |
| 123723 | 0.000321 | 12.27347 | | 11.19785 | | 8.84221 | 10.787 up | | 5.118 up | | 2.107 down | Arylacetamide deacetylase |
| 80746 | 0.00149 | 10.65103 | | 11.09281 | | 7.76901 | 7.371 up | | 10.012 up | | 1.358 up | aryl-alcohol oxidase |
| 21635 | 0.00027 | 8.7988 | | 11.0039 | | 9.09815 | 1.230 down | | 3.747 up | | 4.611 up | asparagine synthase-like protein |
| 104073 | 0.00132 | 9.72738 | | 9.40961 | | 6.58228 | 8.846 up | | 7.097 up | | 1.246 down | aspartate racemase |
| 4109 | 0.000114 | 9.86307 | | 10.5329 | | 8.12676 | 3.331 up | | 5.300 up | | 1.590 up | Aspartate/tyrosine/aromatic aminotransferase |
| 74041 | 0.0172 | 7.75874 | | 9.37499 | | 7.8513 | 1.066 down | | 2.875 up | | 3.065 up | Aspartate/tyrosine/aromatic aminotransferase |
| 53961 | 0.000559 | 11.4449 | | 10.28903 | | 7.41139 | 16.376 up | | 7.349 up | | 2.228 down | Aspartyl protease |
| 106661 | 0.00207 | 4.36641 | | 10.66732 | | 4.16248 | 1.151 up | | 90.813 up | | 78.842 up | aspartyl protease |
| 69555 | 0.00789 | 4.40932 | | 9.41 | | 5.40873 | 1.999 down | | 16.014 up | | 32.015 up | aspartyl protease, Aspergillopepsin-like |
| 54850 | 0.00308 | 11.09898 | | 11.82263 | | 10.24184 | 1.811 up | | 2.991 up | | 1.651 up | AT DNA binding protein, putative |
| 122476 | 0.000176 | 11.0333 | | 12.75703 | | 10.4207 | 1.529 up | | 5.050 up | | 3.302 up | ATG5 protein |
| 77086 | 0.000676 | 9.36738 | | 12.27622 | | 9.69673 | 1.256 down | | 5.977 up | | 7.510 up | ATP dependent DNA ligase domain-containing protein |
| 120893 | 0.035 | 11.97679 | | 12.8746 | | 11.40712 | 1.484 up | | 2.765 up | | 1.863 up | ATPase, AAA family protein, putative |
| 60873 | 0.0018 | 9.84906 | | 11.16422 | | 9.60209 | 1.186 up | | 2.952 up | | 2.488 up | ATP-dependent DNA ligase |
| 109512 | 0.000201 | 11.7428 | | 12.46775 | | 10.96824 | 1.710 up | | 2.827 up | | 1.652 up | AT-rich interaction region |
| 37368 | 0.000415 | 10.689 | | 13.38547 | | 10.54049 | 1.108 up | | 7.184 up | | 6.482 up | autophagy related lipase Atg15, putative |
| 106761 | 0.00183 | 12.10186 | | 13.30266 | | 11.68916 | 1.331 up | | 3.059 up | | 2.298 up | autophagy-related protein 9 , putative |
| 120486 | 0.000641 | 9.08581 | | 11.49898 | | 9.70048 | 1.531 down | | 3.478 up | | 5.326 up | auxiliary protein of DNA polymerase delta |
| 103850 | 0.000628 | 11.23174 | | 12.76638 | | 11.20368 | 1.019 up | | 2.954 up | | 2.897 up | Beta-1,4-mannosyltransferase |
| 68019 | 0.0004 | 9.97877 | | 7.49371 | | 6.45715 | 11.484 up | | 2.051 up | | 5.598 down | beta-lactamase superfamily |
| 112532 | 0.000358 | 8.31806 | | 9.92414 | | 6.59821 | 3.294 up | | 10.027 up | | 3.044 up | bHLH transcriptional regulator |
| 58130 | 0.00362 | 12.99251 | | 14.31717 | | 12.35553 | 1.555 up | | 3.895 up | | 2.504 up | bHLH transcriptional regulator |
| 43269 | 0.000937 | 9.43472 | | 12.18215 | | 9.9915 | 1.470 down | | 4.565 up | | 6.715 up | bHLH transcriptional regulator |
| 21255 | 0.000804 | 12.51531 | | 13.5837 | | 12.18015 | 1.261 up | | 2.645 up | | 2.097 up | bHLH transcriptional regulator |
| 122371 | 0.000335 | 12.8103 | | 13.91614 | | 12.50218 | 1.238 up | | 2.664 up | | 2.152 up | bHLH transcriptional regulator |
| 54809 | 0.00168 | 10.10695 | | 10.77507 | | 8.68819 | 2.673 up | | 4.248 up | | 1.589 up | bicarbonate transporter |
| 70803 | 0.000285 | 9.92378 | | 12.58547 | | 9.58997 | 1.260 up | | 7.975 up | | 6.327 up | bifunctional catalase/peroxidase |
| 66136 | 0.00399 | 10.68681 | | 12.67457 | | 11.12875 | 1.358 down | | 2.919 up | | 3.966 up | bimA |
| 69228 | 0.000507 | 13.22973 | | 11.93621 | | 11.29929 | 3.811 up | | 1.555 up | | 2.451 down | BioA Adenosylmethionine--amino--oxononanoate aminotransferase |
| 21509 | 0.00113 | 11.58367 | | 10.73418 | | 9.1664 | 5.341 up | | 2.964 up | | 1.801 down | blue light inducible protein BLI-3 |
| 124343 | 0.000995 | 12.08242 | | 13.58537 | | 11.67138 | 1.329 up | | 3.768 up | | 2.834 up | Blue light regulator 2, related to N. crassa blue light photoreceptor white collar-2 |
| 61190 | 0.00311 | 9.22283 | | 10.32605 | | 8.82006 | 1.322 up | | 2.840 up | | 2.148 up | BolA domain-containing protein |
| 34197 | 0.000602 | 11.80938 | | 11.34333 | | 10.13185 | 3.198 up | | 2.315 up | | 1.381 down | BolA-like protein |
| 56726 | 0.00024 | 9.57668 | | 12.32092 | | 10.98886 | 2.661 down | | 2.517 up | | 6.700 up | Branched chain alpha-keto acid dehydrogenase complex, alpha subunit |
| 75620 | 0.000131 | 10.39469 | | 11.99491 | | 10.45958 | 1.046 down | | 2.898 up | | 3.031 up | Bromodomain transcription factor |
| 72524 | 0.000615 | 9.77743 | | 7.01449 | | 8.00209 | 3.423 up | | 1.982 down | | 6.787 down | bZIP transcription factor |
| 109538 | 0.00111 | 12.62276 | | 11.66339 | | 9.48491 | 8.802 up | | 4.526 up | | 1.944 down | BZIP transcriptional regulator |
| 120365 | 0.000358 | 11.06706 | | 9.66183 | | 7.78336 | 9.738 up | | 3.676 up | | 2.648 down | BZIP transcriptional regulator |
| 110152 | 0.0194 | 10.75332 | | 11.64301 | | 10.23995 | 1.427 up | | 2.644 up | | 1.852 up | BZIP transcriptional regulator |
| 73654 | 0.00133 | 12.16705 | | 13.85439 | | 12.04996 | 1.084 up | | 3.492 up | | 3.220 up | BZIP transcriptional regulator |
| 81049 | 0.00138 | 9.74311 | | 11.58113 | | 9.13152 | 1.527 up | | 5.462 up | | 3.575 up | C-14 sterol reductase |
| 111567 | 0.000075 | 11.26583 | | 10.67977 | | 7.432 | 14.259 up | | 9.498 up | | 1.501 down | C2H2 transcription factor |
| 122448 | 0.0000871 | 7.53303 | | 12.31527 | | 10.71677 | 9.086 down | | 3.028 up | | 27.516 up | C2H2 transcription factor |
| 67418 | 0.000785 | 8.15885 | | 11.14609 | | 8.60596 | 1.363 down | | 5.816 up | | 7.929 up | C2H2 transcription factor |
| 62805 | 0.00313 | 10.54996 | | 10.87566 | | 8.28099 | 4.819 up | | 6.040 up | | 1.253 up | C2H2 transcriptional regulator |
| 102920 | 0.000447 | 12.10948 | | 12.96719 | | 9.66679 | 5.436 up | | 9.851 up | | 1.812 up | C2H2 transcriptional regulator |
| 120698 | 0.000386 | 12.50585 | | 13.48899 | | 10.31228 | 4.574 up | | 9.042 up | | 1.976 up | C2H2 transcriptional regulator |
| 108357 | 0.000254 | 13.6361 | | 14.9403 | | 10.11002 | 11.520 up | | 28.448 up | | 2.469 up | C2H2 transcriptional regulator |
| 120597 | 0.0024 | 14.15188 | | 13.45371 | | 12.18056 | 3.921 up | | 2.416 up | | 1.622 down | C2H2 transcriptional regulator |
| 121148 | 0.000177 | 12.75473 | | 13.47808 | | 11.82452 | 1.905 up | | 3.146 up | | 1.651 up | C2H2 transcriptional regulator |
| 106064 | 0.000747 | 9.80812 | | 11.49399 | | 9.18661 | 1.538 up | | 4.949 up | | 3.217 up | C2H2 transcriptional regulator |
| 45866 | 0.00174 | 10.62137 | | 12.19763 | | 10.01689 | 1.520 up | | 4.533 up | | 2.981 up | C2H2 transcriptional regulator |
| 120224 | 0.000747 | 11.71521 | | 13.6091 | | 11.36054 | 1.278 up | | 4.752 up | | 3.716 up | C2H2 transcriptional regulator |
| 70577 | 0.000384 | 11.98032 | | 13.23492 | | 11.62967 | 1.275 up | | 3.042 up | | 2.386 up | C2H2 transcriptional regulator |
| 3310 | 0.000715 | 9.03853 | | 10.90661 | | 9.27274 | 1.176 down | | 3.103 up | | 3.650 up | C2H2 transcriptional regulator |
| 25244 | 0.00163 | 11.71508 | | 13.21869 | | 11.64168 | 1.052 up | | 2.983 up | | 2.835 up | C2H2 transcriptional regulator |
| 60132 | 0.000521 | 9.88608 | | 12.12321 | | 9.846 | 1.028 up | | 4.847 up | | 4.714 up | C2H2 transcriptional regulator |
| 22785 | 0.000104 | 12.51301 | | 13.3258 | | 6.8089 | 52.132 up | | 91.576 up | | 1.756 up | C2H2 transcriptional regulator (amdA ?) |
| 53859 | 0.000146 | 12.24297 | | 11.27094 | | 10.35834 | 3.692 up | | 1.882 up | | 1.961 down | C-4 sterol methyl oxidase |
| 34985 | 0.00235 | 9.99902 | | 10.2222 | | 8.48303 | 2.859 up | | 3.338 up | | 1.167 up | C-5 cytosine-specific DNA methylase |
| 121843 | 0.000659 | 12.92335 | | 12.65306 | | 11.56821 | 2.558 up | | 2.121 up | | 1.206 down | C6HC zinc finger protein |
| 74057 | 0.00234 | 12.92541 | | 13.79268 | | 11.83067 | 2.135 up | | 3.896 up | | 1.824 up | Ca2+ permeable channel, related to N. crassa NCU07605.1 |
| 79398 | 0.00155 | 11.29974 | | 12.74002 | | 10.89576 | 1.323 up | | 3.590 up | | 2.713 up | Ca2+ transporter |
| 124222 | 0.00118 | 13.50018 | | 10.95371 | | 11.85446 | 3.129 up | | 1.867 down | | 5.842 down | CaaX-protease, related to E. nidulans rce1, involved in signal transduction |
| 68169 | 0.0003 | 8.21327 | | 7.63653 | | 5.71786 | 5.638 up | | 3.780 up | | 1.491 down | Calcium transporter |
| 4171 | 0.00084 | 9.30332 | | 7.35534 | | 6.81406 | 5.614 up | | 1.455 up | | 3.858 down | calcium transporter |
| 82544 | 0.00547 | 11.94259 | | 12.95677 | | 11.28353 | 1.579 up | | 3.189 up | | 2.019 up | Calcium transporter |
| 62362 | 0.000496 | 12.07256 | | 13.08122 | | 11.30578 | 1.701 up | | 3.423 up | | 2.012 up | calcium transporting ATPase, ion pump |
| 19432 | 0.000639 | 10.08934 | | 12.64036 | | 10.25665 | 1.122 down | | 5.218 up | | 5.860 up | calcium/sodium antiporter, contains 9 transmembrane domains |
| 102381 | 0.00103 | 9.27965 | | 11.42972 | | 9.78127 | 1.415 down | | 3.134 up | | 4.438 up | Calcium-binding EF-hand |
| 67761 | 0.000323 | 9.80325 | | 12.59787 | | 7.46051 | 5.072 up | | 35.196 up | | 6.938 up | calpain-like protease |
| 64370 | 0.00151 | 11.09894 | | 9.01617 | | 8.86592 | 4.701 up | | 1.109 up | | 4.236 down | calpain-like protease |
| 119707 | 0.00368 | 9.26079 | | 11.51293 | | 9.9824 | 1.649 down | | 2.888 up | | 4.763 up | calponin-like actin binding domain |
| 3873 | 0.000747 | 11.42217 | | 12.79235 | | 10.51359 | 1.877 up | | 4.852 up | | 2.585 up | cAMP phosphodiesterase class II PDE1, low affinity |
| 102655 | 0.00216 | 10.92281 | | 12.54707 | | 10.97789 | 1.038 down | | 2.967 up | | 3.082 up | cAMP phosphodiesterase PDE2, high affinity |
| 26462 | 0.000444 | 10.8608 | | 13.06473 | | 9.91385 | 1.927 up | | 8.881 up | | 4.607 up | cAMP-mediated signaling protein SOK1 |
| 66766 | 0.000341 | 4.41806 | | 8.91686 | | 4.59683 | 1.131 down | | 19.973 up | | 22.608 up | Carbon-nitrogen hydrolase |
| 121441 | 0.0012 | 12.83311 | | 7.92599 | | 6.13663 | 103.715 up | | 3.456 up | | 30.004 down | carbounknown proteinlic acid transporter |
| 4875 | 0.00123 | 11.38836 | | 10.77496 | | 9.17821 | 4.627 up | | 3.024 up | | 1.529 down | carboxy-cis,cis-muconate cyclase |
| 78828 | 0.00025 | 12.00361 | | 13.80564 | | 8.71347 | 9.782 up | | 34.111 up | | 3.487 up | carboxylesterase type B |
| 22459 | 0.0000557 | 11.25825 | | 13.83468 | | 7.42459 | 14.257 up | | 85.040 up | | 5.964 up | carboxypeptidase A |
| 120998 | 0.00131 | 12.79643 | | 13.83295 | | 12.22843 | 1.482 up | | 3.040 up | | 2.051 up | Carboxypeptidase Y homolog |
| 112460 | 0.000372 | 12.07398 | | 13.48898 | | 12.00298 | 1.050 up | | 2.801 up | | 2.666 up | Carn_acyltransfCholine/Carnitine o-acyltransferase |
| 122240 | 0.000222 | 13.30069 | | 14.16913 | | 12.25781 | 2.060 up | | 3.761 up | | 1.825 up | Carnitine o-acyltransferase |
| 65736 | 0.000763 | 5.11618 | | 7.86635 | | 5.02494 | 1.065 up | | 7.167 up | | 6.727 up | catabolic 3-dehydroquinase |
| 73818 | 0.000099 | 13.55829 | | 13.69036 | | 9.40546 | 17.788 up | | 19.493 up | | 1.095 up | Catalase |
| 58472 | 0.000255 | 10.77104 | | 11.38206 | | 9.6623 | 2.156 up | | 3.293 up | | 1.527 up | Catalase |
| 67538 | 0.000288 | 11.44063 | | 10.79243 | | 8.30203 | 8.806 up | | 5.619 up | | 1.567 down | Catalase |
| 120371 | 0.0000698 | 13.55321 | | 12.21942 | | 9.46217 | 17.042 up | | 6.761 up | | 2.520 down | Catalase |
| 106245 | 0.000179 | 7.79636 | | 10.08946 | | 6.70769 | 2.126 up | | 10.423 up | | 4.901 up | catalase |
| 67013 | 0.00458 | 8.64833 | | 9.50386 | | 7.95939 | 1.612 up | | 2.916 up | | 1.809 up | catalase |
| 70600 | 0.000817 | 7.6433 | | 4.47491 | | 3.2662 | 20.779 up | | 2.311 up | | 8.990 down | catalase, large subunit type |
| 62165 | 0.0000555 | 10.40953 | | 13.10216 | | 4.59722 | 56.192 up | | 363.281 up | | 6.464 up | catechol dioxigenase |
| 4876 | 0.000743 | 12.36224 | | 12.72557 | | 10.11608 | 4.744 up | | 6.102 up | | 1.286 up | catechol dioxygenase |
| 55731 | 0.0024 | 12.54982 | | 13.97183 | | 12.27012 | 1.213 up | | 3.252 up | | 2.679 up | cation channel family protein |
| 69281 | 0.0016 | 11.41145 | | 12.52963 | | 11.19916 | 1.158 up | | 2.514 up | | 2.170 up | cation diffusion facilitator family transporter |
| 55511 | 0.000674 | 10.8874 | | 12.88168 | | 10.36852 | 1.432 up | | 5.708 up | | 3.984 up | cation efflux family protein |
| 80672 | 0.00176 | 10.48968 | | 13.29231 | | 11.93325 | 2.719 down | | 2.565 up | | 6.977 up | cation efflux family protein, putative |
| 81430 | 0.00017 | 13.39909 | | 12.68944 | | 6.97817 | 85.681 up | | 52.391 up | | 1.635 down | Cation transporting ATPase |
| 81536 | 0.000425 | 8.21459 | | 5.58177 | | 3.83731 | 20.782 up | | 3.350 up | | 6.202 down | cation transporting ATPase |
| 111094 | 0.000708 | 11.43221 | | 8.68929 | | 6.21765 | 37.131 up | | 5.546 up | | 6.694 down | CBM 13 |
| 109235 | 0.000115 | 13.50376 | | 11.58695 | | 8.96824 | 23.191 up | | 6.142 up | | 3.775 down | CBM 18 |
| 70646 | 0.00665 | 10.39278 | | 11.49296 | | 10.06927 | 1.251 up | | 2.682 up | | 2.143 up | CCD1 |
| 79854 | 0.00171 | 11.29225 | | 12.83132 | | 11.31929 | 1.018 down | | 2.852 up | | 2.906 up | CCR4-NOT core complex subunit Not4 |
| 72072 | 0.000586 | 9.5892 | | 7.64455 | | 6.35504 | 9.409 up | | 2.444 up | | 3.849 down | CE1 esterase (PHB?) |
| 107850 | 0.000162 | 9.70504 | | 13.45635 | | 11.03843 | 2.519 down | | 5.343 up | | 13.466 up | CE1 poly(3-hydroxybutyrate) depolymerase |
| 103825 | 0.00102 | 8.62723 | | 9.51932 | | 6.3234 | 4.937 up | | 9.163 up | | 1.855 up | CE16 acetyl esterase |
| 65215 | 0.000105 | 9.89252 | | 12.62321 | | 6.29422 | 12.111 up | | 80.392 up | | 6.637 up | CE4 imidase |
| 105072 | 0.000107 | 10.11197 | | 12.00766 | | 9.91566 | 1.145 up | | 4.263 up | | 3.721 up | CE4 polysaccharide deacetylase |
| 54219 | 0.0000454 | 6.51878 | | 14.76905 | | 3.47858 | 8.226 up | | 2504.782 up | | 304.494 up | CE5 acetyl xylan esterase |
| 73632 | 0.000101 | 7.18943 | | 14.96511 | | 5.24 | 3.862 up | | 846.349 up | | 219.134 up | CE5 acetyl xylan esterase AXE1 |
| 60489 | 0.000216 | 11.1434 | | 13.60129 | | 5.58113 | 47.250 up | | 259.601 up | | 5.494 up | CE5 cutinase |
| 54709 | 0.000462 | 9.51534 | | 11.02999 | | 9.33997 | 1.129 up | | 3.226 up | | 2.857 up | cell cycle control protein cwf14 |
| 70811 | 0.00174 | 11.248 | | 13.06271 | | 11.36546 | 1.084 down | | 3.242 up | | 3.517 up | cell division control protein Cdc4, putative |
| 120156 | 0.00443 | 13.40489 | | 14.45207 | | 12.92762 | 1.392 up | | 2.876 up | | 2.066 up | cell morphogenesis protein PAG1 |
| 120784 | 0.000357 | 12.80146 | | 12.98589 | | 10.42547 | 5.190 up | | 5.898 up | | 1.136 up | cell wall mannoprotein |
| 120823 | 0.0000997 | 13.70551 | | 14.75535 | | 7.12155 | 95.933 up | | 198.610 up | | 2.070 up | cell wall mannoprotein |
| 122870 | 0.0015 | 10.96259 | | 13.83888 | | 11.23582 | 1.208 down | | 6.075 up | | 7.342 up | cell wall protein, CwpA |
| 104277 | 0.000453 | 13.33043 | | 12.781 | | 9.66007 | 12.731 up | | 8.699 up | | 1.463 down | cell wall protein, distantly related to A. niger CwpA. |
| 123475 | 0.000359 | 14.91595 | | 14.12363 | | 13.5096 | 2.650 up | | 1.530 up | | 1.731 down | cell wall Thr-rich mannoprotein. Distantly related to S. cerevisiae Dan4p. |
| 64397 | 0.000134 | 13.13328 | | 14.37863 | | 10.4139 | 6.585 up | | 15.613 up | | 2.370 up | Ceramidase family protein, associated toCellulase signal transduction (PMID: 15288024) |
| 31481 | 0.000105 | 9.29392 | | 12.09015 | | 10.33201 | 2.053 down | | 3.382 up | | 6.946 up | chaperone protein dnaJ 6 |
| 67678 | 0.00219 | 4.33599 | | 6.16288 | | 4.57975 | 1.184 down | | 2.996 up | | 3.547 up | chitin deacetylase |
| 60374 | 0.000156 | 7.40959 | | 8.34174 | | 4.71781 | 6.461 up | | 12.328 up | | 1.908 up | choline oxidase |
| 4136 | 0.00022 | 11.51564 | | 12.67226 | | 11.05767 | 1.373 up | | 3.062 up | | 2.229 up | chromatin (transcription) elongation factor spt5. |
| 73638 | 0.000134 | 9.0132 | | 14.54646 | | 4.4683 | 23.342 up | | 1081.007 up | | 46.310 up | CIP1 |
| 61000 | 0.00904 | 10.12273 | | 11.70724 | | 9.83691 | 1.219 up | | 3.656 up | | 2.999 up | cleavage and polyadenylylation specificity factor, putative |
| 65106 | 0.000212 | 4.4635 | | 9.59276 | | 4.24259 | 1.165 up | | 40.790 up | | 34.999 up | CN_hydrolase |
| 56625 | 0.00872 | 12.22697 | | 12.40491 | | 11.0183 | 2.311 up | | 2.614 up | | 1.131 up | CoA-transferase family III |
| 53373 | 0.000156 | 13.31599 | | 11.9532 | | 10.07157 | 9.476 up | | 3.684 up | | 2.571 down | CoA-transferase family III |
| 61227 | 0.000115 | 8.95471 | | 13.18572 | | 8.47116 | 1.398 up | | 26.255 up | | 18.778 up | cobalamin synthesis protein |
| 50616 | 0.00321 | 10.40231 | | 9.14566 | | 7.73993 | 6.330 up | | 2.649 up | | 2.389 down | Coenzyme A transferase |
| 75655 | 0.00957 | 7.27245 | | 9.2407 | | 7.46746 | 1.144 down | | 3.418 up | | 3.912 up | Component of oligomeric golgi complex Cog8 |
| 21392 | 0.00796 | 12.60825 | | 13.23705 | | 11.8934 | 1.641 up | | 2.537 up | | 1.546 up | Component, nce2, of non-classical secretion pathway |
| 5084 | 0.000289 | 11.91117 | | 6.67483 | | 7.53287 | 20.797 up | | 1.812 down | | 37.696 down | conidiation-specific protein 10 |
| 72379 | 0.00833 | 9.03262 | | 12.15522 | | 7.78125 | 2.380 up | | 20.734 up | | 8.709 up | conidiospore surface protein cmp1 |
| 60136 | 0.0015 | 10.50716 | | 13.00159 | | 11.50508 | 1.997 down | | 2.821 up | | 5.635 up | COPII coat assembly protein Sec16 |
| 121156 | 0.000221 | 8.79435 | | 10.49423 | | 5.84387 | 7.730 up | | 25.112 up | | 3.248 up | Copper amine oxidase |
| 120968 | 0.00303 | 6.19177 | | 7.83668 | | 5.90805 | 1.217 up | | 3.806 up | | 3.127 up | Copper chaperone for superoxide dismutase |
| 3856 | 0.000484 | 10.40882 | | 12.68369 | | 10.86949 | 1.376 down | | 3.516 up | | 4.839 up | copper fist DNA-binding domain-containing protein |
| 69426 | 0.00127 | 8.97151 | | 8.23011 | | 7.3516 | 3.073 up | | 1.838 up | | 1.671 down | copper transporter |
| 104958 | 0.000521 | 8.34966 | | 10.47524 | | 8.77537 | 1.343 down | | 3.248 up | | 4.363 up | CTD kinase subunit gamma, putative |
| 5196 | 0.000213 | 11.13359 | | 12.15514 | | 10.043 | 2.129 up | | 4.323 up | | 2.030 up | C-type cyclin |
| 122963 | 0.000107 | 10.92549 | | 12.87378 | | 8.8948 | 4.086 up | | 15.768 up | | 3.859 up | Cu_amine_oxidCopper amine oxidaseenzyme domain aligned |
| 123029 | 0.00213 | 13.53128 | | 12.28949 | | 9.96368 | 11.856 up | | 5.013 up | | 2.364 down | Cu2+/Zn2+ superoxide dismutase SOD1 |
| 110471 | 0.000243 | 12.45905 | | 10.07181 | | 8.39769 | 16.695 up | | 3.191 up | | 5.231 down | Cu2+/Zn2+ superoxide dismutase SOD1 |
| 63152 | 0.000819 | 11.91569 | | 11.42781 | | 9.6122 | 4.936 up | | 3.520 up | | 1.402 down | Cut9 interacting protein Scn1, putative |
| 41719 | 0.000388 | 9.85476 | | 9.43233 | | 7.82847 | 4.073 up | | 3.039 up | | 1.340 down | CutC family protein |
| 52520 | 0.00027 | 14.2357 | | 14.00311 | | 11.90963 | 5.014 up | | 4.267 up | | 1.174 down | Cyclin |
| 68924 | 0.000142 | 9.09241 | | 14.43928 | | 8.77349 | 1.247 up | | 50.765 up | | 40.697 up | cyclopropane/fatty acid synthase; plant related |
| 63919 | 0.00197 | 9.02126 | | 11.88102 | | 10.23103 | 2.313 down | | 3.138 up | | 7.258 up | Cys/Met metabolism PLP-dependent enzyme |
| 76018 | 0.000106 | 10.47681 | | 12.27482 | | 9.16249 | 2.486 up | | 8.647 up | | 3.477 up | CysKCysteine synthase |
| 68036 | 0.00114 | 13.49206 | | 12.60038 | | 11.63966 | 3.610 up | | 1.946 up | | 1.855 down | Cystathionine beta-lyases/cystathionine gamma-synthases |
| 56350 | 0.000486 | 4.55212 | | 8.0628 | | 5.41529 | 1.819 down | | 6.265 up | | 11.397 up | cysteine synthase, putative |
| 65925 | 0.00118 | 6.89863 | | 8.70829 | | 6.3375 | 1.475 up | | 5.172 up | | 3.505 up | cytidine and deoxycytidylate deaminase zinc-binding region |
| 42825 | 0.000463 | 13.20869 | | 13.78928 | | 11.82233 | 2.614 up | | 3.909 up | | 1.495 up | Cytidine/deoxycytidylate deaminase |
| 31551 | 0.000164 | 9.46283 | | 11.69266 | | 10.35443 | 1.855 down | | 2.528 up | | 4.690 up | Cytidylyltransferase |
| 22481 | 0.00227 | 11.4436 | | 12.85781 | | 10.15132 | 2.449 up | | 6.527 up | | 2.665 up | cytochrome b2, mitochondrial precursor |
| 64377 | 0.00655 | 7.33496 | | 6.68924 | | 5.11964 | 4.643 up | | 2.968 up | | 1.564 down | Cytochrome P only |
| 77512 | 0.000302 | 12.66359 | | 13.88965 | | 10.84852 | 3.518 up | | 8.231 up | | 2.339 up | cytochrome P450 |
| 54166 | 0.00351 | 12.54841 | | 11.87927 | | 11.1393 | 2.655 up | | 1.670 up | | 1.590 down | Cytochrome P450 |
| 105768 | 0.00117 | 4.60674 | | 6.71086 | | 4.80063 | 1.143 down | | 3.758 up | | 4.299 up | Cytochrome P450 |
| 75886 | 0.00127 | 9.7919 | | 12.3805 | | 9.70726 | 1.060 up | | 6.378 up | | 6.015 up | Cytochrome P450 |
| 65107 | 0.00593 | 11.65905 | | 12.33677 | | 10.47712 | 2.268 up | | 3.629 up | | 1.599 up | cytochrome P450 |
| 67377 | 0.00016 | 8.92452 | | 11.46252 | | 7.57782 | 2.543 up | | 14.771 up | | 5.807 up | Cytochrome P450 / E-class P450, group I |
| 69648 | 0.00154 | 3.98874 | | 8.65869 | | 6.45668 | 5.532 down | | 4.601 up | | 25.456 up | Cytochrome P450 CYP11/CYP12/CYP24/CYP27 subfamilies |
| 70842 | 0.000289 | 9.41885 | | 9.27097 | | 3.8936 | 46.054 up | | 41.567 up | | 1.107 down | Cytochrome P450 CYP2 subfamily |
| 67964 | 0.000131 | 13.24343 | | 13.74666 | | 8.01516 | 37.485 up | | 53.131 up | | 1.417 up | Cytochrome P450 CYP2 subfamily |
| 66534 | 0.00103 | 9.55717 | | 8.54873 | | 6.99125 | 5.921 up | | 2.943 up | | 2.011 down | Cytochrome P450 CYP2 subfamily |
| 64900 | 0.000726 | 7.39494 | | 8.73825 | | 5.2189 | 4.519 up | | 11.466 up | | 2.537 up | Cytochrome P450 CYP2 subfamily |
| 27706 | 0.00116 | 10.23613 | | 12.11684 | | 8.66856 | 2.964 up | | 10.915 up | | 3.682 up | Cytochrome P450 CYP2 subfamily |
| 52432 | 0.0000682 | 10.0931 | | 11.5648 | | 9.47129 | 1.538 up | | 4.267 up | | 2.773 up | Cytochrome P450 CYP2 subfamily |
| 59377 | 0.00585 | 5.37697 | | 7.0002 | | 5.43964 | 1.044 down | | 2.949 up | | 3.080 up | Cytochrome P450 CYP2 subfamily |
| 103215 | 0.000464 | 11.54249 | | 12.49462 | | 9.51233 | 4.084 up | | 7.902 up | | 1.934 up | Cytochrome P450 CYP4/CYP19/CYP26 subfamilies |
| 64869 | 0.000389 | 6.58142 | | 10.6922 | | 7.93907 | 2.562 down | | 6.741 up | | 17.276 up | Cytochrome P450 CYP4/CYP19/CYP26 subfamilies |
| 4517 | 0.000336 | 5.18506 | | 10.84276 | | 9.03949 | 14.464 down | | 3.490 up | | 50.481 up | Cytochrome P450 CYP4/CYP19/CYP26 subfamilies |
| 68705 | 0.000151 | 12.22102 | | 12.48304 | | 8.44123 | 13.735 up | | 16.470 up | | 1.199 up | cytochrome P450 monooxygenase |
| 58772 | 0.000221 | 12.1634 | | 12.44472 | | 9.75483 | 5.309 up | | 6.452 up | | 1.215 up | cytochrome P450 monooxygenase |
| 4726 | 0.000522 | 12.91813 | | 13.61709 | | 10.68113 | 4.714 up | | 7.652 up | | 1.623 up | Cytochrome P450 monooxygenase |
| 57555 | 0.000173 | 9.34452 | | 10.25633 | | 5.54272 | 13.946 up | | 26.238 up | | 1.881 up | cytochrome P450 monooxygenase |
| 4999 | 0.00018 | 9.24237 | | 14.0152 | | 4.89991 | 20.286 up | | 554.594 up | | 27.337 up | cytochrome P450 monooxygenase |
| 70956 | 0.000954 | 7.24552 | | 10.49452 | | 4.10733 | 8.804 up | | 83.702 up | | 9.507 up | cytochrome P450 monooxygenase |
| 65141 | 0.000358 | 6.53519 | | 12.01436 | | 8.19377 | 3.157 down | | 14.129 up | | 44.606 up | cytochrome P450 monooxygenase |
| 61212 | 0.000173 | 11.12028 | | 13.40075 | | 10.39689 | 1.651 up | | 8.021 up | | 4.858 up | cytochrome P450 monooxygenase |
| 66453 | 0.000288 | 11.36674 | | 12.41266 | | 10.96372 | 1.322 up | | 2.730 up | | 2.064 up | cytochrome P450 monooxygenase |
| 109746 | 0.000279 | 12.59909 | | 12.4444 | | 9.54628 | 8.298 up | | 7.454 up | | 1.113 down | cytochrome P450 monooxygenase, putative |
| 56966 | 0.00271 | 8.57049 | | 11.01993 | | 8.33657 | 1.176 up | | 6.423 up | | 5.462 up | Cytochrome P450, putative |
| 72137 | 0.00197 | 9.51293 | | 6.95143 | | 5.56719 | 15.409 up | | 2.610 up | | 5.903 down | cytosceleton binding protein |
| 80034 | 0.000902 | 12.23037 | | 11.2993 | | 10.5299 | 3.250 up | | 1.704 up | | 1.906 down | DadA Glycine/D-amino acid oxidases (deaminating) [Amino acid transport and metabolism] |
| 59338 | 0.000259 | 8.03442 | | 9.74601 | | 6.4337 | 3.032 up | | 9.933 up | | 3.275 up | D-Alanine aminotransferase |
| 43347 | 0.00109 | 9.63284 | | 10.62654 | | 8.67314 | 1.944 up | | 3.872 up | | 1.991 up | DASH complex subunit Dad3, putative |
| 59668 | 0.00216 | 10.73983 | | 12.99143 | | 10.34419 | 1.315 up | | 6.264 up | | 4.762 up | DBF2, cell cyle protein kinase |
| 53660 | 0.000769 | 11.58979 | | 11.92832 | | 10.52007 | 2.099 up | | 2.654 up | | 1.264 up | DEAD/DEAH box helicase |
| 58696 | 0.00109 | 10.44601 | | 11.81681 | | 10.13062 | 1.244 up | | 3.218 up | | 2.586 up | DEAD/DEAH box helicase |
| 53986 | 0.00675 | 8.61061 | | 9.81542 | | 8.38188 | 1.171 up | | 2.701 up | | 2.305 up | deounknown proteinribonuclease TatD |
| 4430 | 0.00185 | 8.08039 | | 9.24574 | | 7.32261 | 1.690 up | | 3.792 up | | 2.242 up | Developmental regulatory protein WetA |
| 5296 | 0.000388 | 10.85334 | | 12.11508 | | 9.96396 | 1.852 up | | 4.441 up | | 2.397 up | DHQase_I, Type I 3-dehydroquinase |
| 70961 | 0.00227 | 10.68642 | | 10.48511 | | 8.80388 | 3.687 up | | 3.207 up | | 1.149 down | dienelactone hydrolase |
| 33169 | 0.000255 | 10.41189 | | 11.50299 | | 9.59045 | 1.767 up | | 3.764 up | | 2.130 up | dienelactone hydrolase |
| 53123 | 0.008 | 8.45242 | | 9.69931 | | 8.20303 | 1.188 up | | 2.821 up | | 2.373 up | dihydrodipicolinate synthase, putative |
| 120473 | 0.000133 | 7.9968 | | 11.49496 | | 8.22969 | 1.175 down | | 9.614 up | | 11.299 up | Dihydrolipoamide transacylase (alpha-keto acid dehydrogenase E2 subunit) |
| 21873 | 0.0083 | 11.17586 | | 11.44223 | | 9.75911 | 2.669 up | | 3.211 up | | 1.202 up | dihydrounknown protein-acid dehydratase |
| 4480 | 0.000237 | 13.2099 | | 13.0868 | | 10.91692 | 4.900 up | | 4.499 up | | 1.089 down | dihydroxacetione kinase Dak1 |
| 108835 | 0.000235 | 9.92443 | | 10.89008 | | 9.16527 | 1.692 up | | 3.305 up | | 1.952 up | dihydroxyacetone kinase (Dak kinase) |
| 47077 | 0.00174 | 10.59496 | | 10.17496 | | 9.24372 | 2.551 up | | 1.906 up | | 1.337 down | diounknown proteingenase |
| 111694 | 0.000381 | 10.7542 | | 13.16943 | | 11.70999 | 1.939 down | | 2.750 up | | 5.334 up | dipeptidase |
| 66608 | 0.00177 | 11.32053 | | 13.16571 | | 11.64978 | 1.256 down | | 2.859 up | | 3.592 up | dipeptidyl aminopeptidase, alpha factor processing |
| 56830 | 0.000163 | 11.97912 | | 12.52078 | | 8.82831 | 8.881 up | | 12.928 up | | 1.455 up | dipeptidyl peptidase 5 |
| 2044 | 0.000195 | 9.57945 | | 10.39787 | | 7.30584 | 4.835 up | | 8.526 up | | 1.763 up | dipeptidyl peptidase 5 |
| 61293 | 0.0000875 | 9.03234 | | 10.90989 | | 4.50781 | 23.015 up | | 84.569 up | | 3.674 up | dipeptidyl peptidase 5 |
| 63692 | 0.00147 | 8.7908 | | 8.52069 | | 7.46517 | 2.506 up | | 2.078 up | | 1.205 down | dipeptidyl peptidase 5 |
| 58509 | 0.000603 | 9.51412 | | 11.04862 | | 9.38574 | 1.093 up | | 3.166 up | | 2.896 up | DNA ligase IV involved in non-homologous end joining of double-strand DNA breaks |
| 59147 | 0.000146 | 9.42325 | | 12.10326 | | 9.38647 | 1.025 up | | 6.574 up | | 6.408 up | DNA lyase |
| 107680 | 0.00447 | 10.41541 | | 10.75691 | | 8.61646 | 3.479 up | | 4.408 up | | 1.267 up | DNA photolyase, class I, rapidly regulated by blue light in T. harzianum |
| 77473 | 0.00206 | 8.25961 | | 9.04087 | | 7.35427 | 1.872 up | | 3.218 up | | 1.718 up | DNA photolyase, N-terminal, class 1, FAD-binding |
| 59726 | 0.00103 | 2.84074 | | 4.33995 | | 3.00373 | 1.119 down | | 2.524 up | | 2.826 up | DNA photolyase, N-terminal, class 1, FAD-binding |
| 53105 | 0.000939 | 8.61586 | | 11.73463 | | 8.80859 | 1.142 down | | 7.600 up | | 8.686 up | DNA polymerase alpha catalytic subunit |
| 79187 | 0.000386 | 10.08916 | | 12.57314 | | 10.80099 | 1.637 down | | 3.415 up | | 5.594 up | DNA polymerase alpha-primase complex, polymerase-associated subunit B |
| 58184 | 0.00217 | 10.73306 | | 11.77368 | | 10.30416 | 1.346 up | | 2.769 up | | 2.057 up | DNA polymerase epsilon, subunit B |
| 66394 | 0.00126 | 11.28543 | | 11.46002 | | 8.81286 | 5.550 up | | 6.264 up | | 1.128 up | DNA polymerase family X member. |
| 107805 | 0.00114 | 10.34462 | | 12.10726 | | 10.739 | 1.314 down | | 2.581 up | | 3.393 up | DNA polymerase kappa, a Y family lesion bypass polymerase |
| 3779 | 0.000892 | 11.17572 | | 13.14035 | | 11.3452 | 1.124 down | | 3.470 up | | 3.903 up | DNA polymerase subunit epsilon |
| 56780 | 0.00194 | 10.95313 | | 11.33193 | | 9.48541 | 2.765 up | | 3.596 up | | 1.300 up | DNA polymerase X family |
| 121573 | 0.00281 | 11.23275 | | 12.52969 | | 11.17577 | 1.040 up | | 2.556 up | | 2.457 up | DNA primase, small subunit |
| 79304 | 0.00218 | 11.5003 | | 12.49264 | | 10.41241 | 2.125 up | | 4.228 up | | 1.989 up | DNA repair endonuclease Rad2p of Saccharomyces cerevisiae |
| 62226 | 0.000285 | 9.35846 | | 10.66802 | | 9.04162 | 1.245 up | | 3.087 up | | 2.478 up | DNA repair helicase family protein |
| 53659 | 0.000894 | 10.89409 | | 11.98517 | | 10.57449 | 1.247 up | | 2.658 up | | 2.130 up | DNA repair protein (Tof1) |
| 106769 | 0.000697 | 9.48612 | | 11.91158 | | 10.42793 | 1.920 down | | 2.796 up | | 5.372 up | DNA repair protein Rad18 |
| 104000 | 0.0021 | 10.68596 | | 11.89239 | | 10.56869 | 1.084 up | | 2.503 up | | 2.307 up | DNA repair protein rad18 |
| 62057 | 0.000496 | 11.13956 | | 12.48144 | | 10.6954 | 1.360 up | | 3.448 up | | 2.534 up | DNA repair protein Rhp26/Rad26 |
| 56856 | 0.00102 | 10.05965 | | 11.15616 | | 9.5982 | 1.376 up | | 2.944 up | | 2.138 up | DNA replication complex GINS protein (Psf2), putative |
| 21653 | 0.000181 | 10.60081 | | 12.26915 | | 9.97892 | 1.538 up | | 4.891 up | | 3.178 up | DNA replication complex GINS protein PSF1, putative |
| 122819 | 0.00237 | 10.36482 | | 12.45717 | | 10.26779 | 1.069 up | | 4.561 up | | 4.264 up | DNA replication complex GINS protein PSF3 |
| 59964 | 0.00137 | 12.53435 | | 12.82508 | | 11.38508 | 2.218 up | | 2.713 up | | 1.223 up | DNA replication factor A subunit Ssb3, putative |
| 75063 | 0.00914 | 10.37927 | | 12.12116 | | 10.24868 | 1.094 up | | 3.661 up | | 3.344 up | DNA replication licensing factor mcm6 |
| 80159 | 0.00243 | 11.09913 | | 13.35388 | | 11.24141 | 1.103 down | | 4.324 up | | 4.772 up | DNA-directed DNA polymerase B |
| 74444 | 0.000822 | 11.82479 | | 12.66258 | | 11.02802 | 1.737 up | | 3.104 up | | 1.787 up | DNA-directed RNA polymerase II |
| 80714 | 0.0249 | 12.32816 | | 12.16897 | | 10.57308 | 3.375 up | | 3.022 up | | 1.116 down | Dolichyl-phosphate-mannose a-mannosyltransferase |
| 56203 | 0.00185 | 9.64373 | | 10.26266 | | 8.93117 | 1.638 up | | 2.516 up | | 1.535 up | dolichyl-phosphate-mannose a-mannosyltransferases |
| 80292 | 0.000707 | 12.80984 | | 13.55531 | | 11.61794 | 2.284 up | | 3.830 up | | 1.676 up | DSPc, Dual specificity phosphatases (DSP) |
| 5771 | 0.00518 | 10.12047 | | 11.64588 | | 10.23988 | 1.086 down | | 2.650 up | | 2.878 up | Dual specificity phosphatase |
| 61553 | 0.0016 | 9.68147 | | 8.4464 | | 7.55208 | 4.375 up | | 1.858 up | | 2.353 down | D-xylulose 5-phosphate/D-fructose 6-phosphate phosphoketolase |
| 112283 | 0.000233 | 10.47334 | | 11.56545 | | 9.61284 | 1.815 up | | 3.870 up | | 2.131 up | dynactin Arp1 p62 subunit |
| 68192 | 0.000223 | 9.93089 | | 11.37403 | | 8.30895 | 3.077 up | | 8.369 up | | 2.719 up | dynamin family protein |
| 75290 | 0.00568 | 7.45674 | | 8.44502 | | 6.9799 | 1.391 up | | 2.760 up | | 1.983 up | dynamin GTPase |
| 55417 | 0.00546 | 10.46577 | | 12.0987 | | 10.31698 | 1.108 up | | 3.438 up | | 3.101 up | dynein heavy chain |
| 77672 | 0.00418 | 11.84597 | | 13.12468 | | 11.60875 | 1.178 up | | 2.859 up | | 2.426 up | effector of Sec4; Sro7 / Sro77 |
| 70800 | 0.000944 | 8.94524 | | 9.36882 | | 6.22559 | 6.587 up | | 8.835 up | | 1.341 up | elastinolytic metalloproteinase |
| 22466 | 0.00474 | 11.65926 | | 12.49276 | | 10.91662 | 1.673 up | | 2.981 up | | 1.782 up | endomembrane protein 70 |
| 3914 | 0.00268 | 10.63855 | | 6.1604 | | 7.0596 | 11.950 up | | 1.865 down | | 22.287 down | Endoplasmic reticulum protein EP58 |
| 5598 | 0.0042 | 12.31043 | | 12.90352 | | 11.56326 | 1.678 up | | 2.531 up | | 1.508 up | Endosome-associated ubiquitin isopeptidase |
| 110406 | 0.000164 | 10.83414 | | 11.13493 | | 8.66802 | 4.488 up | | 5.528 up | | 1.231 up | enoyl-CoA hydratase/isomerase |
| 108898 | 0.021 | 8.74139 | | 9.80998 | | 8.32172 | 1.337 up | | 2.805 up | | 2.097 up | enoyl-CoA hydratase/isomerase |
| 69647 | 0.00318 | 6.5826 | | 8.44397 | | 6.87296 | 1.222 down | | 2.971 up | | 3.633 up | enoyl-CoA hydratase/isomerase |
| 6085 | 0.000737 | 3.76561 | | 6.49751 | | 3.97742 | 1.158 down | | 5.736 up | | 6.643 up | Enoyl-CoA hydratase/isomerase, putative |
| 106082 | 0.00232 | 11.93104 | | 12.2141 | | 10.15089 | 3.434 up | | 4.179 up | | 1.216 up | Epl1/Sm1 |
| 104584 | 0.00018 | 10.28634 | | 12.54683 | | 8.86814 | 2.672 up | | 12.805 up | | 4.791 up | Epl1/Sm1 |
| 82662 | 0.00154 | 14.10607 | | 10.49394 | | 11.45125 | 6.297 up | | 1.941 down | | 12.228 down | Epl1/Sm1 |
| 108684 | 0.00564 | 7.82944 | | 8.61516 | | 6.95594 | 1.832 up | | 3.158 up | | 1.723 up | Epl1/Sm1 |
| 110272 | 0.00561 | 8.12023 | | 10.01028 | | 7.38337 | 1.666 up | | 6.177 up | | 3.706 up | Epl1/Sm1 |
| 123955 | 0.000426 | 9.63972 | | 13.84261 | | 9.2461 | 1.313 up | | 24.192 up | | 18.416 up | Epl1/Sm1 |
| 121877 | 0.00125 | 10.63297 | | 11.27024 | | 9.91918 | 1.640 up | | 2.551 up | | 1.555 up | epoxide hydrolase |
| 124187 | 0.00138 | 9.89531 | | 11.96698 | | 9.78226 | 1.081 up | | 4.546 up | | 4.203 up | ER-associated protein degradation |
| 22637 | 0.000389 | 8.38299 | | 11.83534 | | 6.96651 | 2.669 up | | 29.218 up | | 10.946 up | ERCC8 (CSA, CKN1) involved in transcription-coupled nucleotideExcision repair |
| 74379 | 0.000354 | 8.43121 | | 10.93531 | | 9.21617 | 1.723 down | | 3.292 up | | 5.672 up | ERG2 C-8 sterol isomerase |
| 69742 | 0.000192 | 5.50867 | | 10.8932 | | 9.29585 | 13.805 down | | 3.025 up | | 41.773 up | esterase |
| 104394 | 0.000311 | 8.04994 | | 9.09232 | | 7.72539 | 1.252 up | | 2.579 up | | 2.059 up | esterase |
| 58282 | 0.000453 | 13.70872 | | 14.09822 | | 11.69494 | 4.038 up | | 5.290 up | | 1.309 up | esterase family 9 |
| 107938 | 0.000145 | 10.42736 | | 11.88633 | | 9.2898 | 2.200 up | | 6.048 up | | 2.749 up | esterase or lipase |
| 106120 | 0.0000451 | 8.56498 | | 13.76316 | | 8.04923 | 1.429 up | | 52.488 up | | 36.711 up | esterase/lipase |
| 106697 | 0.00102 | 7.13889 | | 4.3959 | | 4.61622 | 5.746 up | | 1.164 down | | 6.694 down | esterase/lipase, HGT |
| 65677 | 0.000233 | 11.08378 | | 11.83026 | | 9.20568 | 3.675 up | | 6.167 up | | 1.677 up | Esterase/lipase/thioesterase |
| 78852 | 0.00148 | 9.23642 | | 11.8624 | | 8.9761 | 1.197 up | | 7.393 up | | 6.173 up | Esterase/lipase/thioesterase |
| 60163 | 0.00205 | 8.81917 | | 9.45241 | | 8.11105 | 1.633 up | | 2.533 up | | 1.551 up | Esterase/lipase/thioesterase domain; alpha/beta hydrolase, putative |
| 58170 | 0.000632 | 11.79848 | | 13.58447 | | 11.86864 | 1.049 down | | 3.284 up | | 3.448 up | ethanolamine kinase, putative |
| 124333 | 0.000138 | 10.40551 | | 12.2428 | | 10.51324 | 1.077 down | | 3.316 up | | 3.573 up | eukaryotic initiation factor 1A (eIF-1A). |
| 1673 | 0.00187 | 9.75821 | | 11.07495 | | 8.99701 | 1.694 up | | 4.222 up | | 2.491 up | Eukaryotic translation initiation factor 4E (eIF-4E) |
| 76514 | 0.00357 | 12.60317 | | 12.99876 | | 11.52785 | 2.107 up | | 2.771 up | | 1.315 up | Eukaryotic-like DNA topoisomerase I |
| 55857 | 0.003 | 10.92307 | | 12.01477 | | 10.62736 | 1.227 up | | 2.616 up | | 2.131 up | Exocyst component Ex084 |
| 55945 | 0.000361 | 10.49242 | | 12.28147 | | 10.50185 | 1.006 down | | 3.433 up | | 3.455 up | Exocyst component Sec5 |
| 57613 | 0.000734 | 11.35472 | | 12.30884 | | 9.32362 | 4.087 up | | 7.918 up | | 1.937 up | exonuclease family protein |
| 107463 | 0.000448 | 10.5335 | | 12.39172 | | 10.94388 | 1.329 down | | 2.727 up | | 3.625 up | exoribonuclease Dhp1 |
| 106405 | 0.0000996 | 5.89979 | | 9.48948 | | 6.0469 | 1.107 down | | 10.872 up | | 12.039 up | extracellular lipase-like protein |
| 105823 | 0.00157 | 9.61719 | | 7.30313 | | 6.86134 | 6.754 up | | 1.358 up | | 4.972 down | FAD binding domain protein |
| 109201 | 0.000362 | 5.27318 | | 9.61067 | | 5.92998 | 1.576 down | | 12.823 up | | 20.217 up | FAD binding domain-containing protein |
| 66726 | 0.000302 | 8.98598 | | 8.9191 | | 5.0611 | 15.188 up | | 14.500 up | | 1.047 down | FAD binding protein |
| 67699 | 0.000165 | 10.94342 | | 10.05853 | | 5.81273 | 35.034 up | | 18.971 up | | 1.846 down | FAD/NAD-oxidoreductase, only in Hypocreaceae |
| 104438 | 0.00143 | 7.71334 | | 8.7911 | | 6.06075 | 3.143 up | | 6.636 up | | 2.110 up | FAD-binding, oxidoreductase |
| 5345 | 0.000213 | 10.5642 | | 12.44345 | | 6.47402 | 17.032 up | | 62.658 up | | 3.678 up | FAD-containing oxidoreductase |
| 111357 | 0.000115 | 12.78656 | | 12.49117 | | 10.44776 | 5.058 up | | 4.122 up | | 1.227 down | FAD-dependent oxidoreductase |
| 48788 | 0.00339 | 12.50434 | | 13.61896 | | 11.92039 | 1.498 up | | 3.245 up | | 2.165 up | fatty acid synthase - candidate FAS2 |
| 48653 | 0.000742 | 10.17729 | | 11.65876 | | 10.08337 | 1.067 up | | 2.980 up | | 2.792 up | F-box domain-containing protein |
| 69224 | 0.00105 | 10.11032 | | 11.85134 | | 10.19688 | 1.061 down | | 3.148 up | | 3.342 up | F-box protein involved in recycling plasma membrane proteins internalized by endocytosis |
| 79756 | 0.00262 | 10.2061 | | 12.02705 | | 10.68955 | 1.398 down | | 2.527 up | | 3.533 up | F-Box/WD40 repeat protein FWD-1, targets the clock component FRQ for degradation |
| 106936 | 0.000145 | 4.87144 | | 11.89702 | | 3.73297 | 2.201 up | | 286.830 up | | 130.289 up | ferric reductase |
| 6107 | 0.000254 | 6.36946 | | 10.40729 | | 5.08191 | 2.441 up | | 40.095 up | | 16.425 up | ferric reductase |
| 110666 | 0.000104 | 4.01541 | | 13.73778 | | 8.80125 | 27.585 down | | 30.622 up | | 844.745 up | ferric reductase |
| 111893 | 0.00027 | 8.78246 | | 12.67144 | | 8.76849 | 1.009 up | | 14.959 up | | 14.814 up | ferric reductase |
| 102820 | 0.000519 | 8.11773 | | 11.19078 | | 6.59715 | 2.869 up | | 24.144 up | | 8.415 up | ferrooxidoreductase |
| 70452 | 0.000342 | 10.73679 | | 12.20535 | | 10.52162 | 1.160 up | | 3.212 up | | 2.767 up | flavin containing amine oxidoreductase |
| 55358 | 0.00018 | 4.49283 | | 11.24778 | | 3.40233 | 2.129 up | | 229.992 up | | 108.004 up | flavin-binding monooxygenase, putative |
| 76696 | 0.000103 | 13.35543 | | 13.6249 | | 8.03528 | 39.950 up | | 48.155 up | | 1.205 up | Flavin-containing monooxygenase |
| 81649 | 0.000388 | 11.47562 | | 11.8968 | | 9.89706 | 2.986 up | | 3.999 up | | 1.339 up | Flavin-containing monooxygenase |
| 76230 | 0.000309 | 10.1164 | | 12.62746 | | 3.12914 | 126.874 up | | 723.234 up | | 5.700 up | Flavin-containing monooxygenase |
| 70988 | 0.0167 | 8.2304 | | 5.713 | | 6.90047 | 2.513 up | | 2.277 down | | 5.725 down | Flavin-containing monooxygenase |
| 50786 | 0.00114 | 11.63375 | | 13.6356 | | 12.18174 | 1.462 down | | 2.739 up | | 4.005 up | Flavin-linked sulfhydryl oxidase |
| 111716 | 0.000332 | 8.92646 | | 9.48252 | | 7.65903 | 2.407 up | | 3.539 up | | 1.470 up | Flavonol reductase/cinnamoyl-CoA reductase |
| 3719 | 0.000449 | 7.55247 | | 8.81851 | | 5.81456 | 3.335 up | | 8.021 up | | 2.405 up | Flavonol reductase/cinnamoyl-CoA reductase |
| 23083 | 0.000114 | 10.59715 | | 12.8558 | | 8.42786 | 4.498 up | | 21.524 up | | 4.785 up | Flavoprotein monooxygenase |
| 4146 | 0.0000849 | 9.65311 | | 12.9214 | | 6.51528 | 8.801 up | | 84.807 up | | 9.635 up | flavoprotein monooxygenase |
| 59095 | 0.000176 | 9.79911 | | 11.25368 | | 7.08174 | 6.576 up | | 18.025 up | | 2.740 up | Flavoprotein monooxygenase, putative |
| 2517 | 0.000681 | 4.71707 | | 6.49522 | | 4.27672 | 1.356 up | | 4.654 up | | 3.429 up | Flavoprotein monooxygenase, putative |
| 81679 | 0.000362 | 7.59558 | | 9.83991 | | 7.80191 | 1.153 down | | 4.106 up | | 4.738 up | Flavoprotein monooxygenase, putative |
| 106480 | 0.00128 | 4.55341 | | 7.32349 | | 4.86687 | 1.242 down | | 5.489 up | | 6.821 up | flavoprotein monooxygenases |
| 70644 | 0.000477 | 10.38628 | | 11.2316 | | 9.76916 | 1.533 up | | 2.755 up | | 1.796 up | Flippase |
| 64029 | 0.00817 | 11.05625 | | 10.0127 | | 9.67755 | 2.600 up | | 1.261 up | | 2.061 down | folylpolyglutamate synthase , putative |
| 21211 | 0.00321 | 12.30492 | | 13.38582 | | 11.85028 | 1.370 up | | 2.898 up | | 2.115 up | Forkhead |
| 70517 | 0.000356 | 10.22655 | | 12.20974 | | 10.45138 | 1.168 down | | 3.383 up | | 3.953 up | Forkhead |
| 107682 | 0.0087 | 8.26571 | | 10.07347 | | 8.47233 | 1.153 down | | 3.033 up | | 3.500 up | Forkhead |
| 81511 | 0.000324 | 11.38999 | | 11.74659 | | 9.26819 | 4.352 up | | 5.572 up | | 1.280 up | formamidase |
| 40346 | 0.00311 | 10.31494 | | 11.83452 | | 10.27358 | 1.029 up | | 2.950 up | | 2.867 up | formamidopyrimidine-DNA glycosylase |
| 121670 | 0.000726 | 12.57701 | | 13.39805 | | 11.79719 | 1.716 up | | 3.033 up | | 1.766 up | Frequency clock protein FRQ-1 |
| 65508 | 0.000254 | 13.05014 | | 13.86899 | | 12.04073 | 2.013 up | | 3.551 up | | 1.763 up | fructose-bisphosphatase |
| 81303 | 0.00369 | 13.00645 | | 14.04935 | | 12.28123 | 1.653 up | | 3.406 up | | 2.060 up | fumarate reductase |
| 77749 | 0.000723 | 11.97159 | | 13.35952 | | 11.14221 | 1.776 up | | 4.650 up | | 2.617 up | fumarylacetoacetase |
| 66592 | 0.00201 | 7.85023 | | 6.3513 | | 4.84106 | 8.051 up | | 2.848 up | | 2.826 down | Fumarylacetoacetate (FAA) hydrolase |
| 108482 | 0.000414 | 8.75533 | | 11.61807 | | 9.00495 | 1.188 down | | 6.118 up | | 7.273 up | fumarylacetoacetate hydrolase |
| 76706 | 0.000497 | 10.32815 | | 11.79274 | | 10.03044 | 1.229 up | | 3.392 up | | 2.759 up | fungal specific transcription factor domain-containing protein |
| 69437 | 0.00164 | 11.30266 | | 12.62086 | | 11.28411 | 1.012 up | | 2.525 up | | 2.493 up | G2/mitotic-specific cyclin |
| 79835 | 0.00494 | 12.35745 | | 13.24156 | | 11.91178 | 1.361 up | | 2.513 up | | 1.845 up | GadB Glutamate decarboxylase and related PLP-dependent proteins |
| 68585 | 0.00179 | 4.80164 | | 7.98644 | | 3.76493 | 2.051 up | | 18.655 up | | 9.093 up | galactose-6-phosphate isomerase |
| 57735 | 0.000442 | 12.87032 | | 13.74067 | | 11.62798 | 2.365 up | | 4.324 up | | 1.828 up | GATA type transcriptional regulator |
| 123771 | 0.000306 | 11.02793 | | 13.07218 | | 10.56619 | 1.377 up | | 5.680 up | | 4.124 up | GCD, Glutaryl-CoA dehydrogenase |
| 103537 | 0.000213 | 6.2059 | | 11.9972 | | 4.82812 | 2.598 up | | 143.916 up | | 55.380 up | GCN5-N-acetyltransferase |
| 106798 | 0.000255 | 9.82462 | | 12.53657 | | 11.00429 | 2.265 down | | 2.892 up | | 6.552 up | GCN5-N-acetyltransferase |
| 106829 | 0.00112 | 11.1679 | | 11.95069 | | 10.28874 | 1.839 up | | 3.164 up | | 1.720 up | GCN5-N-acetyltransferase |
| 58227 | 0.000659 | 5.1196 | | 8.05107 | | 5.63152 | 1.425 down | | 5.350 up | | 7.628 up | GCN5-related acetyltransferase |
| 112665 | 0.00721 | 9.82372 | | 8.02277 | | 8.27703 | 2.921 up | | 1.192 down | | 3.484 down | GCN5-related N-acetyltransferase |
| 2015 | 0.000245 | 12.50688 | | 11.43946 | | 8.64777 | 14.511 up | | 6.924 up | | 2.095 down | GCN5-related N-acetyltransferase |
| 33387 | 0.000744 | 12.85963 | | 13.00697 | | 9.57972 | 9.712 up | | 10.757 up | | 1.107 up | GCN5-related N-acetyltransferase, putative |
| 119819 | 0.00016 | 10.23333 | | 12.126 | | 10.43588 | 1.150 down | | 3.226 up | | 3.713 up | GCPR, mPR-type |
| 103451 | 0.000254 | 9.40953 | | 10.18109 | | 7.93708 | 2.774 up | | 4.737 up | | 1.707 up | GDP-forming succinate-CoA ligase, beta subunit |
| 69868 | 0.000074 | 5.57553 | | 9.66559 | | 5.93787 | 1.285 down | | 13.248 up | | 17.030 up | GDP-mannose a-mannosyltransferases;Distant relative |
| 32364 | 0.000449 | 12.00687 | | 10.52321 | | 7.21768 | 27.649 up | | 9.886 up | | 2.796 down | GDSL lipase |
| 61703 | 0.000158 | 6.9351 | | 10.88696 | | 7.60066 | 1.586 down | | 9.756 up | | 15.474 up | germinal center kinase, related to S. cerevisiae Kic1 |
| 57016 | 0.008 | 12.26066 | | 12.94592 | | 11.58531 | 1.596 up | | 2.567 up | | 1.607 up | germinal center kinase, related to S. cerevisiae Kic1p |
| 21373 | 0.000507 | 10.43582 | | 12.93482 | | 10.76457 | 1.255 down | | 4.501 up | | 5.652 up | germinal center kinase, related to S. cerevisiae Sps1p |
| 120749 | 0.000238 | 13.19302 | | 13.82358 | | 8.56656 | 24.700 up | | 38.240 up | | 1.548 up | GH1 ß-glucosidase BGL2/CEL1a |
| 22197 | 0.000128 | 13.1206 | | 14.34961 | | 10.80234 | 4.987 up | | 11.690 up | | 2.344 up | GH1 ß-glucosidase CEL1b |
| 120229 | 0.0000341 | 7.1759 | | 14.58926 | | 4.00368 | 9.014 up | | 1536.653 up | | 170.467 up | GH10 endo-ß-1,4-xylanase XYN3 |
| 57179 | 0.000189 | 5.09815 | | 9.502 | | 3.60155 | 2.821 up | | 59.732 up | | 21.168 up | GH105/GH88 glycosyl hydrolase |
| 74223 | 0.000338 | 5.29753 | | 13.12734 | | 4.45669 | 1.791 up | | 407.497 up | | 227.512 up | GH11 endo-ß-1,4-xylanase XYN1 |
| 123818 | 0.000106 | 11.69446 | | 15.2787 | | 6.75438 | 30.698 up | | 368.193 up | | 11.994 up | GH11 endo-ß-1,4-xylanase XYN2 |
| 112392 | 0.000104 | 4.89618 | | 13.31656 | | 4.69083 | 1.152 up | | 395.006 up | | 342.601 up | GH11 endo-ß-1,4-xylanase XYN5 |
| 123940 | 0.000104 | 4.83643 | | 14.67437 | | 3.8077 | 2.040 up | | 1867.208 up | | 915.199 up | GH115 methylglurunoyl esterase CIP2 |
| 79606 | 0.000585 | 13.12166 | | 13.85462 | | 10.60999 | 5.702 up | | 9.478 up | | 1.662 up | GH115 xylan-α-1,2-glucuronidase or α-(4-O-methyl)-glucuronidase |
| 123232 | 0.0000533 | 10.75466 | | 14.26383 | | 4.12138 | 99.269 up | | 1130.265 up | | 11.385 up | GH12 endo-ß-1,4-glucanase |
| 59578 | 0.00113 | 7.31945 | | 10.19003 | | 7.37458 | 1.038 down | | 7.039 up | | 7.313 up | GH13 α-glucosidase |
| 108477 | 0.000779 | 12.20554 | | 12.30056 | | 9.42424 | 6.874 up | | 7.342 up | | 1.068 up | GH13 α-glucosidase/oligo α-glucosidase |
| 1885 | 0.000464 | 12.54053 | | 14.09449 | | 11.73769 | 1.744 up | | 5.122 up | | 2.936 up | GH15 glucamylase with starch binding domain |
| 69751 | 0.00196 | 8.28342 | | 8.99631 | | 6.05828 | 4.675 up | | 7.663 up | | 1.639 up | GH16 |
| 76266 | 0.00298 | 12.35088 | | 13.62006 | | 11.83434 | 1.430 up | | 3.447 up | | 2.410 up | GH16 cell wall glucanosyltransferase |
| 55886 | 0.00421 | 7.58107 | | 10.09699 | | 4.3111 | 9.646 up | | 55.173 up | | 5.719 up | GH16 glucan endo-1,3(4)-β-D-glucosidase |
| 56448 | 0.000254 | 6.8206 | | 9.44317 | | 6.79795 | 1.015 up | | 6.255 up | | 6.158 up | GH18 chitinase CHI18-11 |
| 43873 | 0.00158 | 5.41039 | | 8.73865 | | 6.8787 | 2.766 down | | 3.629 up | | 10.043 up | GH18 chitinase CHI18-12 |
| 80833 | 0.0000735 | 9.15884 | | 12.92088 | | 8.11101 | 2.067 up | | 28.048 up | | 13.567 up | GH18 chitinase Chi18-5 |
| 2735 | 0.000275 | 7.09452 | | 10.23926 | | 6.49193 | 1.518 up | | 13.429 up | | 8.844 up | GH18 chitinase CHI18-6 |
| 65162 | 0.00024 | 10.93982 | | 13.51416 | | 8.59075 | 5.094 up | | 30.345 up | | 5.955 up | GH18 endo-N-acetyl-ß-D-glucosaminidase Endo T |
| 68347 | 0.00123 | 8.59614 | | 10.25667 | | 7.18654 | 2.656 up | | 8.398 up | | 3.161 up | GH18, chitinase CHI 18-16 |
| 119859 | 0.0111 | 9.81559 | | 10.52313 | | 8.9195 | 1.861 up | | 3.039 up | | 1.633 up | GH18, chitinase CHI18-13 |
| 124043 | 0.000763 | 3.51068 | | 10.65351 | | 4.44407 | 1.909 down | | 73.999 up | | 141.320 up | GH18, chitinase CHI18-14 |
| 59791 | 0.0000234 | 2.71721 | | 11.27662 | | 3.26277 | 1.459 down | | 258.470 up | | 377.259 up | GH18, chitinase CHI18-15 |
| 110317 | 0.00118 | 3.11463 | | 9.61069 | | 5.29524 | 4.533 down | | 19.910 up | | 90.262 up | GH18, chitinase CHI18-17 |
| 108346 | 0.015 | 3.45563 | | 5.67505 | | 3.63882 | 1.135 down | | 4.101 up | | 4.657 up | GH18, chitinase CHI18-8 |
| 5836 | 0.00019 | 10.7385 | | 13.46877 | | 6.23001 | 22.760 up | | 151.037 up | | 6.635 up | GH2 β-mannosidase |
| 77299 | 0.000191 | 8.93389 | | 10.80901 | | 4.84644 | 16.999 up | | 62.360 up | | 3.668 up | GH2 Exo-β-D-glucosaminidase GLS93 |
| 102909 | 0.00071 | 6.9365 | | 8.85629 | | 5.53527 | 2.641 up | | 9.993 up | | 3.783 up | GH2 protein |
| 76852 | 0.036 | 11.34123 | | 11.07009 | | 9.95828 | 2.608 up | | 2.161 up | | 1.206 down | GH2 β-galactosidase/β-glucuronidase |
| 57857 | 0.000134 | 12.25794 | | 11.09318 | | 9.20502 | 8.298 up | | 3.701 up | | 2.241 down | GH2 β-mannosidase |
| 69245 | 0.000147 | 11.63483 | | 13.69781 | | 4.97817 | 100.891 up | | 421.574 up | | 4.178 up | GH2 β-mannosidase |
| 59689 | 0.0116 | 9.4617 | | 10.04193 | | 8.65863 | 1.744 up | | 2.608 up | | 1.495 up | GH2 β-mannosidase |
| 62166 | 0.000402 | 9.83914 | | 12.49392 | | 9.96901 | 1.094 down | | 5.755 up | | 6.297 up | GH2 β-mannosidase |
| 21725 | 0.00056 | 10.56022 | | 13.30562 | | 10.29973 | 1.197 up | | 8.032 up | | 6.705 up | GH20 exochitinase |
| 105931 | 0.000145 | 7.93005 | | 10.25184 | | 6.57638 | 2.555 up | | 12.776 up | | 4.999 up | GH20 N-acetyl-β-hexosaminidase |
| 103458 | 0.000241 | 5.70013 | | 10.43616 | | 3.70654 | 3.982 up | | 106.125 up | | 26.649 up | GH25 N,O-diacetylmuramidase |
| 65986 | 0.000275 | 10.77344 | | 10.9252 | | 5.59597 | 36.188 up | | 40.203 up | | 1.110 up | GH27 α-galactosidase |
| 27259 | 0.00212 | 7.70757 | | 7.85831 | | 5.8964 | 3.509 up | | 3.895 up | | 1.110 up | GH27 α-galactosidase |
| 59391 | 0.000668 | 10.26214 | | 10.109 | | 6.03405 | 18.740 up | | 16.853 up | | 1.111 down | GH27 α-galactosidase |
| 55999 | 0.000371 | 8.14024 | | 10.52944 | | 5.67279 | 5.530 up | | 28.973 up | | 5.238 up | GH27 α-galactosidase |
| 75015 | 0.00134 | 8.82484 | | 10.04943 | | 7.98904 | 1.784 up | | 4.170 up | | 2.336 up | GH27 α-galactosidase |
| 72632 | 0.000133 | 11.69924 | | 10.74115 | | 4.8898 | 112.161 up | | 57.734 up | | 1.942 down | GH27 α-galactosidase AGL1 |
| 72704 | 0.000275 | 9.45526 | | 11.88418 | | 6.78629 | 6.359 up | | 34.246 up | | 5.384 up | GH27 α-galactosidase AGL3 |
| 112140 | 0.000247 | 4.07985 | | 11.0329 | | 3.30683 | 1.708 up | | 211.728 up | | 123.901 up | GH28 exo-polygalacturonase PGX1 |
| 103049 | 0.000507 | 5.31208 | | 12.34635 | | 3.26235 | 4.140 up | | 542.694 up | | 131.076 up | GH28 endo-polygalacturonase |
| 122780 | 0.0000419 | 11.30576 | | 14.58547 | | 4.8034 | 90.658 up | | 880.435 up | | 9.711 up | GH28 exo-rhamnogalacturonase RGX1 |
| 108671 | 0.000154 | 6.32718 | | 11.16466 | | 3.66328 | 6.337 up | | 181.192 up | | 28.590 up | GH3 β-glucosidase/glucan 1,4-β-glucosidase BGL3f |
| 76672 | 0.0000866 | 7.04683 | | 14.02627 | | 5.82184 | 2.337 up | | 294.971 up | | 126.188 up | GH3 β-glucosidase BGL1/CEL3a |
| 47268 | 0.000355 | 11.20929 | | 13.33961 | | 9.19478 | 4.040 up | | 17.689 up | | 4.378 up | GH3 β-glucosidase BGL3i |
| 104797 | 0.000242 | 11.01803 | | 12.63423 | | 6.94117 | 16.875 up | | 51.734 up | | 3.065 up | GH3 β-glucosidase BGL3j |
| 121735 | 0.000186 | 10.34505 | | 12.48465 | | 6.16037 | 18.185 up | | 80.130 up | | 4.406 up | GH3 β-glucosidase CEL3b |
| 82227 | 0.000115 | 11.71249 | | 13.59055 | | 8.41586 | 9.826 up | | 36.119 up | | 3.675 up | GH3 β-glucosidase CEL3c |
| 46816 | 0.000444 | 8.70765 | | 12.29279 | | 7.39709 | 2.480 up | | 29.768 up | | 12.001 up | GH3 β-glucosidase CEL3d |
| 121127 | 0.0000221 | 10.11473 | | 14.3935 | | 4.12773 | 63.426 up | | 1231.131 up | | 19.410 up | GH3 β-xylosidase BXL1 |
| 58450 | 0.000284 | 4.41688 | | 11.45142 | | 4.07991 | 1.263 up | | 165.594 up | | 131.100 up | GH3 β-xylosidase XYL3b |
| 110894 | 0.000237 | 7.00883 | | 11.91742 | | 7.4401 | 1.348 down | | 22.274 up | | 30.035 up | GH30 endo-β-1 6-galactanase |
| 69276 | 0.000101 | 7.51479 | | 14.41436 | | 5.3886 | 4.365 up | | 521.224 up | | 119.392 up | GH30 endo-β-1,4-xylanase |
| 111849 | 0.000141 | 9.07927 | | 14.01818 | | 5.04766 | 16.354 up | | 501.644 up | | 30.673 up | GH30 endo-β-1,4-xylanase XYN4 |
| 69944 | 0.000127 | 6.23821 | | 12.45085 | | 4.19413 | 4.124 up | | 305.858 up | | 74.163 up | GH31 α-xylosidase/α-glucosidase |
| 82235 | 0.000264 | 12.75967 | | 14.24266 | | 11.52462 | 2.353 up | | 6.579 up | | 2.795 up | GH31 α-glucosidase |
| 60085 | 0.000105 | 9.13304 | | 10.79178 | | 6.99162 | 4.411 up | | 13.930 up | | 3.157 up | GH31 α-glucosidase |
| 80240 | 0.000805 | 12.33175 | | 13.4877 | | 10.84565 | 2.801 up | | 6.242 up | | 2.228 up | GH35 β-galactosidase BGA1 |
| 64827 | 0.000397 | 11.92297 | | 11.34804 | | 10.31306 | 3.052 up | | 2.049 up | | 1.489 down | GH36 raffinose synthase domain protein |
| 124016 | 0.000518 | 11.01465 | | 11.92123 | | 7.2467 | 13.622 up | | 25.537 up | | 1.874 up | GH36 α-galactosidase AGL2 |
| 123226 | 0.000201 | 12.64496 | | 11.19531 | | 7.94836 | 25.930 up | | 9.493 up | | 2.731 down | GH37 α,α-trehalase |
| 3196 | 0.00239 | 12.46691 | | 12.83577 | | 10.89232 | 2.978 up | | 3.846 up | | 1.291 up | GH38 α-mannosidase |
| 3739 | 0.0000284 | 12.45843 | | 12.96967 | | 4.78311 | 204.410 up | | 291.341 up | | 1.425 up | GH43 β-xylosidase/α-L-arabinofuranosidase |
| 68064 | 0.00434 | 11.64029 | | 13.06745 | | 11.62863 | 1.008 up | | 2.710 up | | 2.689 up | GH43 β-xylosidase/α-L-arabinofuranosidase |
| 45717 | 0.000251 | 13.08266 | | 12.45059 | | 9.69935 | 10.434 up | | 6.732 up | | 1.549 down | GH47 α-1,2-mannosidase |
| 79960 | 0.000247 | 10.74907 | | 13.36149 | | 9.91397 | 1.783 up | | 10.909 up | | 6.115 up | GH47 α-1,2-mannosidase |
| 65380 | 0.00053 | 10.51249 | | 12.49731 | | 10.2765 | 1.177 up | | 4.661 up | | 3.958 up | GH47 α-1,2-mannosidase |
| 120312 | 0.0000818 | 10.85671 | | 14.75786 | | 3.69506 | 143.176 up | | 2139.119 up | | 14.940 up | GH5 endo-β-1,4-glucanase EGL2/CEL5a |
| 49976 | 0.000115 | 8.40415 | | 13.63375 | | 5.25985 | 8.841 up | | 331.737 up | | 37.520 up | GH5 endo-β-1,4-glucanase EGL5/CEL45a |
| 56996 | 0.000105 | 4.92696 | | 13.75252 | | 4.57311 | 1.277 up | | 579.801 up | | 453.688 up | GH5 β-Mannanase MAN1 |
| 55319 | 0.000133 | 12.47242 | | 14.25266 | | 10.67762 | 3.469 up | | 11.917 up | | 3.434 up | GH54 α-L-arabinofuranosidase ABF3 |
| 123283 | 0.0000537 | 8.87162 | | 13.88212 | | 6.65586 | 4.645 up | | 149.734 up | | 32.233 up | GH54 α-L-arabinofuranosidase I ABF1 |
| 121746 | 0.000245 | 10.82838 | | 13.77442 | | 8.64557 | 4.540 up | | 34.989 up | | 7.706 up | GH55 exo-1 3-β-glucanase GLUC78 |
| 73248 | 0.00808 | 7.95614 | | 8.82287 | | 6.9459 | 2.014 up | | 3.673 up | | 1.823 up | GH55 exo-1,3-β-glucanase |
| 56418 | 0.000958 | 6.61051 | | 8.32366 | | 5.69651 | 1.884 up | | 6.178 up | | 3.278 up | GH55 β-1 3-glucanase |
| 54242 | 0.000448 | 7.92633 | | 12.78898 | | 11.41913 | 11.257 down | | 2.584 up | | 29.094 up | GH55 β-1,3-glucanase |
| 70845 | 0.000203 | 9.03833 | | 12.91771 | | 9.53681 | 1.412 down | | 10.417 up | | 14.716 up | GH55 β-1,3-glucanase |
| 108776 | 0.00354 | 5.42228 | | 6.67498 | | 5.33171 | 1.064 up | | 2.537 up | | 2.382 up | GH55 β-1,3-glucanase |
| 72567 | 0.0000691 | 12.99037 | | 15.41537 | | 4.23375 | 432.517 up | | 2322.747 up | | 5.370 up | GH6 Cellobiohydrolase CEL6A/CBH2 |
| 22129 | 0.000179 | 10.91782 | | 13.04618 | | 10.08046 | 1.786 up | | 7.812 up | | 4.372 up | GH61 polysaccharide monooxygenase |
| 73643 | 0.0000483 | 7.80786 | | 13.74156 | | 3.88722 | 15.143 up | | 925.664 up | | 61.125 up | GH61 polysaccharide monooxygenase CEL61a |
| 120961 | 0.0000401 | 8.6541 | | 13.80823 | | 2.93212 | 52.781 up | | 1879.465 up | | 35.608 up | GH61 polysaccharide monooxygenase CEL61b |
| 76210 | 0.000085 | 7.01599 | | 14.29814 | | 3.5917 | 10.735 up | | 1670.930 up | | 155.648 up | GH62 α-L-arabinofuranosidase ABF2 |
| 65137 | 0.00675 | 6.48053 | | 4.30623 | | 5.06718 | 2.663 up | | 1.694 down | | 4.513 down | GH64 endo-1,3-β-glucanase |
| 124175 | 0.000392 | 8.33511 | | 12.30438 | | 7.42568 | 1.878 up | | 29.419 up | | 15.662 up | GH64 endo-1,3-β-glucanase |
| 25224 | 0.000405 | 10.00016 | | 9.61454 | | 7.32187 | 6.400 up | | 4.899 up | | 1.306 down | GH65 α,α-trehalase |
| 123456 | 0.000725 | 12.62993 | | 13.11065 | | 8.96564 | 12.678 up | | 17.691 up | | 1.395 up | GH65 α,α-trehalase |
| 72526 | 0.0000499 | 11.59499 | | 14.17817 | | 3.74505 | 230.709 up | | 1382.556 up | | 5.992 up | GH67 α-Glucuronidase GLR1 |
| 123989 | 0.000104 | 14.27507 | | 15.22762 | | 6.8257 | 174.776 up | | 338.244 up | | 1.935 up | GH7 Cellobiohydrolase CBH1/CEL7a |
| 122081 | 0.00011 | 8.68794 | | 14.4052 | | 6.87889 | 3.504 up | | 184.351 up | | 52.609 up | GH7 Endo-β-1,4-glucanase EGL1/CEL7b |
| 71532 | 0.000213 | 12.29357 | | 12.46034 | | 10.84025 | 2.738 up | | 3.073 up | | 1.122 up | GH71 α-1 3-glucanase |
| 120873 | 0.000146 | 4.82384 | | 12.30627 | | 4.03084 | 1.732 up | | 309.850 up | | 178.827 up | GH71 α-1 3-glucanase |
| 73179 | 0.000113 | 6.98958 | | 11.6502 | | 7.69051 | 1.625 down | | 15.559 up | | 25.292 up | GH71 α-1 3-glucanase |
| 78713 | 0.00153 | 11.26503 | | 10.80999 | | 9.8967 | 2.581 up | | 1.883 up | | 1.370 down | GH72 ß-(1-3) glucanosyltransferase |
| 123538 | 0.000369 | 12.83701 | | 13.93046 | | 11.01195 | 3.543 up | | 7.560 up | | 2.133 up | GH72 β-1 3-glucanosyltransferase |
| 49081 | 0.000125 | 8.72972 | | 13.7031 | | 6.09667 | 6.203 up | | 194.879 up | | 31.414 up | GH74 Xyloglucanase CEL74a |
| 70341 | 0.000263 | 8.33458 | | 10.61515 | | 7.2808 | 2.075 up | | 10.086 up | | 4.858 up | GH75 chitosanase |
| 42152 | 0.000596 | 8.70845 | | 8.05093 | | 7.11822 | 3.010 up | | 1.908 up | | 1.577 down | GH75 chitosanase |
| 74807 | 0.000198 | 12.642 | | 13.81748 | | 10.89094 | 3.366 up | | 7.602 up | | 2.258 up | GH76 GPI-anchored α-1,6-mannanase |
| 122495 | 0.000248 | 10.80902 | | 7.87421 | | 6.38527 | 21.462 up | | 2.806 up | | 7.646 down | GH76 α-1,6-mannanase |
| 27395 | 0.000154 | 9.07138 | | 13.51352 | | 6.99798 | 4.208 up | | 91.489 up | | 21.737 up | GH76 α-1,6-mannanase |
| 55802 | 0.000429 | 8.68349 | | 10.79197 | | 7.3465 | 2.526 up | | 10.894 up | | 4.312 up | GH76 α-1,6-mannanase |
| 69123 | 0.000343 | 12.09088 | | 13.63083 | | 11.4008 | 1.613 up | | 4.691 up | | 2.907 up | GH76 α-1,6-mannanase |
| 58887 | 0.016 | 3.37525 | | 5.86647 | | 3.83511 | 1.375 down | | 4.087 up | | 5.622 up | GH78 α-L-rhamnosidase |
| 106575 | 0.000325 | 10.01779 | | 10.44564 | | 7.83538 | 4.539 up | | 6.106 up | | 1.345 up | GH79 β-glucuronidase |
| 73256 | 0.00105 | 5.12995 | | 7.99136 | | 5.04548 | 1.060 up | | 7.705 up | | 7.267 up | GH81 endo-1,3-β-glucanase |
| 69700 | 0.00241 | 6.69319 | | 7.34341 | | 5.84388 | 1.801 up | | 2.827 up | | 1.569 up | GH89 α-N-acetylglucosaminidase |
| 79921 | 0.000149 | 12.94634 | | 14.10558 | | 10.1851 | 6.779 up | | 15.141 up | | 2.233 up | GH92 α-1,2-mannosidase |
| 74198 | 0.000253 | 10.81316 | | 13.10545 | | 6.08352 | 26.531 up | | 129.960 up | | 4.898 up | GH92 α-1,2-mannosidase |
| 60635 | 0.000173 | 5.36203 | | 13.0222 | | 6.67168 | 2.478 down | | 81.601 up | | 202.274 up | GH92 α-1,2-mannosidase |
| 69493 | 0.00336 | 4.7009 | | 6.85669 | | 3.97369 | 1.655 up | | 7.376 up | | 4.456 up | GH92 α-1,2-mannosidase |
| 55733 | 0.000212 | 9.34945 | | 13.58835 | | 9.83744 | 1.402 down | | 13.462 up | | 18.881 up | GH92 α-1,2-mannosidase |
| 58802 | 0.000239 | 9.29927 | | 9.58669 | | 5.72465 | 11.914 up | | 14.540 up | | 1.220 up | GH95 α-L-fucosidase |
| 72488 | 0.000259 | 9.32376 | | 9.69858 | | 6.70929 | 6.123 up | | 7.940 up | | 1.296 up | GH95 α-L-fucosidase |
| 5807 | 0.000105 | 8.79103 | | 10.5242 | | 4.01602 | 27.379 up | | 91.023 up | | 3.324 up | GH95 α-L-fucosidase |
| 123726 | 0.000324 | 7.3482 | | 12.56885 | | 8.12287 | 1.710 down | | 21.795 up | | 37.288 up | glucan endo-1,3(4)-β-D-glucosidase |
| 71072 | 0.000435 | 7.07446 | | 7.58764 | | 3.11341 | 15.573 up | | 22.226 up | | 1.427 up | Gluconate kinase |
| 56934 | 0.0013 | 12.54708 | | 13.69294 | | 11.60696 | 1.918 up | | 4.245 up | | 2.212 up | gluconokinase, thermoresistant glucokinase family |
| 49898 | 0.00107 | 11.17859 | | 12.877 | | 9.54898 | 3.094 up | | 10.042 up | | 3.245 up | glucosamine-6-phosphate isomerase |
| 22915 | 0.000763 | 10.75251 | | 12.70338 | | 9.28437 | 2.766 up | | 10.696 up | | 3.866 up | glucose oxidase |
| 65275 | 0.000316 | 7.91749 | | 10.89018 | | 7.23388 | 1.606 up | | 12.608 up | | 7.850 up | Glucose/sorbosone dehydrogenases |
| 81778 | 0.000457 | 13.29119 | | 13.48219 | | 10.28048 | 8.059 up | | 9.200 up | | 1.141 up | glutaminase A |
| 123009 | 0.000145 | 12.52746 | | 13.26693 | | 7.46728 | 33.363 up | | 55.701 up | | 1.669 up | glutamine synthetase |
| 103487 | 0.00124 | 8.89859 | | 10.23968 | | 8.76576 | 1.096 up | | 2.777 up | | 2.533 up | glutathione S transferase |
| 49946 | 0.00025 | 10.71254 | | 12.42556 | | 9.1955 | 2.862 up | | 9.383 up | | 3.278 up | Glutathione S-transferase |
| 58026 | 0.00199 | 10.52899 | | 10.86459 | | 9.31885 | 2.313 up | | 2.919 up | | 1.261 up | glutathione S-transferase domain-containing protein |
| 107554 | 0.0171 | 11.58166 | | 11.54331 | | 10.04948 | 2.892 up | | 2.816 up | | 1.026 down | glutathione transferase |
| 64172 | 0.000764 | 8.33124 | | 11.02246 | | 7.72454 | 1.522 up | | 9.834 up | | 6.458 up | Glutathione-dependent formaldehyde-activating,GFA |
| 112022 | 0.000165 | 10.13941 | | 7.76785 | | 3.4324 | 104.474 up | | 20.188 up | | 5.174 down | glutathione-S-transferase |
| 56819 | 0.000243 | 12.27927 | | 11.09492 | | 10.29918 | 3.945 up | | 1.735 up | | 2.272 down | glutathione-S-transferase |
| 58356 | 0.000198 | 13.65094 | | 14.19365 | | 11.23376 | 5.341 up | | 7.780 up | | 1.456 up | glycerol kinase |
| 58790 | 0.000255 | 11.52577 | | 13.81079 | | 11.62183 | 1.068 down | | 4.559 up | | 4.873 up | glycerol-3-phosphate phosphatase, putative |
| 68466 | 0.000177 | 4.50724 | | 10.09109 | | 4.8192 | 1.241 down | | 38.636 up | | 47.962 up | glycerone kinase |
| 48603 | 0.00369 | 11.92477 | | 12.24094 | | 10.28878 | 3.108 up | | 3.869 up | | 1.245 up | glycerophosphoryl diester phosphodiesterase family protein |
| 44366 | 0.000258 | 9.94174 | | 13.57929 | | 8.94196 | 1.999 up | | 24.887 up | | 12.445 up | Glycosylphosphatidylinositol-specific phospholipase C |
| 69926 | 0.00779 | 5.4439 | | 6.19711 | | 3.93754 | 2.840 up | | 4.788 up | | 1.685 up | glycosyltransferases, Glycosyltransferases not yet assigned to a family |
| 123978 | 0.000145 | 13.46012 | | 13.20571 | | 8.78318 | 25.579 up | | 21.444 up | | 1.192 down | GMC methanol oxidase |
| 120008 | 0.000763 | 12.11231 | | 14.02231 | | 10.74275 | 2.583 up | | 9.710 up | | 3.758 up | GMC oxidoreductase |
| 105851 | 0.000855 | 7.73426 | | 8.25081 | | 4.95334 | 6.872 up | | 9.831 up | | 1.430 up | GMC oxidoreductase family protein |
| 67494 | 0.000108 | 14.03986 | | 10.82178 | | 9.69165 | 20.367 up | | 2.188 up | | 9.305 down | Golgi GDP-mannose transporter |
| 54669 | 0.0273 | 4.89285 | | 8.6363 | | 6.40202 | 2.846 down | | 4.705 up | | 13.393 up | Golgi matrix protein, rud3, involved in the structural organization of the cis-Golgi |
| 63981 | 0.000183 | 11.82224 | | 13.1258 | | 7.87333 | 15.443 up | | 38.119 up | | 2.468 up | GPCR , contains RGS domain |
| 81383 | 0.000285 | 11.61794 | | 13.8421 | | 8.56092 | 8.322 up | | 38.886 up | | 4.672 up | GPCR , contains RGS domain |
| 37525 | 0.000211 | 10.93722 | | 13.50157 | | 5.99468 | 30.750 up | | 181.885 up | | 5.914 up | GPCR , contains RGS domain |
| 57526 | 0.000372 | 9.64718 | | 7.04667 | | 5.33858 | 19.816 up | | 3.267 up | | 6.065 down | GPCR, mating type pheromone G-protein coupled receptor |
| 123806 | 0.000183 | 11.97527 | | 10.43208 | | 6.06065 | 60.322 up | | 20.698 up | | 2.914 down | GPCR, secretin like |
| 72627 | 0.000733 | 9.44481 | | 7.15745 | | 5.22501 | 18.633 up | | 3.816 up | | 4.881 down | GPCR, secretin like |
| 64018 | 0.000352 | 13.00421 | | 11.9798 | | 9.03467 | 15.665 up | | 7.701 up | | 2.034 down | GPCR, mating type pheromone G-protein coupled receptor |
| 41035 | 0.000569 | 13.21193 | | 14.30984 | | 12.08213 | 2.188 up | | 4.683 up | | 2.140 up | G-protein coupled receptor protein |
| 73516 | 0.00336 | 14.59241 | | 11.87801 | | 12.66002 | 3.816 up | | 1.719 down | | 6.563 down | Grg1 Glucose repressible protein |
| 78988 | 0.000846 | 12.57929 | | 13.43871 | | 11.54495 | 2.048 up | | 3.716 up | | 1.814 up | Gß-WD40 domain protein |
| 70096 | 0.000133 | 9.50838 | | 11.63421 | | 8.37183 | 2.198 up | | 9.595 up | | 4.364 up | Gß-WD40 domain protein |
| 80528 | 0.0022 | 10.64426 | | 13.10682 | | 10.47462 | 1.124 up | | 6.199 up | | 5.511 up | Gß-WD40 protein |
| 77557 | 0.000474 | 11.26662 | | 10.76519 | | 9.61464 | 3.142 up | | 2.219 up | | 1.415 down | GT glycosyltransferases not yet assigned to a family, 3 TMs |
| 80340 | 0.000211 | 9.93872 | | 13.00943 | | 6.20349 | 13.317 up | | 111.890 up | | 8.401 up | GT α-1,6-mannosyltransferase |
| 63828 | 0.00425 | 11.26736 | | 12.25688 | | 10.44453 | 1.768 up | | 3.512 up | | 1.985 up | GT α-1,6-mannosyltransferase |
| 105924 | 0.00062 | 7.88453 | | 10.8669 | | 6.79939 | 2.121 up | | 16.766 up | | 7.902 up | GT1 ß-glycosyltransferase |
| 56328 | 0.00278 | 6.24448 | | 4.3985 | | 3.99504 | 4.754 up | | 1.322 up | | 3.594 down | GT31 b-glycosyltransferases |
| 76151 | 0.00056 | 10.39958 | | 12.48773 | | 9.74015 | 1.579 up | | 6.715 up | | 4.252 up | GT31 ß-glycosyltransferase |
| 64925 | 0.00105 | 11.34773 | | 13.62968 | | 11.83958 | 1.406 down | | 3.458 up | | 4.863 up | GT32 a-glycosyltransferase |
| 68470 | 0.000336 | 6.0281 | | 9.99982 | | 7.05122 | 2.032 down | | 7.720 up | | 15.689 up | GT4 glycosyl transferase |
| 65982 | 0.00297 | 12.1624 | | 13.20694 | | 11.5477 | 1.531 up | | 3.158 up | | 2.062 up | GTPase activating protein for RabGTPases (Ras superfamily of smallGTPases) |
| 76880 | 0.000728 | 11.69288 | | 13.58579 | | 11.63788 | 1.038 up | | 3.858 up | | 3.713 up | GTPase Rsr1 (A. fumigatus) |
| 44251 | 0.000466 | 9.95618 | | 12.65222 | | 9.80397 | 1.111 up | | 7.201 up | | 6.480 up | GTPase-activating protein Msb3 (Sec4/Rabs) |
| 110525 | 0.000306 | 9.17487 | | 11.33865 | | 9.46611 | 1.223 down | | 3.661 up | | 4.480 up | GTP-binding protein, HSR1-related |
| 50583 | 0.000885 | 10.24887 | | 11.1137 | | 9.17575 | 2.103 up | | 3.831 up | | 1.821 up | Guanine deaminase (GDEase) |
| 107005 | 0.00145 | 10.61696 | | 12.54205 | | 11.07746 | 1.376 down | | 2.759 up | | 3.797 up | Guanine nucleotide exchange factor |
| 34726 | 0.000907 | 4.49885 | | 7.5346 | | 4.92405 | 1.342 down | | 6.107 up | | 8.200 up | Guanine nucleotide exchange factor for Ras-likeGTPases (RasGEF) |
| 54408 | 0.00165 | 10.54096 | | 11.70943 | | 10.28272 | 1.196 up | | 2.688 up | | 2.247 up | guanylate kinase, putative |
| 59542 | 0.00205 | 9.93599 | | 12.72835 | | 11.16242 | 2.339 down | | 2.960 up | | 6.927 up | Guanyl-nucleotide exchange factor Sec2 |
| 54846 | 0.00139 | 9.25985 | | 10.28614 | | 8.38404 | 1.835 up | | 3.737 up | | 2.036 up | half-sized ABC transporter |
| 58068 | 0.000403 | 10.17077 | | 11.21803 | | 9.0787 | 2.131 up | | 4.405 up | | 2.066 up | haloacid dehalogenase-like hydrolase |
| 22164 | 0.000464 | 11.20922 | | 13.07821 | | 11.19184 | 1.012 up | | 3.697 up | | 3.652 up | heat shock protein, Hsp40, DnaJ |
| 54441 | 0.000926 | 10.76841 | | 12.64873 | | 10.53542 | 1.175 up | | 4.326 up | | 3.681 up | HECT-domain-containing protein |
| 63756 | 0.000806 | 9.81552 | | 10.26353 | | 8.09782 | 3.289 up | | 4.486 up | | 1.364 up | helicase, DEAD-box superfamily |
| 41664 | 0.00635 | 8.01261 | | 9.67155 | | 7.85152 | 1.118 up | | 3.530 up | | 3.157 up | HET domain-containing protein |
| 41699 | 0.000156 | 6.5596 | | 9.27903 | | 5.49888 | 2.085 up | | 13.738 up | | 6.586 up | HET protein |
| 112083 | 0.000192 | 6.95889 | | 10.81867 | | 9.34361 | 5.222 down | | 2.779 up | | 14.518 up | HET protein |
| 106171 | 0.00164 | 10.22983 | | 12.48616 | | 10.90294 | 1.594 down | | 2.996 up | | 4.777 up | HET protein |
| 112496 | 0.00219 | 9.59633 | | 11.18568 | | 8.95507 | 1.559 up | | 4.693 up | | 3.009 up | heterocompatibility domain protein |
| 123499 | 0.000198 | 10.88086 | | 12.97057 | | 10.90735 | 1.018 down | | 4.179 up | | 4.256 up | HET-s/LopB domain protein |
| 121850 | 0.000229 | 11.41347 | | 13.2151 | | 11.05628 | 1.280 up | | 4.465 up | | 3.486 up | hexose transporter |
| 119989 | 0.000251 | 13.79836 | | 14.41285 | | 7.14368 | 100.752 up | | 154.253 up | | 1.531 up | HFB2 |
| 123967 | 0.000145 | 13.79163 | | 14.34234 | | 4.20263 | 770.153 up | | 1128.125 up | | 1.464 up | HFB3 |
| 106538 | 0.000722 | 9.50594 | | 12.97342 | | 4.34021 | 35.895 up | | 397.060 up | | 11.061 up | HFB4 |
| 105763 | 0.00797 | 10.20321 | | 10.61976 | | 8.96407 | 2.360 up | | 3.150 up | | 1.334 up | HFBs |
| 123039 | 0.000558 | 12.74451 | | 13.1628 | | 11.52171 | 2.333 up | | 3.119 up | | 1.336 up | HFBs |
| 119805 | 0.00102 | 13.13625 | | 14.46644 | | 11.73972 | 2.632 up | | 6.619 up | | 2.514 up | HFBs |
| 104401 | 0.000133 | 11.79163 | | 13.45671 | | 10.87676 | 1.885 up | | 5.979 up | | 3.171 up | HFBs |
| 119963 | 0.001 | 12.77424 | | 14.16168 | | 12.18244 | 1.507 up | | 3.942 up | | 2.616 up | HFBs |
| 77739 | 0.000327 | 10.66311 | | 12.09956 | | 10.50175 | 1.118 up | | 3.026 up | | 2.706 up | HgmAhomogentisate ,-dioxygenase |
| 30578 | 0.000322 | 9.19603 | | 8.30782 | | 6.83159 | 5.149 up | | 2.782 up | | 1.850 down | HhH-GPD family base excision DNA repair protein |
| 68072 | 0.00049 | 9.38162 | | 10.76129 | | 8.66778 | 1.640 up | | 4.267 up | | 2.602 up | HhH-GPD superfamily base excision DNA repair protein |
| 77764 | 0.00307 | 11.94111 | | 13.05137 | | 11.62909 | 1.241 up | | 2.680 up | | 2.158 up | histidine kinase PHY1p, phytochrome-like |
| 59384 | 0.000542 | 8.36968 | | 10.65801 | | 8.45955 | 1.064 down | | 4.589 up | | 4.884 up | histidine kinase class I, M27Mp |
| 62751 | 0.000663 | 11.56425 | | 13.465 | | 10.77718 | 1.725 up | | 6.443 up | | 3.734 up | histidine kinase HHK6 |
| 46708 | 0.000797 | 12.32252 | | 13.92152 | | 12.39324 | 1.050 down | | 2.884 up | | 3.029 up | histidine kinase Nik1 |
| 70943 | 0.000211 | 10.4817 | | 12.15442 | | 9.83734 | 1.563 up | | 4.983 up | | 3.188 up | Histidine kinase, part of a two component signal transduction system |
| 109040 | 0.0105 | 10.54031 | | 12.11141 | | 10.45345 | 1.062 up | | 3.155 up | | 2.971 up | Histidine kinase, part of a two-component signal transduction system |
| 120339 | 0.00135 | 9.36651 | | 11.18276 | | 8.28526 | 2.115 up | | 7.451 up | | 3.521 up | Histone acetyltransferase (MYST family) |
| 110943 | 0.000212 | 9.47219 | | 10.07573 | | 8.11308 | 2.565 up | | 3.897 up | | 1.519 up | Histone acetyltransferase SAGA/ADA, catalytic subunit PCAF/GCN5 and related proteins |
| 64680 | 0.00187 | 11.72124 | | 12.83841 | | 11.48169 | 1.180 up | | 2.561 up | | 2.169 up | Histone acetyltransferase, catalytic subunit of the ADA and SAGA complexes |
| 47838 | 0.00169 | 11.48344 | | 13.17272 | | 11.68651 | 1.151 down | | 2.801 up | | 3.224 up | histone chaperone ASF1 |
| 80797 | 0.00116 | 10.3936 | | 12.03337 | | 10.43951 | 1.032 down | | 3.018 up | | 3.116 up | histone deacetylase |
| 48386 | 0.0102 | 11.41528 | | 12.42105 | | 10.96974 | 1.361 up | | 2.734 up | | 2.008 up | Histone deacetylase; regulates transcription and silencing |
| 80732 | 0.000769 | 10.51147 | | 12.37923 | | 10.92886 | 1.335 down | | 2.732 up | | 3.649 up | histone H3 lysine 36 (K36) methyltransferase |
| 111216 | 0.000697 | 10.40015 | | 12.12761 | | 10.45759 | 1.040 down | | 3.182 up | | 3.311 up | histone H3 methyltransferase |
| 122500 | 0.00182 | 11.3727 | | 12.02758 | | 10.69383 | 1.600 up | | 2.520 up | | 1.574 up | Histone tail methylase containing SET domain |
| 32755 | 0.000145 | 6.8076 | | 8.91466 | | 7.15275 | 1.270 down | | 3.391 up | | 4.308 up | Histone tail methylase containing SET domain |
| 121074 | 0.00199 | 11.3423 | | 12.24937 | | 10.52369 | 1.763 up | | 3.307 up | | 1.875 up | homeobox transcriptional regulator |
| 40758 | 0.000859 | 10.80523 | | 12.21288 | | 10.52425 | 1.215 up | | 3.223 up | | 2.653 up | Homocysteine/selenocysteine methylase (S-methylmethionine-dependent) |
| 70630 | 0.000211 | 13.50308 | | 13.38871 | | 11.48136 | 4.060 up | | 3.751 up | | 1.082 down | homoserine acetyltransferase family protein |
| 122363 | 0.000212 | 8.90643 | | 11.17665 | | 9.41822 | 1.425 down | | 3.383 up | | 4.823 up | Hsp26/Hsp42 |
| 121839 | 0.000941 | 12.54044 | | 12.89382 | | 11.24356 | 2.456 up | | 3.138 up | | 1.277 up | HSP70 |
| 60450 | 0.000108 | 9.64568 | | 11.66498 | | 9.27137 | 1.296 up | | 5.254 up | | 4.053 up | Hus1 protein of Schizosaccharomyces pombe and mammals, a component of the 9-1-1 replication checkpoint clamp |
| 3824 | 0.000496 | 11.03363 | | 13.15235 | | 11.16958 | 1.098 down | | 3.952 up | | 4.343 up | HVA22 domain membrane protein, pathogenicity related |
| 61403 | 0.000307 | 9.90674 | | 11.27744 | | 8.66096 | 2.371 up | | 6.132 up | | 2.585 up | Hydantoinase/oxoprolinase |
| 67639 | 0.000241 | 13.7421 | | 14.12319 | | 11.00157 | 6.683 up | | 8.703 up | | 1.302 up | IlvB, Thiamine pyrophosphate-requiring enzymes |
| 110757 | 0.000308 | 9.78844 | | 9.40117 | | 5.64111 | 17.720 up | | 13.548 up | | 1.307 down | imidazole proprionase-related amidohydrolase |
| 2583 | 0.000247 | 6.40644 | | 9.91989 | | 7.103 | 1.620 down | | 7.046 up | | 11.419 up | Imidazoleglycerol-phosphate synthase subunit H |
| 64009 | 0.00197 | 11.01713 | | 12.44637 | | 10.82627 | 1.141 up | | 3.073 up | | 2.693 up | importin ß KapH |
| 3372 | 0.000244 | 11.21309 | | 12.58222 | | 10.06 | 2.223 up | | 5.744 up | | 2.583 up | IMP-specific 5'-nucleotidase |
| 123550 | 0.000575 | 11.56872 | | 11.8007 | | 9.7237 | 3.592 up | | 4.219 up | | 1.174 up | Indoleamine 2,3-dioxygenase-like protein |
| 111131 | 0.000805 | 9.22548 | | 11.94947 | | 9.96984 | 1.675 down | | 3.943 up | | 6.606 up | initiation factor 2B. |
| 70076 | 0.003 | 11.43092 | | 12.30496 | | 10.59553 | 1.784 up | | 3.270 up | | 1.832 up | Inositol polyphosphate kinase, putative |
| 102612 | 0.000392 | 11.1886 | | 12.83805 | | 10.73994 | 1.364 up | | 4.281 up | | 3.137 up | inositol-pentakisphosphate 2-kinase |
| 63914 | 0.00204 | 12.17139 | | 12.54637 | | 10.86418 | 2.474 up | | 3.209 up | | 1.296 up | integral membrane protein, putative |
| 82499 | 0.00219 | 12.21192 | | 13.50043 | | 11.83333 | 1.300 up | | 3.175 up | | 2.442 up | Integral membrane protein, putative |
| 68026 | 0.00526 | 10.57178 | | 12.18589 | | 9.54401 | 2.038 up | | 6.241 up | | 3.061 up | integral peroxisomal membrane peroxin, putative |
| 71094 | 0.000159 | 8.57775 | | 9.71331 | | 5.92515 | 6.287 up | | 13.814 up | | 2.197 up | Intradiol ring-cleavage dioxygenase |
| 56440 | 0.000538 | 12.89382 | | 13.46623 | | 12.13216 | 1.695 up | | 2.521 up | | 1.487 up | Ion transport protein |
| 38812 | 0.0000787 | 14.5249 | | 14.59321 | | 7.36673 | 142.830 up | | 149.756 up | | 1.048 up | iron transporter |
| 54962 | 0.000356 | 8.27768 | | 10.95955 | | 6.66394 | 3.060 up | | 19.638 up | | 6.416 up | iron transporter |
| 80639 | 0.00126 | 3.53975 | | 9.90117 | | 8.57574 | 32.808 down | | 2.506 up | | 82.220 up | iron transporter |
| 76034 | 0.00149 | 12.10606 | | 11.89055 | | 10.05773 | 4.136 up | | 3.562 up | | 1.161 down | Iron/ascorbate family oxidoreductases |
| 105968 | 0.00932 | 10.53962 | | 12.42088 | | 10.47496 | 1.045 up | | 3.852 up | | 3.683 up | Iron/ascorbate family oxidoreductases |
| 104606 | 0.000318 | 12.24917 | | 13.05365 | | 10.82561 | 2.682 up | | 4.684 up | | 1.746 up | Iron-containing alcohol dehydrogenase |
| 5182 | 0.0014 | 12.57685 | | 11.87597 | | 10.11859 | 5.495 up | | 3.380 up | | 1.625 down | iron-dependent peroxidase |
| 75985 | 0.000503 | 11.16105 | | 12.58688 | | 9.26543 | 3.720 up | | 9.996 up | | 2.686 up | Isochorismatase hydrolase |
| 21758 | 0.00036 | 14.86158 | | 14.67556 | | 12.00539 | 7.240 up | | 6.365 up | | 1.137 down | isocitrate lyase |
| 67772 | 0.0191 | 9.69673 | | 10.96925 | | 8.83078 | 1.822 up | | 4.402 up | | 2.415 up | Isoflavone reductase |
| 31134 | 0.000217 | 12.63509 | | 10.89295 | | 9.80019 | 7.134 up | | 2.132 up | | 3.345 down | isoprenylcysteine carboxyl methyltransferase |
| 79169 | 0.00037 | 12.51591 | | 13.31151 | | 11.48905 | 2.037 up | | 3.536 up | | 1.735 up | K+ channel protein |
| 1997 | 0.00158 | 8.90862 | | 11.08827 | | 9.27768 | 1.291 down | | 3.507 up | | 4.530 up | kinesin |
| 102378 | 0.00123 | 10.54477 | | 12.43628 | | 10.68713 | 1.103 down | | 3.361 up | | 3.710 up | kinesin-like protein, a kinesin-3 family member. |
| 44628 | 0.000776 | 11.30444 | | 12.67239 | | 11.18205 | 1.088 up | | 2.809 up | | 2.581 up | Kynureninase |
| 104418 | 0.00155 | 7.71354 | | 8.43008 | | 6.83978 | 1.832 up | | 3.011 up | | 1.643 up | Kynurenine 3-monooxygenase and related flavoprotein monooxygenases |
| 41617 | 0.000325 | 11.81855 | | 13.63062 | | 11.60573 | 1.158 up | | 4.069 up | | 3.511 up | lae1 |
| 49753 | 0.000396 | 11.048 | | 12.17684 | | 7.91808 | 8.753 up | | 19.143 up | | 2.186 up | L-arabinitol 4-dehydrogenase |
| 55240 | 0.00175 | 11.61429 | | 9.36125 | | 9.88115 | 3.324 up | | 1.433 down | | 4.766 down | large-conductance mechanosensitive channel |
| 122708 | 0.000604 | 10.28022 | | 11.91701 | | 9.96493 | 1.244 up | | 3.869 up | | 3.109 up | Late Golgi protein sorting complex, subunit Vps53 |
| 78462 | 0.0000703 | 8.45209 | | 11.58238 | | 5.1265 | 10.025 up | | 87.783 up | | 8.756 up | L-carnitine dehydratase/alpha-methylacyl-CoA racemase |
| 56470 | 0.00077 | 13.45077 | | 12.33805 | | 11.48324 | 3.910 up | | 1.808 up | | 2.162 down | LdhA Lactate dehydrogenase and related dehydrogenases |
| 56920 | 0.00176 | 13.0454 | | 13.94201 | | 12.58807 | 1.372 up | | 2.556 up | | 1.861 up | Leucine aminopeptidase 1 |
| 53468 | 0.00332 | 9.92727 | | 11.13533 | | 9.68451 | 1.183 up | | 2.733 up | | 2.310 up | Lif1p of Saccharomyces cerevisiae |
| 53569 | 0.0124 | 9.08357 | | 10.97671 | | 9.21274 | 1.093 down | | 3.396 up | | 3.714 up | light-regulation protein, related to Drosophila TIMELESS |
| 121418 | 0.0000425 | 9.10003 | | 14.04199 | | 3.53882 | 47.216 up | | 1451.343 up | | 30.738 up | lipase G-D-S-L |
| 119696 | 0.00095 | 7.38026 | | 8.62009 | | 5.58528 | 3.470 up | | 8.195 up | | 2.361 up | lipase, extracellular |
| 79010 | 0.000114 | 12.33703 | | 13.27746 | | 10.20863 | 4.372 up | | 8.390 up | | 1.919 up | lipase, putative |
| 123204 | 0.000391 | 12.33311 | | 13.92556 | | 12.15618 | 1.130 up | | 3.409 up | | 3.015 up | lipase/esterase |
| 65782 | 0.000811 | 10.84264 | | 11.94614 | | 9.91374 | 1.903 up | | 4.090 up | | 2.148 up | lipase/esterase (α/ß fold) |
| 59887 | 0.00245 | 8.47965 | | 7.16257 | | 5.92128 | 5.890 up | | 2.364 up | | 2.491 down | L-lactate dehydrogenase, putative |
| 62651 | 0.000242 | 12.13307 | | 11.382 | | 8.63364 | 11.309 up | | 6.719 up | | 1.683 down | long-chain fatty acid transporter, |
| 49489 | 0.000696 | 12.88766 | | 13.96364 | | 12.15839 | 1.657 up | | 3.494 up | | 2.108 up | long-chain-fatty-acid-CoA ligase |
| 68097 | 0.0033 | 12.49311 | | 13.34175 | | 11.61868 | 1.833 up | | 3.301 up | | 1.800 up | MADS-box |
| 2076 | 0.000761 | 9.84139 | | 7.54474 | | 6.02485 | 14.089 up | | 2.867 up | | 4.913 down | malate permease |
| 79271 | 0.00055 | 13.80544 | | 14.04108 | | 11.36241 | 5.437 up | | 6.402 up | | 1.177 up | malate synthase |
| 67718 | 0.000298 | 8.13244 | | 9.91629 | | 7.89643 | 1.177 up | | 4.055 up | | 3.443 up | Malate/L-lactate dehydrogenase |
| 79737 | 0.000272 | 11.03131 | | 12.32159 | | 10.13984 | 1.855 up | | 4.537 up | | 2.445 up | Maleylacetoacetate isomerase, putative |
| 119896 | 0.000131 | 11.85475 | | 12.86515 | | 10.73796 | 2.168 up | | 4.368 up | | 2.014 up | malic enzyme |
| 76758 | 0.000946 | 8.59061 | | 9.50388 | | 6.80969 | 3.436 up | | 6.471 up | | 1.883 up | maltose permease |
| 65547 | 0.00147 | 8.17751 | | 9.62688 | | 6.43809 | 3.339 up | | 9.118 up | | 2.730 up | mandelate racemase/muconate lactonase-like protein |
| 109673 | 0.00181 | 7.56772 | | 7.35399 | | 4.78251 | 6.893 up | | 5.944 up | | 1.159 down | Mandelate racemase/muconate lactonizing enzyme |
| 104599 | 0.000809 | 10.44776 | | 9.42654 | | 8.02801 | 5.350 up | | 2.636 up | | 2.029 down | Mandelate racemase/muconate lactonizing enzyme |
| 56690 | 0.000806 | 11.68864 | | 12.53351 | | 10.92146 | 1.701 up | | 3.056 up | | 1.796 up | mannose-1-phosphate guanylyltransferase, putative |
| 58701 | 0.000417 | 8.58851 | | 8.33589 | | 6.15452 | 5.403 up | | 4.535 up | | 1.191 down | Mannose-6-phosphate isomerase |
| 21824 | 0.00749 | 11.11699 | | 12.55516 | | 10.48405 | 1.550 up | | 4.202 up | | 2.709 up | MaoC-like peroxisomal dehydratase |
| 3830 | 0.00144 | 10.26528 | | 12.28712 | | 10.79229 | 1.440 down | | 2.818 up | | 4.061 up | MAPKK Bck-1 like MAP kinase kinase |
| 124341 | 0.000175 | 11.80379 | | 11.58617 | | 7.47568 | 20.085 up | | 17.273 up | | 1.162 down | mating protein MAT1-2-1 |
| 71010 | 0.00185 | 7.97973 | | 8.26687 | | 6.40524 | 2.978 up | | 3.634 up | | 1.220 up | MDR-type ABC transporters |
| 73924 | 0.00108 | 11.1405 | | 12.87486 | | 10.01803 | 2.177 up | | 7.244 up | | 3.327 up | MDR-type ABC transporters |
| 110499 | 0.00055 | 8.72289 | | 11.56005 | | 8.46648 | 1.194 up | | 8.536 up | | 7.146 up | MDR-type ABC transporters |
| 66128 | 0.00955 | 9.73655 | | 12.1255 | | 9.91139 | 1.128 down | | 4.639 up | | 5.237 up | Mec1p/ATR family of phosphatidyl inositol-3-kinase-like proteins |
| 62026 | 0.00943 | 9.33744 | | 10.31655 | | 8.1548 | 2.269 up | | 4.474 up | | 1.971 up | mediator of RNA polymerase II transcription subunit 10, putative |
| 65895 | 0.00132 | 9.02877 | | 10.20728 | | 8.50091 | 1.441 up | | 3.263 up | | 2.263 up | mediator of RNA polymerase II transcription subunit 31 |
| 63815 | 0.00351 | 8.24031 | | 10.98605 | | 8.95773 | 1.644 down | | 4.079 up | | 6.707 up | Membrane coat complex Retromer, subunit Vps26 |
| 103119 | 0.00325 | 4.07778 | | 6.39728 | | 3.92251 | 1.113 up | | 5.558 up | | 4.991 up | membrane dipeptidase GliJ |
| 39590 | 0.00118 | 8.89114 | | 11.1626 | | 9.13852 | 1.187 down | | 4.067 up | | 4.828 up | Membrane protein Erj5 with DnaJ domain |
| 65324 | 0.000476 | 8.73042 | | 12.54147 | | 10.39463 | 3.169 down | | 4.428 up | | 14.035 up | metacaspase CasA |
| 1992 | 0.000288 | 11.97996 | | 10.99184 | | 9.54768 | 5.397 up | | 2.721 up | | 1.983 down | metal dependent phosphohydrolase |
| 68427 | 0.000106 | 11.15598 | | 9.54264 | | 8.13609 | 8.111 up | | 2.651 up | | 3.059 down | metal-dependent phosphohydrolase |
| 5369 | 0.000423 | 3.50992 | | 9.66382 | | 6.63174 | 8.704 down | | 8.179 up | | 71.204 up | Metallocarboxypeptidase, putative |
| 65494 | 0.000284 | 9.71102 | | 10.83885 | | 7.69099 | 4.055 up | | 8.863 up | | 2.185 up | metallopeptidase |
| 41208 | 0.000299 | 10.97032 | | 11.00607 | | 8.97372 | 3.990 up | | 4.090 up | | 1.025 up | metallophosphoesterase domain-containing protein, putative |
| 59244 | 0.00144 | 3.98231 | | 5.64738 | | 4.0229 | 1.028 down | | 3.083 up | | 3.171 up | metalloprotease, putative |
| 3049 | 0.000563 | 13.72196 | | 11.63251 | | 11.01552 | 6.527 up | | 1.533 up | | 4.255 down | methionine aminopeptidase |
| 3823 | 0.000311 | 9.32658 | | 12.25023 | | 10.00149 | 1.596 down | | 4.752 up | | 7.587 up | methionine synthase, vitamin-B12 independent |
| 112126 | 0.000255 | 12.75926 | | 13.1778 | | 8.06977 | 25.803 up | | 34.488 up | | 1.336 up | Methylmalonate semialdehyde dehydrogenase |
| 23237 | 0.00334 | 12.2083 | | 11.03295 | | 10.78171 | 2.688 up | | 1.190 up | | 2.258 down | Methylthioadenosine phosphorylase MTAP |
| 61284 | 0.00152 | 9.55261 | | 11.72833 | | 10.13222 | 1.494 down | | 3.023 up | | 4.518 up | methyltransferase |
| 108914 | 0.000568 | 11.09306 | | 13.78474 | | 11.60389 | 1.424 down | | 4.534 up | | 6.460 up | methyltransferase type 11 |
| 75165 | 0.000236 | 11.49149 | | 13.95963 | | 9.85051 | 3.118 up | | 17.257 up | | 5.533 up | MFS permease |
| 44175 | 0.000179 | 13.28382 | | 13.56692 | | 10.78481 | 5.652 up | | 6.878 up | | 1.216 up | MFS H+ sugar transporter |
| 46819 | 0.0000458 | 8.57032 | | 12.73257 | | 5.82665 | 6.697 up | | 119.919 up | | 17.904 up | MFS hexose transporter |
| 77517 | 0.000267 | 10.14944 | | 12.04087 | | 9.89099 | 1.196 up | | 4.437 up | | 3.710 up | MFS hexose transporter |
| 48444 | 0.000055 | 10.77831 | | 12.27429 | | 8.11916 | 6.316 up | | 17.816 up | | 2.820 up | MFS maltose permease |
| 60467 | 0.000769 | 4.93176 | | 6.52026 | | 4.2546 | 1.598 up | | 4.808 up | | 3.007 up | MFS monocarboxylate transporter, putative |
| 70323 | 0.000333 | 11.67374 | | 12.6042 | | 10.77512 | 1.864 up | | 3.553 up | | 1.905 up | MFS monocarboxylic acid transporter, putative |
| 119710 | 0.000369 | 12.91339 | | 13.49157 | | 11.99453 | 1.890 up | | 2.822 up | | 1.492 up | MFS multidrug transporter |
| 59796 | 0.000307 | 12.69859 | | 12.6894 | | 10.757 | 3.841 up | | 3.816 up | | 1.006 down | MFS permease |
| 5656 | 0.0004 | 10.61865 | | 10.65695 | | 6.59168 | 16.301 up | | 16.740 up | | 1.026 up | MFS permease |
| 110430 | 0.000194 | 11.59184 | | 11.6315 | | 9.86171 | 3.317 up | | 3.410 up | | 1.027 up | MFS permease |
| 27770 | 0.000196 | 10.59011 | | 10.54546 | | 5.7958 | 27.747 up | | 26.902 up | | 1.031 down | MFS permease |
| 3405 | 0.000132 | 15.03648 | | 15.11259 | | 8.10197 | 122.319 up | | 128.945 up | | 1.054 up | MFS permease |
| 119789 | 0.000205 | 13.08899 | | 13.23811 | | 10.3704 | 6.582 up | | 7.299 up | | 1.108 up | MFS permease |
| 74953 | 0.00111 | 10.91875 | | 10.74278 | | 8.77336 | 4.424 up | | 3.916 up | | 1.129 down | MFS permease |
| 55077 | 0.00381 | 10.22266 | | 10.42409 | | 7.47287 | 6.726 up | | 7.734 up | | 1.149 up | MFS permease |
| 104816 | 0.0264 | 6.97767 | | 6.77429 | | 5.21813 | 3.385 up | | 2.940 up | | 1.151 down | MFS permease |
| 53903 | 0.000326 | 9.91096 | | 10.20887 | | 4.27678 | 49.665 up | | 61.057 up | | 1.229 up | MFS permease |
| 123473 | 0.000167 | 11.88666 | | 12.19416 | | 9.08354 | 6.979 up | | 8.637 up | | 1.237 up | MFS permease |
| 70932 | 0.000145 | 10.2827 | | 10.60472 | | 7.37762 | 7.490 up | | 9.363 up | | 1.250 up | MFS permease |
| 81670 | 0.000131 | 12.67942 | | 13.04578 | | 10.73139 | 3.858 up | | 4.973 up | | 1.289 up | MFS permease |
| 75021 | 0.00229 | 12.26034 | | 12.6741 | | 11.00944 | 2.379 up | | 3.170 up | | 1.332 up | MFS permease |
| 60889 | 0.000576 | 7.56748 | | 8.00591 | | 5.8863 | 3.206 up | | 4.345 up | | 1.355 up | MFS permease |
| 109677 | 0.000235 | 10.45641 | | 10.95856 | | 7.14001 | 9.961 up | | 14.109 up | | 1.416 up | MFS permease |
| 76897 | 0.000125 | 14.55408 | | 14.00514 | | 6.94466 | 195.281 up | | 133.479 up | | 1.463 down | MFS permease |
| 71059 | 0.000483 | 9.70037 | | 9.13981 | | 3.8073 | 59.427 up | | 40.294 up | | 1.474 down | MFS permease |
| 54632 | 0.000113 | 13.08162 | | 13.67217 | | 9.02092 | 16.687 up | | 25.128 up | | 1.505 up | MFS permease |
| 60329 | 0.000265 | 11.16882 | | 11.7635 | | 8.48989 | 6.403 up | | 9.670 up | | 1.510 up | MFS permease |
| 80058 | 0.000143 | 11.32284 | | 11.91757 | | 5.76388 | 47.142 up | | 71.194 up | | 1.510 up | MFS permease |
| 59388 | 0.00106 | 11.67669 | | 11.04662 | | 7.72484 | 15.474 up | | 9.998 up | | 1.547 down | MFS permease |
| 55634 | 0.00629 | 7.01291 | | 6.32575 | | 4.51585 | 5.645 up | | 3.506 up | | 1.610 down | MFS permease |
| 50618 | 0.000263 | 13.09672 | | 13.8055 | | 9.34189 | 13.499 up | | 22.063 up | | 1.634 up | MFS permease |
| 121482 | 0.000539 | 12.97375 | | 13.69653 | | 8.96623 | 16.083 up | | 26.543 up | | 1.650 up | MFS permease |
| 121608 | 0.000596 | 11.3236 | | 12.04957 | | 9.11798 | 4.612 up | | 7.629 up | | 1.654 up | MFS permease |
| 122153 | 0.000379 | 13.1578 | | 12.34867 | | 10.02591 | 8.765 up | | 5.002 up | | 1.752 down | MFS permease |
| 54036 | 0.000298 | 11.60665 | | 12.45772 | | 10.39566 | 2.314 up | | 4.175 up | | 1.803 up | MFS permease |
| 65915 | 0.0000566 | 12.60146 | | 13.45494 | | 6.84238 | 54.157 up | | 97.853 up | | 1.806 up | MFS permease |
| 4774 | 0.000136 | 9.5441 | | 10.4255 | | 5.88562 | 12.627 up | | 23.261 up | | 1.842 up | MFS permease |
| 59190 | 0.00509 | 9.98641 | | 10.89503 | | 8.7846 | 2.300 up | | 4.318 up | | 1.877 up | MFS permease |
| 106330 | 0.000214 | 12.98063 | | 13.90498 | | 10.63062 | 5.098 up | | 9.675 up | | 1.897 up | MFS permease |
| 70108 | 0.000638 | 9.22949 | | 8.30249 | | 4.84924 | 20.825 up | | 10.952 up | | 1.901 down | MFS permease |
| 62502 | 0.000315 | 12.31239 | | 13.27106 | | 7.94581 | 20.628 up | | 40.092 up | | 1.943 up | MFS permease |
| 61374 | 0.0000999 | 9.65547 | | 10.61567 | | 6.52547 | 8.754 up | | 17.032 up | | 1.945 up | MFS permease |
| 67334 | 0.000524 | 12.07595 | | 13.10765 | | 10.60194 | 2.777 up | | 5.679 up | | 2.044 up | MFS permease |
| 67752 | 0.000319 | 11.71644 | | 12.75059 | | 6.88483 | 28.474 up | | 58.313 up | | 2.047 up | MFS permease |
| 2068 | 0.000473 | 6.25791 | | 5.20654 | | 3.79421 | 5.516 up | | 2.661 up | | 2.072 down | MFS permease |
| 105260 | 0.000103 | 11.56466 | | 10.51311 | | 3.81224 | 215.630 up | | 104.031 up | | 2.072 down | MFS permease |
| 68812 | 0.000342 | 13.72203 | | 14.78111 | | 11.92857 | 3.466 up | | 7.222 up | | 2.083 up | MFS permease |
| 103179 | 0.000181 | 8.42008 | | 9.5202 | | 4.91556 | 11.349 up | | 24.329 up | | 2.143 up | MFS permease |
| 82309 | 0.0000722 | 11.82562 | | 12.93055 | | 7.53156 | 19.617 up | | 42.194 up | | 2.150 up | MFS permease |
| 56095 | 0.000357 | 14.26173 | | 10.79876 | | 9.46992 | 27.699 up | | 2.512 up | | 11.026 down | MFS permease |
| 28036 | 0.000549 | 8.48686 | | 6.91548 | | 5.19562 | 9.789 up | | 3.294 up | | 2.971 down | MFS permease |
| 69026 | 0.000442 | 9.35644 | | 12.73833 | | 6.14832 | 9.241 up | | 96.336 up | | 10.424 up | MFS permease |
| 56289 | 0.000134 | 7.17429 | | 11.18884 | | 3.0183 | 17.826 up | | 288.122 up | | 16.162 up | MFS permease |
| 67541 | 0.000352 | 6.17568 | | 10.30893 | | 3.8886 | 4.880 up | | 85.646 up | | 17.548 up | MFS permease |
| 77785 | 0.0000873 | 9.56991 | | 10.94965 | | 6.83662 | 6.649 up | | 17.303 up | | 2.602 up | MFS permease |
| 111724 | 0.000954 | 7.95341 | | 9.4113 | | 6.8199 | 2.193 up | | 6.026 up | | 2.747 up | MFS permease |
| 60177 | 0.000368 | 8.55642 | | 10.09081 | | 5.81925 | 6.667 up | | 19.313 up | | 2.896 up | MFS permease |
| 69957 | 0.0000948 | 8.01666 | | 12.88984 | | 4.73698 | 9.711 up | | 284.614 up | | 29.307 up | MFS permease |
| 69611 | 0.000579 | 10.85742 | | 12.44463 | | 9.48101 | 2.596 up | | 7.800 up | | 3.004 up | MFS permease |
| 69651 | 0.000367 | 8.54158 | | 10.17952 | | 4.96743 | 11.910 up | | 37.067 up | | 3.112 up | MFS permease |
| 123241 | 0.0000695 | 11.65156 | | 13.35412 | | 6.68732 | 31.216 up | | 101.603 up | | 3.254 up | MFS permease |
| 70319 | 0.000279 | 6.07918 | | 7.81547 | | 4.49394 | 3.000 up | | 9.997 up | | 3.331 up | MFS permease |
| 38341 | 0.000115 | 9.803 | | 11.55037 | | 8.1902 | 3.058 up | | 10.268 up | | 3.357 up | MFS permease |
| 111888 | 0.000141 | 10.13179 | | 12.02978 | | 5.53622 | 24.177 up | | 90.106 up | | 3.726 up | MFS permease |
| 104549 | 0.000209 | 12.20473 | | 14.12978 | | 7.2466 | 31.084 up | | 118.043 up | | 3.797 up | MFS permease |
| 56684 | 0.000105 | 7.69371 | | 12.74245 | | 3.4029 | 19.573 up | | 647.866 up | | 33.099 up | MFS permease |
| 104320 | 0.000575 | 9.93577 | | 12.08604 | | 7.03219 | 7.482 up | | 33.216 up | | 4.439 up | MFS permease |
| 28409 | 0.000158 | 9.12903 | | 11.42177 | | 5.62021 | 11.383 up | | 55.775 up | | 4.899 up | MFS permease |
| 54005 | 0.000289 | 9.24518 | | 11.79234 | | 6.94425 | 4.927 up | | 28.801 up | | 5.844 up | MFS permease |
| 67692 | 0.019 | 7.05287 | | 10.12178 | | 4.22868 | 7.082 up | | 59.429 up | | 8.391 up | MFS permease |
| 5000 | 0.000448 | 5.36638 | | 8.53513 | | 4.34718 | 2.026 up | | 18.226 up | | 8.992 up | MFS permease |
| 62488 | 0.000205 | 10.02033 | | 13.30446 | | 8.80253 | 2.325 up | | 22.657 up | | 9.741 up | MFS permease |
| 68925 | 0.000662 | 8.23524 | | 5.84437 | | 4.77875 | 10.977 up | | 2.093 up | | 5.244 down | MFS permease |
| 53611 | 0.00026 | 10.79162 | | 8.53695 | | 7.4921 | 9.845 up | | 2.063 up | | 4.772 down | MFS permease |
| 27181 | 0.0000724 | 13.84376 | | 10.60333 | | 10.0652 | 13.723 up | | 1.452 up | | 9.450 down | MFS permease |
| 61278 | 0.00155 | 8.56231 | | 7.35384 | | 7.12167 | 2.714 up | | 1.174 up | | 2.310 down | MFS permease |
| 54972 | 0.00645 | 8.46523 | | 6.57467 | | 6.47158 | 3.982 up | | 1.074 up | | 3.707 down | MFS permease |
| 69563 | 0.000165 | 4.10168 | | 9.92406 | | 6.86059 | 6.768 down | | 8.359 up | | 56.586 up | MFS permease |
| 62171 | 0.000883 | 8.05229 | | 9.82463 | | 7.06562 | 1.981 up | | 6.769 up | | 3.416 up | MFS permease |
| 65153 | 0.000174 | 9.44898 | | 12.36198 | | 8.52232 | 1.900 up | | 14.316 up | | 7.531 up | MFS permease |
| 21422 | 0.00118 | 12.8018 | | 13.81085 | | 11.89814 | 1.870 up | | 3.765 up | | 2.012 up | MFS permease |
| 3765 | 0.0086 | 6.09535 | | 6.84294 | | 5.24989 | 1.796 up | | 3.016 up | | 1.678 up | MFS permease |
| 64314 | 0.0275 | 4.81949 | | 9.55082 | | 5.61711 | 1.738 down | | 15.281 up | | 26.562 up | MFS permease |
| 122013 | 0.00269 | 12.00854 | | 13.17591 | | 11.30221 | 1.631 up | | 3.664 up | | 2.246 up | MFS permease |
| 3330 | 0.000398 | 5.40141 | | 7.00569 | | 4.72493 | 1.598 up | | 4.859 up | | 3.040 up | MFS permease |
| 70830 | 0.00204 | 9.31088 | | 10.16543 | | 8.68356 | 1.544 up | | 2.793 up | | 1.808 up | MFS permease |
| 123809 | 0.00751 | 11.89071 | | 12.64364 | | 11.28519 | 1.521 up | | 2.564 up | | 1.685 up | MFS permease |
| 70172 | 0.00735 | 10.21648 | | 12.02489 | | 9.71932 | 1.411 up | | 4.943 up | | 3.502 up | MFS permease |
| 21595 | 0.00241 | 10.53043 | | 11.657 | | 10.05793 | 1.387 up | | 3.029 up | | 2.183 up | MFS permease |
| 109211 | 0.000486 | 10.90914 | | 12.49923 | | 10.48546 | 1.341 up | | 4.038 up | | 3.010 up | MFS permease |
| 56952 | 0.000723 | 11.16301 | | 12.84087 | | 10.74511 | 1.335 up | | 4.274 up | | 3.199 up | MFS permease |
| 80026 | 0.00188 | 12.5041 | | 13.59766 | | 12.13488 | 1.291 up | | 2.756 up | | 2.134 up | MFS permease |
| 80767 | 0.000653 | 11.22034 | | 12.9995 | | 11.00767 | 1.158 up | | 3.977 up | | 3.432 up | MFS permease |
| 2540 | 0.0027 | 6.39159 | | 7.86303 | | 6.54094 | 1.109 down | | 2.500 up | | 2.772 up | MFS permease |
| 55407 | 0.00169 | 10.8167 | | 12.40788 | | 10.96448 | 1.107 down | | 2.719 up | | 3.012 up | MFS permease |
| 5890 | 0.00563 | 4.06845 | | 7.34125 | | 3.98301 | 1.061 up | | 10.254 up | | 9.665 up | MFS permease |
| 121986 | 0.000133 | 12.04453 | | 10.97026 | | 7.93107 | 17.309 up | | 8.220 up | | 2.105 down | MFS permease |
| 106029 | 0.000253 | 11.57535 | | 9.67775 | | 8.78839 | 6.901 up | | 1.852 up | | 3.725 down | MFS permease |
| 68990 | 0.00157 | 13.1945 | | 11.37038 | | 11.81294 | 2.605 up | | 1.359 down | | 3.540 down | MFS permease |
| 105565 | 0.000309 | 9.23922 | | 10.17502 | | 8.59822 | 1.559 up | | 2.983 up | | 1.912 up | MFS permease |
| 109837 | 0.00483 | 4.07005 | | 5.65028 | | 3.57425 | 1.410 up | | 4.216 up | | 2.990 up | MFS permease |
| 50894 | 0.0000713 | 11.11453 | | 14.68807 | | 6.5857 | 23.084 up | | 274.825 up | | 11.905 up | MFS permease |
| 61496 | 0.000921 | 10.6344 | | 13.0848 | | 11.44013 | 1.748 down | | 3.126 up | | 5.465 up | MFS permease |
| 79116 | 0.00776 | 12.40188 | | 13.5521 | | 11.81635 | 1.500 up | | 3.330 up | | 2.219 up | MFS permease |
| 62971 | 0.000881 | 8.49115 | | 12.27298 | | 8.14751 | 1.268 up | | 17.453 up | | 13.754 up | MFS permease |
| 63145 | 0.00279 | 9.7536 | | 12.0156 | | 9.72644 | 1.018 up | | 4.887 up | | 4.796 up | MFS permease |
| 78833 | 0.0000247 | 12.27268 | | 13.25637 | | 6.74469 | 46.141 up | | 91.245 up | | 1.977 up | MFS permease (fucose permease) |
| 22912 | 0.000096 | 12.76055 | | 13.69479 | | 7.33048 | 43.113 up | | 82.384 up | | 1.910 up | MFS permease (glucose permease HXT1) |
| 65191 | 0.000172 | 8.07484 | | 12.98612 | | 5.05701 | 8.099 up | | 243.724 up | | 30.091 up | MFS permease (maltose permease) |
| 70349 | 0.000169 | 12.45468 | | 10.8255 | | 8.57634 | 14.706 up | | 4.754 up | | 3.093 down | MFS permease LIZ1 |
| 69834 | 0.000101 | 11.63728 | | 13.29601 | | 7.36429 | 19.332 up | | 61.041 up | | 3.157 up | MFS permease, |
| 79202 | 0.0000247 | 11.83411 | | 13.71833 | | 6.87263 | 31.156 up | | 115.016 up | | 3.691 up | MFS permease, associated with cellulose signalling |
| 3532 | 0.00171 | 5.23957 | | 6.78641 | | 4.44943 | 1.729 up | | 5.052 up | | 2.921 up | MFS sugar permease |
| 65926 | 0.00387 | 8.33315 | | 9.80604 | | 8.17763 | 1.113 up | | 3.091 up | | 2.775 up | Mgs1, a DNA-dependent ATPase possibly involved in Okazaki fragment processing |
| 58183 | 0.000837 | 9.83141 | | 11.4752 | | 10.01144 | 1.132 down | | 2.758 up | | 3.124 up | MIF4G domain-containing protein (Initiation factor eIF-4 gamma, middle) |
| 45971 | 0.00168 | 10.14859 | | 10.58991 | | 8.85869 | 2.445 up | | 3.320 up | | 1.357 up | mismatched base pair and cruciform DNA recognition protein |
| 103853 | 0.000115 | 9.76374 | | 12.52624 | | 9.88646 | 1.088 down | | 6.232 up | | 6.785 up | Mitochondrial carnitine/acyl carnitine carrier |
| 110133 | 0.00533 | 11.52346 | | 10.97833 | | 9.7153 | 3.501 up | | 2.399 up | | 1.459 down | Mitochondrial carrier protein |
| 69081 | 0.000329 | 10.50134 | | 12.47003 | | 9.03144 | 2.770 up | | 10.842 up | | 3.914 up | mitochondrial cytochrome b2, putative |
| 47930 | 0.000226 | 9.0986 | | 10.31888 | | 4.05951 | 32.878 up | | 76.604 up | | 2.329 up | Mitochondrial oxoglutarate/malate carrier proteins |
| 121743 | 0.000247 | 12.97893 | | 13.45073 | | 8.33109 | 25.069 up | | 34.766 up | | 1.386 up | Mitochondrial succinate/fumarate antiporter |
| 63047 | 0.000631 | 10.11361 | | 11.90602 | | 10.25434 | 1.102 down | | 3.141 up | | 3.463 up | MMR; HR regulation |
| 82606 | 0.00285 | 11.07683 | | 12.50511 | | 11.07579 | 1.000 up | | 2.693 up | | 2.691 up | Mn2+ homeostasis protein Per1 |
| 22625 | 0.00105 | 9.71681 | | 11.74739 | | 10.09127 | 1.296 down | | 3.151 up | | 4.085 up | Molecular chaperone (DnaJ superfamily) |
| 65819 | 0.000845 | 7.73405 | | 7.90037 | | 6.16866 | 2.959 up | | 3.321 up | | 1.122 up | Molecular chaperone Hsp70 family |
| 78561 | 0.00142 | 11.58319 | | 12.31162 | | 10.8534 | 1.658 up | | 2.747 up | | 1.656 up | Molybdenum cofactor biosynthesis protein |
| 63703 | 0.00135 | 9.81795 | | 10.57607 | | 9.14307 | 1.596 up | | 2.700 up | | 1.691 up | molybdopterin synthase small subunit CnxG, putative |
| 55630 | 0.000582 | 10.45347 | | 7.90762 | | 9.12834 | 2.505 up | | 2.330 down | | 5.839 down | monocarboxylate transporter |
| 69282 | 0.0000992 | 10.89343 | | 8.48185 | | 8.18102 | 6.554 up | | 1.231 up | | 5.320 down | monocarboxylate transporter |
| 66854 | 0.00205 | 9.56856 | | 6.64251 | | 6.44997 | 8.685 up | | 1.142 up | | 7.600 down | Monocarboxylate transporter |
| 64920 | 0.000372 | 9.63892 | | 13.86764 | | 11.68088 | 4.118 down | | 4.552 up | | 18.748 up | Monocarboxylate transporter |
| 69771 | 0.00106 | 9.71578 | | 10.49902 | | 8.86076 | 1.808 up | | 3.112 up | | 1.720 up | Monocarboxylate transporter |
| 106248 | 0.000114 | 11.08785 | | 14.25052 | | 11.67145 | 1.498 down | | 5.975 up | | 8.954 up | monosaccharide transporter (galactose permease ?) |
| 108533 | 0.000755 | 10.20848 | | 12.13777 | | 10.56624 | 1.281 down | | 2.972 up | | 3.808 up | Mrc1p |
| 61532 | 0.00955 | 11.64729 | | 12.74466 | | 11.39291 | 1.192 up | | 2.552 up | | 2.139 up | mRNA binding protein Pumilio 2, putative |
| 38767 | 0.0353 | 9.68644 | | 10.3628 | | 8.93558 | 1.682 up | | 2.689 up | | 1.598 up | mRNA capping enzyme |
| 55636 | 0.000638 | 10.40346 | | 10.1682 | | 8.2389 | 4.483 up | | 3.808 up | | 1.177 down | MRP-type ABC transporter |
| 80028 | 0.000552 | 10.22213 | | 10.75753 | | 8.75614 | 2.762 up | | 4.003 up | | 1.449 up | MRP-type ABC transporter |
| 60116 | 0.000291 | 13.65339 | | 11.35221 | | 11.51143 | 4.413 up | | 1.116 down | | 4.928 down | MRP-type ABC transporter |
| 54954 | 0.0121 | 6.52691 | | 8.14844 | | 6.19378 | 1.259 up | | 3.876 up | | 3.077 up | MRP-type ABC transporter |
| 122374 | 0.000104 | 11.11892 | | 13.9815 | | 5.17481 | 61.567 up | | 447.794 up | | 7.273 up | MRSP1/expansin-like |
| 112325 | 0.00171 | 11.17898 | | 9.9775 | | 9.52884 | 3.138 up | | 1.364 up | | 2.299 down | MRSP1/expansin-like |
| 104423 | 0.00016 | 5.10062 | | 12.48355 | | 4.56812 | 1.446 up | | 241.425 up | | 166.910 up | MRSP1/expansin-like |
| 122375 | 0.000497 | 3.39014 | | 7.68717 | | 3.09336 | 1.228 up | | 24.147 up | | 19.657 up | MRSP1/expansin-like |
| 55566 | 0.000181 | 7.7711 | | 11.03428 | | 7.04868 | 1.649 up | | 15.841 up | | 9.600 up | multicopper oidase |
| 107848 | 0.000358 | 9.8498 | | 12.60106 | | 8.82865 | 2.029 up | | 13.664 up | | 6.733 up | multicopper oxidase type 1, secreted |
| 121098 | 0.00157 | 12.54871 | | 13.57931 | | 11.78045 | 1.703 up | | 3.479 up | | 2.042 up | Multicopper oxidases |
| 124079 | 0.00145 | 3.148 | | 8.07338 | | 3.32746 | 1.132 down | | 26.832 up | | 30.386 up | Multicopper oxidases |
| 104159 | 0.0000623 | 10.13066 | | 11.69311 | | 10.32098 | 1.141 down | | 2.588 up | | 2.953 up | MUS26 of Neurospora crassa |
| 67527 | 0.00413 | 10.42236 | | 11.56536 | | 10.10261 | 1.248 up | | 2.756 up | | 2.208 up | Mus81p, a subunit of the Mus81-Mms4 structure-specific endonuclease |
| 4124 | 0.000215 | 6.34025 | | 11.5845 | | 5.68043 | 1.579 up | | 59.882 up | | 37.903 up | myb transcriptional regulator |
| 1941 | 0.000275 | 7.19253 | | 9.07596 | | 6.74176 | 1.366 up | | 5.042 up | | 3.689 up | myb transcriptional regulator |
| 105643 | 0.00175 | 12.01304 | | 13.25447 | | 11.65301 | 1.283 up | | 3.034 up | | 2.364 up | myb transcriptional regulator |
| 119999 | 0.000781 | 12.5209 | | 14.03134 | | 11.29415 | 2.340 up | | 6.667 up | | 2.848 up | Myb-DNA binding domain protein |
| 22950 | 0.000999 | 10.99473 | | 12.14187 | | 10.72563 | 1.205 up | | 2.668 up | | 2.214 up | MYND domain protein (SamB) |
| 67971 | 0.000196 | 10.1637 | | 11.7273 | | 8.48332 | 3.205 up | | 9.473 up | | 2.955 up | MYND-type Zn-finger protein |
| 119790 | 0.000111 | 10.06893 | | 11.43697 | | 6.62801 | 10.859 up | | 28.031 up | | 2.581 up | myo-inositol oxygenase , |
| 79671 | 0.00066 | 10.94188 | | 12.08618 | | 8.98868 | 3.872 up | | 8.559 up | | 2.210 up | N-acetyl-glucosamine-6-phosphate deacetylase |
| 3101 | 0.00285 | 10.28625 | | 12.54524 | | 10.71855 | 1.349 down | | 3.547 up | | 4.786 up | N-acetylglucosaminyl-phosphatidylinositol deacetylase, putative |
| 30250 | 0.000386 | 9.29899 | | 10.21622 | | 7.69972 | 3.029 up | | 5.721 up | | 1.888 up | N-acetylglucosaminyltransferase |
| 123668 | 0.0019 | 10.53112 | | 10.67273 | | 8.65 | 3.683 up | | 4.063 up | | 1.103 up | N-acetyltransferase activity |
| 111236 | 0.000537 | 11.53581 | | 10.87953 | | 9.79196 | 3.349 up | | 2.125 up | | 1.576 down | N-acetyltransferase of bacterial origin |
| 54230 | 0.000247 | 9.27075 | | 7.74453 | | 5.88075 | 10.483 up | | 3.639 up | | 2.880 down | NACHT domain WD40 repeat-containing protein, related to HET |
| 65771 | 0.00893 | 3.31508 | | 5.70065 | | 4.01573 | 1.625 down | | 3.215 up | | 5.225 up | NACHT domain WD40 repeat-containing protein, related to HET |
| 56499 | 0.00139 | 8.67153 | | 11.99028 | | 9.28495 | 1.529 down | | 6.522 up | | 9.978 up | N-acyl-phosphatidylethanolamine-hydrolyzing phospholipase D, putative |
| 47432 | 0.000128 | 13.90121 | | 13.74477 | | 9.53585 | 20.611 up | | 18.493 up | | 1.114 down | NAD(P) transhydrogenase beta subunit |
| 110271 | 0.00775 | 5.01323 | | 5.46125 | | 4.09704 | 1.887 up | | 2.574 up | | 1.364 up | NAD-dependent epimerase/dehydratase |
| 70316 | 0.000755 | 10.71604 | | 10.60888 | | 8.98751 | 3.313 up | | 3.076 up | | 1.077 down | NAD-dependent epimerase/dehydratase family |
| 120943 | 0.000838 | 13.75983 | | 13.88839 | | 11.85179 | 3.753 up | | 4.102 up | | 1.093 up | NAD-dependent glutamate dehydrogenase |
| 76204 | 0.00261 | 7.17674 | | 7.33882 | | 5.17697 | 3.999 up | | 4.474 up | | 1.118 up | NADH:flavin oxidoreductase/12-oxophytodienoate reductase |
| 76366 | 0.000137 | 12.29476 | | 11.41085 | | 9.4987 | 6.945 up | | 3.763 up | | 1.845 down | NADH:flavin oxidoreductase/12-oxophytodienoate reductase |
| 78357 | 0.00967 | 13.87737 | | 13.1404 | | 12.14538 | 3.321 up | | 1.993 up | | 1.666 down | NADH-quinone oxidoreductase, putative |
| 53079 | 0.000901 | 10.68364 | | 8.58795 | | 9.05205 | 3.098 up | | 1.379 down | | 4.274 down | NADP/FAD dependent oxidoreductase |
| 3447 | 0.00022 | 8.79125 | | 8.7803 | | 4.4831 | 19.809 up | | 19.660 up | | 1.007 down | NADP-dependent alcohol dehydrogenase (class V) |
| 120969 | 0.00324 | 10.53232 | | 11.94872 | | 10.50517 | 1.018 up | | 2.719 up | | 2.669 up | NADPH oxidase regulator NoxR |
| 28781 | 0.00177 | 8.76421 | | 10.19844 | | 8.39217 | 1.294 up | | 3.497 up | | 2.702 up | NDT80/PhoG like DNA-binding domain-containing protein |
| 65832 | 0.000354 | 11.10463 | | 12.47393 | | 11.14934 | 1.031 down | | 2.504 up | | 2.583 up | Neurospora ro-7 |
| 107142 | 0.0033 | 4.26104 | | 6.45733 | | 4.37177 | 1.079 down | | 4.244 up | | 4.582 up | neutral protease 2 |
| 57728 | 0.00343 | 6.87073 | | 8.80396 | | 6.82079 | 1.035 up | | 3.953 up | | 3.819 up | NimA interactive protein, putative |
| 74366 | 0.00173 | 10.51765 | | 12.28739 | | 9.73099 | 1.725 up | | 5.882 up | | 3.409 up | nitrilase |
| 64996 | 0.00104 | 3.94586 | | 8.39732 | | 4.42752 | 1.396 down | | 15.668 up | | 21.878 up | nitrilase |
| 64295 | 0.00055 | 13.48613 | | 12.7704 | | 12.16224 | 2.503 up | | 1.524 up | | 1.642 down | nitrogen permease regulator Npr2, putative |
| 5855 | 0.000276 | 5.74036 | | 7.40359 | | 5.7457 | 1.003 down | | 3.155 up | | 3.167 up | NmrA family protein |
| 109313 | 0.00222 | 5.85975 | | 6.8239 | | 5.42164 | 1.354 up | | 2.643 up | | 1.950 up | NmrA-like family protein |
| 79498 | 0.000794 | 11.98687 | | 13.43194 | | 12.05122 | 1.045 down | | 2.603 up | | 2.722 up | NoxA |
| 68204 | 0.000405 | 8.67123 | | 12.02577 | | 6.75694 | 3.769 up | | 38.554 up | | 10.228 up | NRPS |
| 123786 | 0.00021 | 12.64858 | | 14.28445 | | 9.02264 | 12.345 up | | 38.367 up | | 3.107 up | NRPS |
| 67189 | 0.000379 | 8.1292 | | 9.79034 | | 6.63729 | 2.812 up | | 8.895 up | | 3.162 up | NRPS |
| 23171 | 0.000323 | 10.52038 | | 13.78723 | | 8.94237 | 2.985 up | | 28.737 up | | 9.625 up | NRPS |
| 71005 | 0.000307 | 4.36693 | | 8.46827 | | 5.14813 | 1.718 down | | 9.987 up | | 17.164 up | NRPS |
| 81014 | 0.00289 | 10.57067 | | 12.06727 | | 10.17697 | 1.313 up | | 3.707 up | | 2.821 up | NRPS |
| 70084 | 0.000587 | 10.7259 | | 12.65013 | | 10.80614 | 1.057 down | | 3.590 up | | 3.795 up | Nuclease (Borde 2007) |
| 61794 | 0.000159 | 9.21491 | | 12.56828 | | 10.89187 | 3.197 down | | 3.196 up | | 10.220 up | nuclease, mitochondrial |
| 62977 | 0.000391 | 8.79947 | | 10.13459 | | 7.74373 | 2.078 up | | 5.244 up | | 2.522 up | nucleoside diphosphate sugar epimerase, secreted |
| 73764 | 0.00283 | 11.62076 | | 13.15251 | | 10.84175 | 1.715 up | | 4.961 up | | 2.891 up | nucleoside transporter |
| 120017 | 0.000357 | 14.95707 | | 14.4883 | | 13.31721 | 3.116 up | | 2.251 up | | 1.383 down | oligopeptide transporter |
| 46794 | 0.00136 | 13.13893 | | 12.13794 | | 11.14518 | 3.982 up | | 1.989 up | | 2.001 down | oligopeptide transporter |
| 109487 | 0.000558 | 9.38852 | | 10.62242 | | 7.19085 | 4.587 up | | 10.789 up | | 2.352 up | oligopeptide transporter superfamily domain |
| 76247 | 0.00161 | 9.03986 | | 10.46011 | | 8.4914 | 1.462 up | | 3.914 up | | 2.676 up | O-methyltransferase family protein |
| 5366 | 0.00022 | 11.27648 | | 12.20361 | | 9.56568 | 3.273 up | | 6.224 up | | 1.901 up | O-methyltransferase, putative |
| 50323 | 0.00199 | 7.58615 | | 11.51967 | | 4.13962 | 10.902 up | | 166.577 up | | 15.279 up | OOC1 |
| 66077 | 0.000145 | 3.39357 | | 6.38986 | | 3.1028 | 1.223 up | | 9.761 up | | 7.979 up | ooc1-related protein |
| 58158 | 0.0178 | 12.05655 | | 12.06694 | | 10.66749 | 2.619 up | | 2.638 up | | 1.007 up | OPT family small oligopeptide transporter |
| 27398 | 0.00172 | 10.20121 | | 12.1144 | | 10.24893 | 1.033 down | | 3.643 up | | 3.766 up | origin recognition complex, subunit 2 |
| 123554 | 0.00373 | 10.83278 | | 10.96592 | | 9.4136 | 2.674 up | | 2.932 up | | 1.096 up | OTU-like cysteine protease, putative |
| 68492 | 0.00175 | 12.03925 | | 12.72733 | | 11.1108 | 1.903 up | | 3.066 up | | 1.611 up | ounknown proteinsterol binding protein |
| 53964 | 0.000444 | 3.6861 | | 8.85368 | | 5.0344 | 2.546 down | | 14.116 up | | 35.941 up | oxalate decarboxylase |
| 59746 | 0.000776 | 9.24125 | | 10.37419 | | 8.05956 | 2.268 up | | 4.974 up | | 2.193 up | oxaloacetase-like protein |
| 30476 | 0.000605 | 8.89317 | | 11.35402 | | 8.82454 | 1.048 up | | 5.773 up | | 5.505 up | oxidation resistance protein 1, putative |
| 58264 | 0.000134 | 9.46581 | | 11.51238 | | 9.83631 | 1.292 down | | 3.195 up | | 4.131 up | paxU orthologue ? (indole-terpene biosynthesis?) |
| 122131 | 0.00426 | 12.36141 | | 12.26767 | | 10.04114 | 4.994 up | | 4.680 up | | 1.067 down | PCMTProtein-L-isoaspartate(D-aspartate) O-methyltransferase |
| 68889 | 0.00299 | 11.27917 | | 11.50554 | | 10.12697 | 2.222 up | | 2.600 up | | 1.169 up | PDR-type ABC transporters |
| 82327 | 0.00218 | 12.54943 | | 11.56775 | | 10.53269 | 4.046 up | | 2.049 up | | 1.974 down | PDR-type ABC transporters |
| 59014 | 0.000743 | 9.97282 | | 14.26571 | | 11.7486 | 3.424 down | | 5.724 up | | 19.601 up | PDR-type ABC transporters |
| 76682 | 0.0000506 | 3.70093 | | 13.31481 | | 5.0233 | 2.500 down | | 313.323 up | | 783.543 up | PDR-type ABC transporters |
| 105081 | 0.00381 | 12.42843 | | 13.14425 | | 11.74964 | 1.600 up | | 2.629 up | | 1.642 up | peflin |
| 61258 | 0.00149 | 11.40462 | | 11.14039 | | 9.88012 | 2.876 up | | 2.395 up | | 1.200 down | Peptidase C12, ubiquitin carboxyl-terminal hydrolase 1 |
| 61765 | 0.000571 | 10.45133 | | 12.15813 | | 9.95863 | 1.407 up | | 4.593 up | | 3.264 up | Peptidase D |
| 122083 | 0.000335 | 9.87975 | | 12.74831 | | 10.41515 | 1.449 down | | 5.039 up | | 7.303 up | peptidase family M28 |
| 22402 | 0.00073 | 10.53319 | | 12.50345 | | 10.62323 | 1.064 down | | 3.681 up | | 3.918 up | Peptidase M, neutral zinc metallopeptidases, zinc-binding site |
| 47127 | 0.000113 | 9.97811 | | 13.68459 | | 11.46895 | 2.810 down | | 4.644 up | | 13.054 up | peptidase M18 |
| 69257 | 0.00251 | 8.19091 | | 9.95563 | | 8.40883 | 1.163 down | | 2.921 up | | 3.398 up | Peptidase M22, glycoprotease |
| 105810 | 0.00371 | 9.04128 | | 10.26249 | | 8.78156 | 1.197 up | | 2.791 up | | 2.331 up | Peptidase M4, thermolysin, putative |
| 103039 | 0.000161 | 5.71815 | | 11.7611 | | 9.74962 | 16.352 down | | 4.031 up | | 65.933 up | peptidase S41 |
| 123865 | 0.000196 | 11.32674 | | 13.06287 | | 8.78779 | 5.811 up | | 19.360 up | | 3.331 up | Peptidase S8 and S53, subtilisin, kexin, sedolisin |
| 80980 | 0.0000225 | 11.74108 | | 14.50885 | | 5.80657 | 61.159 up | | 416.530 up | | 6.810 up | peptidyl arginine deiminase |
| 51893 | 0.00054 | 13.3807 | | 14.64847 | | 12.82781 | 1.467 up | | 3.532 up | | 2.407 up | peroxidase/hem containing |
| 77870 | 0.0024 | 11.58144 | | 12.18028 | | 10.60584 | 1.966 up | | 2.978 up | | 1.514 up | peroxin 8, putative |
| 53342 | 0.00149 | 7.10099 | | 10.5026 | | 8.75576 | 3.148 down | | 3.356 up | | 10.567 up | Peroxin-3 |
| 50131 | 0.000531 | 13.39364 | | 13.68335 | | 12.22296 | 2.251 up | | 2.751 up | | 1.222 up | peroxisomal biogenesis factor 11 |
| 80788 | 0.00214 | 11.02674 | | 11.68382 | | 10.18694 | 1.789 up | | 2.822 up | | 1.576 up | Peroxisomal membrane anchor protein (Pex14p), putative |
| 75440 | 0.00261 | 12.10815 | | 12.50378 | | 11.00507 | 2.148 up | | 2.825 up | | 1.315 up | peroxisomal membrane protein (PmpP24) |
| 76701 | 0.0025 | 8.06826 | | 10.47151 | | 8.50912 | 1.357 down | | 3.897 up | | 5.289 up | peroxisomal membrane protein pex16 |
| 39387 | 0.000935 | 10.49463 | | 12.9271 | | 11.0582 | 1.477 down | | 3.652 up | | 5.398 up | Pex2/Pex12 |
| 103703 | 0.00061 | 9.61679 | | 11.74973 | | 10.16293 | 1.460 down | | 3.003 up | | 4.386 up | Pex2/Pex12 amino terminal region |
| 53989 | 0.000356 | 11.08701 | | 11.20798 | | 9.08113 | 4.016 up | | 4.367 up | | 1.087 up | pfkB family kinase, putative |
| 4941 | 0.000564 | 12.33717 | | 10.61611 | | 9.51755 | 7.059 up | | 2.141 up | | 3.296 down | phenylacetyl-CoA ligase, |
| 72549 | 0.000686 | 9.0514 | | 9.91596 | | 8.47751 | 1.488 up | | 2.710 up | | 1.820 up | phenylacrylic acid decarbounknown proteinlase |
| 79673 | 0.000394 | 12.86343 | | 13.55234 | | 12.10524 | 1.691 up | | 2.726 up | | 1.612 up | PhoG |
| 111890 | 0.00322 | 6.62901 | | 6.81562 | | 4.85832 | 3.412 up | | 3.883 up | | 1.138 up | Phosphate transporter |
| 60988 | 0.000113 | 9.73726 | | 11.74038 | | 6.9042 | 7.125 up | | 28.565 up | | 4.008 up | phosphate transporter |
| 124056 | 0.000286 | 12.98625 | | 12.87121 | | 11.42473 | 2.951 up | | 2.725 up | | 1.083 down | phosphatidic acid phosphatase |
| 50104 | 0.000923 | 9.37692 | | 11.48148 | | 9.07422 | 1.233 up | | 5.304 up | | 4.300 up | phosphatidyl synthase (A. fumigatus) |
| 104288 | 0.000978 | 9.67394 | | 9.6034 | | 8.27787 | 2.631 up | | 2.506 up | | 1.050 down | phosphatidylethanolamine-binding protein |
| 3362 | 0.000424 | 12.66472 | | 13.60759 | | 12.13368 | 1.444 up | | 2.777 up | | 1.922 up | Phosphatidylinositol 3- and 4-kinase |
| 80273 | 0.00256 | 11.902 | | 13.5553 | | 11.95184 | 1.035 down | | 3.038 up | | 3.145 up | phosphatidylinositol polyphosphate phosphatase |
| 78274 | 0.00227 | 8.07377 | | 9.64627 | | 7.33898 | 1.664 up | | 4.949 up | | 2.974 up | phosphatidylinositol-3-phosphate 5-kinase; fab1 |
| 2492 | 0.00289 | 10.30999 | | 10.08847 | | 8.33535 | 3.930 up | | 3.370 up | | 1.165 down | Phosphatidylinositol-4-phosphate 5-kinase |
| 82100 | 0.000379 | 9.71865 | | 11.76094 | | 10.07854 | 1.283 down | | 3.209 up | | 4.118 up | PhosphatidylinositolPolyphosphatePhosphatase |
| 66935 | 0.00029 | 7.344 | | 8.20476 | | 5.37157 | 3.924 up | | 7.126 up | | 1.815 up | Phosphatidylserine decarboxylase |
| 29642 | 0.000148 | 3.90252 | | 9.57975 | | 3.90751 | 1.003 down | | 50.993 up | | 51.170 up | Phosphatidylserine decarboxylase |
| 53685 | 0.00128 | 11.33228 | | 12.83759 | | 11.11808 | 1.160 up | | 3.293 up | | 2.838 up | phosphodiesterase/nucleotide pyrophosphatasetype 1 |
| 124115 | 0.000179 | 13.53599 | | 13.26325 | | 9.72003 | 14.083 up | | 11.657 up | | 1.208 down | phosphoenolpyruvate carboxykinase AcuF |
| 80003 | 0.00077 | 11.85565 | | 11.61306 | | 8.18014 | 12.777 up | | 10.799 up | | 1.183 down | Phosphoglycerate dehydrogenase and related dehydrogenases |
| 69061 | 0.00607 | 10.85914 | | 11.81503 | | 10.37602 | 1.397 up | | 2.711 up | | 1.939 up | phosphoglycerate mutase |
| 33371 | 0.00226 | 4.61673 | | 6.48994 | | 5.156 | 1.453 down | | 2.520 up | | 3.663 up | phosphoglycerate mutase family protein |
| 67579 | 0.000335 | 9.78115 | | 12.71786 | | 8.46961 | 2.482 up | | 19.004 up | | 7.656 up | phospholipase A2 |
| 21960 | 0.00027 | 11.53938 | | 13.98927 | | 9.95467 | 2.999 up | | 16.388 up | | 5.463 up | phospholipase C |
| 57975 | 0.00221 | 7.04754 | | 9.91539 | | 8.16185 | 2.164 down | | 3.371 up | | 7.299 up | phospholipase C, related to Aspergillus fumigatus phosphatidylinositol phospholipase C |
| 111102 | 0.00136 | 8.59048 | | 10.34874 | | 8.19699 | 1.313 up | | 4.443 up | | 3.382 up | Phospholipase C, related to Listeria monocytogenesPlcA (Evalue 1.62e-21) |
| 75311 | 0.0101 | 9.18236 | | 10.67289 | | 9.00039 | 1.134 up | | 3.187 up | | 2.809 up | phospholipase C, related to N. crassa phospholipase C |
| 78611 | 0.0000889 | 13.06207 | | 12.24519 | | 10.40701 | 6.298 up | | 3.575 up | | 1.761 down | phospholipase D |
| 79019 | 0.0032 | 10.26415 | | 12.08893 | | 10.16676 | 1.069 up | | 3.789 up | | 3.542 up | Phospholipase D |
| 22331 | 0.00037 | 8.79914 | | 10.65407 | | 8.81985 | 1.014 down | | 3.565 up | | 3.617 up | phospholipase D Active site motif protein / IQ calmodulin-binding motif protein |
| 122091 | 0.000163 | 12.41754 | | 13.73309 | | 10.12516 | 4.898 up | | 12.192 up | | 2.488 up | phospholipase of papatin-family |
| 107263 | 0.000464 | 10.56644 | | 12.81571 | | 10.91205 | 1.270 down | | 3.741 up | | 4.754 up | phospholipase of papatin-family |
| 124256 | 0.00354 | 10.28941 | | 12.38192 | | 10.70988 | 1.338 down | | 3.186 up | | 4.264 up | phospholipase-like protein |
| 75409 | 0.0119 | 11.4895 | | 12.54828 | | 10.64348 | 1.797 up | | 3.744 up | | 2.083 up | Phospholipid-translocating P-type ATPase, flippase; putative |
| 41504 | 0.00258 | 9.95407 | | 12.27793 | | 10.55236 | 1.513 down | | 3.307 up | | 5.006 up | phosphopantetheinyl transferase, putative |
| 3350 | 0.0013 | 11.76583 | | 12.1712 | | 10.64198 | 2.179 up | | 2.886 up | | 1.324 up | phosphopantothenate-cysteine ligase |
| 124132 | 0.000151 | 10.1262 | | 12.08333 | | 10.32129 | 1.144 down | | 3.391 up | | 3.882 up | pH-response regulator protein palA/RIM20 |
| 80523 | 0.000245 | 10.04922 | | 11.20914 | | 8.68138 | 2.580 up | | 5.766 up | | 2.234 up | pH-response regulator protein palC |
| 103108 | 0.000388 | 12.30622 | | 12.53121 | | 9.22223 | 8.479 up | | 9.910 up | | 1.168 up | phytanoyl-CoA dioxygenase family protein |
| 81275 | 0.00034 | 12.45627 | | 11.29581 | | 9.75718 | 6.493 up | | 2.905 up | | 2.235 down | phytanoyl-CoA dioxygenase family protein |
| 71092 | 0.000183 | 10.33715 | | 13.8289 | | 8.5357 | 3.485 up | | 39.211 up | | 11.249 up | phytase |
| 71566 | 0.0032 | 11.53007 | | 12.66082 | | 11.03965 | 1.404 up | | 3.076 up | | 2.189 up | phytase |
| 59482 | 0.000185 | 9.71621 | | 13.59985 | | 7.08804 | 6.182 up | | 91.253 up | | 14.760 up | PKS |
| 82208 | 0.0000857 | 7.50759 | | 12.60968 | | 5.10759 | 5.278 up | | 181.281 up | | 34.346 up | PKS |
| 106272 | 0.00064 | 8.36591 | | 9.66477 | | 7.39192 | 1.964 up | | 4.832 up | | 2.460 up | PKS |
| 60118 | 0.000335 | 8.04303 | | 10.62461 | | 7.21696 | 1.772 up | | 10.612 up | | 5.985 up | PKS |
| 65172 | 0.000507 | 5.02339 | | 6.76016 | | 5.28169 | 1.196 down | | 2.786 up | | 3.332 up | PKS |
| 65116 | 0.00438 | 4.04283 | | 6.65635 | | 3.78865 | 1.192 up | | 7.299 up | | 6.119 up | PKS |
| 111245 | 0.000315 | 10.36504 | | 9.53567 | | 8.43804 | 3.802 up | | 2.140 up | | 1.776 down | PL8 polysaccharide lyase; distantly related to chondroitin lyases |
| 22115 | 0.000597 | 10.13533 | | 11.45086 | | 9.8537 | 1.215 up | | 3.025 up | | 2.488 up | poly polymerase (Poly[ADP-ribose] synthetase) |
| 65128 | 0.00621 | 10.23563 | | 11.83856 | | 10.44564 | 1.156 down | | 2.626 up | | 3.037 up | polynucleotide kinase 3 phosphatase |
| 2474 | 0.000274 | 10.84793 | | 12.65274 | | 10.62837 | 1.164 up | | 4.068 up | | 3.493 up | polyphosphoinositide phosphatase, putative |
| 55723 | 0.000156 | 8.56706 | | 10.06573 | | 5.17821 | 10.474 up | | 29.599 up | | 2.825 up | PotE |
| 60551 | 0.0121 | 9.55606 | | 11.0816 | | 9.66362 | 1.077 down | | 2.672 up | | 2.878 up | PotE Amino acid transporters |
| 54260 | 0.000102 | 9.83792 | | 11.90569 | | 9.21711 | 1.537 up | | 6.446 up | | 4.192 up | potential indoleamine 2,3-dioxygenase |
| 55868 | 0.00103 | 8.27674 | | 11.66618 | | 9.41634 | 2.203 down | | 4.756 up | | 10.479 up | PP2Ac, Protein phosphatase 2A homologues |
| 5659 | 0.00153 | 10.20642 | | 11.46884 | | 9.82832 | 1.299 up | | 3.117 up | | 2.398 up | pre-mRNA splicing factor, putative [Cryptococcus neoformans]. |
| 120571 | 0.00606 | 9.68291 | | 11.25461 | | 9.4312 | 1.190 up | | 3.539 up | | 2.972 up | pre-rRNA-processing protein PNO1 |
| 61304 | 0.00262 | 6.90283 | | 9.23513 | | 6.82511 | 1.055 up | | 5.314 up | | 5.036 up | Prolidase and Aminopeptidase P |
| 69217 | 0.00534 | 9.16997 | | 10.99351 | | 9.56063 | 1.310 down | | 2.699 up | | 3.539 up | protease-like protein |
| 22093 | 0.000357 | 12.87984 | | 10.6812 | | 11.12989 | 3.363 up | | 1.364 down | | 4.590 down | Protein farnesyltransferase, alpha subunit |
| 35867 | 0.00461 | 11.34877 | | 12.55149 | | 11.11509 | 1.175 up | | 2.706 up | | 2.301 up | Protein interacting with poly(A)-binding protein |
| 45652 | 0.00133 | 10.2484 | | 10.49787 | | 8.79635 | 2.735 up | | 3.252 up | | 1.188 up | Protein kinase |
| 53776 | 0.00338 | 11.50618 | | 11.85129 | | 10.3277 | 2.263 up | | 2.875 up | | 1.270 up | Protein kinase |
| 106487 | 0.00297 | 6.44946 | | 5.71989 | | 5.12253 | 2.508 up | | 1.512 up | | 1.658 down | Protein kinase |
| 120806 | 0.000357 | 10.1644 | | 12.80292 | | 10.77534 | 1.527 down | | 4.077 up | | 6.226 up | protein kinase |
| 52021 | 0.00105 | 10.25471 | | 12.58908 | | 10.70246 | 1.363 down | | 3.697 up | | 5.043 up | protein kinase |
| 55719 | 0.000692 | 5.40409 | | 7.83468 | | 5.80111 | 1.316 down | | 4.094 up | | 5.391 up | Protein kinase |
| 106939 | 0.000778 | 5.25918 | | 9.27833 | | 4.86787 | 1.311 up | | 21.265 up | | 16.213 up | protein kinase |
| 122735 | 0.0159 | 10.82946 | | 12.80379 | | 11.1642 | 1.261 down | | 3.115 up | | 3.929 up | protein kinase |
| 120605 | 0.00458 | 12.04049 | | 13.39969 | | 12.00233 | 1.026 up | | 2.634 up | | 2.565 up | protein kinase Rim15, response regulator receiver p |
| 67982 | 0.00879 | 11.14108 | | 12.51402 | | 11.04077 | 1.072 up | | 2.776 up | | 2.589 up | protein kinase WEE1, cell cycle control |
| 62181 | 0.00057 | 12.10399 | | 12.50597 | | 10.9123 | 2.284 up | | 3.018 up | | 1.321 up | Protein kinase, Ca2+-dependent |
| 71315 | 0.00408 | 11.04084 | | 12.13585 | | 10.74663 | 1.226 up | | 2.619 up | | 2.136 up | protein kinase, related to S. cerevisiae Cla4p, a member of the PAK family |
| 62706 | 0.00113 | 6.24569 | | 8.14298 | | 5.77243 | 1.388 up | | 5.171 up | | 3.725 up | protein phosphatase |
| 57129 | 0.00108 | 11.58096 | | 12.9767 | | 11.47167 | 1.078 up | | 2.838 up | | 2.631 up | Protein transport protein Yos1 |
| 3027 | 0.00252 | 10.06944 | | 11.53743 | | 9.78291 | 1.219 up | | 3.374 up | | 2.766 up | Pso2 (Snm1), involved in DNA interstrand crosslink repair |
| 110744 | 0.00161 | 12.24118 | | 12.25174 | | 10.91384 | 2.509 up | | 2.527 up | | 1.007 up | PTH11 GPCR |
| 69904 | 0.000255 | 12.38511 | | 12.19023 | | 9.59479 | 6.917 up | | 6.043 up | | 1.144 down | PTH11 GPCR |
| 103694 | 0.00158 | 11.90161 | | 12.25826 | | 10.75633 | 2.211 up | | 2.832 up | | 1.280 up | PTH11 GPCR |
| 107042 | 0.000967 | 6.98907 | | 7.78483 | | 4.08978 | 7.460 up | | 12.951 up | | 1.735 up | PTH11 GPCR |
| 55561 | 0.00191 | 10.46247 | | 11.66655 | | 8.51983 | 3.844 up | | 8.856 up | | 2.303 up | PTH11 GPCR |
| 27992 | 0.000138 | 10.74893 | | 13.29903 | | 8.45318 | 4.910 up | | 28.757 up | | 5.856 up | PTH11 GPCR |
| 109146 | 0.000175 | 9.86795 | | 4.13661 | | 3.36254 | 90.849 up | | 1.710 up | | 53.125 down | PTH11 GPCR |
| 69500 | 0.0061 | 5.57005 | | 8.88105 | | 6.897 | 2.508 down | | 3.956 up | | 9.924 up | PTH11 GPCR |
| 78499 | 0.000163 | 11.83478 | | 13.12762 | | 11.06816 | 1.701 up | | 4.168 up | | 2.450 up | PTH11 GPCR |
| 57101 | 0.00311 | 4.84348 | | 6.62326 | | 5.051 | 1.154 down | | 2.973 up | | 3.433 up | PTH11 GPCR |
| 27983 | 0.00213 | 3.58767 | | 5.00221 | | 3.5018 | 1.061 up | | 2.829 up | | 2.665 up | PTH11 GPCR |
| 77689 | 0.00047 | 10.52057 | | 12.66635 | | 10.97851 | 1.373 down | | 3.221 up | | 4.425 up | PTPc, Protein tyrosine phosphatases (PTP) |
| 77025 | 0.000679 | 10.78773 | | 12.7307 | | 11.17496 | 1.307 down | | 2.939 up | | 3.844 up | P-type ATPase with putative aminophospholipid translocase activity, neo1 |
| 121308 | 0.000053 | 12.30753 | | 14.12964 | | 7.69928 | 24.390 up | | 86.244 up | | 3.535 up | PutA delta-1-pyrroline-5-carboxylate dehydrogenase |
| 5206 | 0.00328 | 8.2626 | | 10.66213 | | 8.15928 | 1.074 up | | 5.668 up | | 5.276 up | pyridine nucleotide-disulphide oxidoreductase AMID-like |
| 39588 | 0.000177 | 6.94007 | | 7.96254 | | 5.77292 | 2.245 up | | 4.561 up | | 2.031 up | Pyridine nucleotide-disulphide oxidoreductase, |
| 59267 | 0.00127 | 6.97517 | | 8.26517 | | 4.94678 | 4.079 up | | 9.975 up | | 2.445 up | pyruvate decarboxylase |
| 68291 | 0.00254 | 9.89716 | | 11.296 | | 9.77848 | 1.085 up | | 2.862 up | | 2.636 up | pyruvate formate lyase activating enzyme (radical SAM superfamily) |
| 67742 | 0.00268 | 11.25936 | | 12.35125 | | 10.79962 | 1.375 up | | 2.931 up | | 2.131 up | QDE1, RdRP, essential for quelling |
| 102458 | 0.00159 | 11.69163 | | 12.87723 | | 11.2306 | 1.376 up | | 3.130 up | | 2.274 up | QDE3, RecQ helicase, essential for quelling |
| 57424 | 0.000769 | 9.29829 | | 9.87671 | | 8.44022 | 1.812 up | | 2.706 up | | 1.493 up | QIP, Putative exonuclease protein, involved in quelling |
| 80091 | 0.0000689 | 10.61956 | | 11.96538 | | 5.42717 | 36.565 up | | 92.939 up | | 2.541 up | quinate permease |
| 69692 | 0.000307 | 4.44363 | | 11.04161 | | 5.56537 | 2.176 down | | 44.515 up | | 96.870 up | Quinoprotein amine dehydrogenase beta chain-like protein |
| 44278 | 0.000107 | 15.14676 | | 13.92458 | | 13.75149 | 2.630 up | | 1.127 up | | 2.332 down | Rab geranyl transferase escort protein |
| 119879 | 0.00394 | 10.41415 | | 11.60103 | | 10.1615 | 1.191 up | | 2.712 up | | 2.276 up | Rab geranylgeranyl transferase escort protein, putative |
| 57198 | 0.0122 | 10.38608 | | 10.92128 | | 9.47811 | 1.876 up | | 2.719 up | | 1.449 up | Rad10 |
| 69998 | 0.000884 | 9.34475 | | 10.93379 | | 9.3358 | 1.006 up | | 3.027 up | | 3.008 up | Rad14, binsd to damaged DNA during nucleotide excisionRepair |
| 121381 | 0.0011 | 10.54788 | | 12.47258 | | 10.6511 | 1.074 down | | 3.534 up | | 3.796 up | Rad16 |
| 2089 | 0.00468 | 6.17049 | | 6.6679 | | 5.07492 | 2.136 up | | 3.016 up | | 1.411 up | Rad21/Rec8 like protein, N-terminal |
| 75205 | 0.00971 | 11.19921 | | 11.95898 | | 10.48073 | 1.645 up | | 2.786 up | | 1.693 up | Rad4p |
| 61075 | 0.000177 | 9.70662 | | 11.43782 | | 9.89598 | 1.140 down | | 2.911 up | | 3.320 up | Rad4p, involved in nucleotide excisionRepair. |
| 103655 | 0.000173 | 8.44487 | | 10.64163 | | 8.85107 | 1.325 down | | 3.459 up | | 4.584 up | rad55 |
| 58631 | 0.000608 | 10.38714 | | 12.29609 | | 9.93878 | 1.364 up | | 5.124 up | | 3.755 up | Rad5p, a ubiquitin-protein ligase that functions in DNA damage tolerance |
| 60115 | 0.00171 | 11.07348 | | 12.36672 | | 10.79258 | 1.214 up | | 2.977 up | | 2.450 up | Rad9, DNA damage sensor. |
| 58804 | 0.00127 | 11.4571 | | 12.30222 | | 10.9713 | 1.400 up | | 2.515 up | | 1.796 up | Rad9, that functions in DNARepair |
| 53995 | 0.00118 | 10.03256 | | 11.09597 | | 9.61343 | 1.337 up | | 2.794 up | | 2.089 up | RAI1 |
| 107035 | 0.00332 | 8.23773 | | 9.8153 | | 8.28864 | 1.035 down | | 2.881 up | | 2.984 up | RAS small GTPase,Ras subfamily |
| 5278 | 0.00116 | 10.1527 | | 11.67682 | | 8.96365 | 2.280 up | | 6.557 up | | 2.876 up | Ras small GTPase,Rho type |
| 67275 | 0.00115 | 11.2781 | | 13.80086 | | 10.62816 | 1.569 up | | 9.017 up | | 5.746 up | RAS1 |
| 41009 | 0.00013 | 9.17795 | | 12.02199 | | 9.35623 | 1.131 down | | 6.345 up | | 7.180 up | Ras-like GTPase, Rho type |
| 105031 | 0.00174 | 10.23058 | | 11.48879 | | 9.32902 | 1.868 up | | 4.468 up | | 2.391 up | RecQ subfamily of DNA helicases |
| 110535 | 0.00122 | 8.39922 | | 9.30348 | | 7.19122 | 2.310 up | | 4.323 up | | 1.871 up | Regulation of HR towards crossover |
| 105409 | 0.000197 | 10.29412 | | 12.64554 | | 11.14617 | 1.805 down | | 2.827 up | | 5.103 up | regulator of MAPKKK STE50 |
| 121171 | 0.00126 | 8.75532 | | 10.64491 | | 8.73553 | 1.013 up | | 3.756 up | | 3.705 up | replication fork protection component Swi3 |
| 111053 | 0.00205 | 12.40578 | | 10.98029 | | 10.79485 | 3.054 up | | 1.137 up | | 2.686 down | retrograde regulation protein 2 |
| 104451 | 0.000291 | 8.14916 | | 10.07302 | | 7.94538 | 1.151 up | | 4.370 up | | 3.794 up | retrograde regulation protein 2 |
| 75518 | 0.00105 | 8.88048 | | 11.35825 | | 9.27089 | 1.310 down | | 4.249 up | | 5.570 up | REV1 deoxycytidyl transferase involved in DNA translesion synthesis |
| 73190 | 0.000522 | 9.41055 | | 11.42053 | | 9.25325 | 1.115 up | | 4.491 up | | 4.027 up | Rev3p of Saccharomyces cerevisiae, the catalytic subunit of DNA polymerase zeta |
| 44459 | 0.000506 | 10.42027 | | 12.03688 | | 10.56965 | 1.109 down | | 2.764 up | | 3.066 up | Rfc4p (replication factor C) subunit 4 |
| 75210 | 0.00226 | 10.87443 | | 12.53639 | | 10.76609 | 1.077 up | | 3.411 up | | 3.164 up | RhoGEF domain-containing protein |
| 103563 | 0.000143 | 8.83729 | | 10.66347 | | 9.26038 | 1.340 down | | 2.644 up | | 3.545 up | riboflavin biosynthesis protein Rib7 |
| 82560 | 0.00167 | 8.4351 | | 11.14564 | | 9.32819 | 1.857 down | | 3.524 up | | 6.545 up | Ribokinase |
| 4213 | 0.000755 | 13.59582 | | 11.91111 | | 10.51621 | 8.453 up | | 2.629 up | | 3.214 down | ribonuclease T2 |
| 2489 | 0.000532 | 6.69951 | | 8.54485 | | 6.49657 | 1.151 up | | 4.136 up | | 3.593 up | Ribonuclease T2 |
| 102964 | 0.000669 | 7.68234 | | 9.23094 | | 7.0121 | 1.591 up | | 4.655 up | | 2.925 up | ribosomal protein S2. |
| 67504 | 0.00105 | 8.16336 | | 11.37722 | | 7.83881 | 1.252 up | | 11.618 up | | 9.278 up | ribosomal protein S2. |
| 65615 | 0.000133 | 12.63565 | | 11.59072 | | 10.24622 | 5.239 up | | 2.539 up | | 2.063 down | RibosomalRNA methyltransferaseRrmJ/FtsJ domain. |
| 1983 | 0.00315 | 10.00884 | | 10.83915 | | 9.0183 | 1.986 up | | 3.532 up | | 1.778 up | RNA binding domain protein PUA |
| 56003 | 0.00027 | 7.18134 | | 9.39775 | | 5.14668 | 4.097 up | | 19.041 up | | 4.647 up | RNA binding protein MSSP-2 |
| 75260 | 0.000197 | 11.33053 | | 11.45425 | | 9.74854 | 2.993 up | | 3.261 up | | 1.089 up | RNA polymerase H/23 kD subunit |
| 63379 | 0.00195 | 9.2791 | | 11.80343 | | 9.17126 | 1.077 up | | 6.199 up | | 5.753 up | RNA polymerase II transcriptional coactivator, putative |
| 43961 | 0.000442 | 10.31953 | | 11.58717 | | 10.09243 | 1.170 up | | 2.818 up | | 2.407 up | RNA polymerase III transcription initiation factor complex (TFIIIC) subunit |
| 120801 | 0.000371 | 10.9818 | | 12.8857 | | 11.47452 | 1.407 down | | 2.659 up | | 3.742 up | RNA recognition domain protein |
| 49048 | 0.000515 | 10.9142 | | 10.56731 | | 8.75211 | 4.475 up | | 3.519 up | | 1.271 down | RNA-dependent RNA-polymerase |
| 55349 | 0.000448 | 8.97791 | | 10.31129 | | 8.58207 | 1.315 up | | 3.315 up | | 2.519 up | RNAse P Rpr2/Rpp21/SNM1 subunit domain-containing protein, putative |
| 54419 | 0.00312 | 10.05704 | | 10.96118 | | 9.43327 | 1.540 up | | 2.883 up | | 1.871 up | RNase3 domain-containing protein |
| 70127 | 0.0112 | 5.54692 | | 4.47742 | | 3.78753 | 3.385 up | | 1.613 up | | 2.098 down | RTA1 domain protein, putative |
| 41325 | 0.000453 | 7.93339 | | 5.80771 | | 5.84507 | 4.252 up | | 1.026 down | | 4.364 down | RTA1 like protein; 7 TM |
| 22117 | 0.00136 | 12.41945 | | 13.37233 | | 11.94324 | 1.391 up | | 2.692 up | | 1.935 up | RUM1 repressor of b mating type related genes, possibly involved in fruiting body formation |
| 122529 | 0.000803 | 10.63876 | | 12.54115 | | 10.42275 | 1.161 up | | 4.342 up | | 3.738 up | S1/P1 Nuclease |
| 65156 | 0.000388 | 6.32471 | | 8.71484 | | 6.4051 | 1.057 down | | 4.957 up | | 5.242 up | S1/P1 nuclease |
| 77685 | 0.000552 | 11.57978 | | 13.06869 | | 11.27108 | 1.238 up | | 3.476 up | | 2.806 up | SAGA complex bromodomain subunit Spt7, putative |
| 58521 | 0.00175 | 3.4785 | | 5.96415 | | 4.05802 | 1.494 down | | 3.748 up | | 5.600 up | SAM (and some other nucleotide) binding motif |
| 124331 | 0.000682 | 9.76661 | | 11.25057 | | 9.2987 | 1.383 up | | 3.868 up | | 2.797 up | SAM dependent methyltransferase |
| 70355 | 0.000368 | 13.53286 | | 11.11185 | | 10.00256 | 11.553 up | | 2.157 up | | 5.355 down | SAM-dependent methylransferase |
| 60758 | 0.014 | 11.63728 | | 10.77735 | | 10.22665 | 2.658 up | | 1.464 up | | 1.814 down | SAM-dependent methyltransferase |
| 68348 | 0.000131 | 8.57042 | | 12.45246 | | 9.79564 | 2.337 down | | 6.306 up | | 14.743 up | SAM-dependent methyltransferase |
| 65711 | 0.000398 | 12.41159 | | 13.89988 | | 11.63158 | 1.717 up | | 4.817 up | | 2.805 up | SAM-dependent methyltransferase |
| 4442 | 0.00159 | 8.19075 | | 10.92003 | | 7.72629 | 1.379 up | | 9.149 up | | 6.631 up | SAM-dependent methyltransferase |
| 104393 | 0.00145 | 7.4002 | | 8.62553 | | 7.20489 | 1.144 up | | 2.677 up | | 2.338 up | SAM-dependent methyltransferase |
| 59381 | 0.000806 | 11.0649 | | 10.53456 | | 7.99553 | 8.394 up | | 5.811 up | | 1.444 down | SAM-dependent methyltransferases |
| 111110 | 0.000156 | 7.89338 | | 10.34688 | | 6.0676 | 3.544 up | | 19.417 up | | 5.477 up | SAM-dependent methyltransferases |
| 56236 | 0.00065 | 6.41725 | | 11.76481 | | 9.63369 | 9.294 down | | 4.380 up | | 40.717 up | SAM-dependent methyltransferases |
| 121166 | 0.000742 | 11.75397 | | 12.69833 | | 10.83623 | 1.889 up | | 3.635 up | | 1.924 up | SAM-dependent methyltransferases |
| 39911 | 0.00595 | 10.80399 | | 12.01283 | | 10.37361 | 1.347 up | | 3.114 up | | 2.311 up | SAM-dependent methyltransferases |
| 53428 | 0.000682 | 9.5676 | | 9.5526 | | 7.47566 | 4.263 up | | 4.219 up | | 1.010 down | SAM-dependent metyhltransferase |
| 27384 | 0.000115 | 11.26941 | | 13.00798 | | 10.84205 | 1.344 up | | 4.487 up | | 3.337 up | SCF E3 ubiquitin ligase complex F-box protein grr1, putative |
| 55774 | 0.000371 | 9.7191 | | 12.34288 | | 9.75814 | 1.027 down | | 5.999 up | | 6.163 up | Secretion related small GTPase |
| 108202 | 0.00109 | 13.00001 | | 13.14603 | | 11.72821 | 2.414 up | | 2.671 up | | 1.106 up | Secretory protein Ssp120, Ca2+ binding |
| 61127 | 0.000298 | 13.20817 | | 11.20696 | | 11.36338 | 3.592 up | | 1.114 down | | 4.003 down | Serine carboxypeptidase |
| 69489 | 0.00212 | 11.58671 | | 13.30979 | | 11.60592 | 1.013 down | | 3.257 up | | 3.301 up | serine carboxypeptidase |
| 108715 | 0.00204 | 6.10927 | | 6.33891 | | 4.63818 | 2.772 up | | 3.250 up | | 1.172 up | serine peptidase S28 |
| 121968 | 0.00175 | 11.79413 | | 12.25381 | | 9.59842 | 4.581 up | | 6.300 up | | 1.375 up | serine peptidase S28 |
| 106315 | 0.00254 | 7.18993 | | 9.56914 | | 6.97711 | 1.158 up | | 6.029 up | | 5.202 up | serine protease |
| 119822 | 0.00545 | 13.49628 | | 13.68282 | | 12.12069 | 2.594 up | | 2.952 up | | 1.138 up | Serine/threonine protein kinase |
| 112669 | 0.000908 | 10.73197 | | 10.40868 | | 8.2776 | 5.480 up | | 4.380 up | | 1.251 down | Serine/threonine protein kinase |
| 123672 | 0.000369 | 12.20082 | | 12.62955 | | 11.11585 | 2.121 up | | 2.855 up | | 1.346 up | Serine/Threonine protein kinase |
| 78639 | 0.000268 | 11.45344 | | 13.64226 | | 9.06103 | 5.250 up | | 23.938 up | | 4.559 up | Serine/threonine protein kinase |
| 64125 | 0.00539 | 4.88627 | | 7.44678 | | 4.02934 | 1.811 up | | 10.684 up | | 5.899 up | Serine/threonine protein kinase |
| 123313 | 0.00122 | 13.22799 | | 14.17095 | | 12.46949 | 1.691 up | | 3.252 up | | 1.922 up | Serine/threonine protein kinase |
| 58476 | 0.000723 | 10.65702 | | 11.68655 | | 10.14316 | 1.427 up | | 2.914 up | | 2.041 up | serine/threonine protein kinase |
| 81219 | 0.00187 | 11.01645 | | 12.5472 | | 10.56249 | 1.369 up | | 3.957 up | | 2.889 up | Serine/threonine protein kinase |
| 119616 | 0.000211 | 10.94638 | | 12.58471 | | 10.84014 | 1.076 up | | 3.350 up | | 3.113 up | Serine/threonine protein kinase |
| 121944 | 0.000697 | 13.07625 | | 14.28402 | | 12.03938 | 2.051 up | | 4.739 up | | 2.309 up | serine/threonine protein kinase |
| 21306 | 0.00299 | 12.39196 | | 12.92684 | | 11.56453 | 1.774 up | | 2.570 up | | 1.448 up | serine/threonine protein kinase PRP4 |
| 122015 | 0.00161 | 11.45467 | | 12.38401 | | 10.64122 | 1.757 up | | 3.346 up | | 1.904 up | Serine/threonine protein kinase required for receptor-mediated endocytosis |
| 55543 | 0.015 | 10.3181 | | 10.1279 | | 8.51258 | 3.495 up | | 3.063 up | | 1.140 down | serine/threonine protein kinase with similarity to casein kinase I |
| 66950 | 0.0269 | 9.60334 | | 10.14825 | | 8.69046 | 1.882 up | | 2.746 up | | 1.458 up | serine/threonine protein kinase, |
| 103754 | 0.000546 | 8.32366 | | 11.9133 | | 8.19845 | 1.090 up | | 13.130 up | | 12.038 up | serine/threonine protein kinase, PAK/STE20 subfamily |
| 102711 | 0.00398 | 12.75166 | | 13.42752 | | 12.07795 | 1.595 up | | 2.548 up | | 1.597 up | serine/threonine-protein kinase, putative |
| 105891 | 0.000516 | 11.15525 | | 12.37033 | | 9.97623 | 2.264 up | | 5.256 up | | 2.321 up | serine-threonine kinase receptor-associated protein |
| 80761 | 0.0194 | 8.59217 | | 10.58929 | | 9.13976 | 1.461 down | | 2.731 up | | 3.992 up | seryl-tRNA synthetase, class IIa. |
| 65039 | 0.00467 | 11.72156 | | 11.97026 | | 10.33625 | 2.612 up | | 3.103 up | | 1.188 up | sexual development protein |
| 59364 | 0.000183 | 13.68065 | | 10.23373 | | 8.03621 | 50.019 up | | 4.586 up | | 10.905 down | Sexual differentiation process protein ISP4 |
| 109687 | 0.000182 | 10.20641 | | 10.2856 | | 6.18717 | 16.214 up | | 17.129 up | | 1.056 up | Shikimate dehydrogenase |
| 65869 | 0.000131 | 9.50741 | | 10.42837 | | 3.33464 | 72.142 up | | 136.591 up | | 1.893 up | Shikimate kinase |
| 60517 | 0.0000752 | 11.39865 | | 11.28642 | | 6.25306 | 35.398 up | | 32.748 up | | 1.080 down | short chain dehydrogenase/reductase |
| 4682 | 0.000115 | 11.93869 | | 11.66542 | | 8.16442 | 13.682 up | | 11.321 up | | 1.208 down | short chain dehydrogenase/reductase |
| 70334 | 0.000127 | 10.11188 | | 9.68751 | | 5.16415 | 30.861 up | | 22.996 up | | 1.341 down | short chain dehydrogenase/reductase |
| 30668 | 0.000341 | 10.13347 | | 10.91767 | | 9.10586 | 2.038 up | | 3.510 up | | 1.722 up | short chain dehydrogenase/reductase |
| 4990 | 0.00118 | 8.33961 | | 7.43248 | | 5.92857 | 5.318 up | | 2.836 up | | 1.875 down | short chain dehydrogenase/reductase |
| 106164 | 0.0000276 | 10.81427 | | 11.96821 | | 4.45084 | 82.334 up | | 183.210 up | | 2.225 up | short chain dehydrogenase/reductase |
| 3055 | 0.000324 | 12.4797 | | 10.80841 | | 8.15379 | 20.055 up | | 6.296 up | | 3.184 down | short chain dehydrogenase/reductase |
| 77202 | 0.000349 | 11.21977 | | 9.39794 | | 7.78677 | 10.800 up | | 3.055 up | | 3.535 down | short chain dehydrogenase/reductase |
| 123079 | 0.000146 | 11.06076 | | 14.57635 | | 6.23608 | 28.338 up | | 324.094 up | | 11.436 up | short chain dehydrogenase/reductase |
| 22771 | 0.0011 | 9.06286 | | 10.48285 | | 7.87441 | 2.279 up | | 6.098 up | | 2.675 up | short chain dehydrogenase/reductase |
| 67938 | 0.0000976 | 7.50738 | | 9.10836 | | 6.33618 | 2.251 up | | 6.831 up | | 3.033 up | short chain dehydrogenase/reductase |
| 122079 | 0.00111 | 11.28217 | | 12.96814 | | 9.87578 | 2.650 up | | 8.528 up | | 3.217 up | short chain dehydrogenase/reductase |
| 59698 | 0.00532 | 8.0589 | | 8.01789 | | 6.70014 | 2.564 up | | 2.492 up | | 1.028 down | short chain dehydrogenase/reductase |
| 58479 | 0.00191 | 7.76758 | | 6.59335 | | 6.15486 | 3.058 up | | 1.355 up | | 2.256 down | short chain dehydrogenase/reductase |
| 75383 | 0.00477 | 12.90333 | | 11.14189 | | 11.07765 | 3.544 up | | 1.045 up | | 3.390 down | short chain dehydrogenase/reductase |
| 68336 | 0.000483 | 4.57975 | | 6.89048 | | 5.50521 | 1.899 down | | 2.612 up | | 4.961 up | short chain dehydrogenase/reductase |
| 69840 | 0.0402 | 5.99064 | | 6.9271 | | 5.24624 | 1.675 up | | 3.206 up | | 1.913 up | short chain dehydrogenase/reductase |
| 104179 | 0.0025 | 6.60268 | | 7.59831 | | 5.88035 | 1.649 up | | 3.289 up | | 1.993 up | short chain dehydrogenase/reductase |
| 53331 | 0.000378 | 9.89789 | | 11.61959 | | 9.24315 | 1.574 up | | 5.192 up | | 3.298 up | short chain dehydrogenase/reductase |
| 65021 | 0.00332 | 5.65099 | | 6.80394 | | 5.00619 | 1.563 up | | 3.476 up | | 2.223 up | short chain dehydrogenase/reductase |
| 54223 | 0.000236 | 7.24898 | | 9.04932 | | 6.80882 | 1.356 up | | 4.725 up | | 3.483 up | short chain dehydrogenase/reductase |
| 2200 | 0.000547 | 5.90337 | | 7.96476 | | 6.04939 | 1.106 down | | 3.772 up | | 4.173 up | short chain dehydrogenase/reductase |
| 123627 | 0.000647 | 11.90326 | | 13.3251 | | 11.89721 | 1.004 up | | 2.690 up | | 2.679 up | short chain dehydrogenase/reductase |
| 66117 | 0.000308 | 11.64633 | | 11.56529 | | 9.84484 | 3.485 up | | 3.295 up | | 1.057 down | short chain dehydrogenase/reductase |
| 123553 | 0.000354 | 8.77966 | | 9.73845 | | 7.01378 | 3.400 up | | 6.610 up | | 1.943 up | short chain dehydrogenase/reductase |
| 58066 | 0.00146 | 9.9855 | | 11.21064 | | 8.63683 | 2.546 up | | 5.953 up | | 2.337 up | short chain dehydrogenase/reductase |
| 2902 | 0.0149 | 9.82375 | | 9.20406 | | 8.2645 | 2.946 up | | 1.917 up | | 1.536 down | short chain dehydrogenase/reductase |
| 5368 | 0.00372 | 4.70894 | | 6.65139 | | 3.8371 | 1.829 up | | 7.033 up | | 3.843 up | short chain dehydrogenase/reductase |
| 78248 | 0.00129 | 11.10188 | | 12.2023 | | 10.31469 | 1.725 up | | 3.700 up | | 2.144 up | short chain dehydrogenase/reductase |
| 5112 | 0.000527 | 6.92782 | | 7.87683 | | 6.21507 | 1.638 up | | 3.164 up | | 1.930 up | short chain dehydrogenase/reductase |
| 58672 | 0.00453 | 6.69078 | | 7.63666 | | 5.99856 | 1.615 up | | 3.112 up | | 1.926 up | short chain dehydrogenase/reductase |
| 105970 | 0.000232 | 10.67029 | | 13.49835 | | 10.21156 | 1.374 up | | 9.759 up | | 7.101 up | short chain dehydrogenase/reductase |
| 54550 | 0.000356 | 9.42957 | | 9.90705 | | 7.48278 | 3.855 up | | 5.367 up | | 1.392 up | short chain dehydrognease/reductase |
| 54086 | 0.00187 | 13.5857 | | 13.09865 | | 11.46011 | 4.363 up | | 3.113 up | | 1.401 down | short chain dehydrognease/reductase |
| 106516 | 0.00762 | 7.35356 | | 8.03272 | | 6.36752 | 1.980 up | | 3.171 up | | 1.601 up | short chain dehydrognease/reductase |
| 52718 | 0.000281 | 10.30674 | | 11.52137 | | 9.42174 | 1.846 up | | 4.285 up | | 2.320 up | short chain dehydrognease/reductase |
| 107962 | 0.000153 | 8.87524 | | 11.47124 | | 8.0101 | 1.821 up | | 11.013 up | | 6.046 up | short chain dehydrognease/reductase |
| 121478 | 0.0017 | 5.35503 | | 7.889 | | 5.85869 | 1.417 down | | 4.084 up | | 5.791 up | short chain dehydrognease/reductase |
| 104059 | 0.0109 | 3.45001 | | 5.29984 | | 3.78322 | 1.259 down | | 2.861 up | | 3.604 up | short chain dehydrognease/reductase |
| 65588 | 0.000614 | 5.93363 | | 8.55559 | | 5.7102 | 1.167 up | | 7.187 up | | 6.155 up | short chain dehydrognease/reductase |
| 111541 | 0.000745 | 6.57867 | | 7.89544 | | 6.54827 | 1.021 up | | 2.544 up | | 2.491 up | short chain dehydrognease/reductase |
| 123616 | 0.000402 | 13.85401 | | 14.56343 | | 10.84973 | 8.023 up | | 13.120 up | | 1.635 up | short unique protein |
| 80019 | 0.00026 | 13.35295 | | 14.47862 | | 10.56391 | 6.911 up | | 15.081 up | | 2.182 up | short-chain dehydrogenase/reductase |
| 70429 | 0.000134 | 9.0725 | | 10.64014 | | 7.29586 | 3.426 up | | 10.156 up | | 2.964 up | Short-chain dehydrogenase/reductase |
| 104557 | 0.000298 | 10.32295 | | 7.78347 | | 8.43031 | 3.713 up | | 1.565 down | | 5.813 down | short-chain dehydrogenase/reductase |
| 60739 | 0.00141 | 6.34987 | | 7.34224 | | 5.41372 | 1.913 up | | 3.806 up | | 1.989 up | short-chain dehydrogenase/reductase |
| 122556 | 0.000723 | 7.36928 | | 11.42732 | | 7.43466 | 1.046 down | | 15.918 up | | 16.656 up | Short-chain dehydrogenase/reductase |
| 54227 | 0.00156 | 9.66258 | | 9.93534 | | 6.6223 | 8.226 up | | 9.938 up | | 1.208 up | short-chain dehydrogenase/reductase |
| 112590 | 0.000365 | 4.6911 | | 9.17276 | | 3.66708 | 2.033 up | | 45.433 up | | 22.341 up | siderophore biosynthesis lipase/esterase |
| 71008 | 0.00201 | 5.96549 | | 7.88295 | | 4.34458 | 3.075 up | | 11.618 up | | 3.777 up | siderophore transporter |
| 82017 | 0.00102 | 10.65338 | | 11.33816 | | 9.84789 | 1.747 up | | 2.809 up | | 1.607 up | siderophore transporter |
| 78465 | 0.000154 | 11.54411 | | 14.13489 | | 12.31618 | 1.707 down | | 3.527 up | | 6.024 up | siderophore transporter |
| 79377 | 0.000882 | 12.12611 | | 12.09128 | | 10.5823 | 2.915 up | | 2.846 up | | 1.024 down | Sin3 complex subunit Stb2 |
| 107564 | 0.000193 | 9.33524 | | 10.97341 | | 9.17751 | 1.115 up | | 3.472 up | | 3.112 up | Sin3-associated polypeptide Sap18 |
| 5868 | 0.00188 | 11.51942 | | 13.32748 | | 11.94436 | 1.342 down | | 2.608 up | | 3.501 up | Single-stranded nucleic acid binding R3H |
| 61465 | 0.00254 | 10.54338 | | 12.40443 | | 10.02131 | 1.436 up | | 5.216 up | | 3.632 up | SNARE complex subunit Vam7 |
| 106138 | 0.00893 | 8.35506 | | 7.78721 | | 6.87761 | 2.784 up | | 1.878 up | | 1.482 down | SNF2 family DNA-dependent ATPase |
| 22783 | 0.00025 | 11.65886 | | 12.97607 | | 11.59006 | 1.048 up | | 2.613 up | | 2.491 up | SNF2 family DNA-dependent ATPase |
| 2369 | 0.00593 | 9.29621 | | 10.75244 | | 9.32418 | 1.019 down | | 2.691 up | | 2.743 up | SNF2 family domain-containing protein |
| 44747 | 0.00232 | 13.16667 | | 14.11392 | | 11.75925 | 2.652 up | | 5.114 up | | 1.928 up | SNF2 family helicase |
| 109526 | 0.000114 | 8.28029 | | 11.23216 | | 8.55394 | 1.208 down | | 6.400 up | | 7.737 up | SNF2 family helicase/ATPase |
| 2826 | 0.00195 | 8.36563 | | 10.45516 | | 8.808 | 1.358 down | | 3.132 up | | 4.256 up | SNF2-like helicase |
| 45343 | 0.000814 | 10.98741 | | 12.41899 | | 9.75789 | 2.344 up | | 6.325 up | | 2.697 up | Sodium Bile acid symporter family protein |
| 109523 | 0.00309 | 10.84786 | | 12.40095 | | 10.91318 | 1.046 down | | 2.804 up | | 2.934 up | Splicing coactivator SRm160/300, subunit SRm300 |
| 21166 | 0.000895 | 10.82898 | | 11.8861 | | 10.39754 | 1.348 up | | 2.806 up | | 2.080 up | Splicing factor motif |
| 102686 | 0.00107 | 6.88626 | | 7.22739 | | 5.49331 | 2.626 up | | 3.326 up | | 1.266 up | SSCRP |
| 68207 | 0.00144 | 11.15244 | | 10.79235 | | 9.45171 | 3.250 up | | 2.532 up | | 1.283 down | SSCRP |
| 107644 | 0.000781 | 13.3033 | | 12.86512 | | 10.93611 | 5.159 up | | 3.807 up | | 1.354 down | SSCRP |
| 109849 | 0.000896 | 5.85384 | | 6.3593 | | 4.81764 | 2.050 up | | 2.911 up | | 1.419 up | SSCRP |
| 53360 | 0.000085 | 9.97731 | | 10.5627 | | 7.38456 | 6.032 up | | 9.051 up | | 1.500 up | SSCRP |
| 72183 | 0.000987 | 8.58494 | | 9.33875 | | 7.416 | 2.248 up | | 3.791 up | | 1.686 up | SSCRP |
| 122324 | 0.000442 | 11.29565 | | 12.13357 | | 10.13047 | 2.242 up | | 4.008 up | | 1.787 up | SSCRP |
| 102908 | 0.000331 | 13.05558 | | 13.93011 | | 9.40519 | 12.556 up | | 23.021 up | | 1.833 up | SSCRP |
| 106615 | 0.000113 | 8.28752 | | 9.18718 | | 3.71509 | 23.792 up | | 44.387 up | | 1.865 up | SSCRP |
| 103393 | 0.000677 | 6.77226 | | 7.683 | | 4.64824 | 4.359 up | | 8.195 up | | 1.880 up | SSCRP |
| 112508 | 0.00129 | 8.1319 | | 9.08478 | | 6.88936 | 2.366 up | | 4.580 up | | 1.935 up | SSCRP |
| 107971 | 0.00602 | 5.5363 | | 6.56477 | | 4.42181 | 2.165 up | | 4.416 up | | 2.039 up | SSCRP |
| 124259 | 0.000175 | 8.53353 | | 7.31805 | | 4.36281 | 18.010 up | | 7.755 up | | 2.322 down | SSCRP |
| 124277 | 0.0338 | 8.86804 | | 10.14897 | | 7.33811 | 2.887 up | | 7.017 up | | 2.429 up | SSCRP |
| 59151 | 0.000353 | 14.29717 | | 10.80661 | | 9.3263 | 31.360 up | | 2.790 up | | 11.239 down | SSCRP |
| 123199 | 0.000209 | 9.45818 | | 13.19931 | | 6.98307 | 5.560 up | | 74.349 up | | 13.371 up | SSCRP |
| 111915 | 0.000256 | 10.01714 | | 13.88956 | | 4.10905 | 60.050 up | | 879.485 up | | 14.645 up | SSCRP |
| 122422 | 0.000145 | 9.27243 | | 13.26631 | | 8.01646 | 2.388 up | | 38.050 up | | 15.932 up | SSCRP |
| 70919 | 0.000223 | 5.81218 | | 7.72516 | | 4.41379 | 2.636 up | | 9.927 up | | 3.765 up | SSCRP |
| 120697 | 0.000236 | 11.87651 | | 13.82477 | | 5.56516 | 79.415 up | | 306.472 up | | 3.859 up | SSCRP |
| 108663 | 0.000137 | 7.52989 | | 12.49509 | | 4.87221 | 6.310 up | | 197.113 up | | 31.237 up | SSCRP |
| 121739 | 0.000143 | 11.27197 | | 13.43758 | | 9.29001 | 3.950 up | | 17.723 up | | 4.486 up | SSCRP |
| 123236 | 0.0164 | 7.67005 | | 9.84373 | | 4.30822 | 10.280 up | | 46.382 up | | 4.511 up | SSCRP |
| 102851 | 0.00074 | 9.22755 | | 11.64139 | | 7.59754 | 3.095 up | | 16.493 up | | 5.328 up | SSCRP |
| 108261 | 0.000534 | 10.97105 | | 13.43229 | | 8.99524 | 3.933 up | | 21.661 up | | 5.506 up | SSCRP |
| 106371 | 0.000294 | 6.68572 | | 5.03706 | | 3.80411 | 7.369 up | | 2.350 up | | 3.135 down | SSCRP |
| 105844 | 0.00139 | 11.72458 | | 10.08371 | | 9.63961 | 4.242 up | | 1.360 up | | 3.118 down | SSCRP |
| 108349 | 0.00711 | 5.48786 | | 3.95239 | | 3.78995 | 3.244 up | | 1.119 up | | 2.898 down | SSCRP |
| 109828 | 0.000372 | 11.32349 | | 9.58078 | | 9.43671 | 3.698 up | | 1.105 up | | 3.346 down | SSCRP |
| 104050 | 0.000408 | 4.19952 | | 10.2607 | | 6.77027 | 5.941 down | | 11.238 up | | 66.772 up | SSCRP |
| 107007 | 0.000308 | 3.41712 | | 7.75502 | | 5.47754 | 4.171 down | | 4.848 up | | 20.222 up | SSCRP |
| 5026 | 0.000321 | 8.74716 | | 12.66849 | | 10.08977 | 2.536 down | | 5.974 up | | 15.150 up | SSCRP |
| 63526 | 0.000209 | 7.19605 | | 11.04149 | | 8.20154 | 2.007 down | | 7.159 up | | 14.374 up | SSCRP |
| 122127 | 0.0047 | 13.76326 | | 14.48967 | | 12.86822 | 1.859 up | | 3.076 up | | 1.654 up | SSCRP |
| 111495 | 0.000105 | 9.91993 | | 12.64674 | | 9.02978 | 1.853 up | | 12.269 up | | 6.619 up | SSCRP |
| 74060 | 0.032 | 7.87211 | | 10.84224 | | 8.74967 | 1.837 down | | 4.265 up | | 7.836 up | SSCRP |
| 5011 | 0.000603 | 10.64704 | | 12.07295 | | 9.78942 | 1.812 up | | 4.868 up | | 2.686 up | SSCRP |
| 124295 | 0.00121 | 11.8706 | | 12.68045 | | 11.06661 | 1.745 up | | 3.060 up | | 1.753 up | SSCRP |
| 106484 | 0.00345 | 9.1996 | | 10.38966 | | 8.43966 | 1.693 up | | 3.863 up | | 2.281 up | SSCRP |
| 105984 | 0.000435 | 9.21173 | | 11.20714 | | 9.86281 | 1.570 down | | 2.539 up | | 3.987 up | SSCRP |
| 64181 | 0.00168 | 6.49309 | | 8.11326 | | 5.84784 | 1.564 up | | 4.807 up | | 3.074 up | SSCRP |
| 109911 | 0.00186 | 3.92852 | | 5.88685 | | 4.50125 | 1.487 down | | 2.612 up | | 3.886 up | SSCRP |
| 111803 | 0.00399 | 8.86268 | | 11.34353 | | 9.36141 | 1.412 down | | 3.950 up | | 5.582 up | SSCRP |
| 103135 | 0.00145 | 4.57093 | | 7.05676 | | 5.06288 | 1.406 down | | 3.983 up | | 5.601 up | SSCRP |
| 109231 | 0.000402 | 4.17475 | | 8.88339 | | 4.65677 | 1.396 down | | 18.721 up | | 26.148 up | SSCRP |
| 78681 | 0.0088 | 12.3148 | | 13.22068 | | 11.89092 | 1.341 up | | 2.513 up | | 1.873 up | SSCRP |
| 109255 | 0.00195 | 6.95595 | | 8.58798 | | 6.57207 | 1.304 up | | 4.044 up | | 3.099 up | SSCRP |
| 104227 | 0.000257 | 4.10137 | | 11.42646 | | 4.46665 | 1.288 down | | 124.483 up | | 160.351 up | SSCRP |
| 103174 | 0.00853 | 3.04591 | | 4.85909 | | 3.39212 | 1.271 down | | 2.764 up | | 3.514 up | SSCRP |
| 61830 | 0.00613 | 4.6057 | | 6.94046 | | 4.93251 | 1.254 down | | 4.022 up | | 5.044 up | SSCRP |
| 81296 | 0.000243 | 11.41443 | | 13.01188 | | 11.10911 | 1.235 up | | 3.739 up | | 3.026 up | SSCRP |
| 108193 | 0.000942 | 7.21001 | | 9.16402 | | 7.45646 | 1.186 down | | 3.266 up | | 3.874 up | SSCRP |
| 43115 | 0.000576 | 9.52035 | | 11.85162 | | 9.69429 | 1.128 down | | 4.460 up | | 5.032 up | SSCRP |
| 76971 | 0.000395 | 9.3241 | | 11.6618 | | 9.16815 | 1.114 up | | 5.632 up | | 5.054 up | SSCRP |
| 57402 | 0.0000543 | 10.98806 | | 13.04118 | | 11.13415 | 1.106 down | | 3.750 up | | 4.150 up | SSCRP |
| 47733 | 0.000901 | 11.87496 | | 14.08161 | | 12.00116 | 1.091 down | | 4.229 up | | 4.616 up | SSCRP |
| 70806 | 0.000877 | 8.15147 | | 10.13016 | | 8.2782 | 1.091 down | | 3.609 up | | 3.941 up | SSCRP |
| 107347 | 0.00107 | 4.80174 | | 8.2123 | | 4.92277 | 1.087 down | | 9.777 up | | 10.633 up | SSCRP |
| 104181 | 0.000794 | 4.70775 | | 6.22065 | | 4.81959 | 1.080 down | | 2.640 up | | 2.853 up | SSCRP |
| 108418 | 0.000358 | 6.25416 | | 7.84543 | | 6.33413 | 1.056 down | | 2.850 up | | 3.013 up | SSCRP |
| 108543 | 0.000481 | 11.98144 | | 13.82223 | | 12.01322 | 1.022 down | | 3.504 up | | 3.582 up | SSCRP |
| 70542 | 0.000215 | 9.66633 | | 11.36623 | | 9.941 | 1.209 down | | 2.685 up | | 3.248 up | ß-glycosidase (endo-beta-1,3(4)-β-D-glucanase) |
| 58717 | 0.000905 | 7.15516 | | 10.77365 | | 3.78824 | 10.316 up | | 126.711 up | | 12.282 up | ß-lactamase class C |
| 58418 | 0.00272 | 5.83181 | | 6.28214 | | 3.74937 | 4.235 up | | 5.786 up | | 1.366 up | ß-lactamase-like protein |
| 109325 | 0.000163 | 10.43813 | | 13.14477 | | 9.17182 | 2.405 up | | 15.702 up | | 6.528 up | ß-lactamase-like protein |
| 53029 | 0.0392 | 3.86832 | | 4.50443 | | 2.95 | 1.889 up | | 2.937 up | | 1.554 up | ß-lactamase-like protein |
| 107137 | 0.000115 | 13.80499 | | 12.32 | | 10.29579 | 11.386 up | | 4.067 up | | 2.799 down | sterol desaturase family |
| 63159 | 0.00184 | 11.33368 | | 13.31866 | | 11.95925 | 1.542 down | | 2.565 up | | 3.958 up | straitin Pro11 (stalk rot protein) |
| 47635 | 0.00212 | 12.83276 | | 13.79775 | | 12.40846 | 1.341 up | | 2.619 up | | 1.952 up | stress response regulator SrrA |
| 60791 | 0.00018 | 6.62497 | | 10.50565 | | 8.06269 | 2.708 down | | 5.437 up | | 14.729 up | subtilisin like protease |
| 110910 | 0.000655 | 8.00638 | | 10.77691 | | 8.16178 | 1.113 down | | 6.126 up | | 6.823 up | subtilisin like protease |
| 58698 | 0.000144 | 7.41638 | | 9.86237 | | 6.65721 | 1.692 up | | 9.222 up | | 5.448 up | subtilisin like protease (SUB2) |
| 64719 | 0.000308 | 10.24149 | | 11.35318 | | 9.82542 | 1.334 up | | 2.883 up | | 2.160 up | Subtilisin like protease (SUB3) |
| 123234 | 0.000203 | 6.20602 | | 10.94521 | | 3.6518 | 5.873 up | | 156.867 up | | 26.707 up | Subtilisin like protease (SUB9) |
| 51365 | 0.00077 | 14.47014 | | 14.10787 | | 12.92935 | 2.909 up | | 2.263 up | | 1.285 down | Subtilisin-like protease PPRC1 |
| 72321 | 0.0153 | 9.89066 | | 10.56782 | | 8.94093 | 1.931 up | | 3.088 up | | 1.598 up | subtilisin-like serine protease |
| 70383 | 0.000571 | 11.28326 | | 11.27688 | | 8.96717 | 4.979 up | | 4.957 up | | 1.004 down | succinate semialdehyde dehydrogenase, NADP |
| 2223 | 0.000357 | 8.84063 | | 10.82517 | | 8.78748 | 1.037 up | | 4.105 up | | 3.957 up | succinate-CoA ligase, alpha subunit |
| 47603 | 0.00174 | 10.71391 | | 12.32052 | | 10.8022 | 1.063 down | | 2.864 up | | 3.045 up | succinyl-CoA:3-ketoacid-coenzyme A transferase subunit A |
| 111447 | 0.000371 | 9.9285 | | 11.55277 | | 8.51154 | 2.670 up | | 8.231 up | | 3.082 up | Sulfatase |
| 51217 | 0.00257 | 9.17497 | | 11.94288 | | 9.56851 | 1.313 down | | 5.185 up | | 6.811 up | sulfate transporter, putative |
| 76601 | 0.000234 | 12.12319 | | 12.22369 | | 10.33285 | 3.458 up | | 3.708 up | | 1.072 up | Sulfite oxidase, molybdopterin-binding component |
| 106695 | 0.00146 | 10.79111 | | 11.02856 | | 9.34473 | 2.725 up | | 3.212 up | | 1.178 up | Sulfite oxidase, molybdopterin-binding component |
| 62367 | 0.000103 | 11.2697 | | 12.0246 | | 8.20633 | 8.359 up | | 14.106 up | | 1.687 up | Sulfite oxidase, molybdopterin-binding component |
| 123327 | 0.000498 | 13.0037 | | 13.47183 | | 10.77951 | 4.672 up | | 6.463 up | | 1.383 up | SWI-SNF chromatin-remodeling complex protein |
| 123992 | 0.000115 | 11.22247 | | 14.95875 | | 6.99462 | 18.737 up | | 249.714 up | | 13.327 up | swollenin |
| 122736 | 0.00229 | 12.08733 | | 11.70645 | | 10.18152 | 3.747 up | | 2.877 up | | 1.302 down | tagatose bisphosphate aldolase |
| 106473 | 0.00049 | 9.90069 | | 12.12869 | | 10.54693 | 1.565 down | | 2.993 up | | 4.684 up | TATA box binding protein associated factor (TAF) |
| 77767 | 0.000298 | 8.58256 | | 11.53113 | | 9.55434 | 1.961 down | | 3.936 up | | 7.719 up | Taurine catabolism dioxygenase TauD, putative |
| 105238 | 0.000337 | 9.01245 | | 9.31772 | | 6.26118 | 6.733 up | | 8.319 up | | 1.235 up | Taurine catabolism dioxygenase TauD/TfdA |
| 35375 | 0.000208 | 10.69149 | | 12.30912 | | 10.17722 | 1.428 up | | 4.382 up | | 3.068 up | TBC domain protein (GAP) |
| 29716 | 0.000181 | 9.53362 | | 12.94924 | | 10.01423 | 1.395 down | | 7.647 up | | 10.670 up | thiamine-repressible mitochondrial transport protein THI74, putative |
| 42866 | 0.000815 | 12.18271 | | 10.04751 | | 9.96709 | 4.644 up | | 1.057 up | | 4.392 down | thioesterase family protein |
| 55276 | 0.00139 | 9.7049 | | 10.79121 | | 8.5175 | 2.277 up | | 4.835 up | | 2.123 up | thioesterase superfamily protein |
| 68000 | 0.005 | 10.6015 | | 11.68679 | | 9.84662 | 1.687 up | | 3.580 up | | 2.121 up | thiopurine S-methyltransferase, putative |
| 77288 | 0.000194 | 7.936 | | 11.48284 | | 10.10742 | 4.504 down | | 2.594 up | | 11.687 up | Thioredoxin reductase |
| 67446 | 0.00128 | 10.70566 | | 11.57688 | | 8.85436 | 3.608 up | | 6.600 up | | 1.829 up | ThrC Threonine synthase |
| 4622 | 0.00139 | 9.38739 | | 12.0956 | | 10.29322 | 1.873 down | | 3.487 up | | 6.535 up | thymidylate synthase |
| 72521 | 0.00541 | 8.2135 | | 10.20612 | | 8.62551 | 1.330 down | | 2.990 up | | 3.979 up | transcription elongation factor SPT4 |
| 108775 | 0.000457 | 9.09412 | | 11.71657 | | 9.52875 | 1.351 down | | 4.556 up | | 6.157 up | transcription factor AbaA |
| 109619 | 0.00135 | 10.93333 | | 11.79451 | | 10.45927 | 1.389 up | | 2.523 up | | 1.816 up | transcription factor CBF/NF-Y family |
| 73832 | 0.00308 | 11.00965 | | 11.94312 | | 10.3689 | 1.559 up | | 2.977 up | | 1.909 up | transcription factor SipA3 |
| 44781 | 0.00127 | 11.41912 | | 12.19338 | | 10.51203 | 1.875 up | | 3.207 up | | 1.710 up | Transcription initiation factor IID, 18 kDa subunit |
| 103482 | 0.0023 | 11.46912 | | 10.31425 | | 9.46327 | 4.016 up | | 1.803 up | | 2.226 down | transcriptional activator with ariadne RING finger |
| 5664 | 0.000101 | 9.04218 | | 11.47974 | | 9.61512 | 1.487 down | | 3.641 up | | 5.417 up | transcriptional regulator APSES type |
| 54395 | 0.00085 | 9.53497 | | 12.28465 | | 10.47948 | 1.924 down | | 3.494 up | | 6.725 up | Transcriptional regulator FlbA |
| 120127 | 0.00254 | 10.54018 | | 11.39908 | | 9.92949 | 1.526 up | | 2.769 up | | 1.813 up | transcriptional regulator GATA-type zinc finger protein ASD-4 |
| 64175 | 0.000146 | 12.11462 | | 11.70505 | | 10.25804 | 3.621 up | | 2.726 up | | 1.328 down | transcriptional regulator HMG type |
| 54007 | 0.00125 | 7.65142 | | 9.36797 | | 7.38154 | 1.205 up | | 3.962 up | | 3.286 up | transcriptional regulator HMG type |
| 34248 | 0.0101 | 9.50827 | | 11.22366 | | 9.44353 | 1.045 up | | 3.434 up | | 3.283 up | transcriptional regulator HMG type |
| 79414 | 0.000146 | 9.97746 | | 12.2623 | | 9.85344 | 1.089 up | | 5.310 up | | 4.873 up | transcriptional regulator NGG1, histone acetyltransferase subunit-3 like |
| 53484 | 0.000516 | 10.69563 | | 12.42952 | | 11.04444 | 1.273 down | | 2.611 up | | 3.326 up | transcriptional regulator transcriptional regulator APSES type |
| 112330 | 0.000275 | 12.51999 | | 11.28222 | | 10.9621 | 2.944 up | | 1.248 up | | 2.358 down | transcriptional regulator, putative |
| 59740 | 0.00263 | 9.35672 | | 9.57634 | | 7.89969 | 2.745 up | | 3.196 up | | 1.164 up | transcriptional regulator, unknown |
| 53585 | 0.00157 | 11.45802 | | 12.37806 | | 10.59434 | 1.819 up | | 3.443 up | | 1.892 up | transcriptional regulator, unknown |
| 111755 | 0.000696 | 10.28978 | | 11.13871 | | 9.66755 | 1.539 up | | 2.772 up | | 1.801 up | transcriptional regulator, unknown |
| 57957 | 0.00941 | 11.15212 | | 12.68244 | | 11.13136 | 1.014 up | | 2.930 up | | 2.888 up | transcriptional regulator, unknown |
| 123713 | 0.000357 | 12.07365 | | 13.90309 | | 9.73978 | 5.041 up | | 17.917 up | | 3.553 up | transcriptional regulatorMedA, involved in fruiting body development |
| 67907 | 0.00113 | 7.17123 | | 11.19148 | | 8.2052 | 2.047 down | | 7.924 up | | 16.226 up | transfer of mannosylphosphate |
| 31798 | 0.000518 | 9.72154 | | 12.43245 | | 10.34367 | 1.539 down | | 4.253 up | | 6.547 up | transfer of mannosylphosphate |
| 2211 | 0.000325 | 8.13655 | | 10.13482 | | 4.36097 | 13.694 up | | 54.714 up | | 3.995 up | Transketolase |
| 33359 | 0.00196 | 10.04367 | | 11.5285 | | 9.4777 | 1.480 up | | 4.143 up | | 2.798 up | translation initiation factor SUI1 and density-regulated protein. |
| 53316 | 0.00563 | 9.87246 | | 10.61805 | | 9.28843 | 1.499 up | | 2.513 up | | 1.676 up | TRAPP complex component Bet5 |
| 106331 | 0.00121 | 11.50115 | | 12.39902 | | 10.99827 | 1.417 up | | 2.640 up | | 1.863 up | TRAPP complex component Trs120 |
| 71259 | 0.0101 | 11.60905 | | 11.92913 | | 10.44519 | 2.240 up | | 2.797 up | | 1.248 up | triacylglycerol lipase |
| 68606 | 0.00859 | 10.28067 | | 11.20157 | | 9.40478 | 1.835 up | | 3.474 up | | 1.893 up | triose-phosphate isomerase |
| 80911 | 0.00308 | 12.53128 | | 13.14086 | | 11.70869 | 1.768 up | | 2.698 up | | 1.525 up | tripeptide peptidase |
| 73897 | 0.000428 | 9.42005 | | 10.52575 | | 7.37145 | 4.137 up | | 8.903 up | | 2.152 up | Trypsin-like protease |
| 51103 | 0.000464 | 11.12933 | | 13.23001 | | 11.47592 | 1.271 down | | 3.373 up | | 4.289 up | Tryptophan synthase |
| 65741 | 0.000155 | 11.33334 | | 13.4803 | | 9.85747 | 2.781 up | | 12.319 up | | 4.428 up | tryptophanyl-tRNA synthetase |
| 104790 | 0.000304 | 9.7951 | | 11.81411 | | 9.60102 | 1.143 up | | 4.636 up | | 4.053 up | Tubulin gamma chain |
| 21342 | 0.00135 | 12.35079 | | 12.93417 | | 11.33901 | 2.016 up | | 3.021 up | | 1.498 up | TUL1 Golgi-localized RING-finger ubiquitin ligase (E3) |
| 45445 | 0.000292 | 14.39651 | | 14.07874 | | 11.69645 | 6.498 up | | 5.213 up | | 1.246 down | tyrosinase |
| 50793 | 0.00447 | 6.20698 | | 9.4361 | | 7.13074 | 1.897 down | | 4.942 up | | 9.377 up | tyrosinase |
| 61946 | 0.00126 | 10.57318 | | 12.39707 | | 10.33663 | 1.178 up | | 4.171 up | | 3.540 up | tyrosyl-DNA phosphodiesterase, putative |
| 67325 | 0.00349 | 11.86446 | | 10.70071 | | 10.16446 | 3.248 up | | 1.450 up | | 2.240 down | U3 small nucleolar ribonucleoprotein Mpp10 |
| 58321 | 0.00161 | 11.5983 | | 13.19978 | | 10.269 | 2.512 up | | 7.625 up | | 3.034 up | ubiquitin-conjugating enzyme |
| 60000 | 0.000575 | 11.74754 | | 13.60046 | | 11.80209 | 1.038 down | | 3.478 up | | 3.612 up | ubiquitin-conjugating enzyme |
| 51378 | 0.000724 | 12.08107 | | 13.52839 | | 11.74027 | 1.266 up | | 3.453 up | | 2.727 up | Ubiquitin-like modifier-activating enzyme atg-7, putative |
| 43974 | 0.00244 | 10.1851 | | 12.32653 | | 10.61194 | 1.344 down | | 3.282 up | | 4.411 up | UDP-galactose transporter |
| 62182 | 0.00158 | 11.72665 | | 13.71457 | | 11.90459 | 1.131 down | | 3.506 up | | 3.966 up | UDP-glucose:sterol b-glucosyltransferase |
| 74505 | 0.0008 | 10.43862 | | 11.74606 | | 9.78857 | 1.569 up | | 3.883 up | | 2.475 up | UDP-N-acetylglucosamine transferase subunit |
| 29756 | 0.00405 | 9.75268 | | 10.75331 | | 8.94561 | 1.749 up | | 3.500 up | | 2.000 up | UDP-N-acetylglucosamine transporter |
| 81720 | 0.000517 | 11.64932 | | 12.68387 | | 9.91472 | 3.327 up | | 6.817 up | | 2.048 up | Ume5 gen |
| 109486 | 0.00368 | 9.06601 | | 9.07224 | | 7.43318 | 3.101 up | | 3.114 up | | 1.004 up | unique protein |
| 104322 | 0.000193 | 14.11826 | | 14.10341 | | 8.82881 | 39.109 up | | 38.708 up | | 1.010 down | unique protein |
| 121396 | 0.000315 | 12.45524 | | 12.47317 | | 10.46507 | 3.972 up | | 4.022 up | | 1.012 up | unique protein |
| 106958 | 0.000324 | 11.60285 | | 11.5134 | | 9.59269 | 4.028 up | | 3.786 up | | 1.063 down | unique protein |
| 111517 | 0.00688 | 6.31483 | | 6.41489 | | 5.0863 | 2.343 up | | 2.511 up | | 1.071 up | unique protein |
| 123095 | 0.000658 | 6.8047 | | 6.95122 | | 3.11859 | 12.871 up | | 14.247 up | | 1.106 up | unique protein |
| 107141 | 0.00383 | 7.45492 | | 7.64722 | | 5.94843 | 2.841 up | | 3.246 up | | 1.142 up | unique protein |
| 109779 | 0.019 | 9.05134 | | 9.24804 | | 7.27325 | 3.429 up | | 3.930 up | | 1.146 up | unique protein |
| 106584 | 0.0009 | 8.26946 | | 8.48023 | | 6.52865 | 3.342 up | | 3.867 up | | 1.157 up | unique protein |
| 105515 | 0.00253 | 11.50108 | | 11.7381 | | 9.30213 | 4.591 up | | 5.411 up | | 1.178 up | unique protein |
| 109344 | 0.000594 | 5.07147 | | 5.34383 | | 3.74047 | 2.515 up | | 3.038 up | | 1.207 up | unique protein |
| 112277 | 0.000951 | 6.1394 | | 5.82627 | | 4.38459 | 3.374 up | | 2.716 up | | 1.242 down | unique protein |
| 119534 | 0.000759 | 13.97757 | | 13.66003 | | 12.12751 | 3.605 up | | 2.892 up | | 1.246 down | unique protein |
| 103153 | 0.000199 | 8.50848 | | 8.8504 | | 4.97628 | 11.569 up | | 14.663 up | | 1.267 up | unique protein |
| 65810 | 0.0008 | 9.59929 | | 9.97406 | | 8.5383 | 2.086 up | | 2.705 up | | 1.296 up | unique protein |
| 111948 | 0.0152 | 5.8062 | | 6.24585 | | 4.67203 | 2.194 up | | 2.976 up | | 1.356 up | unique protein |
| 106564 | 0.00926 | 6.10478 | | 6.58235 | | 4.73875 | 2.577 up | | 3.589 up | | 1.392 up | unique protein |
| 102386 | 0.00545 | 6.70925 | | 7.20215 | | 5.08367 | 3.085 up | | 4.342 up | | 1.407 up | unique protein |
| 103172 | 0.000356 | 7.15762 | | 6.6618 | | 4.20876 | 7.721 up | | 5.475 up | | 1.410 down | unique protein |
| 104867 | 0.000867 | 8.52377 | | 9.02858 | | 6.25922 | 4.805 up | | 6.818 up | | 1.418 up | unique protein |
| 108145 | 0.00114 | 5.88214 | | 6.39047 | | 4.02103 | 3.632 up | | 5.167 up | | 1.422 up | unique protein |
| 106900 | 0.000537 | 8.28341 | | 8.80473 | | 6.92121 | 2.570 up | | 3.689 up | | 1.435 up | unique protein |
| 111566 | 0.000497 | 8.24541 | | 7.71876 | | 5.00138 | 9.474 up | | 6.576 up | | 1.440 down | unique protein |
| 121475 | 0.00106 | 13.40234 | | 13.93584 | | 11.04375 | 5.128 up | | 7.423 up | | 1.447 up | unique protein |
| 108870 | 0.000364 | 10.57277 | | 11.12225 | | 8.89265 | 3.204 up | | 4.690 up | | 1.463 up | unique protein |
| 105106 | 0.00151 | 12.95609 | | 12.34618 | | 10.38027 | 5.962 up | | 3.906 up | | 1.526 down | unique protein |
| 112400 | 0.000325 | 10.87736 | | 11.51955 | | 8.21851 | 6.315 up | | 9.856 up | | 1.560 up | unique protein |
| 108354 | 0.00327 | 7.07905 | | 7.72324 | | 5.75811 | 2.498 up | | 3.904 up | | 1.562 up | unique protein |
| 122813 | 0.0071 | 10.23621 | | 10.88079 | | 8.96114 | 2.420 up | | 3.783 up | | 1.563 up | unique protein |
| 123608 | 0.00216 | 7.21016 | | 7.90024 | | 6.18054 | 2.041 up | | 3.293 up | | 1.613 up | unique protein |
| 110008 | 0.00113 | 8.77064 | | 8.05196 | | 4.78643 | 15.825 up | | 9.616 up | | 1.645 down | unique protein |
| 111373 | 0.00276 | 8.14413 | | 8.86713 | | 7.08572 | 2.082 up | | 3.437 up | | 1.650 up | unique protein |
| 110026 | 0.000996 | 5.04016 | | 5.78136 | | 3.70215 | 2.528 up | | 4.225 up | | 1.671 up | unique protein |
| 103660 | 0.000154 | 8.49868 | | 9.25564 | | 6.90474 | 3.018 up | | 5.101 up | | 1.689 up | unique protein |
| 107973 | 0.000335 | 9.00771 | | 8.23617 | | 6.11139 | 7.445 up | | 4.361 up | | 1.707 down | unique protein |
| 123429 | 0.000669 | 10.73869 | | 11.5308 | | 9.72351 | 2.021 up | | 3.499 up | | 1.731 up | unique protein |
| 112459 | 0.000733 | 7.66711 | | 8.51834 | | 6.2285 | 2.710 up | | 4.890 up | | 1.804 up | unique protein |
| 103683 | 0.00262 | 6.18488 | | 7.03795 | | 4.50569 | 3.202 up | | 5.784 up | | 1.806 up | unique protein |
| 107218 | 0.0111 | 5.47244 | | 6.34027 | | 4.0026 | 2.769 up | | 5.054 up | | 1.824 up | unique protein |
| 111443 | 0.000868 | 8.50187 | | 7.62491 | | 5.51764 | 7.912 up | | 4.308 up | | 1.836 down | unique protein |
| 110440 | 0.000156 | 9.7206 | | 8.82338 | | 6.45252 | 9.633 up | | 5.172 up | | 1.862 down | unique protein |
| 110987 | 0.000496 | 9.73091 | | 10.63085 | | 7.5224 | 4.621 up | | 8.624 up | | 1.865 up | unique protein |
| 109670 | 0.00166 | 6.17783 | | 7.07843 | | 4.97794 | 2.297 up | | 4.288 up | | 1.866 up | unique protein |
| 111731 | 0.00159 | 6.07823 | | 6.98816 | | 4.12777 | 3.864 up | | 7.262 up | | 1.878 up | unique protein |
| 106251 | 0.00113 | 6.58991 | | 7.51019 | | 5.53695 | 2.074 up | | 3.926 up | | 1.892 up | unique protein |
| 120688 | 0.00375 | 5.25925 | | 6.18138 | | 3.72694 | 2.892 up | | 5.481 up | | 1.894 up | unique protein |
| 70972 | 0.000254 | 10.18127 | | 9.23132 | | 5.36739 | 28.127 up | | 14.559 up | | 1.931 down | unique protein |
| 110244 | 0.0021 | 7.44968 | | 8.4097 | | 6.16226 | 2.440 up | | 4.748 up | | 1.945 up | unique protein |
| 120503 | 0.000276 | 12.14878 | | 13.12752 | | 10.78674 | 2.570 up | | 5.065 up | | 1.970 up | unique protein |
| 112064 | 0.000886 | 9.96877 | | 11.00988 | | 8.02602 | 3.844 up | | 7.911 up | | 2.057 up | unique protein |
| 106253 | 0.00601 | 4.83329 | | 5.89288 | | 3.2036 | 3.094 up | | 6.449 up | | 2.084 up | unique protein |
| 107076 | 0.00103 | 5.62597 | | 6.71783 | | 4.04196 | 2.998 up | | 6.390 up | | 2.131 up | unique protein |
| 103438 | 0.000764 | 10.14492 | | 11.2468 | | 8.52326 | 3.077 up | | 6.604 up | | 2.146 up | unique protein |
| 122140 | 0.00123 | 12.06614 | | 13.17927 | | 10.21815 | 3.599 up | | 7.787 up | | 2.163 up | unique protein |
| 102850 | 0.000179 | 9.00059 | | 10.12306 | | 7.48619 | 2.856 up | | 6.219 up | | 2.177 up | unique protein |
| 120926 | 0.00079 | 6.16207 | | 7.32902 | | 4.09372 | 4.194 up | | 9.417 up | | 2.245 up | unique protein |
| 111050 | 0.00025 | 7.98341 | | 6.8097 | | 5.13315 | 7.211 up | | 3.196 up | | 2.255 down | unique protein |
| 112440 | 0.00227 | 6.07062 | | 7.257 | | 4.74801 | 2.501 up | | 5.692 up | | 2.275 up | unique protein |
| 121597 | 0.000323 | 12.78622 | | 13.97631 | | 11.20155 | 2.999 up | | 6.843 up | | 2.281 up | unique protein |
| 107972 | 0.00295 | 7.37191 | | 8.56445 | | 6.05908 | 2.484 up | | 5.677 up | | 2.285 up | unique protein |
| 111038 | 0.000923 | 10.59263 | | 11.78714 | | 7.98681 | 6.087 up | | 13.932 up | | 2.288 up | unique protein |
| 109972 | 0.000361 | 11.60938 | | 12.8115 | | 9.45484 | 4.452 up | | 10.243 up | | 2.300 up | unique protein |
| 108343 | 0.000534 | 5.6705 | | 6.89722 | | 4.66121 | 2.012 up | | 4.710 up | | 2.340 up | unique protein |
| 104750 | 0.0015 | 5.97949 | | 7.22568 | | 4.76548 | 2.319 up | | 5.502 up | | 2.372 up | unique protein |
| 107913 | 0.000766 | 7.10807 | | 5.84976 | | 3.4644 | 12.498 up | | 5.224 up | | 2.392 down | unique protein |
| 112520 | 0.000132 | 8.73494 | | 10.03818 | | 3.41615 | 39.913 up | | 98.498 up | | 2.467 up | unique protein |
| 109367 | 0.000127 | 9.9926 | | 6.46611 | | 4.97038 | 32.496 up | | 2.820 up | | 11.523 down | unique protein |
| 107494 | 0.000235 | 12.60468 | | 11.11375 | | 8.79131 | 14.058 up | | 5.001 up | | 2.810 down | unique protein |
| 123976 | 0.0000714 | 12.65138 | | 10.95096 | | 7.72401 | 30.428 up | | 9.362 up | | 3.249 down | unique protein |
| 122198 | 0.00157 | 14.40541 | | 12.60079 | | 10.32029 | 16.972 up | | 4.858 up | | 3.493 down | unique protein |
| 107914 | 0.000133 | 9.63276 | | 7.60552 | | 4.68929 | 30.770 up | | 7.548 up | | 4.076 down | unique protein |
| 110810 | 0.00019 | 11.00337 | | 12.37948 | | 9.89386 | 2.157 up | | 5.600 up | | 2.595 up | unique protein |
| 43392 | 0.0021 | 5.42276 | | 6.80553 | | 3.96084 | 2.754 up | | 7.183 up | | 2.607 up | unique protein |
| 108157 | 0.000229 | 12.22669 | | 13.67364 | | 10.29852 | 3.805 up | | 10.375 up | | 2.726 up | unique protein |
| 111527 | 0.000131 | 11.39294 | | 12.97771 | | 7.65281 | 13.362 up | | 40.082 up | | 2.999 up | unique protein |
| 110954 | 0.000564 | 9.96917 | | 11.56152 | | 8.53895 | 2.694 up | | 8.126 up | | 3.015 up | unique protein |
| 103062 | 0.000778 | 5.70176 | | 7.37288 | | 4.64445 | 2.081 up | | 6.627 up | | 3.184 up | unique protein |
| 103276 | 0.000138 | 5.22536 | | 6.93824 | | 3.58552 | 3.116 up | | 10.215 up | | 3.278 up | unique protein |
| 105349 | 0.000435 | 8.31571 | | 10.20992 | | 6.80368 | 2.852 up | | 10.601 up | | 3.717 up | unique protein |
| 112507 | 0.000181 | 6.96137 | | 9.07501 | | 5.95128 | 2.014 up | | 8.716 up | | 4.327 up | unique protein |
| 119552 | 0.0126 | 8.93737 | | 11.09887 | | 7.43724 | 2.828 up | | 12.654 up | | 4.473 up | unique protein |
| 108833 | 0.000476 | 10.66843 | | 12.84977 | | 8.43945 | 4.687 up | | 21.263 up | | 4.535 up | unique protein |
| 109267 | 0.000368 | 5.75459 | | 8.05071 | | 4.3895 | 2.575 up | | 12.651 up | | 4.911 up | unique protein |
| 102970 | 0.000766 | 5.65834 | | 7.98614 | | 4.59834 | 2.084 up | | 10.467 up | | 5.020 up | unique protein |
| 119857 | 0.000119 | 7.14736 | | 12.85032 | | 2.98956 | 17.849 up | | 929.787 up | | 52.090 up | unique protein |
| 122007 | 0.0000928 | 9.81424 | | 12.64318 | | 8.63578 | 2.263 up | | 16.082 up | | 7.105 up | unique protein |
| 120504 | 0.000243 | 9.93168 | | 12.92919 | | 7.94143 | 3.973 up | | 31.729 up | | 7.986 up | unique protein |
| 112540 | 0.000827 | 6.39813 | | 5.80583 | | 4.49213 | 3.747 up | | 2.485 up | | 1.507 down | unique protein |
| 112112 | 0.0101 | 6.15888 | | 5.43141 | | 4.13243 | 4.074 up | | 2.460 up | | 1.655 down | unique protein |
| 110167 | 0.000625 | 12.37159 | | 11.43562 | | 10.14242 | 4.688 up | | 2.450 up | | 1.913 down | unique protein |
| 106242 | 0.000407 | 9.78985 | | 9.49114 | | 8.21634 | 2.976 up | | 2.419 up | | 1.230 down | unique protein |
| 108405 | 0.000505 | 14.23989 | | 13.5776 | | 12.31265 | 3.803 up | | 2.403 up | | 1.582 down | unique protein |
| 111374 | 0.0124 | 6.06101 | | 5.66033 | | 4.41041 | 3.139 up | | 2.378 up | | 1.320 down | unique protein |
| 108807 | 0.00357 | 6.93772 | | 6.4616 | | 5.21243 | 3.306 up | | 2.377 up | | 1.390 down | unique protein |
| 105722 | 0.000079 | 10.75882 | | 9.75922 | | 8.55143 | 4.618 up | | 2.309 up | | 1.999 down | unique protein |
| 105176 | 0.0142 | 5.68585 | | 5.45579 | | 4.25035 | 2.704 up | | 2.306 up | | 1.172 down | unique protein |
| 111951 | 0.000658 | 13.55364 | | 10.49869 | | 11.6922 | 3.633 up | | 2.287 down | | 8.310 down | unique protein |
| 123732 | 0.00184 | 10.92145 | | 10.08423 | | 8.89253 | 4.080 up | | 2.284 up | | 1.786 down | unique protein |
| 104907 | 0.000315 | 9.50488 | | 9.35559 | | 8.17898 | 2.506 up | | 2.260 up | | 1.109 down | unique protein |
| 103145 | 0.00197 | 10.28537 | | 9.89721 | | 8.72586 | 2.947 up | | 2.252 up | | 1.308 down | unique protein |
| 105540 | 0.0299 | 5.25122 | | 4.96651 | | 3.84789 | 2.645 up | | 2.171 up | | 1.218 down | unique protein |
| 106181 | 0.000308 | 11.38815 | | 8.22094 | | 7.10819 | 19.426 up | | 2.162 up | | 8.983 down | unique protein |
| 103799 | 0.000345 | 10.9051 | | 10.10829 | | 9.00369 | 3.735 up | | 2.150 up | | 1.737 down | unique protein |
| 106537 | 0.00262 | 7.20238 | | 5.77619 | | 4.69696 | 5.678 up | | 2.112 up | | 2.687 down | unique protein |
| 108345 | 0.00134 | 5.41519 | | 5.11748 | | 4.08515 | 2.514 up | | 2.045 up | | 1.229 down | unique protein |
| 106089 | 0.000954 | 8.47505 | | 7.9531 | | 6.92519 | 2.927 up | | 2.039 up | | 1.435 down | unique protein |
| 121082 | 0.000401 | 14.19914 | | 13.84008 | | 12.81748 | 2.605 up | | 2.031 up | | 1.282 down | unique protein |
| 123777 | 0.00226 | 12.33279 | | 10.46473 | | 9.44526 | 7.400 up | | 2.027 up | | 3.650 down | unique protein |
| 110366 | 0.0015 | 9.34944 | | 8.9393 | | 7.97168 | 2.598 up | | 1.955 up | | 1.328 down | unique protein |
| 111222 | 0.0206 | 4.50461 | | 4.05664 | | 3.15839 | 2.542 up | | 1.863 up | | 1.364 down | unique protein |
| 110323 | 0.000769 | 8.27067 | | 7.4208 | | 6.55086 | 3.293 up | | 1.827 up | | 1.802 down | unique protein |
| 107297 | 0.00153 | 10.91915 | | 8.28203 | | 9.12482 | 3.468 up | | 1.793 down | | 6.220 down | unique protein |
| 123962 | 0.00145 | 10.20212 | | 8.24859 | | 7.41307 | 6.911 up | | 1.784 up | | 3.873 down | unique protein |
| 124338 | 0.0028 | 11.1215 | | 9.30749 | | 8.49086 | 6.192 up | | 1.761 up | | 3.516 down | unique protein |
| 107278 | 0.0074 | 5.63984 | | 4.93037 | | 4.12111 | 2.865 up | | 1.752 up | | 1.635 down | unique protein |
| 106591 | 0.0136 | 7.85122 | | 6.43672 | | 5.64009 | 4.630 up | | 1.737 up | | 2.665 down | unique protein |
| 105174 | 0.00061 | 5.65855 | | 4.96365 | | 4.18538 | 2.776 up | | 1.715 up | | 1.618 down | unique protein |
| 110214 | 0.00176 | 10.18175 | | 8.96251 | | 8.31597 | 3.644 up | | 1.565 up | | 2.328 down | unique protein |
| 112049 | 0.000629 | 6.57091 | | 4.34165 | | 3.73907 | 7.119 up | | 1.518 up | | 4.688 down | unique protein |
| 105165 | 0.00142 | 5.6975 | | 4.07198 | | 3.47467 | 4.668 up | | 1.512 up | | 3.085 down | unique protein |
| 121594 | 0.00296 | 6.92444 | | 4.77136 | | 4.18269 | 6.688 up | | 1.503 up | | 4.447 down | unique protein |
| 110767 | 0.00204 | 10.65926 | | 8.9616 | | 8.42508 | 4.704 up | | 1.450 up | | 3.243 down | unique protein |
| 103668 | 0.000226 | 9.9527 | | 7.80219 | | 7.30042 | 6.286 up | | 1.415 up | | 4.439 down | unique protein |
| 112276 | 0.00498 | 11.70048 | | 9.39363 | | 9.78624 | 3.769 up | | 1.312 down | | 4.947 down | unique protein |
| 66092 | 0.00644 | 11.37723 | | 9.59372 | | 9.9413 | 2.705 up | | 1.272 down | | 3.442 down | unique protein |
| 109244 | 0.00188 | 10.64611 | | 9.63699 | | 9.31726 | 2.512 up | | 1.248 up | | 2.012 down | unique protein |
| 112237 | 0.00364 | 5.57793 | | 4.33031 | | 4.04603 | 2.891 up | | 1.217 up | | 2.374 down | unique protein |
| 102906 | 0.00246 | 10.73994 | | 8.28841 | | 8.07127 | 6.358 up | | 1.162 up | | 5.469 down | unique protein |
| 110891 | 0.000329 | 10.03792 | | 5.90401 | | 6.00536 | 16.365 up | | 1.072 down | | 17.556 down | unique protein |
| 122614 | 0.000536 | 11.3462 | | 9.68696 | | 9.61934 | 3.310 up | | 1.047 up | | 3.158 down | unique protein |
| 110830 | 0.000276 | 9.83908 | | 8.39148 | | 8.43096 | 2.653 up | | 1.027 down | | 2.727 down | unique protein |
| 110363 | 0.000113 | 5.5803 | | 11.01079 | | 8.2532 | 6.377 down | | 6.762 up | | 43.125 up | unique protein |
| 110128 | 0.017 | 4.27699 | | 7.63276 | | 6.0817 | 3.493 down | | 2.930 up | | 10.237 up | unique protein |
| 105556 | 0.000371 | 3.59363 | | 6.25671 | | 4.68322 | 2.128 down | | 2.976 up | | 6.333 up | unique protein |
| 122629 | 0.00012 | 7.91782 | | 13.20621 | | 8.91681 | 1.998 down | | 19.554 up | | 39.080 up | unique protein |
| 107596 | 0.0203 | 5.00599 | | 6.19631 | | 4.01133 | 1.992 up | | 4.547 up | | 2.282 up | unique protein |
| 108996 | 0.000576 | 9.40955 | | 11.23084 | | 8.45414 | 1.939 up | | 6.852 up | | 3.533 up | unique protein |
| 112018 | 0.00055 | 12.57584 | | 13.98576 | | 11.64648 | 1.904 up | | 5.060 up | | 2.657 up | unique protein |
| 109945 | 0.000156 | 8.56845 | | 11.0048 | | 9.4838 | 1.886 down | | 2.869 up | | 5.412 up | unique protein |
| 108975 | 0.0347 | 6.45289 | | 7.04365 | | 5.54603 | 1.874 up | | 2.823 up | | 1.506 up | unique protein |
| 111444 | 0.00762 | 5.38515 | | 5.94073 | | 4.50525 | 1.840 up | | 2.704 up | | 1.469 up | unique protein |
| 109562 | 0.00319 | 5.55873 | | 7.28646 | | 4.68235 | 1.835 up | | 6.080 up | | 3.312 up | unique protein |
| 104175 | 0.00761 | 4.33337 | | 5.018 | | 3.49671 | 1.785 up | | 2.870 up | | 1.607 up | unique protein |
| 111439 | 0.00147 | 8.53114 | | 9.19826 | | 7.7256 | 1.747 up | | 2.775 up | | 1.587 up | unique protein |
| 106450 | 0.00106 | 12.0582 | | 12.75914 | | 11.26874 | 1.728 up | | 2.809 up | | 1.625 up | unique protein |
| 105710 | 0.000411 | 8.07674 | | 10.15675 | | 7.28893 | 1.726 up | | 7.299 up | | 4.228 up | unique protein |
| 103002 | 0.000526 | 6.37901 | | 9.26044 | | 7.15214 | 1.708 down | | 4.311 up | | 7.368 up | unique protein |
| 107194 | 0.0193 | 3.91521 | | 5.06854 | | 3.18873 | 1.654 up | | 3.680 up | | 2.224 up | unique protein |
| 103614 | 0.00726 | 6.27515 | | 7.19152 | | 5.549 | 1.654 up | | 3.122 up | | 1.887 up | unique protein |
| 108340 | 0.0000873 | 7.64494 | | 12.72032 | | 6.92648 | 1.645 up | | 55.477 up | | 33.716 up | unique protein |
| 106229 | 0.000708 | 7.85954 | | 8.62273 | | 7.14814 | 1.637 up | | 2.779 up | | 1.697 up | unique protein |
| 108852 | 0.00456 | 5.44265 | | 7.38533 | | 4.76593 | 1.598 up | | 6.144 up | | 3.844 up | unique protein |
| 112030 | 0.00987 | 3.93477 | | 4.7673 | | 3.28035 | 1.573 up | | 2.802 up | | 1.780 up | unique protein |
| 112232 | 0.00457 | 3.94901 | | 5.64286 | | 3.29662 | 1.571 up | | 5.084 up | | 3.235 up | unique protein |
| 102887 | 0.0348 | 4.5017 | | 5.34926 | | 3.85836 | 1.561 up | | 2.810 up | | 1.799 up | unique protein |
| 112274 | 0.0197 | 3.99639 | | 5.08879 | | 3.4007 | 1.511 up | | 3.222 up | | 2.132 up | unique protein |
| 107802 | 0.000442 | 8.57 | | 11.26273 | | 7.97476 | 1.510 up | | 9.767 up | | 6.465 up | unique protein |
| 104927 | 0.000311 | 7.68339 | | 11.20095 | | 7.10613 | 1.492 up | | 17.086 up | | 11.452 up | unique protein |
| 61055 | 0.000319 | 6.01476 | | 10.45141 | | 5.43907 | 1.490 up | | 32.274 up | | 21.655 up | unique protein |
| 102884 | 0.00306 | 4.65574 | | 6.85992 | | 4.08185 | 1.488 up | | 6.859 up | | 4.608 up | unique protein |
| 110665 | 0.000181 | 4.63745 | | 8.49971 | | 5.17199 | 1.448 down | | 10.040 up | | 14.543 up | unique protein |
| 109944 | 0.000328 | 4.67617 | | 7.97774 | | 4.14845 | 1.441 up | | 14.214 up | | 9.859 up | unique protein |
| 109287 | 0.000217 | 7.8245 | | 9.86959 | | 7.30817 | 1.430 up | | 5.902 up | | 4.126 up | unique protein |
| 112514 | 0.0106 | 6.12519 | | 7.23451 | | 5.60819 | 1.430 up | | 3.087 up | | 2.157 up | unique protein |
| 112630 | 0.000298 | 5.49742 | | 8.12452 | | 5.99765 | 1.414 down | | 4.367 up | | 6.177 up | unique protein |
| 110691 | 0.000812 | 5.68399 | | 6.55847 | | 5.19061 | 1.407 up | | 2.580 up | | 1.833 up | unique protein |
| 121033 | 0.00328 | 10.26487 | | 12.38534 | | 10.74919 | 1.398 down | | 3.108 up | | 4.348 up | unique protein |
| 108336 | 0.0438 | 4.97232 | | 6.66353 | | 4.4928 | 1.394 up | | 4.502 up | | 3.229 up | unique protein |
| 102787 | 0.00138 | 5.8074 | | 7.87824 | | 6.27115 | 1.379 down | | 3.046 up | | 4.201 up | unique protein |
| 105222 | 0.0141 | 3.83787 | | 5.80379 | | 4.28851 | 1.366 down | | 2.858 up | | 3.906 up | unique protein |
| 109305 | 0.0434 | 4.0301 | | 5.01014 | | 3.58653 | 1.359 up | | 2.682 up | | 1.972 up | unique protein |
| 111465 | 0.000748 | 2.96372 | | 6.69545 | | 3.40086 | 1.353 down | | 9.812 up | | 13.285 up | unique protein |
| 108367 | 0.0171 | 4.59659 | | 6.53746 | | 5.01145 | 1.333 down | | 2.879 up | | 3.839 up | unique protein |
| 108419 | 0.000308 | 5.37024 | | 7.35225 | | 4.96191 | 1.327 up | | 5.242 up | | 3.950 up | unique protein |
| 106445 | 0.00807 | 5.0072 | | 7.10703 | | 5.39931 | 1.312 down | | 3.266 up | | 4.286 up | unique protein |
| 107357 | 0.000659 | 8.38589 | | 10.73684 | | 8.00614 | 1.301 up | | 6.637 up | | 5.101 up | unique protein |
| 110367 | 0.00062 | 8.00142 | | 9.821 | | 7.62145 | 1.301 up | | 4.593 up | | 3.529 up | unique protein |
| 107843 | 0.00143 | 4.75165 | | 7.85738 | | 4.37651 | 1.296 up | | 11.164 up | | 8.608 up | unique protein |
| 107685 | 0.0141 | 5.3052 | | 6.50265 | | 4.93348 | 1.293 up | | 2.967 up | | 2.293 up | unique protein |
| 110733 | 0.00324 | 5.22054 | | 6.34238 | | 4.85575 | 1.287 up | | 2.802 up | | 2.176 up | unique protein |
| 109679 | 0.0028 | 5.93136 | | 7.15971 | | 5.5716 | 1.283 up | | 3.006 up | | 2.342 up | unique protein |
| 112366 | 0.00134 | 11.02538 | | 12.44951 | | 10.68191 | 1.268 up | | 3.404 up | | 2.683 up | unique protein |
| 109243 | 0.000732 | 4.6676 | | 8.54995 | | 5.00816 | 1.266 down | | 11.646 up | | 14.747 up | unique protein |
| 108929 | 0.0000715 | 7.96035 | | 12.117 | | 8.2654 | 1.235 down | | 14.436 up | | 17.835 up | unique protein |
| 107031 | 0.00154 | 8.05792 | | 9.15013 | | 7.75929 | 1.229 up | | 2.622 up | | 2.132 up | unique protein |
| 111593 | 0.00268 | 3.1236 | | 5.57728 | | 3.41684 | 1.225 down | | 4.470 up | | 5.478 up | unique protein |
| 108928 | 0.00126 | 3.79224 | | 6.67923 | | 3.50222 | 1.222 up | | 9.044 up | | 7.397 up | unique protein |
| 108143 | 0.000172 | 6.13427 | | 9.63064 | | 6.42353 | 1.222 down | | 9.234 up | | 11.285 up | unique protein |
| 110007 | 0.00147 | 10.97723 | | 12.72224 | | 10.70604 | 1.206 up | | 4.045 up | | 3.351 up | unique protein |
| 104511 | 0.00188 | 8.20186 | | 10.56698 | | 7.93651 | 1.201 up | | 6.192 up | | 5.151 up | unique protein |
| 108667 | 0.00135 | 7.69588 | | 9.43561 | | 7.43585 | 1.197 up | | 3.999 up | | 3.339 up | unique protein |
| 120311 | 0.00212 | 10.57093 | | 12.05372 | | 10.31619 | 1.193 up | | 3.334 up | | 2.794 up | unique protein |
| 123674 | 0.0000729 | 4.80477 | | 6.31785 | | 4.55523 | 1.188 up | | 3.393 up | | 2.854 up | unique protein |
| 120577 | 0.00102 | 11.27189 | | 13.63549 | | 11.0497 | 1.166 up | | 6.003 up | | 5.146 up | unique protein |
| 109668 | 0.000893 | 5.00098 | | 6.49082 | | 4.77824 | 1.166 up | | 3.277 up | | 2.808 up | unique protein |
| 111289 | 0.0159 | 3.04283 | | 4.7153 | | 3.25952 | 1.162 down | | 2.743 up | | 3.187 up | unique protein |
| 112518 | 0.00348 | 3.54181 | | 5.38171 | | 3.75737 | 1.161 down | | 3.083 up | | 3.579 up | unique protein |
| 112631 | 0.000266 | 5.89586 | | 8.54656 | | 5.68411 | 1.158 up | | 7.272 up | | 6.279 up | unique protein |
| 110261 | 0.000213 | 10.49261 | | 12.97107 | | 10.67021 | 1.130 down | | 4.927 up | | 5.573 up | unique protein |
| 107306 | 0.00202 | 6.28921 | | 7.8263 | | 6.46393 | 1.128 down | | 2.571 up | | 2.902 up | unique protein |
| 111645 | 0.00381 | 4.89099 | | 6.61865 | | 4.73631 | 1.113 up | | 3.686 up | | 3.311 up | unique protein |
| 108075 | 0.00114 | 5.25732 | | 6.99288 | | 5.11096 | 1.106 up | | 3.685 up | | 3.330 up | unique protein |
| 111736 | 0.000905 | 8.33503 | | 10.25933 | | 8.19387 | 1.102 up | | 4.185 up | | 3.795 up | unique protein |
| 107961 | 0.0205 | 5.57168 | | 7.04443 | | 5.44933 | 1.088 up | | 3.021 up | | 2.775 up | unique protein |
| 112215 | 0.000554 | 4.96989 | | 7.42059 | | 4.86001 | 1.079 up | | 5.899 up | | 5.466 up | unique protein |
| 106947 | 0.00861 | 3.32926 | | 4.84623 | | 3.43641 | 1.077 down | | 2.657 up | | 2.861 up | unique protein |
| 123130 | 0.000914 | 10.12606 | | 13.1957 | | 10.21494 | 1.063 down | | 7.894 up | | 8.395 up | unique protein |
| 108605 | 0.000183 | 7.11094 | | 9.2798 | | 7.03559 | 1.053 up | | 4.737 up | | 4.496 up | unique protein |
| 109030 | 0.00205 | 2.60638 | | 4.45701 | | 2.66757 | 1.043 down | | 3.456 up | | 3.606 up | unique protein |
| 105524 | 0.00102 | 4.26428 | | 5.7997 | | 4.20745 | 1.040 up | | 3.015 up | | 2.898 up | unique protein |
| 124339 | 0.00154 | 5.72833 | | 7.15533 | | 5.77987 | 1.036 down | | 2.594 up | | 2.688 up | unique protein |
| 107300 | 0.00161 | 10.97469 | | 12.84793 | | 10.93668 | 1.026 up | | 3.761 up | | 3.663 up | unique protein |
| 109345 | 0.0271 | 3.43536 | | 4.82567 | | 3.46381 | 1.019 down | | 2.570 up | | 2.621 up | unique protein |
| 107742 | 0.00286 | 5.14466 | | 6.56852 | | 5.14225 | 1.001 up | | 2.687 up | | 2.683 up | unique protein |
| 123797 | 0.000134 | 13.98118 | | 14.41792 | | 10.73853 | 9.465 up | | 12.811 up | | 1.353 up | unique protein |
| 71177 | 0.00866 | 4.51455 | | 5.26007 | | 3.61006 | 1.871 up | | 3.138 up | | 1.676 up | unique protein |
| 122975 | 0.0043 | 6.78038 | | 7.24166 | | 4.21914 | 5.902 up | | 8.125 up | | 1.376 up | unique protein with collagen triple helix repeat |
| 107960 | 0.000282 | 9.7737 | | 10.87551 | | 6.70678 | 8.379 up | | 17.985 up | | 2.146 up | unique protein with WSC domain |
| 121285 | 0.000751 | 12.10214 | | 12.27821 | | 9.87218 | 4.691 up | | 5.300 up | | 1.129 up | unique protein, 1 TM |
| 111762 | 0.000817 | 10.23582 | | 9.93205 | | 7.56733 | 6.357 up | | 5.150 up | | 1.234 down | unique protein, 1TM |
| 102960 | 0.000104 | 10.84432 | | 12.8945 | | 10.62876 | 1.161 up | | 4.808 up | | 4.141 up | unique protein, 2 TM |
| 109967 | 0.000469 | 4.41186 | | 6.00217 | | 4.41933 | 1.005 down | | 2.995 up | | 3.011 up | unique protein, 4 TM |
| 34413 | 0.00474 | 5.09098 | | 7.19492 | | 4.00839 | 2.117 up | | 9.104 up | | 4.298 up | unique protein, secreted |
| 112031 | 0.000173 | 9.05466 | | 10.66808 | | 8.35319 | 1.626 up | | 4.975 up | | 3.059 up | unique protein, secreted |
| 103579 | 0.00106 | 6.80396 | | 9.30723 | | 7.12033 | 1.245 down | | 4.553 up | | 5.669 up | unique protein, secreted, 1 TM |
| 121883 | 0.000537 | 10.61821 | | 13.23073 | | 7.8675 | 6.730 up | | 41.161 up | | 6.115 up | unique secreted protein |
| 112396 | 0.00384 | 4.40866 | | 7.8654 | | 6.40683 | 3.994 down | | 2.748 up | | 10.979 up | unique secreted protein |
| 110151 | 0.0000991 | 4.59444 | | 9.20843 | | 5.92105 | 2.508 down | | 9.763 up | | 24.487 up | unique secreted protein |
| 44362 | 0.000112 | 9.48391 | | 12.70166 | | 7.90028 | 2.997 up | | 27.884 up | | 9.303 up | unknown protein |
| 59382 | 0.000919 | 11.81288 | | 9.85192 | | 8.90577 | 7.501 up | | 1.926 up | | 3.893 down | unknown protein |
| 112578 | 0.000437 | 4.55026 | | 7.92857 | | 4.10179 | 1.364 up | | 14.189 up | | 10.398 up | unknown protein |
| 106642 | 0.00404 | 10.42327 | | 12.13312 | | 10.76917 | 1.270 down | | 2.573 up | | 3.271 up | unknown protein |
| 102837 | 0.00019 | 12.16163 | | 13.51892 | | 11.46859 | 1.616 up | | 4.142 up | | 2.562 up | unknown protein with CFEM domain |
| 120484 | 0.000837 | 11.49861 | | 12.96563 | | 11.5896 | 1.065 down | | 2.595 up | | 2.764 up | Unknown coiled-coil protein |
| 23152 | 0.000338 | 10.85361 | | 11.98809 | | 9.76773 | 2.122 up | | 4.660 up | | 2.195 up | unknown LMBR1 domain protein |
| 56117 | 0.000469 | 12.27935 | | 12.28027 | | 9.63614 | 6.247 up | | 6.251 up | | 1.000 up | unknown protein |
| 70373 | 0.000424 | 12.92444 | | 12.92518 | | 8.55517 | 20.667 up | | 20.677 up | | 1.000 up | unknown protein |
| 67286 | 0.000482 | 10.77935 | | 10.7749 | | 8.25548 | 5.751 up | | 5.733 up | | 1.003 down | unknown protein |
| 124134 | 0.00208 | 12.63024 | | 12.62371 | | 10.8037 | 3.546 up | | 3.530 up | | 1.004 down | unknown protein |
| 112371 | 0.000944 | 5.70006 | | 5.70796 | | 4.05994 | 3.116 up | | 3.134 up | | 1.005 up | unknown protein |
| 102619 | 0.00043 | 12.2544 | | 12.2441 | | 10.89738 | 2.561 up | | 2.543 up | | 1.007 down | unknown protein |
| 102411 | 0.00172 | 10.41843 | | 10.44298 | | 8.94017 | 2.786 up | | 2.833 up | | 1.017 up | unknown protein |
| 60949 | 0.000364 | 8.57582 | | 8.54793 | | 6.44441 | 4.381 up | | 4.297 up | | 1.019 down | unknown protein |
| 107111 | 0.00144 | 11.87963 | | 11.84823 | | 9.97898 | 3.733 up | | 3.653 up | | 1.022 down | unknown protein |
| 105808 | 0.00294 | 6.00461 | | 5.96648 | | 4.02537 | 3.942 up | | 3.840 up | | 1.026 down | unknown protein |
| 109311 | 0.000355 | 9.05112 | | 9.08862 | | 6.31102 | 6.681 up | | 6.857 up | | 1.026 up | unknown protein |
| 124198 | 0.00123 | 13.05159 | | 13.08931 | | 10.66729 | 5.220 up | | 5.359 up | | 1.026 up | unknown protein |
| 108577 | 0.0221 | 6.03747 | | 5.99638 | | 4.62365 | 2.664 up | | 2.589 up | | 1.028 down | unknown protein |
| 104856 | 0.00524 | 12.7083 | | 12.74998 | | 11.37214 | 2.524 up | | 2.598 up | | 1.029 up | unknown protein |
| 61298 | 0.0014 | 9.02466 | | 9.07286 | | 7.56794 | 2.744 up | | 2.838 up | | 1.033 up | unknown protein |
| 105682 | 0.00266 | 10.66498 | | 10.7136 | | 9.08619 | 2.987 up | | 3.089 up | | 1.034 up | unknown protein |
| 108546 | 0.0031 | 8.20361 | | 8.25433 | | 6.89613 | 2.475 up | | 2.563 up | | 1.035 up | unknown protein |
| 104820 | 0.0000732 | 9.47761 | | 9.42576 | | 7.89206 | 3.001 up | | 2.895 up | | 1.036 down | unknown protein |
| 121445 | 0.00195 | 9.72221 | | 9.77698 | | 8.24548 | 2.783 up | | 2.890 up | | 1.038 up | unknown protein |
| 62836 | 0.000905 | 12.48135 | | 12.41514 | | 9.87747 | 6.079 up | | 5.806 up | | 1.046 down | unknown protein |
| 61939 | 0.00119 | 11.89343 | | 11.81723 | | 9.46492 | 5.383 up | | 5.106 up | | 1.054 down | unknown protein |
| 21817 | 0.00567 | 12.80687 | | 12.89054 | | 10.90713 | 3.731 up | | 3.954 up | | 1.059 up | unknown protein |
| 23062 | 0.00255 | 13.44399 | | 13.35602 | | 11.17795 | 4.810 up | | 4.525 up | | 1.062 down | unknown protein |
| 54048 | 0.00488 | 10.23164 | | 10.13591 | | 8.51755 | 3.280 up | | 3.070 up | | 1.068 down | unknown protein |
| 107547 | 0.00106 | 9.29302 | | 9.38891 | | 7.47568 | 3.524 up | | 3.766 up | | 1.068 up | unknown protein |
| 21330 | 0.00182 | 10.92395 | | 10.82748 | | 9.45593 | 2.766 up | | 2.587 up | | 1.069 down | unknown protein |
| 123188 | 0.000888 | 8.06328 | | 8.16055 | | 6.23082 | 3.561 up | | 3.809 up | | 1.069 up | unknown protein |
| 5633 | 0.00198 | 9.85791 | | 9.95675 | | 8.40769 | 2.732 up | | 2.926 up | | 1.070 up | unknown protein |
| 68948 | 0.00501 | 11.20568 | | 11.10229 | | 9.51587 | 3.226 up | | 3.003 up | | 1.074 down | unknown protein |
| 122569 | 0.00158 | 11.57598 | | 11.4671 | | 9.92503 | 3.140 up | | 2.912 up | | 1.078 down | unknown protein |
| 105771 | 0.000646 | 11.97977 | | 12.1069 | | 9.68057 | 4.921 up | | 5.375 up | | 1.092 up | unknown protein |
| 68430 | 0.000805 | 12.81978 | | 12.94784 | | 11.03081 | 3.455 up | | 3.776 up | | 1.092 up | unknown protein |
| 109872 | 0.00129 | 12.97975 | | 13.11294 | | 11.42557 | 2.936 up | | 3.220 up | | 1.096 up | unknown protein |
| 39606 | 0.00341 | 10.26352 | | 10.12994 | | 8.78909 | 2.778 up | | 2.533 up | | 1.097 down | unknown protein |
| 76065 | 0.00442 | 11.43387 | | 11.29887 | | 9.63129 | 3.488 up | | 3.176 up | | 1.098 down | unknown protein |
| 120370 | 0.00114 | 12.72266 | | 12.85845 | | 11.20135 | 2.870 up | | 3.153 up | | 1.098 up | unknown protein |
| 78792 | 0.000367 | 10.79133 | | 10.64747 | | 8.65565 | 4.394 up | | 3.977 up | | 1.104 down | unknown protein |
| 23408 | 0.000969 | 10.93012 | | 11.07746 | | 9.02083 | 3.756 up | | 4.160 up | | 1.107 up | unknown protein |
| 123680 | 0.000747 | 10.67498 | | 10.82451 | | 8.33775 | 5.053 up | | 5.605 up | | 1.109 up | unknown protein |
| 119624 | 0.00242 | 7.13608 | | 7.28537 | | 4.92412 | 4.633 up | | 5.138 up | | 1.109 up | unknown protein |
| 76136 | 0.0024 | 12.15523 | | 11.99661 | | 10.51967 | 3.107 up | | 2.783 up | | 1.116 down | unknown protein |
| 80863 | 0.00196 | 13.9503 | | 14.10872 | | 12.64607 | 2.469 up | | 2.756 up | | 1.116 up | unknown protein |
| 49366 | 0.000361 | 12.46327 | | 12.29628 | | 10.04129 | 5.359 up | | 4.773 up | | 1.122 down | unknown protein |
| 59073 | 0.00226 | 9.72964 | | 9.56203 | | 7.30688 | 5.361 up | | 4.773 up | | 1.123 down | unknown protein |
| 70808 | 0.00129 | 9.75936 | | 9.92745 | | 7.07126 | 6.444 up | | 7.241 up | | 1.123 up | unknown protein |
| 30166 | 0.00105 | 10.41598 | | 10.5861 | | 7.47288 | 7.690 up | | 8.653 up | | 1.125 up | unknown protein |
| 111476 | 0.000628 | 10.2433 | | 10.41565 | | 8.83402 | 2.656 up | | 2.993 up | | 1.126 up | unknown protein |
| 105617 | 0.00318 | 8.3064 | | 8.47979 | | 6.69743 | 3.050 up | | 3.439 up | | 1.127 up | unknown protein |
| 60492 | 0.000358 | 11.84387 | | 12.02373 | | 10.63373 | 2.313 up | | 2.620 up | | 1.132 up | unknown protein |
| 110615 | 0.000105 | 11.2421 | | 11.05495 | | 8.41972 | 7.073 up | | 6.212 up | | 1.138 down | unknown protein |
| 79222 | 0.000164 | 9.99732 | | 10.1923 | | 8.42653 | 2.970 up | | 3.400 up | | 1.144 up | unknown protein |
| 122087 | 0.000326 | 11.43041 | | 11.23463 | | 6.8262 | 24.322 up | | 21.235 up | | 1.145 down | unknown protein |
| 122974 | 0.00884 | 11.25425 | | 11.45475 | | 9.91948 | 2.522 up | | 2.898 up | | 1.149 up | unknown protein |
| 58244 | 0.000631 | 11.79552 | | 12.01612 | | 9.944 | 3.608 up | | 4.205 up | | 1.165 up | unknown protein |
| 55454 | 0.00166 | 11.88771 | | 12.10841 | | 10.25605 | 3.098 up | | 3.610 up | | 1.165 up | unknown protein |
| 44624 | 0.00354 | 12.52885 | | 12.30073 | | 10.8254 | 3.256 up | | 2.780 up | | 1.171 down | unknown protein |
| 43893 | 0.00071 | 12.80216 | | 13.0317 | | 11.67682 | 2.181 up | | 2.557 up | | 1.172 up | unknown protein |
| 103671 | 0.000755 | 11.3298 | | 11.09762 | | 9.20977 | 4.347 up | | 3.700 up | | 1.174 down | unknown protein |
| 4677 | 0.000522 | 11.12428 | | 11.35713 | | 9.46969 | 3.148 up | | 3.699 up | | 1.175 up | unknown protein |
| 63290 | 0.00402 | 5.43318 | | 5.66686 | | 4.03084 | 2.643 up | | 3.108 up | | 1.175 up | unknown protein |
| 4659 | 0.0041 | 11.63276 | | 11.86667 | | 10.04702 | 3.001 up | | 3.529 up | | 1.176 up | unknown protein |
| 76269 | 0.0017 | 11.72398 | | 11.48831 | | 9.45938 | 4.805 up | | 4.081 up | | 1.177 down | unknown protein |
| 76713 | 0.000553 | 12.89417 | | 13.13586 | | 10.12408 | 6.821 up | | 8.065 up | | 1.182 up | unknown protein |
| 58774 | 0.00322 | 5.52544 | | 5.27935 | | 3.9131 | 3.057 up | | 2.577 up | | 1.185 down | unknown protein |
| 59768 | 0.00123 | 8.50731 | | 8.7528 | | 7.30184 | 2.306 up | | 2.733 up | | 1.185 up | unknown protein |
| 4851 | 0.000122 | 11.7562 | | 11.50824 | | 6.83647 | 30.268 up | | 25.488 up | | 1.187 down | unknown protein |
| 109317 | 0.000368 | 11.1712 | | 11.42176 | | 8.89455 | 4.845 up | | 5.764 up | | 1.189 up | unknown protein |
| 38048 | 0.000791 | 9.30544 | | 9.5564 | | 7.86708 | 2.710 up | | 3.225 up | | 1.189 up | unknown protein |
| 104102 | 0.000497 | 5.63514 | | 5.37917 | | 4.02995 | 3.042 up | | 2.547 up | | 1.194 down | unknown protein |
| 23238 | 0.000529 | 13.49693 | | 13.76082 | | 12.05849 | 2.710 up | | 3.254 up | | 1.200 up | unknown protein |
| 109444 | 0.00475 | 7.13712 | | 7.40056 | | 6.05993 | 2.109 up | | 2.532 up | | 1.200 up | unknown protein |
| 80335 | 0.00037 | 7.73618 | | 7.46973 | | 4.78059 | 7.757 up | | 6.449 up | | 1.202 down | unknown protein |
| 65695 | 0.00254 | 11.49761 | | 11.76905 | | 10.39084 | 2.153 up | | 2.599 up | | 1.207 up | unknown protein |
| 5530 | 0.00253 | 12.83681 | | 13.12617 | | 11.76444 | 2.102 up | | 2.569 up | | 1.222 up | unknown protein |
| 77743 | 0.000134 | 9.96944 | | 9.67677 | | 6.9412 | 8.158 up | | 6.660 up | | 1.224 down | unknown protein |
| 108669 | 0.000902 | 6.14809 | | 6.44057 | | 4.6883 | 2.750 up | | 3.368 up | | 1.224 up | unknown protein |
| 123687 | 0.000242 | 9.76586 | | 9.47102 | | 8.05162 | 3.281 up | | 2.674 up | | 1.226 down | unknown protein |
| 75742 | 0.00134 | 12.67482 | | 12.9697 | | 11.63769 | 2.052 up | | 2.517 up | | 1.226 up | unknown protein |
| 78626 | 0.000232 | 14.35988 | | 14.05752 | | 11.69234 | 6.353 up | | 5.152 up | | 1.233 down | unknown protein |
| 106252 | 0.00234 | 7.06014 | | 7.36629 | | 4.69396 | 5.155 up | | 6.374 up | | 1.236 up | unknown protein |
| 105356 | 0.000453 | 7.3082 | | 7.00131 | | 5.35541 | 3.871 up | | 3.129 up | | 1.237 down | unknown protein |
| 23015 | 0.000551 | 11.19717 | | 11.50966 | | 9.94587 | 2.380 up | | 2.956 up | | 1.241 up | unknown protein |
| 57383 | 0.000997 | 8.82163 | | 8.50514 | | 6.9394 | 3.686 up | | 2.960 up | | 1.245 down | unknown protein |
| 104435 | 0.00788 | 6.77706 | | 7.09564 | | 5.70347 | 2.104 up | | 2.624 up | | 1.247 up | unknown protein |
| 41518 | 0.00114 | 14.25204 | | 13.93213 | | 11.97838 | 4.835 up | | 3.873 up | | 1.248 down | unknown protein |
| 121717 | 0.000266 | 14.75829 | | 14.42865 | | 12.26844 | 5.617 up | | 4.469 up | | 1.256 down | unknown protein |
| 45598 | 0.00135 | 13.30459 | | 12.9641 | | 10.54098 | 6.790 up | | 5.363 up | | 1.266 down | unknown protein |
| 77466 | 0.000176 | 12.17872 | | 12.52325 | | 10.51702 | 3.163 up | | 4.017 up | | 1.269 up | unknown protein |
| 38640 | 0.000299 | 12.92775 | | 13.27244 | | 11.46194 | 2.762 up | | 3.507 up | | 1.269 up | unknown protein |
| 102966 | 0.000723 | 14.5081 | | 14.16025 | | 12.28375 | 4.673 up | | 3.671 up | | 1.272 down | unknown protein |
| 103446 | 0.00204 | 11.44303 | | 11.79054 | | 9.77077 | 3.187 up | | 4.055 up | | 1.272 up | unknown protein |
| 105538 | 0.00198 | 11.02759 | | 11.3789 | | 9.74252 | 2.436 up | | 3.108 up | | 1.275 up | unknown protein |
| 102521 | 0.00627 | 8.8198 | | 8.4675 | | 6.66215 | 4.461 up | | 3.495 up | | 1.276 down | unknown protein |
| 62556 | 0.000192 | 10.85882 | | 11.21146 | | 9.66137 | 2.293 up | | 2.928 up | | 1.276 up | unknown protein |
| 81591 | 0.00253 | 11.05679 | | 11.41064 | | 9.64081 | 2.668 up | | 3.410 up | | 1.277 up | unknown protein |
| 75078 | 0.0025 | 12.08632 | | 12.44725 | | 10.90153 | 2.273 up | | 2.919 up | | 1.284 up | unknown protein |
| 22257 | 0.000364 | 10.49744 | | 10.8686 | | 8.82692 | 3.183 up | | 4.117 up | | 1.293 up | unknown protein |
| 78984 | 0.000714 | 12.56201 | | 12.93528 | | 10.80047 | 3.390 up | | 4.391 up | | 1.295 up | unknown protein |
| 69933 | 0.000801 | 10.15279 | | 10.52994 | | 8.71835 | 2.702 up | | 3.510 up | | 1.298 up | unknown protein |
| 111610 | 0.00626 | 9.84598 | | 10.22878 | | 8.7424 | 2.148 up | | 2.801 up | | 1.303 up | unknown protein |
| 123019 | 0.000215 | 11.07286 | | 10.68907 | | 9.35312 | 3.293 up | | 2.524 up | | 1.304 down | unknown protein |
| 54134 | 0.00518 | 6.90402 | | 7.2878 | | 5.1804 | 3.302 up | | 4.309 up | | 1.304 up | unknown protein |
| 63464 | 0.00517 | 12.12642 | | 12.51452 | | 10.47628 | 3.138 up | | 4.107 up | | 1.308 up | unknown protein |
| 112380 | 0.0087 | 6.21215 | | 5.82153 | | 4.16293 | 4.138 up | | 3.157 up | | 1.310 down | unknown protein |
| 53722 | 0.000728 | 13.30479 | | 13.69523 | | 11.33079 | 3.928 up | | 5.149 up | | 1.310 up | unknown protein |
| 103886 | 0.000531 | 14.40497 | | 14.01099 | | 11.75922 | 6.258 up | | 4.762 up | | 1.314 down | unknown protein |
| 109296 | 0.00757 | 9.22165 | | 9.61867 | | 8.15483 | 2.094 up | | 2.758 up | | 1.316 up | unknown protein |
| 79237 | 0.00127 | 11.21815 | | 11.61595 | | 9.7433 | 2.779 up | | 3.662 up | | 1.317 up | unknown protein |
| 62285 | 0.00102 | 11.93163 | | 12.33461 | | 8.76421 | 8.984 up | | 11.879 up | | 1.322 up | unknown protein |
| 22510 | 0.000388 | 12.45698 | | 12.86674 | | 10.25211 | 4.610 up | | 6.124 up | | 1.328 up | unknown protein |
| 41362 | 0.00252 | 8.10903 | | 8.51855 | | 6.88054 | 2.343 up | | 3.112 up | | 1.328 up | unknown protein |
| 82123 | 0.00455 | 11.87997 | | 12.29196 | | 10.73248 | 2.215 up | | 2.947 up | | 1.330 up | unknown protein |
| 112285 | 0.000642 | 8.58895 | | 8.17329 | | 6.58355 | 4.015 up | | 3.009 up | | 1.333 down | unknown protein |
| 55193 | 0.00746 | 9.42695 | | 9.84503 | | 8.34568 | 2.115 up | | 2.827 up | | 1.336 up | unknown protein |
| 52438 | 0.000255 | 12.22732 | | 12.64946 | | 10.3437 | 3.690 up | | 4.944 up | | 1.339 up | unknown protein |
| 53561 | 0.000198 | 10.82578 | | 11.24791 | | 7.36831 | 10.985 up | | 14.718 up | | 1.339 up | unknown protein |
| 103852 | 0.000334 | 11.98359 | | 12.41313 | | 10.52806 | 2.742 up | | 3.693 up | | 1.346 up | unknown protein |
| 61987 | 0.00105 | 9.69264 | | 10.13276 | | 8.6859 | 2.009 up | | 2.726 up | | 1.356 up | unknown protein |
| 22694 | 0.00617 | 12.03399 | | 12.4802 | | 10.77559 | 2.392 up | | 3.259 up | | 1.362 up | unknown protein |
| 44399 | 0.000417 | 11.47628 | | 11.9227 | | 10.27626 | 2.297 up | | 3.130 up | | 1.362 up | unknown protein |
| 62789 | 0.000755 | 12.53064 | | 12.98537 | | 11.04764 | 2.795 up | | 3.831 up | | 1.370 up | unknown protein |
| 122952 | 0.000372 | 13.116 | | 13.57958 | | 11.33254 | 3.442 up | | 4.747 up | | 1.378 up | unknown protein |
| 110403 | 0.00191 | 8.85162 | | 8.38717 | | 6.92985 | 3.788 up | | 2.745 up | | 1.379 down | unknown protein |
| 112660 | 0.000659 | 10.12254 | | 10.5903 | | 8.20127 | 3.787 up | | 5.238 up | | 1.382 up | unknown protein |
| 61222 | 0.00246 | 9.08805 | | 9.55775 | | 7.82643 | 2.397 up | | 3.320 up | | 1.384 up | unknown protein |
| 120774 | 0.00035 | 9.07648 | | 9.55001 | | 7.67663 | 2.638 up | | 3.663 up | | 1.388 up | unknown protein |
| 43371 | 0.000308 | 12.31154 | | 12.79134 | | 10.89578 | 2.668 up | | 3.720 up | | 1.394 up | unknown protein |
| 121187 | 0.00112 | 11.13048 | | 11.61549 | | 9.79091 | 2.530 up | | 3.542 up | | 1.399 up | unknown protein |
| 81226 | 0.00232 | 12.16175 | | 12.65179 | | 11.14579 | 2.022 up | | 2.840 up | | 1.404 up | unknown protein |
| 75027 | 0.000273 | 13.84695 | | 13.35442 | | 10.9874 | 7.257 up | | 5.158 up | | 1.406 down | unknown protein |
| 103747 | 0.00065 | 11.68028 | | 12.17188 | | 10.48998 | 2.282 up | | 3.208 up | | 1.406 up | unknown protein |
| 58391 | 0.00139 | 10.889 | | 11.39699 | | 9.68968 | 2.296 up | | 3.265 up | | 1.422 up | unknown protein |
| 105863 | 0.00184 | 10.83607 | | 11.34667 | | 9.69821 | 2.200 up | | 3.134 up | | 1.424 up | unknown protein |
| 34353 | 0.000564 | 9.81278 | | 10.33734 | | 8.21368 | 3.029 up | | 4.357 up | | 1.438 up | unknown protein |
| 105287 | 0.000215 | 12.34994 | | 11.82361 | | 9.81035 | 5.814 up | | 4.036 up | | 1.440 down | unknown protein |
| 102414 | 0.000337 | 9.85354 | | 10.38718 | | 8.42468 | 2.692 up | | 3.897 up | | 1.447 up | unknown protein |
| 107768 | 0.000401 | 11.53574 | | 10.9908 | | 9.42392 | 4.322 up | | 2.962 up | | 1.458 down | unknown protein |
| 109756 | 0.000609 | 10.95891 | | 10.38415 | | 8.74966 | 4.624 up | | 3.104 up | | 1.489 down | unknown protein |
| 119607 | 0.00129 | 12.48482 | | 13.06144 | | 10.89643 | 3.007 up | | 4.484 up | | 1.491 up | unknown protein |
| 102504 | 0.0055 | 5.10253 | | 5.68503 | | 3.70625 | 2.632 up | | 3.941 up | | 1.497 up | unknown protein |
| 105336 | 0.0262 | 5.04407 | | 5.62724 | | 3.75355 | 2.446 up | | 3.664 up | | 1.498 up | unknown protein |
| 58525 | 0.000724 | 9.94532 | | 10.53389 | | 8.06249 | 3.687 up | | 5.545 up | | 1.503 up | unknown protein |
| 5942 | 0.00146 | 11.37343 | | 11.97224 | | 10.13426 | 2.360 up | | 3.575 up | | 1.514 up | unknown protein |
| 107002 | 0.000747 | 10.27397 | | 10.87739 | | 9.16845 | 2.151 up | | 3.269 up | | 1.519 up | unknown protein |
| 111897 | 0.000619 | 9.35627 | | 9.96028 | | 8.34372 | 2.017 up | | 3.066 up | | 1.519 up | unknown protein |
| 5651 | 0.000523 | 8.15519 | | 7.54774 | | 5.81395 | 5.067 up | | 3.325 up | | 1.523 down | unknown protein |
| 1999 | 0.00261 | 11.78436 | | 11.17031 | | 9.16068 | 6.163 up | | 4.026 up | | 1.530 down | unknown protein |
| 106569 | 0.000172 | 9.50594 | | 10.12847 | | 7.87552 | 3.096 up | | 4.766 up | | 1.539 up | unknown protein |
| 107495 | 0.000974 | 11.94538 | | 11.32121 | | 9.976 | 3.915 up | | 2.540 up | | 1.541 down | unknown protein |
| 60565 | 0.000311 | 9.98177 | | 9.34881 | | 6.35907 | 12.318 up | | 7.943 up | | 1.550 down | unknown protein |
| 106868 | 0.0016 | 8.4311 | | 9.0649 | | 6.84489 | 3.002 up | | 4.658 up | | 1.551 up | unknown protein |
| 76906 | 0.0002 | 12.66528 | | 13.30379 | | 11.63422 | 2.043 up | | 3.181 up | | 1.556 up | unknown protein |
| 82074 | 0.00127 | 11.40946 | | 12.06546 | | 10.32623 | 2.118 up | | 3.338 up | | 1.575 up | unknown protein |
| 51528 | 0.00147 | 12.00969 | | 12.66998 | | 10.7853 | 2.336 up | | 3.692 up | | 1.580 up | unknown protein |
| 2343 | 0.000235 | 8.58028 | | 9.25368 | | 7.07894 | 2.831 up | | 4.515 up | | 1.594 up | unknown protein |
| 103252 | 0.000668 | 10.8411 | | 11.51461 | | 9.55198 | 2.443 up | | 3.897 up | | 1.594 up | unknown protein |
| 120975 | 0.0000839 | 9.03417 | | 9.70803 | | 6.64124 | 5.252 up | | 8.379 up | | 1.595 up | unknown protein |
| 110595 | 0.0075 | 11.60469 | | 10.93006 | | 9.44105 | 4.480 up | | 2.806 up | | 1.596 down | unknown protein |
| 54583 | 0.000108 | 11.5301 | | 12.20462 | | 10.18393 | 2.542 up | | 4.057 up | | 1.596 up | unknown protein |
| 105917 | 0.0012 | 11.44678 | | 12.12484 | | 9.62565 | 3.533 up | | 5.653 up | | 1.599 up | unknown protein |
| 121189 | 0.00022 | 9.78349 | | 10.46754 | | 7.01284 | 6.824 up | | 10.963 up | | 1.606 up | unknown protein |
| 122089 | 0.000464 | 10.65723 | | 9.96885 | | 7.42962 | 9.367 up | | 5.812 up | | 1.611 down | unknown protein |
| 110878 | 0.000262 | 12.05752 | | 12.75359 | | 10.95428 | 2.148 up | | 3.480 up | | 1.620 up | unknown protein |
| 54226 | 0.000458 | 10.38595 | | 9.68165 | | 7.26922 | 8.674 up | | 5.323 up | | 1.629 down | unknown protein |
| 107055 | 0.000466 | 11.40786 | | 12.11799 | | 9.66028 | 3.357 up | | 5.493 up | | 1.635 up | unknown protein |
| 67030 | 0.0252 | 5.37457 | | 6.08749 | | 3.85731 | 2.862 up | | 4.691 up | | 1.639 up | unknown protein |
| 119956 | 0.000176 | 14.05947 | | 13.34553 | | 10.19014 | 14.614 up | | 8.909 up | | 1.640 down | unknown protein |
| 48080 | 0.000134 | 11.52094 | | 10.79684 | | 7.45993 | 16.691 up | | 10.104 up | | 1.651 down | unknown protein |
| 59056 | 0.000292 | 10.63399 | | 11.35843 | | 9.24691 | 2.615 up | | 4.321 up | | 1.652 up | unknown protein |
| 102989 | 0.000277 | 11.30795 | | 12.03729 | | 10.16914 | 2.201 up | | 3.650 up | | 1.657 up | unknown protein |
| 62548 | 0.00425 | 5.40733 | | 6.13764 | | 4.19504 | 2.317 up | | 3.843 up | | 1.658 up | unknown protein |
| 2399 | 0.000309 | 11.58526 | | 10.85491 | | 9.24762 | 5.054 up | | 3.046 up | | 1.659 down | unknown protein |
| 66753 | 0.00134 | 6.40956 | | 7.153 | | 4.6722 | 3.334 up | | 5.582 up | | 1.674 up | unknown protein |
| 105816 | 0.000172 | 8.89908 | | 9.64997 | | 5.80186 | 8.557 up | | 14.401 up | | 1.682 up | unknown protein |
| 120510 | 0.00449 | 11.80105 | | 12.55943 | | 10.76192 | 2.054 up | | 3.476 up | | 1.691 up | unknown protein |
| 120326 | 0.00346 | 10.76524 | | 10.00436 | | 8.56937 | 4.581 up | | 2.703 up | | 1.694 down | unknown protein |
| 41663 | 0.000745 | 11.84784 | | 12.60836 | | 9.60026 | 4.748 up | | 8.045 up | | 1.694 up | unknown protein |
| 123911 | 0.00193 | 9.40835 | | 10.16934 | | 8.12398 | 2.435 up | | 4.127 up | | 1.694 up | unknown protein |
| 61164 | 0.000338 | 8.80314 | | 9.57149 | | 6.23833 | 5.916 up | | 10.078 up | | 1.703 up | unknown protein |
| 110493 | 0.000614 | 6.59096 | | 7.36414 | | 5.00138 | 3.009 up | | 5.143 up | | 1.709 up | unknown protein |
| 63881 | 0.00118 | 8.65189 | | 9.4261 | | 7.36311 | 2.443 up | | 4.178 up | | 1.710 up | unknown protein |
| 40156 | 0.000515 | 10.35711 | | 11.13255 | | 8.25966 | 4.279 up | | 7.325 up | | 1.711 up | unknown protein |
| 112470 | 0.00105 | 10.5622 | | 11.3419 | | 9.45692 | 2.151 up | | 3.693 up | | 1.716 up | unknown protein |
| 3976 | 0.00121 | 11.91647 | | 12.6968 | | 10.19141 | 3.305 up | | 5.678 up | | 1.717 up | unknown protein |
| 80594 | 0.00024 | 11.68069 | | 12.46519 | | 9.38482 | 4.910 up | | 8.458 up | | 1.722 up | unknown protein |
| 103147 | 0.000104 | 14.35512 | | 13.56514 | | 10.83459 | 11.475 up | | 6.637 up | | 1.729 down | unknown protein |
| 108132 | 0.00354 | 10.79287 | | 12.11888 | | 10.79317 | none | | 2.506 up | | 2.507 up | unknown protein |
| 68104 | 0.000608 | 11.49515 | | 12.29107 | | 10.0943 | 2.640 up | | 4.584 up | | 1.736 up | unknown protein |
| 67866 | 0.00303 | 6.71188 | | 5.91464 | | 3.82984 | 7.371 up | | 4.242 up | | 1.737 down | unknown protein |
| 78037 | 0.000537 | 11.33549 | | 12.13571 | | 9.88781 | 2.727 up | | 4.749 up | | 1.741 up | unknown protein |
| 71284 | 0.012 | 10.68568 | | 11.48777 | | 9.65714 | 2.039 up | | 3.556 up | | 1.743 up | unknown protein |
| 41171 | 0.000878 | 10.28893 | | 9.47734 | | 7.53212 | 6.758 up | | 3.850 up | | 1.755 down | unknown protein |
| 65735 | 0.000274 | 11.81287 | | 12.62494 | | 10.08332 | 3.316 up | | 5.822 up | | 1.755 up | unknown protein |
| 112439 | 0.00027 | 14.58491 | | 13.76973 | | 11.52723 | 8.326 up | | 4.732 up | | 1.759 down | unknown protein |
| 48599 | 0.000104 | 8.98271 | | 9.79826 | | 7.40069 | 2.993 up | | 5.269 up | | 1.759 up | unknown protein |
| 107197 | 0.00427 | 8.6513 | | 9.46994 | | 6.9807 | 3.183 up | | 5.614 up | | 1.763 up | unknown protein |
| 54335 | 0.000634 | 12.53381 | | 13.3584 | | 11.04153 | 2.813 up | | 4.982 up | | 1.771 up | unknown protein |
| 80096 | 0.000264 | 12.87624 | | 13.70179 | | 11.45215 | 2.683 up | | 4.755 up | | 1.772 up | unknown protein |
| 104879 | 0.00126 | 11.25134 | | 12.08228 | | 9.82862 | 2.680 up | | 4.768 up | | 1.778 up | unknown protein |
| 102776 | 0.000835 | 12.4692 | | 13.29994 | | 11.12359 | 2.541 up | | 4.520 up | | 1.778 up | unknown protein |
| 123354 | 0.00424 | 13.33576 | | 14.18527 | | 12.24445 | 2.130 up | | 3.839 up | | 1.801 up | unknown protein |
| 111253 | 0.000779 | 11.13507 | | 11.98981 | | 10.06509 | 2.099 up | | 3.796 up | | 1.808 up | unknown protein |
| 61097 | 0.000775 | 9.29714 | | 10.16268 | | 7.14381 | 4.448 up | | 8.105 up | | 1.822 up | unknown protein |
| 67084 | 0.000104 | 8.94341 | | 9.81651 | | 5.69757 | 9.486 up | | 17.374 up | | 1.831 up | unknown protein |
| 106586 | 0.00128 | 11.46622 | | 12.34752 | | 9.97479 | 2.811 up | | 5.179 up | | 1.842 up | unknown protein |
| 106828 | 0.000252 | 10.97769 | | 11.86328 | | 9.42481 | 2.934 up | | 5.420 up | | 1.847 up | unknown protein |
| 54372 | 0.000476 | 13.1224 | | 12.23576 | | 10.87526 | 4.747 up | | 2.567 up | | 1.848 down | unknown protein |
| 75235 | 0.000507 | 10.95739 | | 11.8576 | | 9.26265 | 3.237 up | | 6.041 up | | 1.866 up | unknown protein |
| 55855 | 0.00287 | 10.7123 | | 11.63636 | | 9.68857 | 2.033 up | | 3.857 up | | 1.897 up | unknown protein |
| 112525 | 0.000189 | 10.7205 | | 11.65314 | | 9.4103 | 2.479 up | | 4.733 up | | 1.908 up | unknown protein |
| 55881 | 0.00171 | 5.64076 | | 6.57684 | | 4.56731 | 2.104 up | | 4.026 up | | 1.913 up | unknown protein |
| 102652 | 0.00158 | 11.37543 | | 12.31198 | | 10.32574 | 2.070 up | | 3.962 up | | 1.913 up | unknown protein |
| 110711 | 0.00107 | 6.30167 | | 7.24776 | | 5.0542 | 2.374 up | | 4.574 up | | 1.926 up | unknown protein |
| 70922 | 0.0185 | 5.90998 | | 6.87981 | | 4.72679 | 2.270 up | | 4.447 up | | 1.958 up | unknown protein |
| 60270 | 0.000324 | 12.04097 | | 13.01349 | | 10.13425 | 3.749 up | | 7.357 up | | 1.962 up | unknown protein |
| 120206 | 0.00215 | 11.91556 | | 12.89096 | | 10.82208 | 2.133 up | | 4.195 up | | 1.966 up | unknown protein |
| 124092 | 0.000235 | 7.92395 | | 8.91421 | | 4.11264 | 14.038 up | | 27.888 up | | 1.986 up | unknown protein |
| 107503 | 0.00262 | 7.83291 | | 8.82865 | | 6.35432 | 2.786 up | | 5.557 up | | 1.994 up | unknown protein |
| 119619 | 0.000147 | 10.33486 | | 11.33983 | | 7.69986 | 6.211 up | | 12.466 up | | 2.006 up | unknown protein |
| 5347 | 0.000134 | 11.19798 | | 10.18622 | | 8.84171 | 5.120 up | | 2.539 up | | 2.016 down | unknown protein |
| 122994 | 0.0022 | 12.28174 | | 13.29383 | | 10.55816 | 3.302 up | | 6.660 up | | 2.016 up | unknown protein |
| 111552 | 0.00189 | 7.93156 | | 8.95241 | | 6.84034 | 2.130 up | | 4.323 up | | 2.029 up | unknown protein |
| 104463 | 0.000514 | 9.30114 | | 10.3253 | | 7.72314 | 2.985 up | | 6.071 up | | 2.033 up | unknown protein |
| 110534 | 0.000723 | 9.46354 | | 10.49159 | | 8.4605 | 2.004 up | | 4.087 up | | 2.039 up | unknown protein |
| 110238 | 0.00246 | 7.55035 | | 8.58911 | | 6.10629 | 2.720 up | | 5.589 up | | 2.054 up | unknown protein |
| 80137 | 0.000428 | 11.0866 | | 12.12823 | | 10.04011 | 2.065 up | | 4.251 up | | 2.058 up | unknown protein |
| 111135 | 0.000163 | 11.96483 | | 10.92108 | | 9.45448 | 5.697 up | | 2.763 up | | 2.061 down | unknown protein |
| 105904 | 0.000704 | 8.33521 | | 9.37989 | | 6.99304 | 2.535 up | | 5.230 up | | 2.062 up | unknown protein |
| 57776 | 0.000704 | 7.45216 | | 8.50002 | | 5.81482 | 3.110 up | | 6.431 up | | 2.067 up | unknown protein |
| 55126 | 0.000822 | 10.02065 | | 8.97052 | | 7.43007 | 6.023 up | | 2.908 up | | 2.070 down | unknown protein |
| 57098 | 0.000114 | 12.83178 | | 13.88619 | | 11.52018 | 2.482 up | | 5.155 up | | 2.076 up | unknown protein |
| 63416 | 0.000307 | 10.25168 | | 11.30995 | | 8.73652 | 2.858 up | | 5.952 up | | 2.082 up | unknown protein |
| 105631 | 0.0011 | 11.87672 | | 12.94452 | | 10.36319 | 2.855 up | | 5.984 up | | 2.096 up | unknown protein |
| 22831 | 0.000516 | 10.72277 | | 11.79115 | | 9.59169 | 2.190 up | | 4.593 up | | 2.097 up | unknown protein |
| 108096 | 0.000898 | 7.01556 | | 8.10148 | | 5.97028 | 2.063 up | | 4.380 up | | 2.122 up | unknown protein |
| 103063 | 0.0043 | 6.61388 | | 7.70485 | | 5.01762 | 3.023 up | | 6.440 up | | 2.130 up | unknown protein |
| 107856 | 0.000341 | 7.31818 | | 8.4098 | | 6.00734 | 2.480 up | | 5.287 up | | 2.131 up | unknown protein |
| 104623 | 0.0018 | 11.64861 | | 12.76455 | | 10.51929 | 2.187 up | | 4.741 up | | 2.167 up | unknown protein |
| 3529 | 0.00143 | 10.61675 | | 11.74552 | | 9.52275 | 2.134 up | | 4.667 up | | 2.186 up | unknown protein |
| 56218 | 0.000355 | 11.42959 | | 12.56642 | | 9.08795 | 5.068 up | | 11.146 up | | 2.198 up | unknown protein |
| 108356 | 0.00114 | 11.88988 | | 13.03732 | | 10.68813 | 2.300 up | | 5.095 up | | 2.215 up | unknown protein |
| 103059 | 0.000473 | 6.73701 | | 7.89845 | | 5.73474 | 2.003 up | | 4.480 up | | 2.236 up | unknown protein |
| 108238 | 0.000558 | 9.69024 | | 10.85198 | | 8.57165 | 2.171 up | | 4.857 up | | 2.237 up | unknown protein |
| 65957 | 0.000141 | 9.79837 | | 10.96586 | | 8.53912 | 2.393 up | | 5.376 up | | 2.246 up | unknown protein |
| 104551 | 0.000181 | 7.14583 | | 8.32666 | | 5.3032 | 3.586 up | | 8.131 up | | 2.267 up | unknown protein |
| 5614 | 0.000183 | 10.73006 | | 11.91703 | | 7.7776 | 7.740 up | | 17.623 up | | 2.276 up | unknown protein |
| 46266 | 0.000558 | 10.73299 | | 11.92809 | | 9.16109 | 2.972 up | | 6.806 up | | 2.289 up | unknown protein |
| 53500 | 0.000133 | 10.93585 | | 9.73261 | | 8.13303 | 6.978 up | | 3.030 up | | 2.302 down | unknown protein |
| 65950 | 0.003 | 8.28931 | | 9.49503 | | 6.78421 | 2.838 up | | 6.546 up | | 2.306 up | unknown protein |
| 108382 | 0.00321 | 7.5677 | | 8.79051 | | 6.43048 | 2.199 up | | 5.133 up | | 2.334 up | unknown protein |
| 120803 | 0.000243 | 12.4554 | | 13.67952 | | 11.30431 | 2.220 up | | 5.188 up | | 2.336 up | unknown protein |
| 46127 | 0.00185 | 11.67953 | | 12.9047 | | 9.80694 | 3.661 up | | 8.560 up | | 2.337 up | unknown protein |
| 4886 | 0.000491 | 11.16506 | | 12.40033 | | 10.1149 | 2.070 up | | 4.875 up | | 2.354 up | unknown protein |
| 110159 | 0.000173 | 8.36812 | | 9.61414 | | 6.69552 | 3.187 up | | 7.561 up | | 2.371 up | unknown protein |
| 120215 | 0.00143 | 10.32896 | | 11.57665 | | 8.81834 | 2.849 up | | 6.766 up | | 2.374 up | unknown protein |
| 77593 | 0.000408 | 7.95197 | | 9.20441 | | 6.76827 | 2.271 up | | 5.411 up | | 2.382 up | unknown protein |
| 122874 | 0.000977 | 11.59503 | | 12.86983 | | 10.57103 | 2.033 up | | 4.920 up | | 2.419 up | unknown protein |
| 56593 | 0.00771 | 6.68173 | | 7.95839 | | 4.99821 | 3.212 up | | 7.782 up | | 2.422 up | unknown protein |
| 121556 | 0.000247 | 10.54948 | | 11.83413 | | 9.24956 | 2.462 up | | 5.998 up | | 2.436 up | unknown protein |
| 34252 | 0.00058 | 13.06971 | | 11.78212 | | 10.29429 | 6.846 up | | 2.804 up | | 2.441 down | unknown protein |
| 75230 | 0.000357 | 8.18359 | | 9.47616 | | 7.02629 | 2.230 up | | 5.463 up | | 2.449 up | unknown protein |
| 60768 | 0.000734 | 10.98502 | | 12.27805 | | 9.25983 | 3.306 up | | 8.101 up | | 2.450 up | unknown protein |
| 124141 | 0.000113 | 12.6802 | | 13.97983 | | 10.1023 | 5.970 up | | 14.697 up | | 2.461 up | unknown protein |
| 107279 | 0.000951 | 11.70955 | | 13.0166 | | 9.96499 | 3.350 up | | 8.291 up | | 2.474 up | unknown protein |
| 123888 | 0.00047 | 12.50621 | | 11.1983 | | 9.48537 | 8.116 up | | 3.278 up | | 2.475 down | unknown protein |
| 53401 | 0.000133 | 10.02646 | | 11.34304 | | 8.42326 | 3.038 up | | 7.567 up | | 2.490 up | unknown protein |
| 2499 | 0.00027 | 7.60932 | | 8.92943 | | 6.32447 | 2.436 up | | 6.083 up | | 2.496 up | unknown protein |
| 74508 | 0.00225 | 12.00241 | | 10.67591 | | 9.18522 | 7.047 up | | 2.810 up | | 2.507 down | unknown protein |
| 59372 | 0.000497 | 8.22433 | | 6.87359 | | 3.41577 | 28.023 up | | 10.987 up | | 2.550 down | unknown protein |
| 110831 | 0.000145 | 9.3424 | | 7.97945 | | 6.4321 | 7.517 up | | 2.922 up | | 2.572 down | unknown protein |
| 103336 | 0.000999 | 7.45768 | | 6.01099 | | 4.45181 | 8.032 up | | 2.946 up | | 2.725 down | unknown protein |
| 60560 | 0.00149 | 7.92037 | | 6.41952 | | 4.59332 | 10.035 up | | 3.546 up | | 2.830 down | unknown protein |
| 62114 | 0.00129 | 8.19964 | | 6.65475 | | 5.06093 | 8.807 up | | 3.018 up | | 2.917 down | unknown protein |
| 58639 | 0.000352 | 11.60079 | | 10.01699 | | 8.23548 | 10.305 up | | 3.437 up | | 2.997 down | unknown protein |
| 112239 | 0.0000566 | 13.13512 | | 8.74029 | | 5.16287 | 251.121 up | | 11.937 up | | 21.036 down | unknown protein |
| 53777 | 0.00113 | 11.75112 | | 10.16154 | | 8.74752 | 8.020 up | | 2.664 up | | 3.009 down | unknown protein |
| 82374 | 0.00223 | 12.42871 | | 10.65622 | | 8.9363 | 11.254 up | | 3.294 up | | 3.416 down | unknown protein |
| 106258 | 0.000493 | 9.3684 | | 7.3642 | | 5.37132 | 15.967 up | | 3.980 up | | 4.011 down | unknown protein |
| 111362 | 0.000408 | 12.82966 | | 10.57266 | | 9.10828 | 13.190 up | | 2.759 up | | 4.779 down | unknown protein |
| 60370 | 0.000267 | 11.5 | | 8.98603 | | 7.08225 | 21.373 up | | 3.741 up | | 5.711 down | unknown protein |
| 121439 | 0.000442 | 12.2735 | | 9.60312 | | 7.85002 | 21.458 up | | 3.370 up | | 6.366 down | unknown protein |
| 104304 | 0.000115 | 10.27929 | | 7.43011 | | 5.8389 | 21.711 up | | 3.013 up | | 7.205 down | unknown protein |
| 60445 | 0.000112 | 11.41754 | | 8.40294 | | 4.29631 | 139.221 up | | 17.227 up | | 8.081 down | unknown protein |
| 41425 | 0.00146 | 8.86707 | | 5.77066 | | 4.32271 | 23.334 up | | 2.728 up | | 8.552 down | unknown protein |
| 103009 | 0.000235 | 12.98562 | | 9.80426 | | 6.79689 | 72.944 up | | 8.040 up | | 9.071 down | unknown protein |
| 104295 | 0.0000845 | 7.2433 | | 10.71426 | | 4.56709 | 6.391 up | | 70.873 up | | 11.088 up | unknown protein |
| 112258 | 0.00655 | 7.21619 | | 10.81866 | | 4.28184 | 7.644 up | | 92.848 up | | 12.146 up | unknown protein |
| 103191 | 0.000274 | 5.09735 | | 9.08914 | | 4.03693 | 2.085 up | | 33.179 up | | 15.909 up | unknown protein |
| 120912 | 0.00216 | 5.94597 | | 7.27898 | | 4.7368 | 2.312 up | | 5.824 up | | 2.519 up | unknown protein |
| 60482 | 0.000183 | 12.29045 | | 13.62587 | | 9.23946 | 8.287 up | | 20.914 up | | 2.523 up | unknown protein |
| 106645 | 0.000176 | 11.23941 | | 12.61676 | | 10.20414 | 2.049 up | | 5.324 up | | 2.597 up | unknown protein |
| 40534 | 0.00101 | 10.76785 | | 12.14754 | | 9.71149 | 2.079 up | | 5.411 up | | 2.602 up | unknown protein |
| 59152 | 0.000254 | 8.61864 | | 10.01228 | | 6.27725 | 5.067 up | | 13.315 up | | 2.627 up | unknown protein |
| 81343 | 0.0000299 | 10.62917 | | 12.02398 | | 9.2441 | 2.611 up | | 6.867 up | | 2.629 up | unknown protein |
| 33827 | 0.00024 | 12.93569 | | 14.33174 | | 11.06766 | 3.650 up | | 9.606 up | | 2.631 up | unknown protein |
| 110647 | 0.000265 | 8.41923 | | 9.82637 | | 7.01743 | 2.642 up | | 7.007 up | | 2.652 up | Unknown protein |
| 3481 | 0.000935 | 10.81216 | | 12.26952 | | 9.76169 | 2.071 up | | 5.687 up | | 2.746 up | unknown protein |
| 66583 | 0.000327 | 11.85658 | | 13.33398 | | 9.38268 | 5.555 up | | 15.468 up | | 2.784 up | unknown protein |
| 121065 | 0.000309 | 10.16716 | | 11.66425 | | 8.58565 | 2.992 up | | 8.447 up | | 2.822 up | unknown protein |
| 31041 | 0.00467 | 4.71284 | | 6.22339 | | 3.53087 | 2.268 up | | 6.464 up | | 2.849 up | unknown protein |
| 69479 | 0.00128 | 8.64505 | | 10.16536 | | 7.59934 | 2.064 up | | 5.921 up | | 2.868 up | unknown protein |
| 107552 | 0.000424 | 10.23977 | | 11.76342 | | 8.76293 | 2.783 up | | 8.002 up | | 2.875 up | unknown protein |
| 120110 | 0.00118 | 10.90137 | | 12.43099 | | 9.60275 | 2.459 up | | 7.102 up | | 2.887 up | unknown protein |
| 110342 | 0.000181 | 9.43057 | | 10.96232 | | 6.67908 | 6.734 up | | 19.470 up | | 2.891 up | unknown protein |
| 106539 | 0.000804 | 7.02652 | | 8.57131 | | 5.5509 | 2.781 up | | 8.113 up | | 2.917 up | unknown protein |
| 107779 | 0.000262 | 8.5019 | | 10.05594 | | 7.37682 | 2.181 up | | 6.404 up | | 2.936 up | unknown protein |
| 106877 | 0.000786 | 10.16013 | | 11.7329 | | 7.94966 | 4.628 up | | 13.767 up | | 2.974 up | unknown protein |
| 120931 | 0.000181 | 7.19941 | | 12.00923 | | 4.32345 | 7.340 up | | 205.897 up | | 28.047 up | unknown protein |
| 72091 | 0.000236 | 12.16518 | | 13.75402 | | 9.18946 | 7.866 up | | 23.662 up | | 3.008 up | unknown protein |
| 30017 | 0.000652 | 7.98811 | | 9.58442 | | 6.95768 | 2.042 up | | 6.176 up | | 3.023 up | unknown protein |
| 108502 | 0.000145 | 10.6617 | | 12.25982 | | 9.59908 | 2.088 up | | 6.323 up | | 3.027 up | unknown protein |
| 64656 | 0.00103 | 10.85233 | | 12.48183 | | 6.70318 | 17.742 up | | 54.896 up | | 3.094 up | unknown protein |
| 44640 | 0.000462 | 12.15162 | | 13.78373 | | 10.69608 | 2.742 up | | 8.501 up | | 3.099 up | unknown protein |
| 107775 | 0.000133 | 8.70012 | | 10.34889 | | 7.31941 | 2.603 up | | 8.165 up | | 3.135 up | unknown protein |
| 119933 | 0.000959 | 11.15966 | | 12.8191 | | 10.08706 | 2.103 up | | 6.643 up | | 3.158 up | unknown protein |
| 69189 | 0.00105 | 6.49454 | | 8.158 | | 4.94535 | 2.926 up | | 9.270 up | | 3.167 up | unknown protein |
| 2703 | 0.000501 | 11.86717 | | 13.53113 | | 9.95941 | 3.752 up | | 11.890 up | | 3.168 up | unknown protein |
| 74745 | 0.0000549 | 11.84352 | | 13.50897 | | 5.77761 | 66.991 up | | 212.506 up | | 3.172 up | unknown protein |
| 67239 | 0.000248 | 9.63057 | | 11.29766 | | 8.58847 | 2.059 up | | 6.539 up | | 3.175 up | unknown protein |
| 55272 | 0.000222 | 13.40404 | | 15.09642 | | 10.26556 | 8.805 up | | 28.459 up | | 3.231 up | unknown protein |
| 77780 | 0.00546 | 8.92896 | | 10.64112 | | 7.36987 | 2.946 up | | 9.654 up | | 3.276 up | unknown protein |
| 120031 | 0.0000543 | 12.53191 | | 14.265 | | 6.56634 | 62.490 up | | 207.743 up | | 3.324 up | unknown protein |
| 67324 | 0.000102 | 10.61752 | | 12.38778 | | 9.11542 | 2.832 up | | 9.662 up | | 3.411 up | unknown protein |
| 70967 | 0.00682 | 4.81646 | | 6.59152 | | 3.23278 | 2.997 up | | 10.258 up | | 3.422 up | unknown protein |
| 123710 | 0.000354 | 9.34583 | | 11.12317 | | 7.90106 | 2.722 up | | 9.331 up | | 3.427 up | unknown protein |
| 122526 | 0.000844 | 6.71864 | | 8.49806 | | 4.95711 | 3.390 up | | 11.639 up | | 3.432 up | unknown protein |
| 37067 | 0.000177 | 10.99249 | | 12.81416 | | 9.71956 | 2.416 up | | 8.542 up | | 3.534 up | unknown protein |
| 68842 | 0.000204 | 10.64067 | | 12.47515 | | 9.58694 | 2.075 up | | 7.403 up | | 3.566 up | unknown protein |
| 32716 | 0.000389 | 8.95787 | | 10.85026 | | 7.93974 | 2.025 up | | 7.518 up | | 3.712 up | unknown protein |
| 102910 | 0.000518 | 9.2598 | | 11.24459 | | 8.21503 | 2.063 up | | 8.165 up | | 3.958 up | unknown protein |
| 79677 | 0.000423 | 10.976 | | 12.96896 | | 9.73117 | 2.369 up | | 9.433 up | | 3.980 up | unknown protein |
| 123207 | 0.000115 | 12.4303 | | 14.4294 | | 10.06025 | 5.169 up | | 20.665 up | | 3.997 up | unknown protein |
| 56840 | 0.000101 | 9.02452 | | 14.3249 | | 6.16911 | 7.237 up | | 285.190 up | | 39.406 up | unknown protein |
| 104437 | 0.000968 | 11.49203 | | 13.4941 | | 9.89338 | 3.028 up | | 12.131 up | | 4.005 up | unknown protein |
| 2837 | 0.00103 | 9.88414 | | 11.92854 | | 8.42047 | 2.758 up | | 11.377 up | | 4.125 up | unknown protein |
| 75937 | 0.000486 | 6.46849 | | 8.52975 | | 4.75487 | 3.279 up | | 13.688 up | | 4.173 up | unknown protein |
| 76075 | 0.000075 | 11.72661 | | 13.79008 | | 9.2221 | 5.674 up | | 23.719 up | | 4.179 up | unknown protein |
| 53053 | 0.000237 | 10.18502 | | 12.32119 | | 8.87375 | 2.481 up | | 10.908 up | | 4.395 up | unknown protein |
| 75998 | 0.000372 | 10.63068 | | 12.76877 | | 8.26393 | 5.157 up | | 22.703 up | | 4.401 up | unknown protein |
| 122823 | 0.000763 | 6.45332 | | 8.59483 | | 5.30134 | 2.222 up | | 9.804 up | | 4.412 up | unknown protein |
| 69963 | 0.000169 | 9.7684 | | 11.94233 | | 7.91997 | 3.601 up | | 16.249 up | | 4.512 up | unknown protein |
| 106625 | 0.000198 | 5.27246 | | 7.45788 | | 4.05346 | 2.327 up | | 10.588 up | | 4.548 up | unknown protein |
| 64274 | 0.00027 | 7.90283 | | 10.09262 | | 4.54314 | 10.265 up | | 46.833 up | | 4.562 up | unknown protein |
| 104333 | 0.000208 | 9.03594 | | 11.25375 | | 8.03353 | 2.003 up | | 9.319 up | | 4.651 up | unknown protein |
| 121702 | 0.000373 | 6.88236 | | 9.10103 | | 5.85252 | 2.041 up | | 9.503 up | | 4.654 up | unknown protein |
| 54768 | 0.0000332 | 11.24927 | | 13.52512 | | 6.0309 | 37.229 up | | 180.295 up | | 4.842 up | unknown protein |
| 108586 | 0.0000437 | 8.02743 | | 13.6377 | | 4.2302 | 13.902 up | | 679.105 up | | 48.849 up | unknown protein |
| 102999 | 0.000358 | 6.79067 | | 12.41377 | | 4.3126 | 5.571 up | | 274.596 up | | 49.286 up | unknown protein |
| 111990 | 0.000444 | 9.9146 | | 12.24109 | | 8.89153 | 2.032 up | | 10.193 up | | 5.015 up | unknown protein |
| 54790 | 0.000179 | 8.87148 | | 11.33056 | | 6.39468 | 5.566 up | | 30.608 up | | 5.498 up | unknown protein |
| 73881 | 0.000514 | 10.37816 | | 12.85169 | | 8.9923 | 2.613 up | | 14.514 up | | 5.554 up | unknown protein |
| 102468 | 0.00015 | 8.9335 | | 11.42776 | | 6.41908 | 5.713 up | | 32.193 up | | 5.634 up | unknown protein |
| 107507 | 0.000374 | 9.92341 | | 12.45955 | | 7.96418 | 3.888 up | | 22.554 up | | 5.800 up | unknown protein |
| 103455 | 0.000149 | 9.41297 | | 11.9886 | | 7.85578 | 2.942 up | | 17.542 up | | 5.961 up | unknown protein |
| 122582 | 0.000564 | 9.89756 | | 12.50034 | | 6.94662 | 7.732 up | | 46.971 up | | 6.074 up | unknown protein |
| 69857 | 0.000254 | 7.35925 | | 10.07833 | | 4.51541 | 7.179 up | | 47.272 up | | 6.584 up | unknown protein |
| 105330 | 0.000888 | 5.23447 | | 8.06098 | | 4.20307 | 2.044 up | | 14.499 up | | 7.093 up | unknown protein |
| 106623 | 0.000195 | 6.64677 | | 9.4804 | | 4.51431 | 4.384 up | | 31.256 up | | 7.128 up | unknown protein |
| 109346 | 0.000357 | 10.50326 | | 13.33888 | | 9.48749 | 2.021 up | | 14.433 up | | 7.138 up | unknown protein |
| 56211 | 0.000961 | 8.68884 | | 11.62781 | | 7.09147 | 3.025 up | | 23.204 up | | 7.668 up | unknown protein |
| 75380 | 0.000468 | 6.54032 | | 9.48041 | | 4.83553 | 3.259 up | | 25.017 up | | 7.674 up | unknown protein |
| 111778 | 0.000175 | 7.80025 | | 10.7492 | | 6.55729 | 2.366 up | | 18.276 up | | 7.721 up | unknown protein |
| 102904 | 0.000138 | 9.62261 | | 12.65518 | | 8.39518 | 2.341 up | | 19.159 up | | 8.182 up | unknown protein |
| 59801 | 0.000486 | 7.6542 | | 10.81595 | | 5.76887 | 3.694 up | | 33.061 up | | 8.949 up | unknown protein |
| 52476 | 0.00012 | 7.52749 | | 13.95861 | | 4.02309 | 11.348 up | | 979.246 up | | 86.290 up | unknown protein |
| 108591 | 0.000369 | 9.80624 | | 13.00994 | | 8.03299 | 3.418 up | | 31.492 up | | 9.213 up | unknown protein |
| 55990 | 0.000255 | 9.54735 | | 12.79527 | | 3.70609 | 57.331 up | | 544.647 up | | 9.499 up | unknown protein |
| 79726 | 0.000155 | 9.56325 | | 12.84739 | | 6.54119 | 8.123 up | | 79.132 up | | 9.741 up | unknown protein |
| 64834 | 0.000528 | 10.99709 | | 10.60826 | | 9.29215 | 3.260 up | | 2.489 up | | 1.309 down | unknown protein |
| 112267 | 0.00113 | 11.16755 | | 8.19004 | | 9.49661 | 3.184 up | | 2.473 down | | 7.876 down | unknown protein |
| 54659 | 0.000216 | 12.91242 | | 9.25301 | | 7.94933 | 31.191 up | | 2.468 up | | 12.635 down | unknown protein |
| 59665 | 0.0105 | 10.18701 | | 9.63698 | | 8.36041 | 3.547 up | | 2.422 up | | 1.464 down | unknown protein |
| 2322 | 0.000878 | 9.60623 | | 8.82452 | | 7.5534 | 4.149 up | | 2.413 up | | 1.719 down | unknown protein |
| 121653 | 0.00107 | 13.04292 | | 11.62666 | | 10.35737 | 6.433 up | | 2.410 up | | 2.668 down | unknown protein |
| 109404 | 0.00077 | 13.38913 | | 12.54702 | | 11.28193 | 4.308 up | | 2.403 up | | 1.792 down | unknown protein |
| 123697 | 0.00915 | 13.70386 | | 12.88886 | | 11.62825 | 4.215 up | | 2.395 up | | 1.759 down | Unknown protein |
| 66034 | 0.0155 | 7.75755 | | 7.28585 | | 6.02789 | 3.316 up | | 2.391 up | | 1.386 down | unknown protein |
| 47286 | 0.0023 | 13.64227 | | 13.14834 | | 11.89092 | 3.366 up | | 2.390 up | | 1.408 down | unknown protein |
| 119972 | 0.0053 | 12.65069 | | 12.32975 | | 11.08717 | 2.955 up | | 2.366 up | | 1.249 down | unknown protein |
| 111875 | 0.00961 | 11.88971 | | 11.63967 | | 10.39703 | 2.814 up | | 2.366 up | | 1.189 down | unknown protein |
| 112147 | 0.00197 | 7.12839 | | 6.69797 | | 5.48999 | 3.113 up | | 2.310 up | | 1.347 down | unknown protein |
| 112115 | 0.0105 | 4.72615 | | 4.47377 | | 3.27123 | 2.741 up | | 2.301 up | | 1.191 down | unknown protein |
| 109708 | 0.00199 | 11.58587 | | 11.10792 | | 9.90862 | 3.198 up | | 2.296 up | | 1.392 down | unknown protein |
| 51415 | 0.00118 | 13.00304 | | 12.24767 | | 11.07446 | 3.806 up | | 2.255 up | | 1.688 down | unknown protein |
| 102836 | 0.0285 | 7.03991 | | 6.82548 | | 5.65524 | 2.611 up | | 2.250 up | | 1.160 down | unknown protein |
| 81659 | 0.00128 | 12.30897 | | 11.12862 | | 9.96297 | 5.084 up | | 2.243 up | | 2.266 down | unknown protein |
| 2730 | 0.000379 | 13.27852 | | 12.06629 | | 10.90498 | 5.182 up | | 2.236 up | | 2.316 down | unknown protein |
| 4862 | 0.000247 | 10.89652 | | 10.42534 | | 9.27389 | 3.079 up | | 2.221 up | | 1.386 down | unknown protein |
| 62053 | 0.000241 | 11.97742 | | 11.58051 | | 10.44585 | 2.890 up | | 2.195 up | | 1.316 down | unknown protein |
| 108199 | 0.00301 | 10.84082 | | 10.54657 | | 9.42544 | 2.667 up | | 2.175 up | | 1.226 down | unknown protein |
| 112568 | 0.00576 | 5.74064 | | 4.87171 | | 3.75261 | 3.966 up | | 2.172 up | | 1.826 down | unknown protein |
| 79990 | 0.00775 | 11.18302 | | 10.9063 | | 9.79742 | 2.612 up | | 2.156 up | | 1.211 down | unknown protein |
| 64898 | 0.00503 | 11.55514 | | 10.73082 | | 9.62345 | 3.815 up | | 2.154 up | | 1.770 down | unknown protein |
| 56804 | 0.0143 | 5.69828 | | 5.25806 | | 4.15349 | 2.917 up | | 2.150 up | | 1.356 down | unknown protein |
| 66929 | 0.000938 | 12.9402 | | 11.881 | | 10.78456 | 4.455 up | | 2.138 up | | 2.083 down | unknown protein |
| 70894 | 0.000552 | 12.75939 | | 10.29467 | | 9.19971 | 11.791 up | | 2.136 up | | 5.520 down | unknown protein |
| 21971 | 0.00166 | 10.75689 | | 10.19267 | | 9.10432 | 3.143 up | | 2.126 up | | 1.478 down | unknown protein |
| 111059 | 0.00142 | 9.52873 | | 8.47901 | | 7.40887 | 4.346 up | | 2.099 up | | 2.070 down | unknown protein |
| 112551 | 0.000442 | 10.57172 | | 9.99978 | | 8.92956 | 3.121 up | | 2.099 up | | 1.486 down | unknown protein |
| 52701 | 0.00257 | 12.4541 | | 11.85944 | | 10.80421 | 3.138 up | | 2.078 up | | 1.510 down | unknown protein |
| 108540 | 0.00131 | 13.17387 | | 11.10697 | | 10.0523 | 8.703 up | | 2.077 up | | 4.189 down | unknown protein |
| 57002 | 0.00112 | 12.40822 | | 11.28048 | | 10.23163 | 4.520 up | | 2.068 up | | 2.185 down | unknown protein |
| 112193 | 0.000262 | 12.22694 | | 9.63064 | | 10.66577 | 2.950 up | | 2.049 down | | 6.047 down | unknown protein |
| 67133 | 0.00309 | 11.33519 | | 10.5412 | | 9.52128 | 3.515 up | | 2.027 up | | 1.733 down | unknown protein |
| 102996 | 0.00086 | 6.86425 | | 5.93289 | | 4.93349 | 3.812 up | | 1.999 up | | 1.907 down | unknown protein |
| 108158 | 0.000247 | 12.79035 | | 12.07014 | | 11.07414 | 3.285 up | | 1.994 up | | 1.647 down | unknown protein |
| 34274 | 0.00209 | 12.59463 | | 12.01064 | | 11.01452 | 2.989 up | | 1.994 up | | 1.498 down | unknown protein |
| 104302 | 0.000223 | 7.81719 | | 6.29904 | | 5.33396 | 5.591 up | | 1.952 up | | 2.864 down | unknown protein |
| 107217 | 0.00228 | 6.95849 | | 5.47724 | | 4.51529 | 5.438 up | | 1.947 up | | 2.791 down | unknown protein |
| 122860 | 0.000867 | 12.68327 | | 12.2294 | | 11.27786 | 2.648 up | | 1.933 up | | 1.369 down | unknown protein |
| 111168 | 0.00399 | 7.67471 | | 6.33945 | | 5.40504 | 4.822 up | | 1.911 up | | 2.523 down | unknown protein |
| 123460 | 0.000619 | 12.93604 | | 12.28299 | | 11.34911 | 3.004 up | | 1.910 up | | 1.572 down | unknown protein |
| 40374 | 0.00102 | 9.74994 | | 9.12024 | | 8.18662 | 2.955 up | | 1.910 up | | 1.547 down | unknown protein |
| 46764 | 0.00721 | 13.59515 | | 12.47309 | | 11.55571 | 4.110 up | | 1.888 up | | 2.176 down | unknown protein |
| 75361 | 0.000306 | 11.70557 | | 11.06337 | | 10.15159 | 2.936 up | | 1.881 up | | 1.560 down | unknown protein |
| 104215 | 0.000158 | 8.09495 | | 6.11376 | | 5.20681 | 7.403 up | | 1.875 up | | 3.948 down | unknown protein |
| 109249 | 0.000664 | 10.7273 | | 9.77663 | | 8.88931 | 3.575 up | | 1.849 up | | 1.932 down | unknown protein |
| 42848 | 0.00118 | 14.25584 | | 13.34723 | | 12.46251 | 3.466 up | | 1.846 up | | 1.877 down | unknown protein |
| 21120 | 0.00202 | 12.30919 | | 11.40321 | | 10.53984 | 3.409 up | | 1.819 up | | 1.873 down | unknown protein |
| 41942 | 0.00174 | 10.25396 | | 9.67507 | | 8.82072 | 2.700 up | | 1.807 up | | 1.493 down | unknown protein |
| 65739 | 0.00118 | 10.31917 | | 8.3554 | | 7.5058 | 7.029 up | | 1.802 up | | 3.900 down | unknown protein |
| 105223 | 0.00484 | 8.62201 | | 6.89569 | | 6.0649 | 5.885 up | | 1.778 up | | 3.308 down | unknown protein |
| 111579 | 0.0081 | 6.40707 | | 5.43802 | | 4.6229 | 3.444 up | | 1.759 up | | 1.957 down | unknown protein |
| 61383 | 0.00028 | 11.923 | | 11.37753 | | 10.57145 | 2.551 up | | 1.748 up | | 1.459 down | unknown protein |
| 70329 | 0.0178 | 5.35771 | | 4.35396 | | 3.56305 | 3.469 up | | 1.730 up | | 2.005 down | unknown protein |
| 120928 | 0.000742 | 7.96888 | | 7.37596 | | 6.5938 | 2.593 up | | 1.719 up | | 1.508 down | unknown protein |
| 109410 | 0.00113 | 10.69992 | | 9.85895 | | 9.08168 | 3.070 up | | 1.713 up | | 1.791 down | unknown protein |
| 56700 | 0.00181 | 7.40856 | | 5.33946 | | 4.57865 | 7.110 up | | 1.694 up | | 4.196 down | unknown protein |
| 40943 | 0.000446 | 11.96989 | | 10.43278 | | 9.69365 | 4.844 up | | 1.669 up | | 2.902 down | unknown protein |
| 74515 | 0.000839 | 13.53451 | | 12.83073 | | 12.11404 | 2.676 up | | 1.643 up | | 1.628 down | unknown protein |
| 45317 | 0.00404 | 12.29533 | | 9.96793 | | 10.67985 | 3.064 up | | 1.637 down | | 5.019 down | unknown protein |
| 103156 | 0.000134 | 8.27739 | | 7.54164 | | 6.8335 | 2.720 up | | 1.633 up | | 1.665 down | unknown protein |
| 71034 | 0.00569 | 6.98942 | | 6.25789 | | 5.57298 | 2.669 up | | 1.607 up | | 1.660 down | unknown protein |
| 122293 | 0.00211 | 11.06394 | | 10.31633 | | 9.66529 | 2.636 up | | 1.570 up | | 1.679 down | unknown protein |
| 107888 | 0.00148 | 11.76845 | | 10.70991 | | 10.09252 | 3.195 up | | 1.534 up | | 2.082 down | unknown protein |
| 80685 | 0.00194 | 10.18267 | | 8.69623 | | 8.10455 | 4.222 up | | 1.507 up | | 2.801 down | unknown protein |
| 48482 | 0.00862 | 12.49402 | | 11.54323 | | 10.95265 | 2.910 up | | 1.505 up | | 1.932 down | unknown protein |
| 107855 | 0.00149 | 5.74117 | | 4.41238 | | 3.85202 | 3.704 up | | 1.474 up | | 2.511 down | unknown protein |
| 56026 | 0.00138 | 10.91919 | | 9.85236 | | 9.29197 | 3.089 up | | 1.474 up | | 2.094 down | unknown protein |
| 109321 | 0.00498 | 6.26849 | | 4.52909 | | 3.98524 | 4.867 up | | 1.457 up | | 3.338 down | unknown protein |
| 59491 | 0.000486 | 8.78579 | | 7.2344 | | 6.69332 | 4.264 up | | 1.455 up | | 2.930 down | unknown protein |
| 112390 | 0.00177 | 14.25944 | | 13.18515 | | 12.65691 | 3.036 up | | 1.442 up | | 2.105 down | unknown protein |
| 120767 | 0.00255 | 12.90776 | | 10.91932 | | 11.43381 | 2.777 up | | 1.428 down | | 3.968 down | unknown protein |
| 105718 | 0.00159 | 11.10073 | | 6.36574 | | 6.86295 | 18.866 up | | 1.411 down | | 26.630 down | unknown protein |
| 59002 | 0.00153 | 7.02323 | | 6.14183 | | 5.64561 | 2.598 up | | 1.410 up | | 1.842 down | unknown protein |
| 4494 | 0.0135 | 12.22435 | | 11.26139 | | 10.8054 | 2.673 up | | 1.371 up | | 1.949 down | unknown protein |
| 105051 | 0.000703 | 14.28428 | | 12.51085 | | 12.93322 | 2.550 up | | 1.340 down | | 3.418 down | unknown protein |
| 51558 | 0.00362 | 12.02165 | | 10.79611 | | 10.38789 | 3.103 up | | 1.327 up | | 2.338 down | unknown protein |
| 111194 | 0.00199 | 14.45879 | | 11.40988 | | 11.79962 | 6.316 up | | 1.310 down | | 8.275 down | unknown protein |
| 105765 | 0.000336 | 11.39363 | | 9.18251 | | 9.55585 | 3.574 up | | 1.295 down | | 4.630 down | unknown protein |
| 104137 | 0.00257 | 10.04927 | | 8.69426 | | 8.35205 | 3.242 up | | 1.267 up | | 2.557 down | unknown protein |
| 33632 | 0.00346 | 13.90713 | | 12.14133 | | 12.46144 | 2.723 up | | 1.248 down | | 3.400 down | unknown protein |
| 102830 | 0.00197 | 12.69377 | | 10.591 | | 10.29684 | 5.266 up | | 1.226 up | | 4.295 down | unknown protein |
| 62484 | 0.00168 | 10.97877 | | 9.04429 | | 9.33908 | 3.115 up | | 1.226 down | | 3.822 down | unknown protein |
| 103740 | 0.0117 | 9.92987 | | 8.86486 | | 8.58357 | 2.542 up | | 1.215 up | | 2.092 down | unknown protein |
| 57643 | 0.000226 | 10.94808 | | 6.09555 | | 5.81797 | 35.020 up | | 1.212 up | | 28.890 down | unknown protein |
| 106154 | 0.00495 | 6.09454 | | 4.42207 | | 4.68254 | 2.661 up | | 1.197 down | | 3.187 down | unknown protein |
| 109117 | 0.00156 | 12.91971 | | 11.30893 | | 11.56521 | 2.557 up | | 1.194 down | | 3.054 down | unknown protein |
| 107853 | 0.00149 | 10.62251 | | 7.98979 | | 8.22947 | 5.252 up | | 1.180 down | | 6.201 down | unknown protein |
| 66786 | 0.00135 | 12.32281 | | 9.97522 | | 10.18394 | 4.404 up | | 1.155 down | | 5.089 down | unknown protein |
| 110768 | 0.000592 | 13.16172 | | 11.42067 | | 11.2366 | 3.797 up | | 1.136 up | | 3.342 down | unknown protein |
| 59759 | 0.000634 | 6.01195 | | 4.40523 | | 4.22383 | 3.453 up | | 1.133 up | | 3.045 down | unknown protein |
| 124177 | 0.0021 | 13.2304 | | 11.67136 | | 11.51766 | 3.277 up | | 1.112 up | | 2.946 down | unknown protein |
| 124293 | 0.00244 | 14.92732 | | 13.08938 | | 12.9457 | 3.949 up | | 1.104 up | | 3.574 down | unknown protein |
| 75394 | 0.000497 | 12.26592 | | 10.82623 | | 10.6852 | 2.991 up | | 1.102 up | | 2.712 down | unknown protein |
| 106356 | 0.00469 | 12.25514 | | 10.9888 | | 10.8499 | 2.648 up | | 1.101 up | | 2.405 down | unknown protein |
| 104425 | 0.00135 | 8.59089 | | 6.13625 | | 6.05624 | 5.794 up | | 1.057 up | | 5.481 down | unknown protein |
| 107502 | 0.000817 | 11.83661 | | 10.31116 | | 10.38369 | 2.737 up | | 1.051 down | | 2.878 down | unknown protein |
| 109883 | 0.000247 | 12.04714 | | 10.57343 | | 10.64613 | 2.640 up | | 1.051 down | | 2.777 down | unknown protein |
| 104272 | 0.00227 | 13.45287 | | 10.69179 | | 10.74744 | 6.522 up | | 1.039 down | | 6.779 down | unknown protein |
| 104741 | 0.0115 | 11.06038 | | 9.43353 | | 9.4871 | 2.975 up | | 1.037 down | | 3.088 down | unknown protein |
| 123686 | 0.000761 | 14.90794 | | 13.19459 | | 13.1524 | 3.376 up | | 1.029 up | | 3.279 down | unknown protein |
| 65718 | 0.00179 | 14.37996 | | 12.70501 | | 12.71408 | 3.173 up | | 1.006 down | | 3.193 down | unknown protein |
| 102892 | 0.000343 | 13.3925 | | 11.94664 | | 11.94512 | 2.727 up | | 1.001 up | | 2.724 down | unknown protein |
| 59940 | 0.0022 | 12.0545 | | 10.71785 | | 10.71608 | 2.528 up | | 1.001 up | | 2.525 down | unknown protein |
| 103576 | 0.000301 | 6.50542 | | 10.25716 | | 6.5048 | none | | 13.476 up | | 13.470 up | unknown protein |
| 2033 | 0.000944 | 4.81145 | | 8.58555 | | 7.17477 | 5.145 down | | 2.658 up | | 13.680 up | unknown protein |
| 69303 | 0.000275 | 7.39694 | | 11.94734 | | 9.14341 | 3.355 down | | 6.983 up | | 23.431 up | unknown protein |
| 54052 | 0.00464 | 7.02083 | | 10.80124 | | 8.45912 | 2.709 down | | 5.070 up | | 13.740 up | unknown protein |
| 108144 | 0.000602 | 4.06779 | | 7.3743 | | 5.34193 | 2.418 down | | 4.090 up | | 9.893 up | unknown protein |
| 119881 | 0.00156 | 10.80015 | | 13.45054 | | 12.03786 | 2.358 down | | 2.662 up | | 6.278 up | unknown protein |
| 67008 | 0.00103 | 9.31516 | | 11.93125 | | 10.54462 | 2.344 down | | 2.614 up | | 6.130 up | unknown protein |
| 3063 | 0.000222 | 9.8181 | | 12.79735 | | 11.04366 | 2.338 down | | 3.372 up | | 7.885 up | unknown protein |
| 70218 | 0.000392 | 8.81426 | | 11.96438 | | 9.9691 | 2.226 down | | 3.986 up | | 8.877 up | unknown protein |
| 57253 | 0.00341 | 8.42491 | | 11.10661 | | 9.57718 | 2.222 down | | 2.886 up | | 6.416 up | unknown protein |
| 43101 | 0.000954 | 4.43231 | | 6.9468 | | 5.53962 | 2.154 down | | 2.652 up | | 5.713 up | unknown protein |
| 111103 | 0.000653 | 8.89114 | | 12.08256 | | 9.9748 | 2.119 down | | 4.310 up | | 9.135 up | unknown protein |
| 123476 | 0.0000845 | 8.73683 | | 12.50963 | | 9.8147 | 2.110 down | | 6.475 up | | 13.668 up | unknown protein |
| 71123 | 0.000172 | 7.27129 | | 11.24921 | | 8.32707 | 2.078 down | | 7.579 up | | 15.757 up | unknown protein |
| 120819 | 0.000611 | 8.34433 | | 11.07186 | | 9.38144 | 2.052 down | | 3.227 up | | 6.623 up | unknown protein |
| 111450 | 0.0016 | 7.3054 | | 10.0944 | | 8.3298 | 2.034 down | | 3.397 up | | 6.911 up | unknown protein |
| 103112 | 0.00042 | 6.88205 | | 9.73218 | | 7.89808 | 2.022 down | | 3.565 up | | 7.210 up | unknown protein |
| 55374 | 0.000155 | 4.87097 | | 10.45945 | | 8.46864 | 12.106 down | | 3.974 up | | 48.117 up | unknown protein |
| 108191 | 0.00356 | 5.65925 | | 6.47727 | | 4.66275 | 1.995 up | | 3.517 up | | 1.762 up | unknown protein |
| 104556 | 0.0004 | 5.79074 | | 8.12478 | | 4.79391 | 1.995 up | | 10.062 up | | 5.042 up | unknown protein |
| 69181 | 0.000275 | 2.95474 | | 7.52067 | | 3.95084 | 1.994 down | | 11.874 up | | 23.685 up | unknown protein |
| 112040 | 0.00112 | 7.12085 | | 9.06542 | | 6.12956 | 1.987 up | | 7.652 up | | 3.849 up | unknown protein |
| 121499 | 0.000456 | 9.05702 | | 9.6313 | | 8.06585 | 1.987 up | | 2.959 up | | 1.488 up | unknown protein |
| 65965 | 0.000669 | 12.34963 | | 13.78685 | | 11.3608 | 1.984 up | | 5.374 up | | 2.707 up | unknown protein |
| 102379 | 0.000163 | 7.10873 | | 8.57851 | | 6.12223 | 1.981 up | | 5.488 up | | 2.769 up | unknown protein |
| 67109 | 0.000351 | 6.13067 | | 10.4137 | | 7.11752 | 1.981 down | | 9.823 up | | 19.467 up | unknown protein |
| 120826 | 0.00012 | 12.0208 | | 13.05641 | | 11.03472 | 1.980 up | | 4.060 up | | 2.049 up | unknown protein |
| 62300 | 0.000133 | 7.21428 | | 9.31742 | | 6.22941 | 1.979 up | | 8.503 up | | 4.296 up | unknown protein |
| 57558 | 0.000197 | 9.30052 | | 11.84309 | | 8.31844 | 1.975 up | | 11.508 up | | 5.826 up | unknown protein |
| 102668 | 0.000866 | 9.23258 | | 12.00518 | | 10.21495 | 1.975 down | | 3.458 up | | 6.833 up | unknown protein |
| 108348 | 0.00352 | 5.19245 | | 6.51148 | | 4.21204 | 1.973 up | | 4.922 up | | 2.494 up | unknown protein |
| 107012 | 0.00301 | 10.13143 | | 10.89892 | | 9.15078 | 1.973 up | | 3.359 up | | 1.702 up | unknown protein |
| 107253 | 0.00106 | 6.69124 | | 7.13188 | | 5.71131 | 1.972 up | | 2.676 up | | 1.357 up | unknown protein |
| 104276 | 0.00101 | 9.56864 | | 10.21458 | | 8.58946 | 1.971 up | | 3.084 up | | 1.564 up | unknown protein |
| 108613 | 0.000336 | 9.95028 | | 10.86472 | | 8.97179 | 1.970 up | | 3.713 up | | 1.884 up | unknown protein |
| 109721 | 0.00228 | 7.51979 | | 9.15842 | | 6.54183 | 1.969 up | | 6.133 up | | 3.113 up | unknown protein |
| 122571 | 0.00167 | 10.62457 | | 11.3923 | | 9.64884 | 1.966 up | | 3.348 up | | 1.702 up | unknown protein |
| 54819 | 0.00178 | 5.33948 | | 8.76271 | | 6.31205 | 1.962 down | | 5.466 up | | 10.727 up | unknown protein |
| 120000 | 0.00149 | 10.24863 | | 11.22873 | | 9.27924 | 1.958 up | | 3.862 up | | 1.972 up | unknown protein |
| 63435 | 0.000341 | 5.57682 | | 8.06581 | | 6.54634 | 1.958 down | | 2.866 up | | 5.613 up | unknown protein |
| 108014 | 0.000453 | 6.91833 | | 8.67673 | | 5.94995 | 1.956 up | | 6.619 up | | 3.383 up | unknown protein |
| 32849 | 0.000452 | 11.61 | | 12.85288 | | 10.64418 | 1.953 up | | 4.622 up | | 2.366 up | unknown protein |
| 65949 | 0.000219 | 8.32376 | | 11.5836 | | 9.28551 | 1.947 down | | 4.918 up | | 9.578 up | unknown protein |
| 122931 | 0.000964 | 11.72424 | | 12.79047 | | 10.76354 | 1.946 up | | 4.075 up | | 2.093 up | unknown protein |
| 107460 | 0.000146 | 8.97015 | | 11.81383 | | 8.00928 | 1.946 up | | 13.972 up | | 7.178 up | unknown protein |
| 119947 | 0.00048 | 10.65933 | | 12.24473 | | 9.69939 | 1.945 up | | 5.837 up | | 3.000 up | unknown protein |
| 103050 | 0.0114 | 5.95722 | | 6.89568 | | 4.99735 | 1.945 up | | 3.727 up | | 1.916 up | unknown protein |
| 106051 | 0.00263 | 11.84356 | | 12.96354 | | 10.88674 | 1.941 up | | 4.218 up | | 2.173 up | unknown protein |
| 42513 | 0.00042 | 8.04674 | | 10.98789 | | 7.09212 | 1.938 up | | 14.884 up | | 7.680 up | unknown protein |
| 103411 | 0.00583 | 5.48855 | | 7.455 | | 4.53728 | 1.933 up | | 7.556 up | | 3.908 up | unknown protein |
| 119844 | 0.00134 | 7.04465 | | 9.16776 | | 6.09995 | 1.924 up | | 8.384 up | | 4.356 up | unknown protein |
| 103366 | 0.00175 | 7.76434 | | 8.86538 | | 6.82693 | 1.915 up | | 4.108 up | | 2.145 up | unknown protein |
| 76949 | 0.00174 | 13.28365 | | 13.94857 | | 12.34725 | 1.913 up | | 3.034 up | | 1.585 up | unknown protein |
| 51868 | 0.000115 | 9.97001 | | 10.54757 | | 9.03591 | 1.910 up | | 2.851 up | | 1.492 up | unknown protein |
| 65286 | 0.00499 | 9.49417 | | 10.23183 | | 8.56192 | 1.908 up | | 3.181 up | | 1.667 up | unknown protein |
| 58746 | 0.017 | 11.67405 | | 12.15923 | | 10.74264 | 1.907 up | | 2.669 up | | 1.399 up | unknown protein |
| 64757 | 0.00746 | 10.59715 | | 11.20153 | | 9.6667 | 1.905 up | | 2.897 up | | 1.520 up | unknown protein |
| 109330 | 0.00298 | 11.95467 | | 12.88534 | | 11.02523 | 1.904 up | | 3.630 up | | 1.906 up | unknown protein |
| 123265 | 0.00227 | 10.91921 | | 12.17354 | | 9.99065 | 1.903 up | | 4.540 up | | 2.385 up | unknown protein |
| 62633 | 0.00018 | 6.58263 | | 9.92542 | | 5.65641 | 1.900 up | | 19.279 up | | 10.145 up | unknown protein |
| 43199 | 0.0024 | 4.02517 | | 5.35535 | | 3.10744 | 1.889 up | | 4.749 up | | 2.514 up | unknown protein |
| 44684 | 0.00032 | 9.50469 | | 12.17214 | | 10.42134 | 1.887 down | | 3.365 up | | 6.353 up | unknown protein |
| 54391 | 0.00113 | 12.18201 | | 13.10409 | | 11.2714 | 1.879 up | | 3.562 up | | 1.894 up | unknown protein |
| 80871 | 0.0021 | 11.71443 | | 12.80779 | | 10.80742 | 1.875 up | | 4.001 up | | 2.133 up | unknown protein |
| 102489 | 0.0214 | 4.77321 | | 5.83636 | | 3.86591 | 1.875 up | | 3.918 up | | 2.089 up | unknown protein |
| 120609 | 0.000684 | 9.0997 | | 9.94842 | | 8.20276 | 1.862 up | | 3.353 up | | 1.800 up | unknown protein |
| 66788 | 0.000144 | 9.09402 | | 12.86572 | | 8.19776 | 1.861 up | | 25.421 up | | 13.658 up | unknown protein |
| 74933 | 0.00231 | 10.8554 | | 12.4181 | | 9.95964 | 1.860 up | | 5.496 up | | 2.954 up | unknown protein |
| 106818 | 0.00686 | 12.88729 | | 14.01704 | | 11.9918 | 1.860 up | | 4.070 up | | 2.188 up | unknown protein |
| 106219 | 0.000602 | 7.71228 | | 10.50022 | | 8.60693 | 1.859 down | | 3.714 up | | 6.906 up | unknown protein |
| 5072 | 0.000388 | 8.78633 | | 11.15316 | | 9.68012 | 1.858 down | | 2.776 up | | 5.158 up | unknown protein |
| 74129 | 0.00649 | 11.51923 | | 12.11741 | | 10.62564 | 1.857 up | | 2.812 up | | 1.513 up | unknown protein |
| 108559 | 0.00115 | 13.24206 | | 13.9616 | | 12.35656 | 1.847 up | | 3.042 up | | 1.646 up | unknown protein |
| 5844 | 0.000869 | 10.20345 | | 12.35125 | | 9.31895 | 1.846 up | | 8.181 up | | 4.431 up | unknown protein |
| 54427 | 0.0053 | 10.13281 | | 11.97912 | | 9.25404 | 1.838 up | | 6.611 up | | 3.595 up | unknown protein |
| 107360 | 0.000145 | 9.60691 | | 11.2995 | | 8.73147 | 1.834 up | | 5.929 up | | 3.232 up | unknown protein |
| 76820 | 0.00018 | 10.68431 | | 13.25921 | | 9.81351 | 1.828 up | | 10.895 up | | 5.958 up | unknown protein |
| 106035 | 0.000213 | 10.03079 | | 11.03577 | | 9.16297 | 1.824 up | | 3.662 up | | 2.006 up | unknown protein |
| 102441 | 0.00122 | 6.34835 | | 7.24376 | | 5.48228 | 1.822 up | | 3.390 up | | 1.860 up | unknown protein |
| 110315 | 0.00409 | 10.82315 | | 11.93109 | | 9.96215 | 1.816 up | | 3.914 up | | 2.155 up | unknown protein |
| 109337 | 0.00102 | 10.39545 | | 11.04702 | | 9.5384 | 1.811 up | | 2.845 up | | 1.570 up | unknown protein |
| 69384 | 0.000316 | 11.42372 | | 12.62697 | | 10.56865 | 1.808 up | | 4.164 up | | 2.302 up | unknown protein |
| 119792 | 0.00549 | 9.6889 | | 11.93946 | | 10.54313 | 1.807 down | | 2.632 up | | 4.758 up | unknown protein |
| 55041 | 0.000436 | 10.31991 | | 12.16832 | | 9.46896 | 1.803 up | | 6.495 up | | 3.601 up | unknown protein |
| 107346 | 0.000394 | 6.95859 | | 8.24533 | | 6.10762 | 1.803 up | | 4.400 up | | 2.439 up | unknown protein |
| 22590 | 0.000955 | 7.63603 | | 8.76392 | | 6.79337 | 1.793 up | | 3.919 up | | 2.185 up | unknown protein |
| 122134 | 0.000428 | 11.51242 | | 12.19461 | | 10.66964 | 1.793 up | | 2.877 up | | 1.604 up | unknown protein |
| 108263 | 0.000307 | 11.90717 | | 12.91146 | | 11.0657 | 1.791 up | | 3.594 up | | 2.005 up | unknown protein |
| 121146 | 0.000463 | 10.71082 | | 11.90497 | | 9.87008 | 1.790 up | | 4.097 up | | 2.288 up | unknown protein |
| 36159 | 0.000554 | 10.52788 | | 11.6616 | | 9.68843 | 1.789 up | | 3.926 up | | 2.194 up | unknown protein |
| 22573 | 0.00127 | 11.40813 | | 12.02636 | | 10.57079 | 1.786 up | | 2.742 up | | 1.534 up | unknown protein |
| 105650 | 0.00201 | 10.53371 | | 11.80561 | | 9.69969 | 1.782 up | | 4.304 up | | 2.414 up | unknown protein |
| 56140 | 0.000125 | 12.6705 | | 13.62405 | | 11.83781 | 1.781 up | | 3.449 up | | 1.936 up | unknown protein |
| 31227 | 0.000602 | 11.48151 | | 13.21956 | | 10.65093 | 1.778 up | | 5.932 up | | 3.335 up | unknown protein |
| 110197 | 0.00439 | 4.78724 | | 5.65069 | | 3.95825 | 1.776 up | | 3.232 up | | 1.819 up | unknown protein |
| 60028 | 0.000298 | 3.54075 | | 8.58851 | | 4.36998 | 1.776 down | | 18.616 up | | 33.076 up | unknown protein |
| 30075 | 0.00053 | 9.93078 | | 11.68169 | | 9.10226 | 1.775 up | | 5.977 up | | 3.365 up | unknown protein |
| 122455 | 0.00131 | 7.5162 | | 10.20096 | | 8.33732 | 1.766 down | | 3.639 up | | 6.429 up | unknown protein |
| 57574 | 0.000421 | 11.2521 | | 12.71717 | | 10.43382 | 1.763 up | | 4.868 up | | 2.760 up | unknown protein |
| 3508 | 0.00122 | 12.94578 | | 13.8715 | | 12.13014 | 1.760 up | | 3.343 up | | 1.899 up | unknown protein |
| 107464 | 0.000115 | 7.23247 | | 10.04516 | | 8.04783 | 1.759 down | | 3.992 up | | 7.025 up | unknown protein |
| 111957 | 0.000107 | 6.43557 | | 7.90121 | | 5.62107 | 1.758 up | | 4.857 up | | 2.761 up | unknown protein |
| 121014 | 0.00477 | 10.75441 | | 11.6102 | | 9.94646 | 1.750 up | | 3.168 up | | 1.809 up | unknown protein |
| 67751 | 0.00104 | 8.56714 | | 10.26815 | | 7.76497 | 1.743 up | | 5.669 up | | 3.251 up | unknown protein |
| 120193 | 0.00231 | 10.90903 | | 11.94114 | | 10.11009 | 1.739 up | | 3.557 up | | 2.045 up | unknown protein |
| 65223 | 0.00518 | 8.89421 | | 10.07822 | | 8.09878 | 1.735 up | | 3.943 up | | 2.272 up | unknown protein |
| 56897 | 0.000446 | 7.53255 | | 8.17103 | | 6.73698 | 1.735 up | | 2.702 up | | 1.556 up | unknown protein |
| 108557 | 0.00133 | 8.78827 | | 11.04439 | | 7.99347 | 1.734 up | | 8.287 up | | 4.777 up | unknown protein |
| 5924 | 0.000476 | 8.61634 | | 10.10541 | | 7.82219 | 1.734 up | | 4.867 up | | 2.807 up | unknown protein |
| 38781 | 0.0024 | 8.90193 | | 10.82811 | | 8.10783 | 1.733 up | | 6.589 up | | 3.800 up | unknown protein |
| 104197 | 0.00135 | 4.22513 | | 7.45222 | | 3.43103 | 1.733 up | | 16.236 up | | 9.363 up | unknown protein |
| 21193 | 0.0109 | 10.8563 | | 11.45375 | | 10.06697 | 1.728 up | | 2.614 up | | 1.513 up | unknown protein |
| 121660 | 0.000211 | 12.10043 | | 13.26931 | | 11.3124 | 1.726 up | | 3.882 up | | 2.248 up | unknown protein |
| 5319 | 0.00313 | 10.22722 | | 11.99372 | | 9.4409 | 1.724 up | | 5.867 up | | 3.402 up | unknown protein |
| 61517 | 0.00253 | 11.68743 | | 12.92019 | | 10.90279 | 1.722 up | | 4.048 up | | 2.350 up | unknown protein |
| 67472 | 0.000173 | 9.14117 | | 13.41306 | | 8.35752 | 1.721 up | | 33.256 up | | 19.318 up | unknown protein |
| 45689 | 0.000516 | 11.12287 | | 12.02161 | | 10.33899 | 1.721 up | | 3.210 up | | 1.864 up | unknown protein |
| 68028 | 0.000183 | 8.51745 | | 10.34043 | | 7.74611 | 1.706 up | | 6.039 up | | 3.538 up | unknown protein |
| 22879 | 0.00269 | 11.02424 | | 11.82526 | | 10.25391 | 1.705 up | | 2.971 up | | 1.742 up | unknown protein |
| 65971 | 0.00561 | 5.2724 | | 6.09148 | | 4.50653 | 1.700 up | | 2.999 up | | 1.764 up | unknown protein |
| 26598 | 0.000686 | 8.86816 | | 11.53606 | | 9.63364 | 1.699 down | | 3.738 up | | 6.355 up | unknown protein |
| 81423 | 0.00329 | 11.58965 | | 13.05516 | | 10.82941 | 1.693 up | | 4.677 up | | 2.761 up | unknown protein |
| 106037 | 0.00675 | 11.90959 | | 12.95278 | | 11.1566 | 1.685 up | | 3.473 up | | 2.060 up | unknown protein |
| 56512 | 0.000308 | 11.12408 | | 12.13255 | | 10.37213 | 1.684 up | | 3.387 up | | 2.011 up | unknown protein |
| 42858 | 0.000235 | 7.28552 | | 11.25943 | | 6.5353 | 1.682 up | | 26.430 up | | 15.713 up | unknown protein |
| 110882 | 0.00458 | 11.36292 | | 12.09809 | | 10.61433 | 1.680 up | | 2.796 up | | 1.664 up | unknown protein |
| 78706 | 0.000209 | 10.69867 | | 12.91745 | | 9.95273 | 1.677 up | | 7.806 up | | 4.654 up | unknown protein |
| 105346 | 0.000244 | 9.43888 | | 10.85929 | | 8.69312 | 1.676 up | | 4.488 up | | 2.676 up | unknown protein |
| 65402 | 0.00295 | 9.22556 | | 11.44794 | | 9.96829 | 1.673 down | | 2.788 up | | 4.666 up | unknown protein |
| 120558 | 0.000372 | 9.40329 | | 11.88043 | | 10.14501 | 1.672 down | | 3.329 up | | 5.567 up | unknown protein |
| 109273 | 0.0022 | 6.79558 | | 9.31041 | | 7.5333 | 1.667 down | | 3.427 up | | 5.715 up | unknown protein |
| 67052 | 0.000875 | 8.84785 | | 11.21611 | | 9.58536 | 1.667 down | | 3.096 up | | 5.163 up | unknown protein |
| 32069 | 0.00174 | 10.47116 | | 12.87568 | | 11.20773 | 1.666 down | | 3.177 up | | 5.294 up | unknown protein |
| 30635 | 0.00114 | 9.74994 | | 10.68514 | | 9.01658 | 1.662 up | | 3.178 up | | 1.912 up | unknown protein |
| 110382 | 0.00158 | 9.19891 | | 9.85715 | | 8.4653 | 1.662 up | | 2.624 up | | 1.578 up | unknown protein |
| 107245 | 0.00128 | 11.3814 | | 12.63523 | | 10.64862 | 1.661 up | | 3.963 up | | 2.384 up | unknown protein |
| 105196 | 0.000497 | 11.6218 | | 13.16029 | | 10.89243 | 1.657 up | | 4.816 up | | 2.904 up | unknown protein |
| 108962 | 0.000937 | 10.01959 | | 11.08052 | | 9.29027 | 1.657 up | | 3.458 up | | 2.086 up | unknown protein |
| 44306 | 0.000416 | 8.95861 | | 11.75653 | | 8.23031 | 1.656 up | | 11.521 up | | 6.954 up | unknown protein |
| 109199 | 0.00193 | 7.10266 | | 7.98302 | | 6.37604 | 1.654 up | | 3.046 up | | 1.840 up | unknown protein |
| 103712 | 0.000144 | 6.25556 | | 9.10785 | | 6.98102 | 1.653 down | | 4.367 up | | 7.221 up | unknown protein |
| 111632 | 0.000637 | 8.70658 | | 11.14038 | | 7.98457 | 1.649 up | | 8.912 up | | 5.403 up | unknown protein |
| 107551 | 0.0221 | 9.50393 | | 11.57207 | | 8.78615 | 1.644 up | | 6.896 up | | 4.193 up | unknown protein |
| 69222 | 0.00126 | 9.57511 | | 12.16733 | | 10.29263 | 1.644 down | | 3.667 up | | 6.030 up | unknown protein |
| 58475 | 0.00105 | 6.05333 | | 7.77856 | | 5.34142 | 1.637 up | | 5.415 up | | 3.306 up | unknown protein |
| 109004 | 0.000497 | 11.08454 | | 11.80438 | | 10.3739 | 1.636 up | | 2.695 up | | 1.647 up | unknown protein |
| 80756 | 0.00303 | 12.09023 | | 12.74403 | | 11.38182 | 1.634 up | | 2.570 up | | 1.573 up | unknown protein |
| 104291 | 0.000324 | 7.52865 | | 10.71972 | | 8.23629 | 1.633 down | | 5.592 up | | 9.132 up | unknown protein |
| 122576 | 0.000161 | 10.38271 | | 12.93895 | | 9.67539 | 1.632 up | | 9.603 up | | 5.881 up | unknown protein |
| 123426 | 0.000812 | 11.84608 | | 12.75292 | | 11.14044 | 1.630 up | | 3.057 up | | 1.874 up | unknown protein |
| 40343 | 0.0003 | 10.92628 | | 11.67454 | | 10.22249 | 1.628 up | | 2.735 up | | 1.679 up | unknown protein |
| 121553 | 0.000254 | 10.40156 | | 12.47864 | | 9.69995 | 1.626 up | | 6.862 up | | 4.219 up | unknown protein |
| 107616 | 0.00122 | 10.96243 | | 11.61887 | | 10.26099 | 1.626 up | | 2.563 up | | 1.576 up | unknown protein |
| 78343 | 0.000507 | 8.7434 | | 11.79005 | | 9.44559 | 1.626 down | | 5.078 up | | 8.262 up | unknown protein |
| 56376 | 0.000291 | 7.8597 | | 10.74861 | | 8.56186 | 1.626 down | | 4.552 up | | 7.407 up | unknown protein |
| 109323 | 0.000744 | 6.37569 | | 7.92584 | | 5.67441 | 1.625 up | | 4.761 up | | 2.928 up | unknown protein |
| 59417 | 0.00318 | 10.35956 | | 11.59005 | | 9.65879 | 1.625 up | | 3.813 up | | 2.346 up | unknown protein |
| 61576 | 0.000616 | 13.17811 | | 13.90117 | | 12.47695 | 1.625 up | | 2.683 up | | 1.650 up | unknown protein |
| 80282 | 0.00244 | 13.34245 | | 13.99571 | | 12.6468 | 1.619 up | | 2.547 up | | 1.572 up | unknown protein |
| 22741 | 0.000877 | 10.19135 | | 11.64396 | | 9.4966 | 1.618 up | | 4.430 up | | 2.737 up | unknown protein |
| 67079 | 0.00188 | 7.90212 | | 8.64338 | | 7.21064 | 1.614 up | | 2.699 up | | 1.671 up | unknown protein |
| 31415 | 0.000357 | 10.17314 | | 12.44687 | | 10.86364 | 1.613 down | | 2.996 up | | 4.835 up | unknown protein |
| 109994 | 0.00235 | 8.71045 | | 11.09375 | | 8.02519 | 1.607 up | | 8.389 up | | 5.217 up | unknown protein |
| 103080 | 0.000723 | 9.82384 | | 11.25425 | | 9.13906 | 1.607 up | | 4.332 up | | 2.695 up | unknown protein |
| 120416 | 0.00122 | 11.37088 | | 12.38458 | | 10.68616 | 1.607 up | | 3.245 up | | 2.019 up | unknown protein |
| 38573 | 0.00328 | 8.34373 | | 9.17251 | | 7.66244 | 1.603 up | | 2.848 up | | 1.776 up | unknown protein |
| 112490 | 0.00514 | 7.17367 | | 8.18743 | | 6.49331 | 1.602 up | | 3.235 up | | 2.019 up | unknown protein |
| 42346 | 0.000769 | 10.43373 | | 12.43844 | | 11.11368 | 1.602 down | | 2.504 up | | 4.013 up | unknown protein |
| 55575 | 0.000221 | 10.05169 | | 13.04016 | | 10.73055 | 1.600 down | | 4.957 up | | 7.936 up | unknown protein |
| 68497 | 0.000146 | 11.36559 | | 12.33556 | | 10.6905 | 1.596 up | | 3.127 up | | 1.958 up | unknown protein |
| 107168 | 0.00185 | 5.89258 | | 7.18131 | | 5.22015 | 1.593 up | | 3.893 up | | 2.443 up | unknown protein |
| 40519 | 0.000679 | 8.78784 | | 11.88439 | | 9.46028 | 1.593 down | | 5.367 up | | 8.553 up | unknown protein |
| 55668 | 0.00545 | 10.67667 | | 12.33171 | | 10.00844 | 1.589 up | | 5.004 up | | 3.149 up | unknown protein |
| 80741 | 0.00348 | 8.33621 | | 9.70977 | | 7.66845 | 1.588 up | | 4.116 up | | 2.591 up | unknown protein |
| 81889 | 0.00176 | 11.65633 | | 12.56076 | | 10.99123 | 1.585 up | | 2.968 up | | 1.871 up | unknown protein |
| 44943 | 0.00163 | 13.03909 | | 13.88412 | | 12.37511 | 1.584 up | | 2.846 up | | 1.796 up | unknown protein |
| 108645 | 0.000439 | 8.08945 | | 11.3667 | | 8.7499 | 1.580 down | | 6.133 up | | 9.695 up | unknown protein |
| 61632 | 0.0012 | 11.08257 | | 11.89737 | | 10.42716 | 1.575 up | | 2.770 up | | 1.759 up | unknown protein |
| 67562 | 0.00199 | 11.58357 | | 14.0503 | | 12.23928 | 1.575 down | | 3.508 up | | 5.527 up | unknown protein |
| 103129 | 0.0228 | 4.97755 | | 5.65217 | | 4.32384 | 1.573 up | | 2.511 up | | 1.596 up | unknown protein |
| 111095 | 0.0000549 | 7.57077 | | 10.28803 | | 6.91638 | 1.573 up | | 10.350 up | | 6.576 up | unknown protein |
| 102482 | 0.00145 | 9.13811 | | 11.80485 | | 9.79179 | 1.573 down | | 4.036 up | | 6.349 up | unknown protein |
| 45252 | 0.000579 | 11.22624 | | 13.72343 | | 11.87739 | 1.570 down | | 3.595 up | | 5.645 up | unknown protein |
| 103463 | 0.000819 | 5.76145 | | 7.01972 | | 5.11083 | 1.569 up | | 3.755 up | | 2.392 up | unknown protein |
| 59936 | 0.000546 | 9.18157 | | 11.2103 | | 9.8294 | 1.566 down | | 2.604 up | | 4.080 up | unknown protein |
| 53076 | 0.00319 | 8.64818 | | 10.07469 | | 8.00258 | 1.564 up | | 4.205 up | | 2.687 up | unknown protein |
| 103065 | 0.000145 | 6.67395 | | 10.05741 | | 7.32002 | 1.564 down | | 6.668 up | | 10.435 up | unknown protein |
| 110509 | 0.000775 | 10.43277 | | 11.4066 | | 9.78869 | 1.562 up | | 3.069 up | | 1.964 up | unknown protein |
| 56614 | 0.00189 | 9.70371 | | 11.87862 | | 9.06155 | 1.560 up | | 7.047 up | | 4.515 up | unknown protein |
| 121889 | 0.00317 | 12.78611 | | 13.88963 | | 12.14676 | 1.557 up | | 3.347 up | | 2.148 up | unknown protein |
| 109087 | 0.00039 | 7.97525 | | 8.96187 | | 7.33583 | 1.557 up | | 3.086 up | | 1.981 up | unknown protein |
| 120654 | 0.00277 | 13.38003 | | 14.07968 | | 12.74389 | 1.554 up | | 2.524 up | | 1.624 up | unknown protein |
| 22661 | 0.00334 | 11.36766 | | 13.18765 | | 10.73528 | 1.550 up | | 5.473 up | | 3.530 up | unknown protein |
| 121579 | 0.000723 | 11.14537 | | 12.09838 | | 10.51244 | 1.550 up | | 3.002 up | | 1.935 up | unknown protein |
| 40338 | 0.00024 | 9.31434 | | 11.38548 | | 9.94419 | 1.547 down | | 2.715 up | | 4.202 up | unknown protein |
| 62826 | 0.000446 | 10.3243 | | 12.90151 | | 9.69545 | 1.546 up | | 9.228 up | | 5.967 up | unknown protein |
| 122833 | 0.00257 | 12.43871 | | 13.75678 | | 11.81187 | 1.544 up | | 3.850 up | | 2.493 up | unknown protein |
| 108309 | 0.000537 | 11.87006 | | 13.00934 | | 11.24351 | 1.543 up | | 3.400 up | | 2.202 up | unknown protein |
| 52539 | 0.00154 | 11.33364 | | 12.23598 | | 10.70763 | 1.543 up | | 2.884 up | | 1.869 up | unknown protein |
| 5578 | 0.000405 | 9.54359 | | 11.79492 | | 10.16964 | 1.543 down | | 3.085 up | | 4.761 up | unknown protein |
| 82599 | 0.00134 | 11.65929 | | 12.79178 | | 11.03383 | 1.542 up | | 3.382 up | | 2.192 up | unknown protein |
| 53431 | 0.0153 | 8.29778 | | 10.27293 | | 8.92265 | 1.542 down | | 2.549 up | | 3.931 up | unknown protein |
| 5058 | 0.0032 | 9.30401 | | 11.53759 | | 9.92836 | 1.541 down | | 3.050 up | | 4.703 up | unknown protein |
| 23347 | 0.000103 | 10.54713 | | 12.50184 | | 9.92348 | 1.540 up | | 5.972 up | | 3.876 up | unknown protein |
| 63733 | 0.000218 | 8.98296 | | 11.66022 | | 8.36053 | 1.539 up | | 9.847 up | | 6.396 up | unknown protein |
| 102947 | 0.000263 | 8.81639 | | 11.17745 | | 8.1969 | 1.536 up | | 7.892 up | | 5.137 up | unknown protein |
| 55838 | 0.00103 | 11.36845 | | 13.63803 | | 11.98713 | 1.535 down | | 3.140 up | | 4.821 up | unknown protein |
| 106605 | 0.00205 | 12.13596 | | 13.11071 | | 11.52134 | 1.531 up | | 3.009 up | | 1.965 up | unknown protein |
| 108635 | 0.00108 | 6.1576 | | 7.92873 | | 5.54354 | 1.530 up | | 5.224 up | | 3.413 up | unknown protein |
| 108866 | 0.000817 | 8.15349 | | 10.26633 | | 8.76767 | 1.530 down | | 2.825 up | | 4.325 up | unknown protein |
| 53893 | 0.0015 | 11.01761 | | 13.01086 | | 11.62749 | 1.526 down | | 2.608 up | | 3.981 up | unknown protein |
| 66877 | 0.000517 | 9.94855 | | 11.21805 | | 9.34006 | 1.524 up | | 3.675 up | | 2.410 up | unknown protein |
| 41690 | 0.00167 | 8.70043 | | 10.71633 | | 9.30856 | 1.524 down | | 2.653 up | | 4.044 up | unknown protein |
| 103073 | 0.000506 | 5.68498 | | 8.06599 | | 6.29178 | 1.522 down | | 3.420 up | | 5.208 up | unknown protein |
| 106055 | 0.00429 | 9.57523 | | 11.31545 | | 8.97019 | 1.521 up | | 5.081 up | | 3.340 up | unknown protein |
| 111638 | 0.000485 | 10.42493 | | 11.96326 | | 9.81967 | 1.521 up | | 4.418 up | | 2.904 up | unknown protein |
| 68548 | 0.000427 | 10.22897 | | 11.64022 | | 9.6266 | 1.518 up | | 4.037 up | | 2.659 up | unknown protein |
| 79361 | 0.000338 | 9.99677 | | 12.90413 | | 10.59266 | 1.511 down | | 4.963 up | | 7.502 up | unknown protein |
| 22284 | 0.000126 | 12.53758 | | 13.86086 | | 11.94396 | 1.509 up | | 3.776 up | | 2.502 up | unknown protein |
| 105975 | 0.000808 | 7.89431 | | 8.6976 | | 7.3005 | 1.509 up | | 2.633 up | | 1.745 up | unknown protein |
| 35768 | 0.00107 | 10.3306 | | 11.99232 | | 9.73842 | 1.507 up | | 4.769 up | | 3.163 up | unknown protein |
| 39351 | 0.00108 | 6.4601 | | 7.83364 | | 5.87178 | 1.503 up | | 3.895 up | | 2.591 up | unknown protein |
| 106636 | 0.000661 | 8.51983 | | 10.21204 | | 7.93266 | 1.502 up | | 4.854 up | | 3.231 up | unknown protein |
| 121569 | 0.00286 | 11.93818 | | 12.70962 | | 11.35144 | 1.501 up | | 2.563 up | | 1.706 up | Unknown protein |
| 66751 | 0.00031 | 5.10687 | | 7.52347 | | 5.69287 | 1.501 down | | 3.556 up | | 5.339 up | unknown protein |
| 74946 | 0.000449 | 12.038 | | 12.81083 | | 11.4581 | 1.494 up | | 2.553 up | | 1.708 up | unknown protein |
| 106493 | 0.00257 | 2.2827 | | 5.42199 | | 2.86247 | 1.494 down | | 5.895 up | | 8.810 up | unknown protein |
| 74987 | 0.0031 | 11.10045 | | 13.19916 | | 11.67772 | 1.492 down | | 2.870 up | | 4.283 up | unknown protein |
| 79085 | 0.00302 | 12.64121 | | 13.66307 | | 12.06621 | 1.489 up | | 3.024 up | | 2.030 up | unknown protein |
| 122501 | 0.000226 | 10.98568 | | 12.00267 | | 10.41254 | 1.487 up | | 3.010 up | | 2.023 up | unknown protein |
| 106731 | 0.000594 | 7.58334 | | 9.7466 | | 8.1552 | 1.486 down | | 3.013 up | | 4.479 up | unknown protein |
| 105466 | 0.0108 | 9.63774 | | 10.68499 | | 9.06727 | 1.485 up | | 3.068 up | | 2.066 up | unknown protein |
| 104356 | 0.00313 | 9.49218 | | 11.41871 | | 10.06006 | 1.482 down | | 2.564 up | | 3.801 up | unknown protein |
| 47670 | 0.00539 | 11.25955 | | 12.35475 | | 10.69415 | 1.479 up | | 3.161 up | | 2.136 up | unknown protein |
| 56469 | 0.00301 | 11.28545 | | 12.37925 | | 10.72006 | 1.479 up | | 3.158 up | | 2.134 up | unknown protein |
| 119704 | 0.000462 | 9.91754 | | 11.99675 | | 10.48206 | 1.478 down | | 2.857 up | | 4.225 up | unknown protein |
| 110140 | 0.00218 | 6.90092 | | 8.38453 | | 6.33729 | 1.477 up | | 4.133 up | | 2.796 up | unknown protein |
| 4952 | 0.00391 | 6.58519 | | 7.76902 | | 6.0235 | 1.476 up | | 3.353 up | | 2.271 up | unknown protein |
| 69013 | 0.00407 | 11.07288 | | 11.90557 | | 10.51144 | 1.475 up | | 2.628 up | | 1.781 up | unknown protein |
| 106342 | 0.000194 | 9.09282 | | 11.89898 | | 9.65361 | 1.475 down | | 4.741 up | | 6.994 up | unknown protein |
| 103995 | 0.00356 | 11.0246 | | 12.98582 | | 11.57633 | 1.465 down | | 2.656 up | | 3.893 up | unknown protein |
| 65816 | 0.00122 | 10.48966 | | 11.61318 | | 9.94058 | 1.463 up | | 3.187 up | | 2.178 up | unknown protein |
| 103282 | 0.00169 | 11.64139 | | 12.64505 | | 11.09429 | 1.461 up | | 2.929 up | | 2.005 up | unknown protein |
| 66353 | 0.00112 | 11.57892 | | 12.74978 | | 11.03385 | 1.459 up | | 3.285 up | | 2.251 up | unknown protein |
| 109846 | 0.000444 | 6.92152 | | 8.28637 | | 6.37689 | 1.458 up | | 3.756 up | | 2.575 up | unknown protein |
| 69267 | 0.00127 | 11.49695 | | 12.50094 | | 10.95459 | 1.456 up | | 2.920 up | | 2.005 up | unknown protein |
| 122067 | 0.000944 | 9.57332 | | 11.49038 | | 10.1162 | 1.456 down | | 2.592 up | | 3.776 up | unknown protein |
| 107869 | 0.00231 | 5.119 | | 6.75718 | | 4.57755 | 1.455 up | | 4.530 up | | 3.112 up | unknown protein |
| 105423 | 0.000306 | 9.40194 | | 12.1007 | | 9.94283 | 1.454 down | | 4.462 up | | 6.492 up | unknown protein |
| 61420 | 0.000114 | 9.96168 | | 11.19873 | | 9.42209 | 1.453 up | | 3.426 up | | 2.357 up | unknown protein |
| 123343 | 0.00284 | 9.32623 | | 10.34207 | | 8.78711 | 1.453 up | | 2.938 up | | 2.022 up | unknown protein |
| 122897 | 0.00346 | 11.11273 | | 12.51235 | | 10.57387 | 1.452 up | | 3.833 up | | 2.638 up | unknown protein |
| 74932 | 0.000361 | 8.82535 | | 11.77402 | | 9.36371 | 1.452 down | | 5.315 up | | 7.720 up | unknown protein |
| 53501 | 0.000731 | 11.70747 | | 12.85253 | | 11.17031 | 1.451 up | | 3.209 up | | 2.211 up | unknown protein |
| 69316 | 0.00129 | 9.77233 | | 11.47218 | | 9.23626 | 1.450 up | | 4.710 up | | 3.248 up | unknown protein |
| 59028 | 0.000183 | 6.69451 | | 10.02144 | | 7.23005 | 1.449 down | | 6.922 up | | 10.034 up | unknown protein |
| 58535 | 0.0023 | 3.47976 | | 5.5127 | | 4.01548 | 1.449 down | | 2.822 up | | 4.092 up | unknown protein |
| 53226 | 0.000434 | 9.58693 | | 11.9626 | | 10.11602 | 1.443 down | | 3.596 up | | 5.189 up | unknown protein |
| 54710 | 0.000329 | 11.93884 | | 12.81995 | | 11.41131 | 1.441 up | | 2.654 up | | 1.841 up | unknown protein |
| 105854 | 0.00364 | 11.10735 | | 12.22826 | | 10.58646 | 1.434 up | | 3.120 up | | 2.174 up | unknown protein |
| 103651 | 0.00249 | 3.33391 | | 5.25097 | | 3.85444 | 1.434 down | | 2.632 up | | 3.776 up | unknown protein |
| 77167 | 0.00006 | 9.3735 | | 10.99602 | | 8.85534 | 1.432 up | | 4.409 up | | 3.079 up | unknown protein |
| 67988 | 0.000781 | 7.16674 | | 9.22318 | | 7.68457 | 1.431 down | | 2.905 up | | 4.159 up | unknown protein |
| 68106 | 0.00122 | 8.18816 | | 10.84549 | | 8.70453 | 1.430 down | | 4.410 up | | 6.308 up | unknown protein |
| 106296 | 0.000245 | 5.98292 | | 8.23382 | | 5.46844 | 1.428 up | | 6.799 up | | 4.759 up | unknown protein |
| 110773 | 0.00114 | 5.78264 | | 7.62212 | | 6.29583 | 1.427 down | | 2.507 up | | 3.578 up | unknown protein |
| 63125 | 0.000515 | 8.53342 | | 9.60168 | | 8.02053 | 1.426 up | | 2.992 up | | 2.096 up | unknown protein |
| 31447 | 0.0161 | 4.6993 | | 6.5594 | | 5.21159 | 1.426 down | | 2.545 up | | 3.630 up | unknown protein |
| 103437 | 0.00042 | 7.74842 | | 9.99437 | | 7.23708 | 1.425 up | | 6.761 up | | 4.743 up | unknown protein |
| 68425 | 0.00158 | 9.34767 | | 10.96819 | | 8.83589 | 1.425 up | | 4.384 up | | 3.074 up | unknown protein |
| 54868 | 0.00423 | 11.03967 | | 12.03975 | | 10.53147 | 1.422 up | | 2.844 up | | 2.000 up | unknown protein |
| 21221 | 0.00142 | 11.39038 | | 12.41191 | | 10.88324 | 1.421 up | | 2.885 up | | 2.030 up | unknown protein |
| 57397 | 0.000308 | 6.76371 | | 9.52078 | | 7.27086 | 1.421 down | | 4.756 up | | 6.760 up | unknown protein |
| 47167 | 0.000657 | 11.83416 | | 12.80972 | | 11.32885 | 1.419 up | | 2.791 up | | 1.966 up | unknown protein |
| 111884 | 0.000369 | 4.89161 | | 5.99777 | | 4.38676 | 1.418 up | | 3.054 up | | 2.152 up | unknown protein |
| 107082 | 0.00134 | 7.78 | | 9.76839 | | 8.28447 | 1.418 down | | 2.797 up | | 3.967 up | unknown protein |
| 62663 | 0.000189 | 7.26078 | | 10.73548 | | 7.76451 | 1.417 down | | 7.840 up | | 11.116 up | unknown protein |
| 106249 | 0.000406 | 9.83966 | | 12.15699 | | 10.34257 | 1.417 down | | 3.517 up | | 4.984 up | unknown protein |
| 69375 | 0.000808 | 9.23999 | | 11.49857 | | 8.7385 | 1.415 up | | 6.774 up | | 4.785 up | unknown protein |
| 21747 | 0.000223 | 7.5029 | | 8.81508 | | 7.00157 | 1.415 up | | 3.514 up | | 2.483 up | unknown protein |
| 3580 | 0.000222 | 9.77064 | | 10.66718 | | 9.26972 | 1.415 up | | 2.634 up | | 1.861 up | unknown protein |
| 45512 | 0.00116 | 10.57631 | | 12.22036 | | 10.07562 | 1.414 up | | 4.422 up | | 3.125 up | unknown protein |
| 51650 | 0.000254 | 13.58819 | | 14.47995 | | 13.08749 | 1.414 up | | 2.625 up | | 1.855 up | unknown protein |
| 58857 | 0.00544 | 7.93659 | | 8.79431 | | 7.4379 | 1.412 up | | 2.560 up | | 1.812 up | unknown protein |
| 4290 | 0.00295 | 11.32593 | | 12.92254 | | 10.82904 | 1.411 up | | 4.267 up | | 3.024 up | unknown protein |
| 106659 | 0.00132 | 8.96852 | | 9.94978 | | 8.47097 | 1.411 up | | 2.787 up | | 1.974 up | unknown protein |
| 69825 | 0.000632 | 8.08231 | | 11.07805 | | 8.57944 | 1.411 down | | 5.651 up | | 7.976 up | unknown protein |
| 63011 | 0.000115 | 10.73489 | | 13.05204 | | 10.2422 | 1.407 up | | 7.012 up | | 4.983 up | unknown protein |
| 122666 | 0.000899 | 13.01401 | | 13.91978 | | 12.5265 | 1.402 up | | 2.626 up | | 1.873 up | unknown protein |
| 105291 | 0.00398 | 5.50566 | | 9.21365 | | 5.98905 | 1.398 down | | 9.347 up | | 13.068 up | unknown protein |
| 122995 | 0.000435 | 8.00094 | | 10.37899 | | 7.51847 | 1.397 up | | 7.262 up | | 5.198 up | unknown protein |
| 65760 | 0.0043 | 11.27758 | | 12.20011 | | 10.79806 | 1.394 up | | 2.642 up | | 1.895 up | unknown protein |
| 123842 | 0.00108 | 11.04146 | | 12.68554 | | 10.56968 | 1.386 up | | 4.334 up | | 3.125 up | unknown protein |
| 110651 | 0.000723 | 9.95288 | | 11.32542 | | 9.48148 | 1.386 up | | 3.589 up | | 2.589 up | unknown protein |
| 105385 | 0.00437 | 4.13879 | | 5.16828 | | 3.66759 | 1.386 up | | 2.829 up | | 2.041 up | unknown protein |
| 62340 | 0.000712 | 9.33645 | | 11.30057 | | 9.80829 | 1.386 down | | 2.813 up | | 3.901 up | unknown protein |
| 109284 | 0.00341 | 4.40685 | | 5.7933 | | 3.93781 | 1.384 up | | 3.618 up | | 2.614 up | unknown protein |
| 66438 | 0.00556 | 12.53303 | | 13.64544 | | 12.06407 | 1.384 up | | 2.992 up | | 2.162 up | unknown protein |
| 81939 | 0.00131 | 11.49684 | | 12.93511 | | 11.02852 | 1.383 up | | 3.749 up | | 2.709 up | unknown protein |
| 25947 | 0.000482 | 9.61757 | | 12.42965 | | 10.08555 | 1.383 down | | 5.077 up | | 7.022 up | unknown protein |
| 106457 | 0.000902 | 8.65284 | | 11.92844 | | 8.18525 | 1.382 up | | 13.390 up | | 9.683 up | unknown protein |
| 75580 | 0.000213 | 10.00351 | | 12.10274 | | 10.47099 | 1.382 down | | 3.098 up | | 4.284 up | unknown protein |
| 55671 | 0.00745 | 4.36509 | | 5.8228 | | 3.89946 | 1.380 up | | 3.792 up | | 2.746 up | unknown protein |
| 112609 | 0.000708 | 8.12249 | | 10.01608 | | 7.65844 | 1.379 up | | 5.125 up | | 3.715 up | unknown protein |
| 39753 | 0.00398 | 7.60739 | | 10.01567 | | 8.07166 | 1.379 down | | 3.847 up | | 5.308 up | unknown protein |
| 112080 | 0.00358 | 9.61871 | | 10.82852 | | 9.15605 | 1.378 up | | 3.187 up | | 2.313 up | unknown protein |
| 70921 | 0.0026 | 5.99464 | | 7.52602 | | 5.53258 | 1.377 up | | 3.981 up | | 2.890 up | unknown protein |
| 57078 | 0.000369 | 8.17663 | | 10.82072 | | 7.71654 | 1.375 up | | 8.599 up | | 6.251 up | unknown protein |
| 21737 | 0.0134 | 12.96124 | | 13.84701 | | 12.50386 | 1.373 up | | 2.537 up | | 1.847 up | unknown protein |
| 77193 | 0.00147 | 11.48898 | | 12.73513 | | 11.03352 | 1.371 up | | 3.252 up | | 2.372 up | unknown protein |
| 80733 | 0.000622 | 6.62559 | | 9.00952 | | 7.08013 | 1.370 down | | 3.808 up | | 5.219 up | unknown protein |
| 60867 | 0.00667 | 11.62882 | | 12.72858 | | 11.17498 | 1.369 up | | 2.935 up | | 2.143 up | unknown protein |
| 106801 | 0.0048 | 10.74283 | | 12.52341 | | 11.19618 | 1.369 down | | 2.509 up | | 3.435 up | unknown protein |
| 46493 | 0.00398 | 7.19868 | | 9.1159 | | 7.65168 | 1.368 down | | 2.759 up | | 3.776 up | unknown protein |
| 65360 | 0.000444 | 10.70897 | | 12.47636 | | 10.25788 | 1.367 up | | 4.654 up | | 3.404 up | unknown protein |
| 55709 | 0.00293 | 8.95866 | | 10.36601 | | 8.50763 | 1.367 up | | 3.625 up | | 2.652 up | unknown protein |
| 123255 | 0.000744 | 12.91023 | | 14.06167 | | 12.45834 | 1.367 up | | 3.038 up | | 2.221 up | unknown protein |
| 78061 | 0.00107 | 12.03915 | | 13.22323 | | 11.5911 | 1.364 up | | 3.099 up | | 2.272 up | unknown protein |
| 77998 | 0.000111 | 9.87042 | | 13.1358 | | 9.42444 | 1.362 up | | 13.098 up | | 9.615 up | unknown protein |
| 60095 | 0.000754 | 9.43273 | | 11.24716 | | 9.87797 | 1.361 down | | 2.583 up | | 3.517 up | unknown protein |
| 57730 | 0.000493 | 11.38399 | | 13.03094 | | 10.93976 | 1.360 up | | 4.260 up | | 3.131 up | unknown protein |
| 21152 | 0.0082 | 11.00977 | | 12.02149 | | 10.56563 | 1.360 up | | 2.743 up | | 2.016 up | unknown protein |
| 22341 | 0.000203 | 11.49583 | | 12.86127 | | 11.05305 | 1.359 up | | 3.502 up | | 2.576 up | unknown protein |
| 110220 | 0.000262 | 8.84917 | | 11.17411 | | 8.40678 | 1.358 up | | 6.808 up | | 5.010 up | unknown protein |
| 104838 | 0.0024 | 7.81738 | | 10.51222 | | 8.25461 | 1.354 down | | 4.781 up | | 6.474 up | unknown protein |
| 74390 | 0.00364 | 5.27865 | | 7.90877 | | 5.71447 | 1.352 down | | 4.576 up | | 6.190 up | unknown protein |
| 124249 | 0.0162 | 10.6457 | | 11.75259 | | 10.21094 | 1.351 up | | 2.911 up | | 2.153 up | unknown protein |
| 54890 | 0.00157 | 11.87259 | | 12.85095 | | 11.43792 | 1.351 up | | 2.662 up | | 1.970 up | unknown protein |
| 65046 | 0.000728 | 9.01718 | | 10.86286 | | 8.58627 | 1.348 up | | 4.845 up | | 3.594 up | unknown protein |
| 32543 | 0.00126 | 10.07107 | | 11.3728 | | 9.63936 | 1.348 up | | 3.325 up | | 2.465 up | unknown protein |
| 62314 | 0.00296 | 8.62303 | | 10.50732 | | 8.19403 | 1.346 up | | 4.970 up | | 3.691 up | unknown protein |
| 61771 | 0.00178 | 9.61878 | | 10.76767 | | 9.19135 | 1.344 up | | 2.982 up | | 2.217 up | unknown protein |
| 105994 | 0.00704 | 12.67358 | | 13.65159 | | 12.24606 | 1.344 up | | 2.649 up | | 1.969 up | unknown protein |
| 122176 | 0.00227 | 11.06059 | | 12.81274 | | 10.63472 | 1.343 up | | 4.525 up | | 3.368 up | unknown protein |
| 112502 | 0.000175 | 11.82984 | | 13.50506 | | 11.40349 | 1.343 up | | 4.291 up | | 3.193 up | unknown protein |
| 102599 | 0.0133 | 9.78269 | | 10.86386 | | 9.35654 | 1.343 up | | 2.842 up | | 2.115 up | unknown protein |
| 81465 | 0.00122 | 11.35086 | | 12.57317 | | 10.93181 | 1.337 up | | 3.119 up | | 2.333 up | unknown protein |
| 55818 | 0.0012 | 10.24705 | | 12.14719 | | 10.66473 | 1.335 down | | 2.794 up | | 3.732 up | unknown protein |
| 119779 | 0.015 | 5.08566 | | 6.07565 | | 4.66974 | 1.334 up | | 2.649 up | | 1.986 up | unknown protein |
| 110023 | 0.000999 | 9.70304 | | 11.69284 | | 10.11712 | 1.332 down | | 2.980 up | | 3.971 up | unknown protein |
| 73587 | 0.000603 | 11.43665 | | 13.44034 | | 11.84989 | 1.331 down | | 3.011 up | | 4.010 up | unknown protein |
| 104691 | 0.00223 | 11.70103 | | 12.8388 | | 11.29164 | 1.328 up | | 2.922 up | | 2.200 up | unknown protein |
| 107996 | 0.000726 | 7.60075 | | 9.35132 | | 7.19216 | 1.327 up | | 4.466 up | | 3.364 up | unknown protein |
| 73048 | 0.000843 | 13.23884 | | 14.2746 | | 12.83058 | 1.327 up | | 2.720 up | | 2.050 up | unknown protein |
| 78843 | 0.000349 | 10.87332 | | 12.89275 | | 11.28072 | 1.326 down | | 3.056 up | | 4.054 up | unknown protein |
| 103180 | 0.00123 | 9.19143 | | 10.23013 | | 8.78522 | 1.325 up | | 2.722 up | | 2.054 up | unknown protein |
| 27939 | 0.00037 | 10.60898 | | 12.1985 | | 10.2068 | 1.321 up | | 3.977 up | | 3.009 up | unknown protein |
| 79779 | 0.00112 | 8.11985 | | 10.41901 | | 8.52208 | 1.321 down | | 3.724 up | | 4.921 up | unknown protein |
| 64322 | 0.00109 | 10.29587 | | 11.40459 | | 9.8952 | 1.320 up | | 2.846 up | | 2.156 up | unknown protein |
| 30068 | 0.00329 | 7.86863 | | 9.10883 | | 7.46818 | 1.319 up | | 3.118 up | | 2.362 up | unknown protein |
| 103947 | 0.000589 | 10.93928 | | 12.18153 | | 10.54171 | 1.317 up | | 3.116 up | | 2.365 up | unknown protein |
| 65095 | 0.00399 | 5.17312 | | 6.4223 | | 4.7761 | 1.316 up | | 3.130 up | | 2.377 up | unknown protein |
| 66719 | 0.0221 | 10.07246 | | 11.97276 | | 10.46651 | 1.314 down | | 2.840 up | | 3.732 up | unknown protein |
| 29142 | 0.000164 | 10.60353 | | 12.04364 | | 10.20995 | 1.313 up | | 3.564 up | | 2.713 up | unknown protein |
| 1912 | 0.000483 | 11.9052 | | 13.00153 | | 11.51145 | 1.313 up | | 2.809 up | | 2.138 up | unknown protein |
| 22875 | 0.00436 | 11.94954 | | 12.9498 | | 11.556 | 1.313 up | | 2.627 up | | 2.000 up | unknown protein |
| 108681 | 0.00169 | 4.07425 | | 5.91739 | | 3.68456 | 1.310 up | | 4.700 up | | 3.587 up | unknown protein |
| 53870 | 0.000291 | 9.41557 | | 11.70909 | | 9.80522 | 1.310 down | | 3.742 up | | 4.902 up | unknown protein |
| 59302 | 0.00207 | 10.75442 | | 12.94724 | | 11.14356 | 1.309 down | | 3.491 up | | 4.571 up | unknown protein |
| 110523 | 0.00151 | 10.13163 | | 12.11449 | | 10.52011 | 1.309 down | | 3.019 up | | 3.952 up | unknown protein |
| 120236 | 0.000432 | 11.44915 | | 12.94584 | | 11.06471 | 1.305 up | | 3.683 up | | 2.821 up | unknown protein |
| 78242 | 0.00129 | 10.52017 | | 12.78275 | | 10.13688 | 1.304 up | | 6.258 up | | 4.798 up | unknown protein |
| 112494 | 0.000515 | 3.81065 | | 5.74133 | | 4.19418 | 1.304 down | | 2.922 up | | 3.812 up | unknown protein |
| 105079 | 0.00313 | 10.31747 | | 11.66033 | | 9.93552 | 1.303 up | | 3.305 up | | 2.536 up | unknown protein |
| 64044 | 0.000308 | 9.11265 | | 11.90813 | | 8.73259 | 1.301 up | | 9.035 up | | 6.942 up | unknown protein |
| 42418 | 0.00107 | 9.8324 | | 12.01268 | | 10.21217 | 1.301 down | | 3.483 up | | 4.532 up | unknown protein |
| 104651 | 0.00176 | 9.49492 | | 11.43576 | | 9.87473 | 1.301 down | | 2.950 up | | 3.839 up | unknown protein |
| 60956 | 0.00437 | 11.30765 | | 12.90188 | | 10.92912 | 1.300 up | | 3.925 up | | 3.019 up | unknown protein |
| 102779 | 0.0009 | 6.02706 | | 8.02016 | | 6.39933 | 1.294 down | | 3.075 up | | 3.980 up | unknown protein |
| 69118 | 0.00079 | 10.35818 | | 12.12698 | | 10.72823 | 1.292 down | | 2.636 up | | 3.407 up | unknown protein |
| 55406 | 0.000803 | 10.85063 | | 12.09977 | | 10.48164 | 1.291 up | | 3.069 up | | 2.377 up | unknown protein |
| 108530 | 0.00261 | 9.81199 | | 11.05428 | | 9.44339 | 1.291 up | | 3.054 up | | 2.365 up | unknown protein |
| 75566 | 0.000496 | 10.77685 | | 12.25385 | | 10.41169 | 1.288 up | | 3.585 up | | 2.783 up | unknown protein |
| 1949 | 0.00228 | 9.0443 | | 11.01122 | | 9.40428 | 1.283 down | | 3.046 up | | 3.909 up | unknown protein |
| 103205 | 0.000148 | 6.71838 | | 10.47832 | | 6.36254 | 1.279 up | | 17.337 up | | 13.547 up | unknown protein |
| 57291 | 0.00088 | 10.43346 | | 12.40996 | | 10.78892 | 1.279 down | | 3.075 up | | 3.935 up | unknown protein |
| 104469 | 0.00167 | 12.73098 | | 14.16043 | | 12.37636 | 1.278 up | | 3.443 up | | 2.693 up | unknown protein |
| 123144 | 0.00221 | 11.66472 | | 13.07549 | | 11.31222 | 1.276 up | | 3.394 up | | 2.658 up | unknown protein |
| 5270 | 0.00159 | 8.94152 | | 11.03537 | | 9.29333 | 1.276 down | | 3.345 up | | 4.268 up | unknown protein |
| 78725 | 0.000448 | 9.57563 | | 11.95947 | | 9.22418 | 1.275 up | | 6.658 up | | 5.219 up | unknown protein |
| 107282 | 0.00202 | 10.69237 | | 12.73718 | | 10.3412 | 1.275 up | | 5.263 up | | 4.126 up | unknown protein |
| 77829 | 0.00102 | 11.16747 | | 12.33639 | | 10.81638 | 1.275 up | | 2.867 up | | 2.248 up | unknown protein |
| 34611 | 0.00188 | 11.23672 | | 12.93656 | | 11.58728 | 1.275 down | | 2.547 up | | 3.248 up | unknown protein |
| 59112 | 0.000662 | 10.93765 | | 12.56715 | | 10.5876 | 1.274 up | | 3.943 up | | 3.094 up | unknown protein |
| 103186 | 0.00782 | 4.2594 | | 5.36158 | | 3.90997 | 1.274 up | | 2.735 up | | 2.146 up | unknown protein |
| 71019 | 0.000442 | 9.97624 | | 11.13488 | | 9.63025 | 1.271 up | | 2.837 up | | 2.232 up | unknown protein |
| 111689 | 0.00431 | 10.76011 | | 12.7698 | | 11.10514 | 1.270 down | | 3.170 up | | 4.026 up | unknown protein |
| 27132 | 0.00049 | 9.35903 | | 11.78588 | | 9.70227 | 1.268 down | | 4.238 up | | 5.377 up | unknown protein |
| 63702 | 0.0016 | 11.90065 | | 12.88754 | | 11.56144 | 1.265 up | | 2.507 up | | 1.981 up | unknown protein |
| 65483 | 0.00112 | 13.14062 | | 14.67902 | | 12.80158 | 1.264 up | | 3.674 up | | 2.904 up | unknown protein |
| 112086 | 0.00173 | 3.65054 | | 5.03614 | | 3.31228 | 1.264 up | | 3.303 up | | 2.612 up | unknown protein |
| 35137 | 0.0197 | 3.7686 | | 4.75511 | | 3.43104 | 1.263 up | | 2.503 up | | 1.981 up | unknown protein |
| 60756 | 0.000203 | 8.01993 | | 11.02438 | | 7.68341 | 1.262 up | | 10.132 up | | 8.024 up | unknown protein |
| 110600 | 0.00308 | 9.35679 | | 11.85624 | | 9.69247 | 1.261 down | | 4.480 up | | 5.654 up | unknown protein |
| 121239 | 0.000221 | 10.8793 | | 11.98493 | | 10.54598 | 1.259 up | | 2.711 up | | 2.151 up | unknown protein |
| 47069 | 0.000968 | 9.8497 | | 12.30394 | | 10.18202 | 1.259 down | | 4.352 up | | 5.480 up | unknown protein |
| 64777 | 0.000898 | 9.42525 | | 12.06519 | | 9.75715 | 1.258 down | | 4.952 up | | 6.233 up | unknown protein |
| 109729 | 0.00178 | 11.07968 | | 12.40487 | | 10.74954 | 1.257 up | | 3.149 up | | 2.505 up | unknown protein |
| 121639 | 0.00247 | 9.34796 | | 10.62239 | | 9.01753 | 1.257 up | | 3.041 up | | 2.419 up | unknown protein |
| 120451 | 0.00129 | 6.66585 | | 10.35422 | | 6.99584 | 1.257 down | | 10.255 up | | 12.891 up | unknown protein |
| 76200 | 0.000164 | 11.85452 | | 13.53954 | | 12.18397 | 1.256 down | | 2.558 up | | 3.215 up | unknown protein |
| 5607 | 0.000537 | 9.18711 | | 11.49432 | | 8.86056 | 1.254 up | | 6.206 up | | 4.949 up | unknown protein |
| 108702 | 0.00095 | 5.67457 | | 6.99835 | | 5.34752 | 1.254 up | | 3.140 up | | 2.503 up | unknown protein |
| 102467 | 0.00137 | 4.8032 | | 8.15313 | | 4.47706 | 1.253 up | | 12.782 up | | 10.196 up | unknown protein |
| 79501 | 0.00143 | 11.28698 | | 12.77266 | | 10.96268 | 1.252 up | | 3.506 up | | 2.800 up | unknown protein |
| 57335 | 0.00105 | 11.25339 | | 12.44981 | | 10.93107 | 1.250 up | | 2.865 up | | 2.291 up | unknown protein |
| 108951 | 0.000874 | 10.50933 | | 12.19254 | | 10.19498 | 1.243 up | | 3.993 up | | 3.211 up | unknown protein |
| 108566 | 0.000999 | 5.95017 | | 8.29918 | | 6.2645 | 1.243 down | | 4.097 up | | 5.094 up | unknown protein |
| 104071 | 0.000977 | 4.46762 | | 6.89125 | | 4.15547 | 1.241 up | | 6.661 up | | 5.365 up | unknown protein |
| 52446 | 0.017 | 11.58389 | | 12.86198 | | 11.27152 | 1.241 up | | 3.011 up | | 2.425 up | unknown protein |
| 57476 | 0.000828 | 10.52404 | | 13.3784 | | 10.83662 | 1.241 down | | 5.823 up | | 7.231 up | unknown protein |
| 124246 | 0.000875 | 9.39301 | | 11.10179 | | 9.0833 | 1.239 up | | 4.051 up | | 3.268 up | unknown protein |
| 123540 | 0.000867 | 11.34513 | | 13.08447 | | 11.65535 | 1.239 down | | 2.692 up | | 3.338 up | unknown protein |
| 60123 | 0.00478 | 10.69425 | | 12.0819 | | 10.38761 | 1.236 up | | 3.236 up | | 2.616 up | unknown protein |
| 30084 | 0.000514 | 7.89316 | | 11.06204 | | 7.58976 | 1.234 up | | 11.098 up | | 8.993 up | unknown protein |
| 71196 | 0.00133 | 5.1026 | | 6.5282 | | 4.80002 | 1.233 up | | 3.313 up | | 2.686 up | unknown protein |
| 77505 | 0.00225 | 10.89248 | | 12.00686 | | 10.58994 | 1.233 up | | 2.670 up | | 2.165 up | unknown protein |
| 76690 | 0.00139 | 10.20244 | | 12.46461 | | 10.50352 | 1.232 down | | 3.893 up | | 4.797 up | unknown protein |
| 52709 | 0.00057 | 11.65627 | | 12.69215 | | 11.35542 | 1.231 up | | 2.525 up | | 2.050 up | unknown protein |
| 122139 | 0.00249 | 11.51131 | | 13.6376 | | 11.81215 | 1.231 down | | 3.544 up | | 4.365 up | unknown protein |
| 66303 | 0.00253 | 8.60117 | | 10.8116 | | 8.89882 | 1.229 down | | 3.765 up | | 4.628 up | unknown protein |
| 60779 | 0.000994 | 9.66669 | | 11.03498 | | 9.36995 | 1.228 up | | 3.171 up | | 2.581 up | unknown protein |
| 109304 | 0.0135 | 3.86501 | | 5.08463 | | 3.56817 | 1.228 up | | 2.860 up | | 2.328 up | unknown protein |
| 39578 | 0.00078 | 8.89901 | | 10.69505 | | 8.60405 | 1.226 up | | 4.260 up | | 3.472 up | unknown protein |
| 120458 | 0.0113 | 8.72112 | | 11.38153 | | 9.01426 | 1.225 down | | 5.159 up | | 6.322 up | unknown protein |
| 69688 | 0.000472 | 3.07755 | | 5.8522 | | 3.36962 | 1.224 down | | 5.588 up | | 6.843 up | unknown protein |
| 123030 | 0.00035 | 10.09763 | | 12.65164 | | 10.38906 | 1.223 down | | 4.798 up | | 5.872 up | unknown protein |
| 107811 | 0.000605 | 4.88333 | | 7.07459 | | 4.59391 | 1.222 up | | 5.581 up | | 4.567 up | unknown protein |
| 104382 | 0.000844 | 9.60168 | | 10.82156 | | 9.31387 | 1.220 up | | 2.843 up | | 2.329 up | unknown protein |
| 69777 | 0.00123 | 7.40273 | | 10.24116 | | 7.69035 | 1.220 down | | 5.859 up | | 7.152 up | unknown protein |
| 41794 | 0.000326 | 9.45215 | | 11.84214 | | 9.73647 | 1.217 down | | 4.303 up | | 5.241 up | unknown protein |
| 104762 | 0.000128 | 7.57251 | | 11.25435 | | 7.85683 | 1.217 down | | 10.537 up | | 12.833 up | unknown protein |
| 109392 | 0.00132 | 5.33836 | | 7.93483 | | 5.61936 | 1.215 down | | 4.977 up | | 6.048 up | unknown protein |
| 112233 | 0.00417 | 10.34408 | | 12.12926 | | 10.62563 | 1.215 down | | 2.835 up | | 3.446 up | unknown protein |
| 77848 | 0.000374 | 9.28493 | | 11.04456 | | 9.56693 | 1.215 down | | 2.784 up | | 3.386 up | unknown protein |
| 105216 | 0.00447 | 5.56157 | | 7.50365 | | 5.28123 | 1.214 up | | 4.666 up | | 3.842 up | unknown protein |
| 105894 | 0.0181 | 5.39032 | | 7.07393 | | 5.66809 | 1.212 down | | 2.649 up | | 3.212 up | unknown protein |
| 61076 | 0.00168 | 10.01961 | | 11.62794 | | 9.7446 | 1.210 up | | 3.689 up | | 3.048 up | unknown protein |
| 106429 | 0.00612 | 7.24532 | | 8.40507 | | 6.96978 | 1.210 up | | 2.704 up | | 2.234 up | unknown protein |
| 5007 | 0.00187 | 7.38579 | | 10.32134 | | 7.11194 | 1.209 up | | 9.249 up | | 7.650 up | unknown protein |
| 105969 | 0.000253 | 8.0121 | | 10.20539 | | 7.74067 | 1.207 up | | 5.520 up | | 4.573 up | unknown protein |
| 22000 | 0.000332 | 10.26937 | | 12.62331 | | 10.54145 | 1.207 down | | 4.233 up | | 5.112 up | unknown protein |
| 79119 | 0.00551 | 11.02228 | | 13.01915 | | 11.29476 | 1.207 down | | 3.304 up | | 3.991 up | unknown protein |
| 35186 | 0.0025 | 7.04002 | | 8.26768 | | 6.77158 | 1.204 up | | 2.820 up | | 2.341 up | unknown protein |
| 63955 | 0.000882 | 9.69887 | | 12.07076 | | 9.43155 | 1.203 up | | 6.229 up | | 5.176 up | unknown protein |
| 68640 | 0.00159 | 7.37714 | | 8.74759 | | 7.11037 | 1.203 up | | 3.110 up | | 2.585 up | unknown protein |
| 121647 | 0.0017 | 11.47107 | | 13.17345 | | 11.7387 | 1.203 down | | 2.703 up | | 3.254 up | unknown protein |
| 23087 | 0.00171 | 8.69805 | | 10.09258 | | 8.43258 | 1.202 up | | 3.160 up | | 2.629 up | unknown protein |
| 110843 | 0.000347 | 6.57745 | | 8.36515 | | 6.31328 | 1.200 up | | 4.146 up | | 3.452 up | unknown protein |
| 5898 | 0.000515 | 10.45022 | | 12.45641 | | 10.71228 | 1.199 down | | 3.349 up | | 4.017 up | unknown protein |
| 105288 | 0.000402 | 8.28519 | | 10.23305 | | 8.02465 | 1.197 up | | 4.621 up | | 3.858 up | unknown protein |
| 67941 | 0.00183 | 8.17481 | | 10.84167 | | 8.43471 | 1.197 down | | 5.303 up | | 6.350 up | unknown protein |
| 122898 | 0.00391 | 11.00121 | | 12.58858 | | 11.26034 | 1.196 down | | 2.510 up | | 3.005 up | unknown protein |
| 112523 | 0.00157 | 6.37992 | | 7.53316 | | 6.12423 | 1.193 up | | 2.655 up | | 2.224 up | unknown protein |
| 6014 | 0.00066 | 9.3024 | | 11.07882 | | 9.55774 | 1.193 down | | 2.870 up | | 3.425 up | unknown protein |
| 105171 | 0.0168 | 3.65085 | | 5.2442 | | 3.39756 | 1.191 up | | 3.596 up | | 3.017 up | unknown protein |
| 103064 | 0.000527 | 8.77498 | | 10.9625 | | 9.02624 | 1.190 down | | 3.827 up | | 4.555 up | unknown protein |
| 65018 | 0.000267 | 10.10108 | | 11.71931 | | 9.85113 | 1.189 up | | 3.650 up | | 3.069 up | unknown protein |
| 77744 | 0.000774 | 11.42227 | | 13.21487 | | 11.17271 | 1.188 up | | 4.118 up | | 3.464 up | unknown protein |
| 54487 | 0.000264 | 10.83533 | | 12.29667 | | 10.58668 | 1.188 up | | 3.271 up | | 2.753 up | unknown protein |
| 121226 | 0.000498 | 10.37332 | | 13.44828 | | 10.62225 | 1.188 down | | 7.091 up | | 8.426 up | unknown protein |
| 109142 | 0.00227 | 9.26639 | | 11.37361 | | 9.51385 | 1.187 down | | 3.629 up | | 4.308 up | unknown protein |
| 106023 | 0.000721 | 11.08411 | | 12.31137 | | 10.83805 | 1.185 up | | 2.776 up | | 2.341 up | unknown protein |
| 120730 | 0.000447 | 9.95603 | | 12.60701 | | 10.19986 | 1.184 down | | 5.304 up | | 6.280 up | unknown protein |
| 60216 | 0.000747 | 12.52348 | | 13.69666 | | 12.28044 | 1.183 up | | 2.668 up | | 2.255 up | unknown protein |
| 109489 | 0.00251 | 3.87939 | | 5.44082 | | 3.64037 | 1.180 up | | 3.483 up | | 2.951 up | unknown protein |
| 111891 | 0.000211 | 5.46659 | | 9.72837 | | 5.22707 | 1.180 up | | 22.647 up | | 19.183 up | unknown protein |
| 107349 | 0.00299 | 3.43436 | | 4.67104 | | 3.19765 | 1.178 up | | 2.776 up | | 2.356 up | unknown protein |
| 33502 | 0.000657 | 9.81495 | | 11.57752 | | 9.57876 | 1.177 up | | 3.996 up | | 3.393 up | unknown protein |
| 21900 | 0.0015 | 11.86241 | | 13.20277 | | 11.62647 | 1.177 up | | 2.982 up | | 2.532 up | unknown protein |
| 78042 | 0.00191 | 11.54524 | | 12.88853 | | 11.31055 | 1.176 up | | 2.985 up | | 2.537 up | unknown protein |
| 112681 | 0.00732 | 6.14237 | | 7.26738 | | 5.9097 | 1.175 up | | 2.562 up | | 2.181 up | unknown protein |
| 4558 | 0.000181 | 9.06953 | | 12.19958 | | 9.30266 | 1.175 down | | 7.448 up | | 8.754 up | unknown protein |
| 53246 | 0.0106 | 10.52936 | | 12.28342 | | 10.29795 | 1.173 up | | 3.959 up | | 3.373 up | unknown protein |
| 104199 | 0.000497 | 10.89875 | | 12.52317 | | 10.66961 | 1.172 up | | 3.613 up | | 3.083 up | unknown protein |
| 75879 | 0.00113 | 9.9831 | | 11.83418 | | 10.21232 | 1.172 down | | 3.077 up | | 3.607 up | unknown protein |
| 77836 | 0.000372 | 9.58157 | | 10.9188 | | 9.35432 | 1.170 up | | 2.957 up | | 2.526 up | unknown protein |
| 122856 | 0.000502 | 11.45004 | | 13.06787 | | 11.67712 | 1.170 down | | 2.622 up | | 3.069 up | unknown protein |
| 65147 | 0.000295 | 8.06564 | | 10.30713 | | 7.84143 | 1.168 up | | 5.523 up | | 4.728 up | unknown protein |
| 107900 | 0.00205 | 7.77833 | | 9.28372 | | 7.55472 | 1.167 up | | 3.314 up | | 2.839 up | unknown protein |
| 80265 | 0.0104 | 9.90947 | | 11.91476 | | 10.13201 | 1.166 down | | 3.440 up | | 4.014 up | unknown protein |
| 4905 | 0.00256 | 6.27181 | | 8.81722 | | 6.05097 | 1.165 up | | 6.803 up | | 5.837 up | unknown protein |
| 67597 | 0.000121 | 4.8108 | | 12.5899 | | 4.58969 | 1.165 up | | 256.037 up | | 219.655 up | unknown protein |
| 82153 | 0.00219 | 10.396 | | 12.52097 | | 10.61635 | 1.165 down | | 3.744 up | | 4.361 up | unknown protein |
| 3787 | 0.00185 | 9.1406 | | 10.93017 | | 9.36156 | 1.165 down | | 2.966 up | | 3.457 up | unknown protein |
| 107151 | 0.000707 | 10.9326 | | 13.07781 | | 10.71232 | 1.164 up | | 5.153 up | | 4.423 up | unknown protein |
| 58115 | 0.000233 | 8.20491 | | 10.12547 | | 7.9865 | 1.163 up | | 4.404 up | | 3.785 up | unknown protein |
| 62676 | 0.00407 | 10.71435 | | 12.04577 | | 10.49649 | 1.163 up | | 2.926 up | | 2.516 up | unknown protein |
| 48295 | 0.00852 | 10.86204 | | 12.01106 | | 10.64401 | 1.163 up | | 2.579 up | | 2.217 up | unknown protein |
| 65671 | 0.000284 | 10.64933 | | 12.56612 | | 10.86746 | 1.163 down | | 3.245 up | | 3.775 up | unknown protein |
| 40996 | 0.00362 | 8.40551 | | 10.84281 | | 8.62241 | 1.162 down | | 4.660 up | | 5.416 up | unknown protein |
| 119815 | 0.00179 | 10.26977 | | 12.4577 | | 10.48638 | 1.161 down | | 3.921 up | | 4.556 up | unknown protein |
| 106471 | 0.00062 | 10.93105 | | 13.44634 | | 11.145 | 1.159 down | | 4.929 up | | 5.717 up | unknown protein |
| 29115 | 0.000339 | 9.55428 | | 11.44585 | | 9.34153 | 1.158 up | | 4.299 up | | 3.710 up | unknown protein |
| 120351 | 0.000692 | 9.69477 | | 12.0438 | | 9.90653 | 1.158 down | | 4.399 up | | 5.094 up | unknown protein |
| 123126 | 0.00309 | 9.11682 | | 11.10746 | | 9.32956 | 1.158 down | | 3.429 up | | 3.974 up | unknown protein |
| 53605 | 0.000266 | 9.88211 | | 11.79267 | | 10.09352 | 1.157 down | | 3.247 up | | 3.759 up | unknown protein |
| 78401 | 0.00341 | 10.85337 | | 12.80852 | | 11.06363 | 1.156 down | | 3.351 up | | 3.877 up | unknown protein |
| 77234 | 0.000801 | 9.24272 | | 11.44137 | | 9.03673 | 1.153 up | | 5.295 up | | 4.590 up | unknown protein |
| 54171 | 0.00027 | 9.32617 | | 11.03081 | | 9.52718 | 1.149 down | | 2.835 up | | 3.259 up | unknown protein |
| 64011 | 0.0011 | 10.44464 | | 11.93334 | | 10.24466 | 1.148 up | | 3.223 up | | 2.806 up | unknown protein |
| 106108 | 0.00191 | 10.8187 | | 12.23643 | | 10.61924 | 1.148 up | | 3.067 up | | 2.671 up | unknown protein |
| 119816 | 0.00116 | 11.19531 | | 13.20421 | | 11.39533 | 1.148 down | | 3.503 up | | 4.024 up | unknown protein |
| 123968 | 0.000973 | 12.69545 | | 14.32011 | | 12.89265 | 1.146 down | | 2.689 up | | 3.083 up | unknown protein |
| 105447 | 0.00111 | 4.99253 | | 6.89921 | | 4.79836 | 1.144 up | | 4.289 up | | 3.749 up | unknown protein |
| 120432 | 0.000212 | 11.40465 | | 13.04468 | | 11.59514 | 1.141 down | | 2.731 up | | 3.116 up | unknown protein |
| 107185 | 0.000566 | 5.90081 | | 8.4119 | | 5.71288 | 1.139 up | | 6.493 up | | 5.700 up | unknown protein |
| 54616 | 0.000499 | 10.15093 | | 12.23602 | | 10.33819 | 1.138 down | | 3.726 up | | 4.243 up | unknown protein |
| 70223 | 0.00376 | 8.66225 | | 11.24242 | | 8.84687 | 1.136 down | | 5.261 up | | 5.980 up | unknown protein |
| 108069 | 0.00166 | 10.0855 | | 12.52441 | | 10.27068 | 1.136 down | | 4.769 up | | 5.422 up | unknown protein |
| 74437 | 0.0109 | 9.08716 | | 10.90887 | | 8.90506 | 1.134 up | | 4.010 up | | 3.534 up | unknown protein |
| 61402 | 0.000359 | 11.29785 | | 12.54348 | | 11.11768 | 1.133 up | | 2.686 up | | 2.371 up | unknown protein |
| 31869 | 0.000943 | 10.42366 | | 12.93888 | | 10.60421 | 1.133 down | | 5.044 up | | 5.716 up | unknown protein |
| 123282 | 0.000808 | 8.44621 | | 10.8167 | | 8.26676 | 1.132 up | | 5.856 up | | 5.171 up | unknown protein |
| 103560 | 0.00131 | 7.46709 | | 9.51926 | | 7.28906 | 1.131 up | | 4.691 up | | 4.147 up | unknown protein |
| 61863 | 0.00173 | 10.58855 | | 12.01326 | | 10.40993 | 1.131 up | | 3.038 up | | 2.684 up | unknown protein |
| 119825 | 0.00127 | 3.61454 | | 5.80328 | | 3.79202 | 1.130 down | | 4.031 up | | 4.559 up | unknown protein |
| 109870 | 0.000491 | 10.26588 | | 12.11347 | | 10.44242 | 1.130 down | | 3.184 up | | 3.598 up | unknown protein |
| 40918 | 0.000322 | 11.24121 | | 12.85007 | | 11.41398 | 1.127 down | | 2.705 up | | 3.050 up | unknown protein |
| 112212 | 0.000342 | 10.17708 | | 12.78142 | | 10.00809 | 1.124 up | | 6.836 up | | 6.081 up | unknown protein |
| 68850 | 0.0000402 | 8.96982 | | 11.61132 | | 8.80214 | 1.123 up | | 7.008 up | | 6.239 up | unknown protein |
| 111186 | 0.00132 | 9.8567 | | 11.3961 | | 10.02524 | 1.123 down | | 2.586 up | | 2.906 up | unknown protein |
| 5916 | 0.00144 | 10.93729 | | 12.44435 | | 10.77009 | 1.122 up | | 3.191 up | | 2.842 up | unknown protein |
| 123256 | 0.0011 | 7.44255 | | 9.60543 | | 7.60975 | 1.122 down | | 3.988 up | | 4.478 up | unknown protein |
| 36855 | 0.00464 | 8.04817 | | 10.05362 | | 8.21474 | 1.122 down | | 3.577 up | | 4.015 up | unknown protein |
| 56070 | 0.000977 | 10.24008 | | 11.83206 | | 10.07511 | 1.121 up | | 3.379 up | | 3.014 up | unknown protein |
| 122889 | 0.00178 | 8.0132 | | 10.26548 | | 8.17725 | 1.120 down | | 4.252 up | | 4.764 up | unknown protein |
| 54462 | 0.00539 | 4.15332 | | 5.93434 | | 4.3172 | 1.120 down | | 3.067 up | | 3.436 up | unknown protein |
| 103812 | 0.00269 | 7.56589 | | 9.53562 | | 7.4024 | 1.119 up | | 4.386 up | | 3.916 up | unknown protein |
| 55519 | 0.00129 | 9.49915 | | 11.74743 | | 9.33794 | 1.118 up | | 5.312 up | | 4.751 up | unknown protein |
| 120420 | 0.00523 | 10.87159 | | 12.13757 | | 10.7095 | 1.118 up | | 2.690 up | | 2.404 up | unknown protein |
| 63717 | 0.000292 | 10.50346 | | 12.71774 | | 10.66481 | 1.118 down | | 4.149 up | | 4.640 up | unknown protein |
| 109889 | 0.00235 | 5.85161 | | 7.56358 | | 6.01312 | 1.118 down | | 2.929 up | | 3.276 up | unknown protein |
| 106570 | 0.000531 | 8.85245 | | 10.37807 | | 8.69202 | 1.117 up | | 3.217 up | | 2.879 up | unknown protein |
| 111923 | 0.000108 | 6.77044 | | 10.61267 | | 6.60967 | 1.117 up | | 16.033 up | | 14.342 up | unknown protein |
| 53334 | 0.00205 | 8.51294 | | 10.40902 | | 8.67288 | 1.117 down | | 3.331 up | | 3.722 up | unknown protein |
| 104230 | 0.000206 | 11.76016 | | 13.24889 | | 11.91962 | 1.116 down | | 2.512 up | | 2.806 up | unknown protein |
| 25018 | 0.000901 | 11.01886 | | 12.50927 | | 10.86146 | 1.115 up | | 3.133 up | | 2.809 up | unknown protein |
| 78095 | 0.00313 | 10.57866 | | 11.78029 | | 10.42663 | 1.111 up | | 2.555 up | | 2.299 up | unknown protein |
| 78463 | 0.0000865 | 7.98643 | | 10.82408 | | 8.13826 | 1.110 down | | 6.434 up | | 7.148 up | unknown protein |
| 4366 | 0.00111 | 8.04864 | | 9.60176 | | 7.89818 | 1.109 up | | 3.257 up | | 2.934 up | unknown protein |
| 72581 | 0.00356 | 8.82326 | | 10.25828 | | 8.67457 | 1.108 up | | 2.997 up | | 2.703 up | unknown protein |
| 107131 | 0.00128 | 9.63515 | | 11.29255 | | 9.78429 | 1.108 down | | 2.844 up | | 3.154 up | unknown protein |
| 81757 | 0.00644 | 11.51207 | | 13.06985 | | 11.66119 | 1.108 down | | 2.654 up | | 2.944 up | unknown protein |
| 119800 | 0.00105 | 11.17342 | | 12.67172 | | 11.31906 | 1.106 down | | 2.553 up | | 2.825 up | unknown protein |
| 120747 | 0.00174 | 8.48988 | | 10.36566 | | 8.34459 | 1.105 up | | 4.058 up | | 3.670 up | unknown protein |
| 107488 | 0.00722 | 3.67866 | | 6.5042 | | 3.53579 | 1.104 up | | 7.826 up | | 7.088 up | unknown protein |
| 53570 | 0.000806 | 6.16763 | | 8.48434 | | 6.0246 | 1.104 up | | 5.501 up | | 4.981 up | unknown protein |
| 108674 | 0.00254 | 9.26933 | | 11.18943 | | 9.12666 | 1.103 up | | 4.177 up | | 3.784 up | unknown protein |
| 108906 | 0.000338 | 9.7574 | | 11.6607 | | 9.89907 | 1.103 down | | 3.390 up | | 3.740 up | unknown protein |
| 108649 | 0.000254 | 11.17295 | | 13.3411 | | 11.03224 | 1.102 up | | 4.954 up | | 4.494 up | unknown protein |
| 112377 | 0.000933 | 8.37987 | | 10.21883 | | 8.51908 | 1.101 down | | 3.248 up | | 3.577 up | unknown protein |
| 80807 | 0.00502 | 10.21146 | | 11.73669 | | 10.07316 | 1.100 up | | 3.167 up | | 2.878 up | unknown protein |
| 32203 | 0.0000559 | 11.11883 | | 13.04806 | | 11.25637 | 1.100 down | | 3.462 up | | 3.808 up | unknown protein |
| 67540 | 0.00132 | 7.22045 | | 8.72202 | | 7.35799 | 1.100 down | | 2.574 up | | 2.831 up | unknown protein |
| 57696 | 0.00102 | 10.36438 | | 12.82671 | | 10.22787 | 1.099 up | | 6.058 up | | 5.511 up | unknown protein |
| 6011 | 0.000307 | 11.21896 | | 12.96465 | | 11.08229 | 1.099 up | | 3.686 up | | 3.353 up | unknown protein |
| 119633 | 0.00139 | 11.0103 | | 12.47904 | | 11.14681 | 1.099 down | | 2.517 up | | 2.767 up | unknown protein |
| 42027 | 0.00174 | 10.32998 | | 12.26718 | | 10.46578 | 1.098 down | | 3.485 up | | 3.829 up | unknown protein |
| 121178 | 0.00121 | 11.44914 | | 13.42454 | | 11.58393 | 1.097 down | | 3.581 up | | 3.932 up | unknown protein |
| 120985 | 0.000327 | 7.13585 | | 8.41319 | | 7.00341 | 1.096 up | | 2.656 up | | 2.423 up | unknown protein |
| 33482 | 0.000134 | 7.53899 | | 10.80975 | | 7.40606 | 1.096 up | | 10.583 up | | 9.651 up | unknown protein |
| 123441 | 0.00021 | 9.28382 | | 11.42432 | | 9.15192 | 1.095 up | | 4.831 up | | 4.409 up | unknown protein |
| 103900 | 0.00385 | 3.16071 | | 6.50389 | | 3.29283 | 1.095 down | | 9.260 up | | 10.148 up | unknown protein |
| 67024 | 0.000394 | 10.50447 | | 13.27546 | | 10.37592 | 1.093 up | | 7.461 up | | 6.825 up | unknown protein |
| 35240 | 0.0021 | 11.22504 | | 12.5103 | | 11.09589 | 1.093 up | | 2.665 up | | 2.437 up | unknown protein |
| 104422 | 0.000263 | 3.21869 | | 8.75875 | | 3.09126 | 1.092 up | | 50.825 up | | 46.528 up | unknown protein |
| 110386 | 0.000641 | 7.65819 | | 10.53565 | | 7.53252 | 1.091 up | | 8.017 up | | 7.348 up | unknown protein |
| 65102 | 0.0188 | 3.64994 | | 4.89738 | | 3.52328 | 1.091 up | | 2.592 up | | 2.374 up | unknown protein |
| 57274 | 0.00101 | 11.0991 | | 12.76733 | | 11.22235 | 1.089 down | | 2.917 up | | 3.178 up | unknown protein |
| 63837 | 0.00179 | 6.96703 | | 9.07847 | | 6.84983 | 1.084 up | | 4.686 up | | 4.321 up | unknown protein |
| 76659 | 0.000263 | 7.35495 | | 10.43199 | | 7.23948 | 1.083 up | | 9.141 up | | 8.438 up | unknown protein |
| 110096 | 0.00213 | 8.02508 | | 9.5805 | | 7.90962 | 1.083 up | | 3.184 up | | 2.939 up | unknown protein |
| 67607 | 0.00112 | 4.49011 | | 6.30556 | | 4.37509 | 1.082 up | | 3.811 up | | 3.519 up | unknown protein |
| 66776 | 0.0272 | 6.90052 | | 8.13757 | | 6.78663 | 1.082 up | | 2.550 up | | 2.357 up | unknown protein |
| 109801 | 0.0121 | 6.34346 | | 8.24559 | | 6.45791 | 1.082 down | | 3.452 up | | 3.737 up | unknown protein |
| 54902 | 0.00128 | 7.58859 | | 9.14209 | | 7.47497 | 1.081 up | | 3.175 up | | 2.935 up | unknown protein |
| 55036 | 0.00011 | 6.8873 | | 10.15021 | | 7.00071 | 1.081 down | | 8.873 up | | 9.599 up | unknown protein |
| 109946 | 0.00104 | 3.22167 | | 5.51141 | | 3.3349 | 1.081 down | | 4.520 up | | 4.889 up | unknown protein |
| 110088 | 0.00174 | 11.89824 | | 13.93068 | | 12.01143 | 1.081 down | | 3.782 up | | 4.090 up | unknown protein |
| 106494 | 0.000747 | 3.70408 | | 6.85671 | | 3.59418 | 1.079 up | | 9.596 up | | 8.892 up | unknown protein |
| 111561 | 0.000386 | 7.43981 | | 9.77197 | | 7.33003 | 1.079 up | | 5.433 up | | 5.035 up | unknown protein |
| 108517 | 0.000368 | 10.18748 | | 11.90514 | | 10.07909 | 1.078 up | | 3.545 up | | 3.289 up | unknown protein |
| 111326 | 0.000233 | 4.46324 | | 7.22307 | | 4.57066 | 1.077 down | | 6.287 up | | 6.773 up | unknown protein |
| 121289 | 0.000267 | 10.42675 | | 11.90854 | | 10.53474 | 1.077 down | | 2.591 up | | 2.792 up | unknown protein |
| 60557 | 0.00128 | 3.25599 | | 5.05917 | | 3.36056 | 1.075 down | | 3.245 up | | 3.489 up | unknown protein |
| 68717 | 0.0035 | 8.48558 | | 10.75702 | | 8.58616 | 1.072 down | | 4.502 up | | 4.828 up | unknown protein |
| 122102 | 0.000134 | 9.88894 | | 11.73741 | | 9.99056 | 1.072 down | | 3.356 up | | 3.601 up | unknown protein |
| 67738 | 0.00353 | 6.91407 | | 8.35207 | | 7.01485 | 1.072 down | | 2.526 up | | 2.709 up | unknown protein |
| 65533 | 0.0034 | 10.32028 | | 11.82041 | | 10.42 | 1.071 down | | 2.639 up | | 2.828 up | unknown protein |
| 109903 | 0.00153 | 10.26181 | | 12.15282 | | 10.16419 | 1.070 up | | 3.968 up | | 3.708 up | unknown protein |
| 103917 | 0.0041 | 9.36943 | | 11.29828 | | 9.46725 | 1.070 down | | 3.557 up | | 3.807 up | unknown protein |
| 40114 | 0.000883 | 11.23011 | | 13.00004 | | 11.1335 | 1.069 up | | 3.646 up | | 3.410 up | unknown protein |
| 106024 | 0.00164 | 7.72663 | | 9.47987 | | 7.62957 | 1.069 up | | 3.605 up | | 3.371 up | unknown protein |
| 57905 | 0.000351 | 8.70379 | | 10.95936 | | 8.8009 | 1.069 down | | 4.464 up | | 4.775 up | unknown protein |
| 81109 | 0.00173 | 10.74062 | | 12.59301 | | 10.64718 | 1.066 up | | 3.852 up | | 3.610 up | unknown protein |
| 21215 | 0.00113 | 10.62484 | | 12.72064 | | 10.71705 | 1.066 down | | 4.009 up | | 4.274 up | unknown protein |
| 104413 | 0.000181 | 10.42847 | | 12.65337 | | 10.33642 | 1.065 up | | 4.982 up | | 4.674 up | unknown protein |
| 121960 | 0.000245 | 9.79537 | | 11.67435 | | 9.70361 | 1.065 up | | 3.919 up | | 3.678 up | unknown protein |
| 76125 | 0.000278 | 8.22082 | | 10.47486 | | 8.13045 | 1.064 up | | 5.078 up | | 4.770 up | unknown protein |
| 64601 | 0.00341 | 10.18404 | | 11.48274 | | 10.09361 | 1.064 up | | 2.619 up | | 2.460 up | unknown protein |
| 62709 | 0.00453 | 10.55252 | | 12.00095 | | 10.6418 | 1.063 down | | 2.565 up | | 2.729 up | unknown protein |
| 120794 | 0.000285 | 10.56519 | | 12.00412 | | 10.65379 | 1.063 down | | 2.549 up | | 2.711 up | unknown protein |
| 123675 | 0.000148 | 8.39703 | | 11.64708 | | 8.48312 | 1.061 down | | 8.962 up | | 9.513 up | unknown protein |
| 55242 | 0.000177 | 9.5448 | | 11.65634 | | 9.4619 | 1.059 up | | 4.577 up | | 4.321 up | unknown protein |
| 104054 | 0.032 | 3.82363 | | 5.13159 | | 3.74167 | 1.058 up | | 2.620 up | | 2.475 up | unknown protein |
| 56942 | 0.000324 | 10.65974 | | 12.60165 | | 10.5795 | 1.057 up | | 4.061 up | | 3.842 up | unknown protein |
| 78448 | 0.00017 | 8.91596 | | 10.35738 | | 8.99656 | 1.057 down | | 2.568 up | | 2.715 up | unknown protein |
| 43770 | 0.00242 | 7.93844 | | 9.88492 | | 7.85857 | 1.056 up | | 4.073 up | | 3.854 up | unknown protein |
| 1737 | 0.00125 | 9.58336 | | 12.13561 | | 9.66129 | 1.055 down | | 5.557 up | | 5.865 up | unknown protein |
| 112458 | 0.000959 | 11.17043 | | 13.38548 | | 11.24817 | 1.055 down | | 4.399 up | | 4.642 up | unknown protein |
| 106018 | 0.00241 | 10.85915 | | 12.13154 | | 10.78266 | 1.054 up | | 2.547 up | | 2.415 up | unknown protein |
| 4422 | 0.00178 | 11.30337 | | 12.81399 | | 11.22836 | 1.053 up | | 3.001 up | | 2.849 up | unknown protein |
| 106467 | 0.00699 | 9.96651 | | 11.3146 | | 9.89147 | 1.053 up | | 2.681 up | | 2.545 up | unknown protein |
| 110692 | 0.000371 | 10.98944 | | 12.81233 | | 11.06437 | 1.053 down | | 3.358 up | | 3.537 up | unknown protein |
| 59838 | 0.000435 | 9.28315 | | 11.22507 | | 9.35516 | 1.051 down | | 3.655 up | | 3.842 up | unknown protein |
| 6067 | 0.00136 | 9.81146 | | 11.67051 | | 9.74101 | 1.050 up | | 3.809 up | | 3.627 up | unknown protein |
| 23146 | 0.0000495 | 11.72254 | | 13.57231 | | 11.793 | 1.050 down | | 3.432 up | | 3.604 up | unknown protein |
| 121185 | 0.000179 | 9.67164 | | 11.1742 | | 9.74235 | 1.050 down | | 2.697 up | | 2.833 up | unknown protein |
| 121399 | 0.00028 | 9.89924 | | 12.2786 | | 9.8293 | 1.049 up | | 5.461 up | | 5.203 up | unknown protein |
| 121786 | 0.00161 | 10.53018 | | 12.10978 | | 10.46065 | 1.049 up | | 3.136 up | | 2.988 up | unknown protein |
| 23173 | 0.000808 | 12.05504 | | 13.52438 | | 11.98518 | 1.049 up | | 2.906 up | | 2.768 up | unknown protein |
| 108712 | 0.000256 | 11.07055 | | 12.58872 | | 11.14016 | 1.049 down | | 2.729 up | | 2.864 up | unknown protein |
| 109319 | 0.0195 | 5.25814 | | 6.61625 | | 5.19021 | 1.048 up | | 2.687 up | | 2.563 up | unknown protein |
| 55629 | 0.00195 | 9.15147 | | 10.61764 | | 9.22036 | 1.048 down | | 2.634 up | | 2.762 up | unknown protein |
| 54089 | 0.00984 | 5.43197 | | 6.81878 | | 5.36542 | 1.047 up | | 2.738 up | | 2.614 up | unknown protein |
| 103898 | 0.000316 | 8.04207 | | 10.46672 | | 8.10763 | 1.046 down | | 5.130 up | | 5.368 up | unknown protein |
| 109437 | 0.000121 | 9.88423 | | 11.96202 | | 9.947 | 1.044 down | | 4.041 up | | 4.221 up | unknown protein |
| 120176 | 0.00382 | 10.69875 | | 12.45798 | | 10.63839 | 1.042 up | | 3.529 up | | 3.385 up | unknown protein |
| 110631 | 0.000486 | 8.60774 | | 10.16019 | | 8.66646 | 1.041 down | | 2.816 up | | 2.933 up | unknown protein |
| 76249 | 0.000258 | 11.45311 | | 13.25412 | | 11.51041 | 1.040 down | | 3.348 up | | 3.484 up | unknown protein |
| 108278 | 0.000552 | 9.48078 | | 11.50978 | | 9.53691 | 1.039 down | | 3.925 up | | 4.081 up | unknown protein |
| 61142 | 0.000219 | 6.837 | | 10.70288 | | 6.78248 | 1.038 up | | 15.141 up | | 14.579 up | unknown protein |
| 26019 | 0.00112 | 10.54762 | | 12.052 | | 10.60199 | 1.038 down | | 2.732 up | | 2.837 up | unknown protein |
| 62603 | 0.00273 | 8.98497 | | 10.84888 | | 9.03742 | 1.037 down | | 3.509 up | | 3.639 up | unknown protein |
| 4996 | 0.000614 | 9.23429 | | 11.78621 | | 9.18369 | 1.035 up | | 6.073 up | | 5.864 up | unknown protein |
| 78886 | 0.000735 | 8.78929 | | 11.11009 | | 8.73958 | 1.035 up | | 5.171 up | | 4.996 up | unknown protein |
| 109747 | 0.00436 | 10.06727 | | 11.83667 | | 10.11613 | 1.034 down | | 3.295 up | | 3.409 up | unknown protein |
| 107445 | 0.0000697 | 3.37287 | | 5.07465 | | 3.41893 | 1.032 down | | 3.150 up | | 3.253 up | unknown protein |
| 5446 | 0.00061 | 10.46326 | | 12.37486 | | 10.50855 | 1.031 down | | 3.645 up | | 3.762 up | unknown protein |
| 55443 | 0.000267 | 9.93003 | | 12.0027 | | 9.88608 | 1.030 up | | 4.336 up | | 4.206 up | unknown protein |
| 102973 | 0.000516 | 9.62483 | | 11.58505 | | 9.66822 | 1.030 down | | 3.775 up | | 3.891 up | unknown protein |
| 32263 | 0.000173 | 10.05047 | | 11.72031 | | 10.09424 | 1.030 down | | 3.086 up | | 3.181 up | unknown protein |
| 55039 | 0.000305 | 5.97177 | | 7.70957 | | 6.01196 | 1.028 down | | 3.243 up | | 3.335 up | unknown protein |
| 103949 | 0.00425 | 7.71401 | | 9.89359 | | 7.75342 | 1.027 down | | 4.408 up | | 4.530 up | unknown protein |
| 122304 | 0.000846 | 12.3706 | | 13.93759 | | 12.40999 | 1.027 down | | 2.883 up | | 2.962 up | unknown protein |
| 28787 | 0.000245 | 10.34126 | | 11.94688 | | 10.30292 | 1.026 up | | 3.125 up | | 3.043 up | unknown protein |
| 79426 | 0.000149 | 8.94646 | | 11.86187 | | 8.90988 | 1.025 up | | 7.738 up | | 7.544 up | unknown protein |
| 52521 | 0.000396 | 8.62679 | | 10.95843 | | 8.59116 | 1.025 up | | 5.159 up | | 5.033 up | unknown protein |
| 67108 | 0.000754 | 9.97407 | | 12.21762 | | 10.01035 | 1.025 down | | 4.618 up | | 4.735 up | unknown protein |
| 70861 | 0.000276 | 9.61541 | | 11.40135 | | 9.65194 | 1.025 down | | 3.362 up | | 3.448 up | unknown protein |
| 122255 | 0.00523 | 10.47779 | | 12.27755 | | 10.44345 | 1.024 up | | 3.565 up | | 3.481 up | unknown protein |
| 106362 | 0.00565 | 5.45538 | | 7.00374 | | 5.48974 | 1.024 down | | 2.856 up | | 2.924 up | unknown protein |
| 69423 | 0.000802 | 10.46139 | | 12.0693 | | 10.49085 | 1.020 down | | 2.986 up | | 3.048 up | unknown protein |
| 53858 | 0.00139 | 8.78907 | | 10.38157 | | 8.8179 | 1.020 down | | 2.956 up | | 3.015 up | unknown protein |
| 111981 | 0.000216 | 10.30324 | | 11.78402 | | 10.33241 | 1.020 down | | 2.735 up | | 2.790 up | unknown protein |
| 56341 | 0.00149 | 10.10607 | | 11.53678 | | 10.13573 | 1.020 down | | 2.640 up | | 2.695 up | unknown protein |
| 108025 | 0.000779 | 3.86798 | | 7.2409 | | 3.84132 | 1.018 up | | 10.553 up | | 10.359 up | unknown protein |
| 80725 | 0.000659 | 11.23256 | | 13.33323 | | 11.20836 | 1.016 up | | 4.361 up | | 4.289 up | unknown protein |
| 111202 | 0.0019 | 9.19591 | | 10.79596 | | 9.17176 | 1.016 up | | 3.082 up | | 3.031 up | unknown protein |
| 106152 | 0.000976 | 5.09361 | | 7.89294 | | 5.07077 | 1.015 up | | 7.072 up | | 6.961 up | unknown protein |
| 78476 | 0.00534 | 9.78101 | | 11.21289 | | 9.76041 | 1.014 up | | 2.736 up | | 2.697 up | unknown protein |
| 109096 | 0.00205 | 9.33754 | | 11.11962 | | 9.35776 | 1.014 down | | 3.391 up | | 3.439 up | unknown protein |
| 68208 | 0.00448 | 10.57776 | | 12.12136 | | 10.55853 | 1.013 up | | 2.954 up | | 2.915 up | unknown protein |
| 21129 | 0.000544 | 11.5301 | | 13.02157 | | 11.511 | 1.013 up | | 2.849 up | | 2.811 up | unknown protein |
| 123278 | 0.0124 | 4.55613 | | 6.2797 | | 4.53879 | 1.012 up | | 3.342 up | | 3.302 up | unknown protein |
| 122428 | 0.000395 | 10.12117 | | 12.2647 | | 10.10758 | 1.009 up | | 4.460 up | | 4.418 up | unknown protein |
| 62962 | 0.00425 | 10.21172 | | 11.6001 | | 10.19805 | 1.009 up | | 2.642 up | | 2.617 up | unknown protein |
| 122656 | 0.00225 | 9.98228 | | 11.76602 | | 9.96988 | 1.008 up | | 3.472 up | | 3.443 up | unknown protein |
| 81865 | 0.000529 | 10.89588 | | 12.35015 | | 10.88435 | 1.008 up | | 2.762 up | | 2.740 up | unknown protein |
| 28353 | 0.000977 | 10.69362 | | 12.64088 | | 10.68448 | 1.006 up | | 3.880 up | | 3.856 up | unknown protein |
| 104217 | 0.00209 | 8.28088 | | 10.11476 | | 8.28888 | 1.005 down | | 3.545 up | | 3.564 up | unknown protein |
| 106337 | 0.00134 | 8.48622 | | 10.60371 | | 8.49092 | 1.003 down | | 4.325 up | | 4.339 up | unknown protein |
| 22417 | 0.000444 | 10.65407 | | 12.16954 | | 10.65705 | 1.002 down | | 2.853 up | | 2.858 up | unknown protein |
| 122817 | 0.000537 | 10.17382 | | 12.91696 | | 10.17537 | 1.001 down | | 6.688 up | | 6.695 up | unknown protein |
| 59359 | 0.000403 | 11.95301 | | 13.28805 | | 11.95464 | 1.001 down | | 2.519 up | | 2.522 up | unknown protein |
| 35317 | 0.00789 | 11.09734 | | 12.49833 | | 11.09875 | 1.000 down | | 2.638 up | | 2.640 up | unknown protein |
| 68566 | 0.000668 | 11.98824 | | 12.19217 | | 9.15575 | 7.123 up | | 8.204 up | | 1.151 up | unknown protein |
| 79089 | 0.00027 | 11.28506 | | 13.09214 | | 10.92207 | 1.286 up | | 4.500 up | | 3.499 up | unknown protein |
| 69613 | 0.00588 | 6.92999 | | 6.79 | | 3.43335 | 11.287 up | | 10.243 up | | 1.101 down | unknown protein (Duf227) |
| 107780 | 0.00224 | 10.47612 | | 12.192 | | 10.25623 | 1.164 up | | 3.825 up | | 3.284 up | unknown protein containing a putative BTB/POZ domain |
| 23228 | 0.000175 | 12.74621 | | 14.91879 | | 13.18196 | 1.352 down | | 3.333 up | | 4.508 up | unknown protein Duf1479 |
| 62872 | 0.0000358 | 13.50017 | | 14.30659 | | 4.97546 | 368.293 up | | 644.095 up | | 1.748 up | unknown protein GPR1/FUN34/yaaH-like |
| 26151 | 0.000448 | 11.84271 | | 12.85704 | | 11.07724 | 1.699 up | | 3.433 up | | 2.019 up | unknown protein of Lalv9 family |
| 108894 | 0.000747 | 8.35251 | | 11.90949 | | 8.27804 | 1.052 up | | 12.392 up | | 11.769 up | unknown protein UPF0183 |
| 104593 | 0.00132 | 6.60147 | | 4.5661 | | 4.08942 | 5.704 up | | 1.391 up | | 4.099 down | unknown protein with ankyrin, Leu-zipper and WD40 |
| 112399 | 0.000197 | 9.24252 | | 10.32505 | | 8.20976 | 2.045 up | | 4.332 up | | 2.117 up | unknown protein with chromo domain |
| 1879 | 0.00571 | 11.37548 | | 13.51859 | | 11.89501 | 1.433 down | | 3.081 up | | 4.417 up | unknown protein with dDENN domain |
| 121417 | 0.00203 | 4.73804 | | 6.94185 | | 5.35871 | 1.537 down | | 2.996 up | | 4.606 up | unknown protein with fasciclin domain |
| 69131 | 0.0193 | 11.42283 | | 11.96892 | | 10.38835 | 2.048 up | | 2.990 up | | 1.460 up | Unknown protein with FYVE/PHD zinc finger domain. |
| 44764 | 0.000208 | 8.3801 | | 9.9177 | | 7.95389 | 1.343 up | | 3.900 up | | 2.903 up | Unknown protein with GRF zinc finger domain. |
| 64392 | 0.000621 | 10.09694 | | 12.37223 | | 10.27497 | 1.131 down | | 4.278 up | | 4.840 up | unknown protein with Kelch domain |
| 41001 | 0.000483 | 7.82673 | | 9.99255 | | 7.58164 | 1.185 up | | 5.318 up | | 4.487 up | unknown protein with Nif domain |
| 62765 | 0.00174 | 12.67206 | | 13.30388 | | 11.92127 | 1.682 up | | 2.607 up | | 1.549 up | unknown protein with NUDIX domain (hydrolase) |
| 120060 | 0.00132 | 12.37868 | | 13.80361 | | 11.41677 | 1.947 up | | 5.230 up | | 2.685 up | unknown protein with pleckstrin like domain |
| 108201 | 0.000933 | 12.53159 | | 12.20884 | | 9.66355 | 7.300 up | | 5.837 up | | 1.250 down | Unknown protein with reductase domain |
| 105432 | 0.00192 | 11.6402 | | 13.15504 | | 11.50647 | 1.097 up | | 3.135 up | | 2.857 up | unknown protein with RING finger SH3 domain |
| 55637 | 0.000651 | 9.83446 | | 11.37769 | | 8.54911 | 2.437 up | | 7.103 up | | 2.914 up | Unknown protein |
| 5275 | 0.000328 | 12.7541 | | 11.73314 | | 11.01229 | 3.344 up | | 1.648 up | | 2.029 down | unknown protein with SET domain |
| 37271 | 0.000391 | 11.14785 | | 13.28408 | | 11.75036 | 1.518 down | | 2.895 up | | 4.396 up | unknown protein with SH3 domain |
| 81097 | 0.000324 | 10.82954 | | 12.16922 | | 10.71158 | 1.085 up | | 2.746 up | | 2.530 up | unknown protein with TLC domain |
| 50299 | 0.000726 | 9.07472 | | 11.48237 | | 9.01902 | 1.039 up | | 5.514 up | | 5.306 up | unknown protein with TPR repeats |
| 58814 | 0.00338 | 4.93027 | | 7.66787 | | 3.75255 | 2.262 up | | 15.087 up | | 6.669 up | unknown protein with WD repeats |
| 65977 | 0.00135 | 9.98727 | | 12.1171 | | 10.37701 | 1.310 down | | 3.340 up | | 4.376 up | unknown protein with WD repeats |
| 70639 | 0.00445 | 8.58035 | | 9.741 | | 8.30361 | 1.211 up | | 2.708 up | | 2.235 up | unknown protein with WD repeats |
| 50707 | 0.00187 | 10.995 | | 13.07016 | | 11.07403 | 1.056 down | | 3.989 up | | 4.213 up | unknown protein with WD repeats |
| 74962 | 0.000681 | 11.39871 | | 10.92487 | | 9.57801 | 3.532 up | | 2.543 up | | 1.388 down | unknown protein with WD40 repeats |
| 62809 | 0.00273 | 10.30436 | | 11.72619 | | 9.97808 | 1.253 up | | 3.359 up | | 2.679 up | Unknown protein with WD40 repeats |
| 121011 | 0.00197 | 10.95582 | | 12.97984 | | 11.29638 | 1.266 down | | 3.211 up | | 4.067 up | Unknown protein with Zn-finger, FYVE type domain. |
| 80149 | 0.000935 | 14.85826 | | 14.4655 | | 12.46781 | 5.243 up | | 3.993 up | | 1.312 down | unknown protein, |
| 54352 | 0.000723 | 10.87852 | | 9.83931 | | 6.12039 | 27.060 up | | 13.167 up | | 2.055 down | unknown protein, |
| 66696 | 0.00143 | 8.01038 | | 4.93038 | | 5.20322 | 6.999 up | | 1.208 down | | 8.456 down | unknown protein, Duf636 |
| 120837 | 0.000186 | 13.14514 | | 13.75803 | | 11.42964 | 3.284 up | | 5.022 up | | 1.529 up | unknown protein, 1 TM domain |
| 110797 | 0.0000248 | 5.20926 | | 11.91675 | | 4.29443 | 1.885 up | | 197.037 up | | 104.510 up | unknown protein, 1 TM, only in Gibberella |
| 122108 | 0.000104 | 14.44909 | | 14.21154 | | 6.89569 | 187.845 up | | 159.326 up | | 1.178 down | unknown protein, 1TM, only in Gibberella and Chaetomium |
| 78645 | 0.00294 | 10.6158 | | 12.70905 | | 10.48098 | 1.097 up | | 4.685 up | | 4.267 up | unknown protein, 2 TM |
| 79828 | 0.000246 | 12.41158 | | 13.37777 | | 11.84787 | 1.478 up | | 2.887 up | | 1.953 up | unknown protein, 3 TM |
| 74580 | 0.000159 | 14.11674 | | 14.12416 | | 10.10484 | 16.132 up | | 16.215 up | | 1.005 up | unknown protein, 3 TM domains |
| 107844 | 0.000296 | 4.59635 | | 8.86151 | | 5.12085 | 1.438 down | | 13.367 up | | 19.228 up | unknown protein, 3 TM, only in Gibberella and Chaetomium |
| 106490 | 0.00239 | 4.38898 | | 7.63779 | | 4.30637 | 1.058 up | | 10.066 up | | 9.505 up | unknown protein, 3 TM, only in Neurospora and Chaetomium |
| 56546 | 0.0000867 | 10.43754 | | 12.73805 | | 7.05714 | 10.413 up | | 51.300 up | | 4.926 up | unknown protein, 3TM |
| 81122 | 0.000217 | 11.48148 | | 11.73894 | | 6.95838 | 22.992 up | | 27.484 up | | 1.195 up | unknown protein, 4 TM, only in Sordariomycetes |
| 122579 | 0.000204 | 9.39484 | | 12.6659 | | 7.53162 | 3.638 up | | 35.121 up | | 9.653 up | unknown protein, 4TM |
| 55950 | 0.000213 | 10.88819 | | 12.31136 | | 10.27824 | 1.526 up | | 4.092 up | | 2.681 up | unknown protein, 5 TM |
| 2071 | 0.00028 | 9.67752 | | 11.95265 | | 10.02701 | 1.274 down | | 3.799 up | | 4.840 up | unknown protein, 5 TM |
| 70500 | 0.000128 | 8.55704 | | 10.24645 | | 6.15096 | 5.300 up | | 17.094 up | | 3.225 up | unknown protein, 6TM |
| 123120 | 0.000568 | 10.84987 | | 12.00417 | | 6.64314 | 18.465 up | | 41.098 up | | 2.225 up | unknown protein, 8 TM |
| 4626 | 0.00218 | 13.27213 | | 12.0983 | | 11.92679 | 2.540 up | | 1.126 up | | 2.256 down | unknown protein, C2 domain |
| 109952 | 0.00457 | 3.84407 | | 6.02283 | | 4.62868 | 1.722 down | | 2.628 up | | 4.527 up | unknown protein, containing ankyrin repeats |
| 54511 | 0.000354 | 12.20574 | | 13.33289 | | 10.32644 | 3.678 up | | 8.035 up | | 2.184 up | unknown protein, contains BTB/POZ domain (=protein binding) |
| 111145 | 0.0186 | 4.98682 | | 4.08033 | | 3.39755 | 3.008 up | | 1.605 up | | 1.874 down | unknown protein, contains DEAD/DEAH box helicase, only in Hypocreaceae |
| 69537 | 0.00187 | 8.18314 | | 9.26928 | | 7.76552 | 1.335 up | | 2.835 up | | 2.123 up | unknown protein, contains F-box and WD repeat |
| 5359 | 0.00199 | 12.9139 | | 12.1042 | | 10.55266 | 5.138 up | | 2.931 up | | 1.752 down | unknown protein, Duf1348 |
| 59368 | 0.0000556 | 8.62714 | | 13.68945 | | 3.84452 | 27.524 up | | 919.645 up | | 33.412 up | unknown protein, Duf341 |
| 102562 | 0.00107 | 7.74783 | | 6.44402 | | 6.24581 | 2.832 up | | 1.147 up | | 2.468 down | unknown protein, Duf899 domain |
| 104200 | 0.000356 | 6.25752 | | 9.56644 | | 6.80115 | 1.457 down | | 6.798 up | | 9.910 up | unknown protein, F-box |
| 106043 | 0.000226 | 10.09125 | | 7.8767 | | 6.576 | 11.433 up | | 2.463 up | | 4.641 down | unknown protein, GFA-domain |
| 60810 | 0.000186 | 13.50693 | | 13.28325 | | 6.37743 | 140.020 up | | 119.911 up | | 1.167 down | unknown protein, GPR1/FUN34/yaaH protein, 6TMs |
| 70608 | 0.000221 | 12.02843 | | 6.74095 | | 5.10586 | 121.311 up | | 3.106 up | | 39.056 down | unknown protein, HHE domains |
| 80252 | 0.00072 | 9.70292 | | 11.36259 | | 7.45406 | 4.753 up | | 15.017 up | | 3.159 up | unknown protein, in Sordariomycetes |
| 110035 | 0.00078 | 4.35991 | | 8.90608 | | 6.61188 | 4.763 down | | 4.904 up | | 23.363 up | unknown protein, in Sordariomycetes |
| 48211 | 0.00487 | 13.38772 | | 12.78466 | | 11.84529 | 2.912 up | | 1.917 up | | 1.518 down | unknown protein, intracellular |
| 79345 | 0.000625 | 8.07118 | | 11.61662 | | 9.04444 | 1.963 down | | 5.947 up | | 11.675 up | unknown protein, Mpv17/PMP22 family |
| 32712 | 0.00028 | 13.61375 | | 14.14754 | | 11.46735 | 4.427 up | | 6.409 up | | 1.447 up | unknown protein, NACHT domain/ankyrin repeats |
| 107704 | 0.000113 | 9.42637 | | 13.73795 | | 10.09505 | 1.589 down | | 12.491 up | | 19.857 up | unknown protein, only in A. fumigatus |
| 112478 | 0.00164 | 6.50479 | | 9.23322 | | 6.99597 | 1.405 down | | 4.714 up | | 6.627 up | unknown protein, only in A. oryzae |
| 4526 | 0.000216 | 11.84867 | | 12.48903 | | 10.51193 | 2.525 up | | 3.936 up | | 1.558 up | unknown protein, only in ascomycota |
| 109716 | 0.000943 | 13.33118 | | 14.06668 | | 10.89573 | 5.409 up | | 9.006 up | | 1.664 up | unknown protein, only in ascomycota |
| 63413 | 0.000431 | 12.75864 | | 13.73628 | | 10.44826 | 4.960 up | | 9.767 up | | 1.969 up | unknown protein, only in ascomycota |
| 61798 | 0.000146 | 11.22522 | | 12.31127 | | 8.63681 | 6.014 up | | 12.767 up | | 2.122 up | unknown protein, only in ascomycota |
| 122971 | 0.00025 | 10.98731 | | 12.83376 | | 8.564 | 5.363 up | | 19.289 up | | 3.596 up | unknown protein, only in Chaetomium, A. oryzae, fumigatus and Magnaporthe |
| 46002 | 0.000274 | 14.15847 | | 13.59823 | | 10.97841 | 9.063 up | | 6.146 up | | 1.474 down | unknown protein, only in fungi |
| 107525 | 0.000183 | 9.36045 | | 10.10121 | | 7.5642 | 3.473 up | | 5.803 up | | 1.671 up | unknown protein, only in fungi |
| 53267 | 0.00201 | 9.81644 | | 11.30712 | | 8.35923 | 2.745 up | | 7.716 up | | 2.810 up | unknown protein, only in fungi |
| 3987 | 0.00115 | 9.83811 | | 12.38545 | | 10.99827 | 2.234 down | | 2.615 up | | 5.845 up | unknown protein, only in fungi |
| 78027 | 0.0014 | 10.42966 | | 11.61678 | | 10.14405 | 1.218 up | | 2.775 up | | 2.276 up | unknown protein, only in fungi |
| 106676 | 0.000397 | 7.83975 | | 8.46892 | | 5.71079 | 4.374 up | | 6.765 up | | 1.546 up | unknown protein, only in Gibberella |
| 71170 | 0.000491 | 9.64175 | | 13.57043 | | 10.13294 | 1.405 down | | 10.833 up | | 15.228 up | unknown protein, only in Gibberella |
| 123539 | 0.00412 | 11.70568 | | 12.20641 | | 10.42173 | 2.435 up | | 3.445 up | | 1.414 up | unknown protein, only in Gibberella and Arabidopsis |
| 59197 | 0.0105 | 7.9112 | | 8.12382 | | 6.6637 | 2.374 up | | 2.751 up | | 1.158 up | unknown protein, only in Gibberella and Chaetomium |
| 109275 | 0.000266 | 12.44126 | | 12.93916 | | 10.44578 | 3.987 up | | 5.630 up | | 1.412 up | unknown protein, only in Gibberella and Chaetomium |
| 119806 | 0.0016 | 12.18386 | | 13.30179 | | 11.807 | 1.298 up | | 2.818 up | | 2.170 up | unknown protein, only in Gibberella, Magnaporthe and Chaetomium |
| 68706 | 0.00103 | 13.00431 | | 13.49864 | | 11.61541 | 2.618 up | | 3.689 up | | 1.408 up | unknown protein, only in hypocreaceae |
| 103421 | 0.00666 | 10.86192 | | 11.8281 | | 9.70231 | 2.233 up | | 4.364 up | | 1.953 up | unknown protein, only in Hypocreaceae |
| 103920 | 0.000266 | 9.85225 | | 12.88186 | | 10.04511 | 1.143 down | | 7.144 up | | 8.165 up | unknown protein, only in Magnaporthe |
| 106294 | 0.00518 | 9.99563 | | 9.99954 | | 8.5973 | 2.635 up | | 2.643 up | | 1.002 up | unknown protein, only in Magnaporthe, Neurospora and Chaetomium |
| 105445 | 0.0285 | 2.81455 | | 4.87927 | | 3.41895 | 1.520 down | | 2.751 up | | 4.183 up | unknown protein, only in Neurospora and A. fumigatus |
| 49928 | 0.000558 | 8.73219 | | 11.7479 | | 9.29177 | 1.473 down | | 5.487 up | | 8.087 up | unknown protein, only in Sordariomycetes |
| 110418 | 0.0102 | 9.7976 | | 11.65693 | | 9.62607 | 1.126 up | | 4.086 up | | 3.628 up | unknown protein, only in Sordariomycetes |
| 76141 | 0.000741 | 12.10251 | | 12.97535 | | 9.12185 | 7.893 up | | 14.455 up | | 1.831 up | unknown protein, only present in ascomycota |
| 106129 | 0.00025 | 11.55211 | | 8.90083 | | 6.74431 | 28.008 up | | 4.458 up | | 6.282 down | unknown protein, only present in ascomycota |
| 76359 | 0.000458 | 7.74171 | | 10.99794 | | 9.65692 | 3.771 down | | 2.533 up | | 9.554 up | unknown protein, only present in ascomycota |
| 109848 | 0.00027 | 8.86942 | | 10.79537 | | 9.06343 | 1.143 down | | 3.321 up | | 3.799 up | unknown protein, only present in ascomycota |
| 44700 | 0.000627 | 12.0795 | | 12.95373 | | 9.27684 | 6.977 up | | 12.789 up | | 1.833 up | unknown protein, only present in ascomycota and Bacteriophage phBC6A51 |
| 119576 | 0.000637 | 10.68184 | | 11.57425 | | 8.63735 | 4.125 up | | 7.657 up | | 1.856 up | unknown protein, only present in ascomycota and Streptomyces |
| 111887 | 0.000544 | 9.56759 | | 11.56492 | | 4.81756 | 26.909 up | | 107.437 up | | 3.992 up | unknown protein, only present in fungi |
| 42972 | 0.000655 | 11.46869 | | 11.80332 | | 9.19026 | 4.851 up | | 6.117 up | | 1.261 up | unknown protein, only present in Gibberella and Magnaporthe |
| 64922 | 0.00056 | 11.74197 | | 13.58095 | | 11.56612 | 1.129 up | | 4.041 up | | 3.577 up | unknown protein, only present in Gibberella, Neurospora and Magnaporthe |
| 61642 | 0.000942 | 13.96081 | | 13.54725 | | 10.63159 | 10.050 up | | 7.545 up | | 1.331 down | unknown protein, secreted |
| 111138 | 0.000101 | 10.05611 | | 9.57545 | | 7.14462 | 7.523 up | | 5.392 up | | 1.395 down | unknown protein, secreted |
| 108642 | 0.00226 | 11.50935 | | 10.79184 | | 5.449 | 66.734 up | | 40.584 up | | 1.644 down | unknown protein, secreted |
| 4454 | 0.000503 | 12.56262 | | 11.61853 | | 9.68357 | 7.356 up | | 3.823 up | | 1.923 down | unknown protein, secreted |
| 106879 | 0.000312 | 9.96795 | | 13.29459 | | 7.97483 | 3.980 up | | 39.940 up | | 10.032 up | unknown protein, secreted |
| 108655 | 0.000126 | 5.91325 | | 9.33227 | | 3.14542 | 6.810 up | | 72.849 up | | 10.696 up | unknown protein, secreted |
| 55887 | 0.00012 | 6.50473 | | 12.3626 | | 6.84628 | 1.267 down | | 45.769 up | | 57.995 up | unknown protein, secreted |
| 105275 | 0.00424 | 7.42761 | | 10.22118 | | 7.96795 | 1.454 down | | 4.767 up | | 6.933 up | unknown protein, secreted, contains parallel beta-helix repeat |
| 40618 | 0.000229 | 9.31922 | | 12.7326 | | 9.89374 | 1.489 down | | 7.154 up | | 10.654 up | unknown protein, secreted, only in ascomycota |
| 108676 | 0.0344 | 6.73313 | | 4.88764 | | 5.22082 | 2.852 up | | 1.259 down | | 3.593 down | unknown protein, secreted, only in ascomycota and Acidothermicus cellulolyticus |
| 108233 | 0.00822 | 7.69495 | | 8.46028 | | 6.87924 | 1.760 up | | 2.991 up | | 1.699 up | unknown protein, secreted, only in fungi |
| 122095 | 0.000136 | 12.45873 | | 13.74386 | | 11.54533 | 1.883 up | | 4.590 up | | 2.437 up | unknown protein, secreted, only present in ascomycota |
| 124283 | 0.000197 | 8.91817 | | 13.48594 | | 9.26476 | 1.271 down | | 18.650 up | | 23.715 up | unknown protein, SET and MYND domains |
| 70991 | 0.000141 | 5.83002 | | 8.85258 | | 5.00987 | 1.765 up | | 14.347 up | | 8.126 up | unknown protein, ThiJ/PfpI domain |
| 108721 | 0.000336 | 3.1056 | | 7.88473 | | 4.46803 | 2.571 down | | 10.678 up | | 27.457 up | unknown protein, unique in fungi |
| 122590 | 0.000596 | 12.65296 | | 13.10127 | | 11.17756 | 2.780 up | | 3.793 up | | 1.364 up | unknown protein, unique in fungi, 1TM |
| 67642 | 0.000378 | 10.79639 | | 12.1566 | | 8.07671 | 6.587 up | | 16.910 up | | 2.567 up | unknown protein, UPF0075 |
| 119981 | 0.000254 | 13.86554 | | 13.90247 | | 12.4949 | 2.585 up | | 2.652 up | | 1.025 up | unknown protein, vacuolar membrane |
| 47971 | 0.00127 | 10.22546 | | 12.82814 | | 10.56368 | 1.264 down | | 4.804 up | | 6.074 up | unknown protein, WD domains |
| 26994 | 0.00193 | 10.48719 | | 11.77284 | | 10.14484 | 1.267 up | | 3.090 up | | 2.437 up | unknown protein, with F-box/WD domains |
| 122527 | 0.000292 | 11.23961 | | 10.82003 | | 7.60036 | 12.460 up | | 9.315 up | | 1.337 down | unknown proteinwith Duf718 domain |
| 53824 | 0.000134 | 11.12239 | | 8.11237 | | 6.17625 | 30.827 up | | 3.826 up | | 8.055 down | unknown secreted protein |
| 43430 | 0.00265 | 13.01166 | | 13.72612 | | 12.38705 | 1.541 up | | 2.529 up | | 1.640 up | unknown secreted protein |
| 67902 | 0.00287 | 11.31726 | | 13.26782 | | 11.40751 | 1.064 down | | 3.630 up | | 3.865 up | unknown secreted protein |
| 5016 | 0.000133 | 12.03884 | | 13.85246 | | 10.46243 | 2.982 up | | 10.483 up | | 3.515 up | unknown secreted protein, only in Gibberella, Magnaporthe, Chaetomium and rats (!) |
| 68997 | 0.000917 | 6.61232 | | 10.37376 | | 8.78566 | 4.510 down | | 3.006 up | | 13.561 up | unknown TPR domain protein, unknown |
| 102673 | 0.000364 | 10.8956 | | 12.11985 | | 10.29563 | 1.515 up | | 3.541 up | | 2.336 up | unknwon zinc finger protein |
| 76009 | 0.00385 | 10.76444 | | 11.29615 | | 9.85289 | 1.881 up | | 2.719 up | | 1.445 up | unknwon zinc finger protein |
| 120983 | 0.00823 | 12.86176 | | 13.21845 | | 11.39959 | 2.755 up | | 3.528 up | | 1.280 up | uracil phosphoribosyl transferase |
| 3462 | 0.00453 | 12.10767 | | 10.52295 | | 10.78049 | 2.509 up | | 1.195 down | | 2.999 down | UreF urease accessory protein |
| 68527 | 0.000269 | 10.23246 | | 12.2463 | | 9.96422 | 1.204 up | | 4.863 up | | 4.038 up | UVSD; probable regulator of DNA damage response |
| 36373 | 0.000322 | 6.68792 | | 9.47751 | | 7.98065 | 2.449 down | | 2.822 up | | 6.914 up | Vacuolar assembly/sorting protein VPS9 |
| 72086 | 0.000838 | 6.16341 | | 8.96453 | | 3.48786 | 6.388 up | | 44.528 up | | 6.969 up | Vacuolar carboxypeptidase Cps1 |
| 112519 | 0.000357 | 10.33189 | | 12.19646 | | 9.5513 | 1.717 up | | 6.255 up | | 3.641 up | vacuolar endopolyphosphatase |
| 62477 | 0.000585 | 8.95842 | | 11.19084 | | 9.70192 | 1.674 down | | 2.806 up | | 4.699 up | Vacuolar protein sorting-associated protein Vps28 |
| 65200 | 0.000995 | 10.35705 | | 11.73936 | | 10.08395 | 1.208 up | | 3.150 up | | 2.606 up | Vacuolar protein sorting-associated protein Vsp60 |
| 121495 | 0.00329 | 4.54956 | | 6.94868 | | 3.81266 | 1.666 up | | 8.790 up | | 5.274 up | Vacuolar proteinase B (yscB), a serine protease of the subtilisin family |
| 61605 | 0.00181 | 5.91722 | | 8.92645 | | 6.1499 | 1.175 down | | 6.852 up | | 8.051 up | Vacuolar sorting protein VPS1, dynamin, and related proteins |
| 108706 | 0.000379 | 10.71443 | | 12.13171 | | 10.29709 | 1.335 up | | 3.566 up | | 2.670 up | Vacuolar sorting protien/ubiquitin receptor Vps23 |
| 65821 | 0.00337 | 11.90972 | | 13.23869 | | 11.73871 | 1.125 up | | 2.828 up | | 2.512 up | vacuolar targeting protein Atg18 |
| 58638 | 0.0184 | 9.73352 | | 11.14732 | | 9.56107 | 1.126 up | | 3.002 up | | 2.664 up | vacuolar-sorting protein SNF8, putative |
| 65880 | 0.00161 | 11.62793 | | 12.70509 | | 10.56684 | 2.086 up | | 4.402 up | | 2.109 up | voltage gated chloride channel |
| 102737 | 0.00252 | 9.91356 | | 11.37844 | | 8.64991 | 2.401 up | | 6.627 up | | 2.760 up | VosA ? |
| 58990 | 0.00381 | 8.93573 | | 10.77045 | | 9.11696 | 1.133 down | | 3.145 up | | 3.567 up | v-SNARE Bos1, ER-Golgi |
| 45476 | 0.0148 | 12.77457 | | 13.48262 | | 12.11158 | 1.583 up | | 2.586 up | | 1.633 up | v-Snare Sft1; intra-Golgi |
| 2241 | 0.0163 | 12.42586 | | 13.31166 | | 11.97825 | 1.363 up | | 2.519 up | | 1.847 up | WASP-like pretein las17p |
| 4913 | 0.000801 | 12.1622 | | 12.77741 | | 10.87957 | 2.432 up | | 3.726 up | | 1.531 up | WD repeat domain-containing phosphoinositide-interacting protein |
| 81949 | 0.00303 | 12.74473 | | 13.30463 | | 11.74968 | 1.993 up | | 2.938 up | | 1.474 up | Winged helix repressor DNA-binding |
| 78797 | 0.00244 | 12.89125 | | 13.23471 | | 11.00468 | 3.697 up | | 4.691 up | | 1.268 up | xanthine dehydrogenase |
| 81271 | 0.000405 | 10.45855 | | 12.51089 | | 9.66497 | 1.733 up | | 7.189 up | | 4.147 up | xylitol dehydrogenase XDH1 |
| 107776 | 0.000482 | 11.39178 | | 13.50924 | | 9.66075 | 3.319 up | | 14.404 up | | 4.339 up | xylose reductase |
| 104072 | 0.000163 | 9.89514 | | 13.35834 | | 5.41849 | 22.264 up | | 245.546 up | | 11.028 up | xylose transporter |
| 44965 | 0.00301 | 12.8514 | | 11.16712 | | 9.95713 | 7.434 up | | 2.313 up | | 3.213 down | zinc binding oxidoreductase |
| 22210 | 0.000133 | 10.5382 | | 13.21128 | | 7.8445 | 6.469 up | | 41.263 up | | 6.377 up | Zinc carboxypeptidase |
| 71496 | 0.00127 | 5.03966 | | 7.93787 | | 5.71364 | 1.595 down | | 4.672 up | | 7.454 up | Zinc-binding dehydrogenase |
| 108920 | 0.00423 | 11.89009 | | 11.41878 | | 10.27804 | 3.056 up | | 2.204 up | | 1.386 down | zinc-binding dehydrogenase |
| 59322 | 0.000133 | 9.45163 | | 7.34903 | | 6.07983 | 10.351 up | | 2.410 up | | 4.294 down | Zinc-binding oxidoreductase |
| 53526 | 0.000408 | 7.86657 | | 8.28115 | | 5.59299 | 4.835 up | | 6.444 up | | 1.332 up | Zinc-containing alcohol dehydrogenase |
| 56839 | 0.000175 | 8.58022 | | 10.16269 | | 7.3215 | 2.392 up | | 7.166 up | | 2.994 up | Zinc-containing alcohol dehydrogenase |
| 66827 | 0.023 | 9.66211 | | 9.01617 | | 7.95289 | 3.269 up | | 2.089 up | | 1.564 down | Zinc-containing alcohol dehydrogenase |
| 108985 | 0.000472 | 6.86975 | | 9.53406 | | 6.21633 | 1.572 up | | 9.970 up | | 6.339 up | Zinc-containing alcohol dehydrogenase |
| 56326 | 0.00109 | 6.23267 | | 7.44231 | | 5.76503 | 1.382 up | | 3.198 up | | 2.312 up | Zinc-containing alcohol dehydrogenase |
| 53343 | 0.00113 | 12.02383 | | 13.02686 | | 11.08593 | 1.915 up | | 3.839 up | | 2.004 up | Zinc-dependent metalloprotease, ADAM_fungal subgroup |
| 47987 | 0.00146 | 10.79473 | | 12.41811 | | 10.88663 | 1.065 down | | 2.890 up | | 3.080 up | ZIP Zinc transporter |
| 107479 | 0.000147 | 8.29651 | | 13.0327 | | 10.35239 | 4.157 down | | 6.409 up | | 26.652 up | ZIP zinc/iron transporter |
| 58634 | 0.0043 | 11.63536 | | 11.31506 | | 10.05693 | 2.986 up | | 2.391 up | | 1.248 down | Zn2Cys6 Fungal transcriptional regulator |
| 122271 | 0.000256 | 12.24659 | | 14.0743 | | 12.6591 | 1.330 down | | 2.666 up | | 3.549 up | Zn2Cys6 transcription regulator, C. albicans Fcr1 |
| 105239 | 0.0022 | 11.55589 | | 11.54779 | | 9.5766 | 3.942 up | | 3.920 up | | 1.005 down | Zn2Cys6 transcriptional regulator |
| 52875 | 0.00351 | 12.72333 | | 12.7515 | | 11.24958 | 2.777 up | | 2.832 up | | 1.019 up | Zn2Cys6 transcriptional regulator |
| 70414 | 0.0024 | 9.67928 | | 9.64626 | | 8.15041 | 2.885 up | | 2.820 up | | 1.023 down | Zn2Cys6 transcriptional regulator |
| 61476 | 0.000689 | 9.72729 | | 9.68947 | | 6.92571 | 6.972 up | | 6.791 up | | 1.026 down | Zn2Cys6 transcriptional regulator |
| 106654 | 0.00226 | 8.92844 | | 8.88974 | | 7.06056 | 3.649 up | | 3.553 up | | 1.027 down | Zn2Cys6 transcriptional regulator |
| 79725 | 0.0018 | 10.00449 | | 10.07568 | | 8.47864 | 2.879 up | | 3.025 up | | 1.050 up | Zn2Cys6 transcriptional regulator |
| 65854 | 0.000828 | 10.86707 | | 10.77139 | | 3.66964 | 146.771 up | | 137.353 up | | 1.068 down | Zn2Cys6 transcriptional regulator |
| 105263 | 0.00389 | 11.69007 | | 11.59045 | | 10.19391 | 2.820 up | | 2.632 up | | 1.071 down | Zn2Cys6 transcriptional regulator |
| 123509 | 0.00157 | 9.77373 | | 9.90475 | | 7.69123 | 4.235 up | | 4.638 up | | 1.095 up | Zn2Cys6 transcriptional regulator |
| 47479 | 0.000476 | 11.08248 | | 10.92695 | | 8.85299 | 4.689 up | | 4.210 up | | 1.113 down | Zn2Cys6 transcriptional regulator |
| 112539 | 0.00127 | 6.70192 | | 6.51668 | | 5.03294 | 3.179 up | | 2.796 up | | 1.137 down | Zn2Cys6 transcriptional regulator |
| 55105 | 0.000133 | 9.03661 | | 8.82398 | | 5.46604 | 11.880 up | | 10.252 up | | 1.158 down | Zn2Cys6 transcriptional regulator |
| 70570 | 0.000676 | 9.58595 | | 9.85443 | | 7.7955 | 3.459 up | | 4.166 up | | 1.204 up | Zn2Cys6 transcriptional regulator |
| 122783 | 0.000505 | 12.47611 | | 12.7451 | | 11.22665 | 2.377 up | | 2.864 up | | 1.204 up | Zn2Cys6 transcriptional regulator |
| 60282 | 0.0000523 | 12.04127 | | 12.3312 | | 8.96051 | 8.460 up | | 10.343 up | | 1.222 up | Zn2Cys6 transcriptional regulator |
| 123717 | 0.00137 | 11.35172 | | 11.66973 | | 10.09829 | 2.384 up | | 2.971 up | | 1.246 up | Zn2Cys6 transcriptional regulator |
| 112401 | 0.00359 | 11.01065 | | 11.37361 | | 9.31606 | 3.236 up | | 4.162 up | | 1.286 up | Zn2Cys6 transcriptional regulator |
| 111446 | 0.000812 | 12.13425 | | 11.73491 | | 8.50888 | 12.340 up | | 9.356 up | | 1.318 down | Zn2Cys6 transcriptional regulator |
| 71080 | 0.00136 | 9.415 | | 9.00906 | | 7.26244 | 4.446 up | | 3.355 up | | 1.324 down | Zn2Cys6 transcriptional regulator |
| 119620 | 0.0024 | 11.0414 | | 11.45952 | | 10.00467 | 2.051 up | | 2.741 up | | 1.336 up | Zn2Cys6 transcriptional regulator |
| 105980 | 0.000246 | 11.20409 | | 11.64152 | | 9.05768 | 4.427 up | | 5.995 up | | 1.354 up | Zn2Cys6 transcriptional regulator |
| 36703 | 0.000974 | 10.56245 | | 11.12403 | | 9.28387 | 2.425 up | | 3.580 up | | 1.475 up | Zn2Cys6 transcriptional regulator |
| 36468 | 0.000804 | 11.21395 | | 10.6266 | | 8.91139 | 4.933 up | | 3.283 up | | 1.502 down | Zn2Cys6 transcriptional regulator |
| 69972 | 0.000352 | 11.65825 | | 12.26592 | | 9.74194 | 3.774 up | | 5.751 up | | 1.523 up | Zn2Cys6 transcriptional regulator |
| 60931 | 0.00204 | 8.79107 | | 9.40293 | | 7.10235 | 3.223 up | | 4.926 up | | 1.528 up | Zn2Cys6 transcriptional regulator |
| 120228 | 0.0000622 | 10.89373 | | 11.5101 | | 6.6849 | 18.492 up | | 28.348 up | | 1.533 up | Zn2Cys6 transcriptional regulator |
| 72993 | 0.0022 | 6.32835 | | 6.94859 | | 5.25754 | 2.100 up | | 3.228 up | | 1.537 up | Zn2Cys6 transcriptional regulator |
| 120715 | 0.00157 | 11.32827 | | 11.95088 | | 10.25421 | 2.105 up | | 3.241 up | | 1.539 up | Zn2Cys6 transcriptional regulator |
| 52368 | 0.000201 | 12.73381 | | 13.48874 | | 11.14778 | 3.002 up | | 5.066 up | | 1.687 up | Zn2Cys6 transcriptional regulator |
| 65746 | 0.00055 | 11.34687 | | 12.21085 | | 10.16357 | 2.270 up | | 4.133 up | | 1.820 up | Zn2Cys6 transcriptional regulator |
| 58389 | 0.000424 | 11.44843 | | 12.33136 | | 10.22587 | 2.333 up | | 4.303 up | | 1.844 up | Zn2Cys6 transcriptional regulator |
| 104104 | 0.000531 | 9.22364 | | 10.13552 | | 7.45209 | 3.414 up | | 6.423 up | | 1.881 up | Zn2Cys6 transcriptional regulator |
| 49232 | 0.00075 | 12.71594 | | 13.62944 | | 11.52073 | 2.289 up | | 4.313 up | | 1.883 up | Zn2Cys6 transcriptional regulator |
| 112524 | 0.000206 | 11.16468 | | 12.13078 | | 10.13696 | 2.038 up | | 3.982 up | | 1.953 up | Zn2Cys6 transcriptional regulator |
| 121107 | 0.000798 | 11.65555 | | 12.62789 | | 10.59351 | 2.087 up | | 4.096 up | | 1.962 up | Zn2Cys6 transcriptional regulator |
| 60578 | 0.000372 | 11.7064 | | 12.69852 | | 9.91407 | 3.463 up | | 6.889 up | | 1.989 up | Zn2Cys6 transcriptional regulator |
| 111088 | 0.000544 | 7.46437 | | 8.46229 | | 6.44091 | 2.032 up | | 4.059 up | | 1.997 up | Zn2Cys6 transcriptional regulator |
| 62622 | 0.000742 | 9.11977 | | 10.1243 | | 7.03155 | 4.252 up | | 8.531 up | | 2.006 up | Zn2Cys6 transcriptional regulator |
| 59353 | 0.00048 | 9.09581 | | 10.11944 | | 7.99247 | 2.148 up | | 4.367 up | | 2.033 up | Zn2Cys6 transcriptional regulator |
| 124260 | 0.000306 | 9.65078 | | 10.71267 | | 7.80529 | 3.593 up | | 7.502 up | | 2.087 up | Zn2Cys6 transcriptional regulator |
| 105834 | 0.000531 | 8.99989 | | 10.1226 | | 7.56837 | 2.697 up | | 5.873 up | | 2.177 up | Zn2Cys6 transcriptional regulator |
| 104380 | 0.00154 | 8.36221 | | 7.21645 | | 5.38186 | 7.891 up | | 3.566 up | | 2.212 down | Zn2Cys6 transcriptional regulator |
| 53067 | 0.000178 | 10.05288 | | 11.19989 | | 8.5934 | 2.750 up | | 6.090 up | | 2.214 up | Zn2Cys6 transcriptional regulator |
| 62244 | 0.00105 | 10.92218 | | 12.07991 | | 9.29196 | 3.095 up | | 6.906 up | | 2.231 up | Zn2Cys6 transcriptional regulator |
| 80139 | 0.00105 | 10.28201 | | 11.47973 | | 8.40883 | 3.663 up | | 8.402 up | | 2.293 up | Zn2Cys6 transcriptional regulator |
| 54502 | 0.000275 | 10.81259 | | 12.04943 | | 8.13485 | 6.398 up | | 15.080 up | | 2.356 up | Zn2Cys6 transcriptional regulator |
| 72611 | 0.000164 | 13.77497 | | 12.4992 | | 8.34019 | 43.254 up | | 17.864 up | | 2.421 down | Zn2Cys6 transcriptional regulator |
| 69695 | 0.000273 | 10.00053 | | 8.54261 | | 6.46902 | 11.563 up | | 4.209 up | | 2.747 down | Zn2Cys6 transcriptional regulator |
| 112134 | 0.000977 | 9.68981 | | 8.11445 | | 5.54851 | 17.646 up | | 5.921 up | | 2.980 down | Zn2Cys6 transcriptional regulator |
| 107858 | 0.000944 | 7.85758 | | 9.19807 | | 5.90289 | 3.876 up | | 9.816 up | | 2.532 up | Zn2Cys6 transcriptional regulator |
| 26255 | 0.00107 | 8.74336 | | 10.13311 | | 7.73374 | 2.013 up | | 5.275 up | | 2.620 up | Zn2Cys6 transcriptional regulator |
| 77513 | 0.0000874 | 11.59059 | | 13.1548 | | 10.01774 | 2.974 up | | 8.797 up | | 2.957 up | Zn2Cys6 transcriptional regulator |
| 121138 | 0.00016 | 6.20847 | | 10.59988 | | 3.65778 | 5.859 up | | 122.964 up | | 20.986 up | Zn2Cys6 transcriptional regulator |
| 36913 | 0.00183 | 7.60669 | | 9.21392 | | 6.30687 | 2.461 up | | 7.500 up | | 3.046 up | Zn2Cys6 transcriptional regulator |
| 105269 | 0.000217 | 9.61019 | | 11.2194 | | 4.13339 | 44.533 up | | 135.862 up | | 3.050 up | Zn2Cys6 transcriptional regulator |
| 27600 | 0.00025 | 10.99426 | | 12.61674 | | 8.87203 | 4.353 up | | 13.405 up | | 3.079 up | Zn2Cys6 transcriptional regulator |
| 103751 | 0.000303 | 9.63055 | | 11.27348 | | 8.3691 | 2.397 up | | 7.486 up | | 3.123 up | Zn2Cys6 transcriptional regulator |
| 70351 | 0.000104 | 10.42174 | | 12.1371 | | 8.51586 | 3.747 up | | 12.305 up | | 3.283 up | Zn2Cys6 transcriptional regulator |
| 55274 | 0.00168 | 9.38757 | | 11.34301 | | 7.60234 | 3.446 up | | 13.367 up | | 3.878 up | Zn2Cys6 transcriptional regulator |
| 120988 | 0.000354 | 10.44608 | | 12.60446 | | 9.34489 | 2.145 up | | 9.576 up | | 4.464 up | Zn2Cys6 transcriptional regulator |
| 121415 | 0.0000248 | 8.46805 | | 14.15052 | | 4.21591 | 19.055 up | | 978.625 up | | 51.356 up | Zn2Cys6 transcriptional regulator |
| 72076 | 0.0009 | 8.80585 | | 11.42626 | | 7.70701 | 2.141 up | | 13.170 up | | 6.149 up | Zn2Cys6 transcriptional regulator |
| 121474 | 0.000515 | 12.36048 | | 11.246 | | 9.99148 | 5.165 up | | 2.385 up | | 2.165 down | Zn2Cys6 transcriptional regulator |
| 106259 | 0.00106 | 8.18287 | | 6.83825 | | 5.6 | 5.991 up | | 2.359 up | | 2.539 down | Zn2Cys6 transcriptional regulator |
| 109748 | 0.000291 | 12.04081 | | 11.80038 | | 10.59423 | 2.725 up | | 2.307 up | | 1.181 down | Zn2Cys6 transcriptional regulator |
| 105784 | 0.00183 | 8.10195 | | 6.35934 | | 5.31537 | 6.899 up | | 2.061 up | | 3.346 down | Zn2Cys6 transcriptional regulator |
| 111515 | 0.00057 | 8.58534 | | 6.5735 | | 5.74359 | 7.168 up | | 1.777 up | | 4.032 down | Zn2Cys6 transcriptional regulator |
| 105255 | 0.0015 | 9.45529 | | 8.56329 | | 7.98253 | 2.775 up | | 1.495 up | | 1.855 down | Zn2Cys6 transcriptional regulator |
| 112560 | 0.00467 | 10.08637 | | 8.78803 | | 8.68841 | 2.635 up | | 1.071 up | | 2.459 down | Zn2Cys6 transcriptional regulator |
| 123510 | 0.00158 | 10.54866 | | 8.36176 | | 8.26753 | 4.860 up | | 1.067 up | | 4.553 down | Zn2Cys6 transcriptional regulator |
| 103034 | 0.0102 | 8.45035 | | 6.60704 | | 6.57067 | 3.679 up | | 1.025 up | | 3.588 down | Zn2Cys6 transcriptional regulator |
| 103230 | 0.000661 | 11.41877 | | 12.94278 | | 10.45623 | 1.948 up | | 5.604 up | | 2.875 up | Zn2Cys6 transcriptional regulator |
| 49372 | 0.00347 | 13.20921 | | 13.6316 | | 12.28705 | 1.894 up | | 2.539 up | | 1.340 up | Zn2Cys6 transcriptional regulator |
| 56077 | 0.00696 | 12.22824 | | 13.10296 | | 11.31115 | 1.888 up | | 3.462 up | | 1.833 up | Zn2Cys6 transcriptional regulator |
| 68455 | 0.00121 | 10.9018 | | 11.36549 | | 10.01191 | 1.853 up | | 2.555 up | | 1.379 up | Zn2Cys6 transcriptional regulator |
| 105475 | 0.00166 | 12.37584 | | 13.27909 | | 11.49833 | 1.837 up | | 3.436 up | | 1.870 up | Zn2Cys6 transcriptional regulator |
| 51907 | 0.000256 | 10.12224 | | 12.64593 | | 10.98928 | 1.823 down | | 3.152 up | | 5.750 up | Zn2Cys6 transcriptional regulator |
| 106009 | 0.00238 | 11.46135 | | 12.22994 | | 10.59534 | 1.822 up | | 3.105 up | | 1.703 up | Zn2Cys6 transcriptional regulator |
| 109328 | 0.00193 | 8.39911 | | 10.67434 | | 7.53376 | 1.821 up | | 8.818 up | | 4.840 up | Zn2Cys6 transcriptional regulator |
| 60215 | 0.000206 | 9.62885 | | 11.14961 | | 8.78341 | 1.796 up | | 5.155 up | | 2.869 up | Zn2Cys6 transcriptional regulator |
| 121121 | 0.000586 | 9.73995 | | 11.77707 | | 8.90271 | 1.786 up | | 7.332 up | | 4.104 up | Zn2Cys6 transcriptional regulator |
| 105979 | 0.000243 | 9.24384 | | 10.449 | | 8.41176 | 1.780 up | | 4.104 up | | 2.305 up | Zn2Cys6 transcriptional regulator |
| 105989 | 0.00368 | 4.03289 | | 6.22825 | | 4.86471 | 1.779 down | | 2.573 up | | 4.580 up | Zn2Cys6 transcriptional regulator |
| 78895 | 0.000698 | 11.43866 | | 11.99883 | | 10.62779 | 1.754 up | | 2.586 up | | 1.474 up | Zn2Cys6 transcriptional regulator |
| 54437 | 0.000457 | 11.4335 | | 12.42594 | | 10.66311 | 1.705 up | | 3.393 up | | 1.989 up | Zn2Cys6 transcriptional regulator |
| 22755 | 0.0051 | 13.04396 | | 14.22286 | | 12.29626 | 1.679 up | | 3.801 up | | 2.264 up | Zn2Cys6 transcriptional regulator |
| 121682 | 0.000514 | 11.36897 | | 13.07765 | | 10.64249 | 1.654 up | | 5.408 up | | 3.268 up | Zn2Cys6 transcriptional regulator |
| 109549 | 0.00167 | 7.0163 | | 8.65291 | | 6.29901 | 1.644 up | | 5.112 up | | 3.109 up | Zn2Cys6 transcriptional regulator |
| 105849 | 0.00247 | 9.65292 | | 10.73339 | | 8.95649 | 1.620 up | | 3.426 up | | 2.114 up | Zn2Cys6 transcriptional regulator |
| 111408 | 0.000435 | 9.4082 | | 12.14057 | | 10.10062 | 1.616 down | | 4.112 up | | 6.645 up | Zn2Cys6 transcriptional regulator |
| 62297 | 0.000713 | 11.36745 | | 12.49162 | | 10.68455 | 1.605 up | | 3.499 up | | 2.179 up | Zn2Cys6 transcriptional regulator |
| 69077 | 0.000254 | 8.8349 | | 10.59979 | | 8.18948 | 1.564 up | | 5.315 up | | 3.398 up | Zn2Cys6 transcriptional regulator |
| 82241 | 0.000896 | 8.82723 | | 10.86387 | | 8.20861 | 1.535 up | | 6.299 up | | 4.102 up | Zn2Cys6 transcriptional regulator |
| 57760 | 0.00273 | 11.59077 | | 12.6241 | | 11.01479 | 1.490 up | | 3.051 up | | 2.046 up | Zn2Cys6 transcriptional regulator |
| 60897 | 0.0016 | 11.61882 | | 13.40466 | | 11.05026 | 1.483 up | | 5.113 up | | 3.448 up | Zn2Cys6 transcriptional regulator |
| 67483 | 0.000267 | 8.81534 | | 11.72291 | | 8.2608 | 1.468 up | | 11.020 up | | 7.503 up | Zn2Cys6 transcriptional regulator |
| 60558 | 0.00138 | 9.18226 | | 11.10017 | | 8.63442 | 1.461 up | | 5.524 up | | 3.778 up | Zn2Cys6 transcriptional regulator |
| 60665 | 0.00112 | 7.81112 | | 8.75525 | | 7.31468 | 1.410 up | | 2.714 up | | 1.924 up | Zn2Cys6 transcriptional regulator |
| 22925 | 0.000922 | 13.22738 | | 14.28091 | | 12.74451 | 1.397 up | | 2.900 up | | 2.075 up | Zn2Cys6 transcriptional regulator |
| 121875 | 0.000932 | 7.87434 | | 8.88598 | | 7.46223 | 1.330 up | | 2.682 up | | 2.016 up | Zn2Cys6 transcriptional regulator |
| 3449 | 0.00111 | 10.51593 | | 12.65909 | | 10.91741 | 1.320 down | | 3.344 up | | 4.417 up | Zn2Cys6 transcriptional regulator |
| 76039 | 0.0000616 | 9.16824 | | 12.01768 | | 9.56712 | 1.318 down | | 5.466 up | | 7.207 up | Zn2Cys6 transcriptional regulator |
| 2096 | 0.000821 | 9.24854 | | 11.32206 | | 8.88235 | 1.288 up | | 5.425 up | | 4.209 up | Zn2Cys6 transcriptional regulator |
| 65133 | 0.00888 | 7.83134 | | 9.00485 | | 7.46761 | 1.286 up | | 2.902 up | | 2.255 up | Zn2Cys6 transcriptional regulator |
| 21997 | 0.000476 | 8.64819 | | 11.53003 | | 9.00044 | 1.276 down | | 5.774 up | | 7.370 up | Zn2Cys6 transcriptional regulator |
| 70071 | 0.000796 | 11.05233 | | 12.36349 | | 10.72012 | 1.258 up | | 3.123 up | | 2.481 up | Zn2Cys6 transcriptional regulator |
| 106476 | 0.00724 | 7.40435 | | 8.80438 | | 7.07673 | 1.254 up | | 3.311 up | | 2.639 up | Zn2Cys6 transcriptional regulator |
| 76705 | 0.00192 | 11.75317 | | 13.26349 | | 11.4421 | 1.240 up | | 3.534 up | | 2.848 up | Zn2Cys6 transcriptional regulator |
| 3605 | 0.00445 | 11.24153 | | 12.41929 | | 10.94475 | 1.228 up | | 2.778 up | | 2.262 up | Zn2Cys6 transcriptional regulator |
| 59067 | 0.000306 | 9.94209 | | 12.51338 | | 10.23403 | 1.224 down | | 4.854 up | | 5.943 up | Zn2Cys6 transcriptional regulator |
| 79871 | 0.000176 | 11.56678 | | 12.62395 | | 11.292 | 1.209 up | | 2.517 up | | 2.080 up | Zn2Cys6 transcriptional regulator |
| 4885 | 0.000603 | 11.27812 | | 13.05067 | | 11.50441 | 1.169 down | | 2.920 up | | 3.416 up | Zn2Cys6 transcriptional regulator |
| 112129 | 0.00406 | 4.67198 | | 6.43435 | | 4.46162 | 1.156 up | | 3.925 up | | 3.392 up | Zn2Cys6 transcriptional regulator |
| 108381 | 0.00158 | 9.11244 | | 10.39307 | | 8.91634 | 1.145 up | | 2.783 up | | 2.429 up | Zn2Cys6 transcriptional regulator |
| 108872 | 0.00181 | 10.47765 | | 11.7925 | | 10.29358 | 1.136 up | | 2.826 up | | 2.487 up | Zn2Cys6 transcriptional regulator |
| 105520 | 0.000528 | 8.37688 | | 11.57543 | | 8.53369 | 1.114 down | | 8.234 up | | 9.180 up | Zn2Cys6 transcriptional regulator |
| 121164 | 0.000686 | 9.97386 | | 11.41136 | | 9.82489 | 1.108 up | | 3.003 up | | 2.708 up | Zn2Cys6 transcriptional regulator |
| 121412 | 0.000266 | 10.1461 | | 13.85889 | | 10.2877 | 1.103 down | | 11.886 up | | 13.111 up | Zn2Cys6 transcriptional regulator |
| 104994 | 0.00261 | 9.38126 | | 10.93499 | | 9.48691 | 1.075 down | | 2.728 up | | 2.935 up | Zn2Cys6 transcriptional regulator |
| 111013 | 0.00134 | 9.2772 | | 11.19081 | | 9.20431 | 1.051 up | | 3.962 up | | 3.767 up | Zn2Cys6 transcriptional regulator |
| 70547 | 0.000497 | 9.70384 | | 11.60629 | | 9.63268 | 1.050 up | | 3.927 up | | 3.738 up | Zn2Cys6 transcriptional regulator |
| 67339 | 0.00388 | 9.91631 | | 11.77267 | | 9.95679 | 1.028 down | | 3.520 up | | 3.620 up | Zn2Cys6 transcriptional regulator |
| 106244 | 0.00125 | 10.67611 | | 12.21997 | | 10.70204 | 1.018 down | | 2.863 up | | 2.915 up | Zn2Cys6 transcriptional regulator |
| 108013 | 0.0221 | 4.27881 | | 6.32892 | | 4.26667 | 1.008 up | | 4.176 up | | 4.141 up | Zn2Cys6 transcriptional regulator |
| 112036 | 0.000839 | 8.75043 | | 10.33227 | | 8.73776 | 1.008 up | | 3.019 up | | 2.993 up | Zn2Cys6 transcriptional regulator |
| 122208 | 0.000247 | 12.80324 | | 13.8498 | | 7.9798 | 28.313 up | | 58.485 up | | 2.065 up | Zn2Cys6 transcriptional regulator XYR1 |
| 26163 | 0.000127 | 9.09819 | | 10.64885 | | 4.60323 | 22.548 up | | 66.056 up | | 2.929 up | Zn2Cys6 transcriptional regulator CLR-2 |
| 70197 | 0.000482 | 11.1473 | | 10.42774 | | 6.73244 | 21.330 up | | 12.953 up | | 1.646 down | Zn-dependent ß-lactamase |
| 59642 | 0.000242 | 11.28962 | | 11.57032 | | 8.93982 | 5.097 up | | 6.192 up | | 1.214 up | α/ß hydrolase lipase/epoxide hydrolase |
| 69529 | 0.0000354 | 5.54935 | | 12.46494 | | 9.5534 | 16.044 down | | 7.524 up | | 120.725 up | α-ketoglutarate dependent (FeII) dioxygenase |
| 58580 | 0.000661 | 3.93374 | | 7.915 | | 4.34412 | 1.329 down | | 11.883 up | | 15.793 up | α-ketoglutarate dependent (FeII) oxygenase |
| 5337 | 0.0000851 | 9.06058 | | 10.7402 | | 5.91865 | 8.827 up | | 28.276 up | | 3.203 up | γ-glutamyltranspeptidase |
| 5645 | 0.00227 | 10.48423 | | 12.467 | | 9.72372 | 1.694 up | | 6.695 up | | 3.952 up | γ-glutamyltranspeptidase |
