# Supplementary material for: Comparative analysis of the Trichoderma reesei transcriptome during growth on the cellulase inducing substrates wheat straw and lactose
Source: Biotechnol Biofuels. 2013 Sep 9;6:127. doi: 10.1186/1754-6834-6-127 (PMC3847502; doi:10.1186/1754-6834-6-127)
Supplement: Additional file 3: Table S3 — Concentration of iron in the culture supernatant on lactose and wheat straw. [file 1754-6834-6-127-S3.docx]

**Supplementary Table S3**: Concentration of iron in the culture supernatant on lactoseand wheat straw

| Time |  | Lactose | | wheat straw | |
| --- | --- | --- | --- | --- | --- |
| ____________________ | ____________ | __________ | ________ | ________ | _______ |
|  |  | Fe [ppm] | S.D. | Fe [ppm] | S.D. |
| Medium only |  | 1.43 | ± 0.019 | 0.23 | ± 0.015 |
| Inoculated medium (t=0) |  | 1.44 | ± 0.033 | 0.28 | ± 0.0019 |
| Time of RNA extraction* | not inoculated | 1.46 | ± 0.05 | 0.28 | ± 0.006 |
|  | inoculated | 1.38 | ± 0.006 | 0.064 | ± 0.0099 |

* 28 h on lactose, and 50 h on wheat straw; values are means of two biological replicates
