# Supplementary material for: Comparative analysis of the Trichoderma reesei transcriptome during growth on the cellulase inducing substrates wheat straw and lactose
Source: Biotechnol Biofuels. 2013 Sep 9;6:127. doi: 10.1186/1754-6834-6-127 (PMC3847502; doi:10.1186/1754-6834-6-127)
Supplement: Additional file 6: Table S6 — Presence of the XYR1 binding consensus motif in the first 1000 bp upstream of the start codon in the genes upregulated in the Δxyr1 mutant strain. [file 1754-6834-6-127-S6.docx]

**Supplementary Table S6.**

Presence of the XYR1 binding consensus motif in the first 1000 bp upstream of the start codon in the genes upregulated in the *Δxyr1* mutant strain.

| Protein ID | protein function | number of motifs |
| --- | --- | --- |
| 56996 | GH5 ß-mannanase MAN1 | 6 |
| 57857 | GH2 ß-mannosidase | 5 |
| 124043 | GH18 chitinase CHI18-14 | 4 |
| 69493 | GH92 α-mannosidase | 4 |
| 80833 | GH18 chitinase CHI18-5 | 3 |
| 72632 | GH27 α-galactosidase AGL1 | 2 |
| 42152 | GH75 chitosanase | 2 |
| 122495 | GH76 α-mannanase | 2 |
| 27259 | GH27 α-galactosidase | 2 |
| 56448 | GH18 chitinase CHI18-11 | 2 |
| 69245 | GH2 ß-mannosidase | 1 |
| 124016 | GH36 α-galactosidase | 1 |
| 75015 | GH27 α-galactosidase | 1 |
| 68347 | GH18 chitinase CHI18-16 | 0 |
| 74198 | GH92 α-mannosidase | 0 |
| 59082 | CH18 chitinase CHI18-2 | 0 |
